# Peptide‐Carbazolyl Cyanobenzene Conjugates: Enabling Biomolecule Functionalization via Photoredox and Energy Transfer Catalysis

**DOI:** 10.1002/anie.202507602

**Published:** 2025-06-30

**Authors:** Xing‐Yu Liu, Wei Cai, Anne‐Sophie Chauvin, Beat Fierz, Jerome Waser

**Affiliations:** ^1^ Laboratory of Catalysis and Organic Synthesis (LCSO) Ecole Polytechnique Fédérale de Lausanne, EPFL Lausanne 1015 Switzerland; ^2^ Laboratory of Biophysical Chemistry of Macromolecules (LCBM) Ecole Polytechnique Fédérale de Lausanne, EPFL Lausanne 1015 Switzerland; ^3^ Group of Coordination Chemistry Institut des Sciences et Ingénierie Chimiques, Ecole Polytechnique Fédérale de Lausanne, EPFL Lausanne 1015 Switzerland

**Keywords:** Biomolecule functionalization, Labeling, Peptides, Photocatalysts

## Abstract

Since their discovery in 2012, carbazolyl (iso)phthalonitrile (Cz(I)PN) derivatives have found significant applications as photocatalysts (PCs) in organic chemistry. Herein, we introduce two efficient methods for incorporating carbazolyl cyanobenzenes into various peptide sequences. The first method involves a photomediated decarboxylative functionalization of the C‐terminus of peptides, leading to the formation of various carbazolyl benzonitrile (CzBN) derivatives. The second method exploits a cysteine‐selective S_N_Ar reaction on a fluorinated arene precursor, resulting in novel peptide‐3CzIPN (triscarbazolyl‐isophthalonitrile) conjugates. Both types of conjugates maintain delayed fluorescence properties, exhibit similar or wider redox potential, and possess higher excited state energy when compared to currently used cyanoarenes. We demonstrated the photocatalytic activity of these conjugates first through a photo‐mediated peptide C‐terminal decarboxylative alkynylation. Then, water‐soluble peptide conjugates were used to catalyze a thiol‐ene reaction on cysteine in aqueous media. Finally, we achieved protein labeling via aryl azide excitation both in vitro and at the cellular level using peptide‐CzIPN conjugates. By incorporating a peptide ligand of the protein integrin α_v_β_3_, proximity‐driven labeling next to this target was realized by aryl azide excitation in living cells, showing an excellent overlap with antibody‐based imaging. These findings reveal the potential of cyanoarene‐peptide conjugates for proximity‐driven photochemistry in a complex biological context.

## Graphical abstract

We introduce two methods for incorporating carbazolyl cyanobenzenes onto peptides: a C‐terminus decarboxylative functionalization and a Cys‐selective S_N_Ar reaction. Both conjugates exhibit excellent photoredox/energy‐transfer properties, enabling their use for C‐terminal alkynylation and Cys‐selective thiol‐ene reactions on peptides, as well as proximity‐driven labeling next to integrin αvβ3 by aryl azide excitation in living cells.

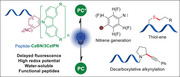

## Introduction

Visible‐light‐mediated biomolecule functionalization has emerged as a powerful tool for achieving spatiotemporal control of reactions within complex biological systems.^[^ ^1^, ^2^, ^3^, ^4^, ^5^ ^]^ Compared to traditional UV‐induced transformations, the combination of visible light and photocatalysts (PCs) enables the generation of highly reactive intermediates under milder conditions in close proximity to the excited PCs. The PC can then be localized in a desired site on a biomolecule using a binding small molecule,^[^ ^6^, ^7^, ^8^ ^]^ a peptide, or a protein/antibody.^[^ ^4^, ^9^, ^10^, ^11^, ^12^ ^]^ To facilitate site‐specific functionalization in intricate and highly diluted biological environments, PCs have been designed with a wide redox potential window for single‐electron transfer (SET) and a long‐lived, high‐energy excited state for efficient energy transfer (EnT). In contrast, the use of non‐toxic organic photosensitizers is much less exploited. Recent studies have demonstrated the potential of organic dyes, including rhodamine,^[^ ^13^ ^]^ fluorescein,^[^ ^14^ ^]^ flavin,^[^ ^15^, ^16^ ^]^ porphyrin,^[^ ^17^ ^]^ and Eosyn Y^[^ ^18^ ^]^ in enabling biomolecule functionalization in living systems through visible light excitation. Given the excellent biocompatibility of organic photosensitizers, these recent pioneering breakthroughs are promising and demonstrate the urgent need for novel organic dyes with unique photophysical properties and improved biocompatibility.

**Scheme 1:**
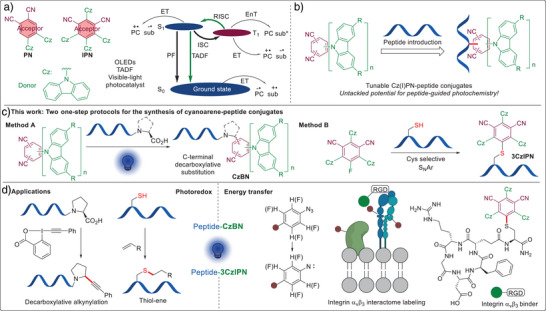
a) Structure of Cz(I)PN derivatives and energy diagram for their use as PCs in ET (electron transfer) and EnT based transformations. b) Peptide‐Cz(I)PN‐conjugates: unprecedented hybrid catalysts for peptide‐directed photochemistry. c) Synthesis of CzBN‐peptide conjugates via decarboxylative arylation at peptide C‐terminus (**Method A**) and 3CzIPN‐peptide conjugates via S_N_Ar on cysteine (**Method B**). d) Application to biomolecule functionalization.

Carbazolyl (iso)phthalonitrile (Cz(I)PN) derivatives are a class of organic dyes with carbazoles as electron donors and a dicyanobenzene as an electron acceptor (Scheme 1a).^[^ ^19^ ^]^ The small energy gap between the singlet and triplet excited states of these compounds enhances reversible intersystem crossing (RISC), making them suitable for use in organic light‐emitting diodes (OLEDs) with thermally activated delayed fluorescence (TADF). Excellent quantum yield (up to 94.6%), a long‐excited state lifetime (up to 5.1 µs), and a wide redox potential window make them also good PCs in different light‐driven transformations.^[^ ^20^, ^21^, ^22^, ^23^, ^24^ ^]^ Additionally, the fine‐tuning of the PCs can be easily realized by adding electron‐withdrawing and electron‐donating groups on the carbazole motifs. Since the seminal discovery by Zhang in 2016,^[^ ^25^ ^]^ over 20 structural variants of CzIPN have been investigated for their photocatalytic properties. These derivatives have shown potential not only as substitutes for traditional iridium (Ir) and ruthenium (Ru) catalysts but also have enabled novel transformations for photo‐mediated small molecule functionalization.

Despite the extensive use of Cz(I)PN derivatives in SET and EnT catalysis for small molecule functionalization, their applications in biological contexts have been limited.^[^ ^26^ ^]^ The incorporation of peptide sequences interacting with biological targets would enable the use of Cz(I)PN for proximity‐driven reactions, with the added advantage of increasing their water solubility (Scheme 1b). Surprisingly, such a strategy has not been reported yet. Herein, we describe two efficient one‐step approaches to synthesize cyanoarenes bearing functional peptide sequences (Scheme 1c). The first method (**Method A**) involves the photo‐mediated decarboxylative substitution of a cyanide group in Cz(I)PN. A broad scope of carbazolyl benzonitriles (CzBNs) was obtained, but the reaction resulted in the formation of diastereoisomers at the newly formed benzylic center at the C‐terminus. This is not expected to have an impact on biological applications if the C‐terminus is not part of the binding interactions with an eventual target. Nevertheless, to prevent the generation of two diastereoisomers and introduce the dye on other positions of the peptides, we developed a cysteine‐selective S_N_Ar reaction using a fluoroisophthalonitrile precursor, resulting in the formation of a triscarbazolyl‐isophthalonitrile (3CzIPN) dye (**Method B**). Both the CzBN and the 3CzIPN conjugates exhibit photophysical properties and redox potentials that are similar to or superior to the non‐modified 4CzIPN. These properties make them suitable as PCs in various photo‐mediated biomolecule functionalizations, both in vitro and at the cellular level. We demonstrated the first successful decarboxylative alkynylation and thiol‐ene reactions on peptides. As a first example in a complex biological setting, we then realized a proximity‐driven labeling next to integrin α_v_β_3_ via aryl azide excitation enabled by a binding peptide‐CzIPN conjugate, which showed an excellent overlap with antibody‐based imaging.

## Results and Discussion

### Discovery and Synthesis of the New Dyes

**Scheme 2:**
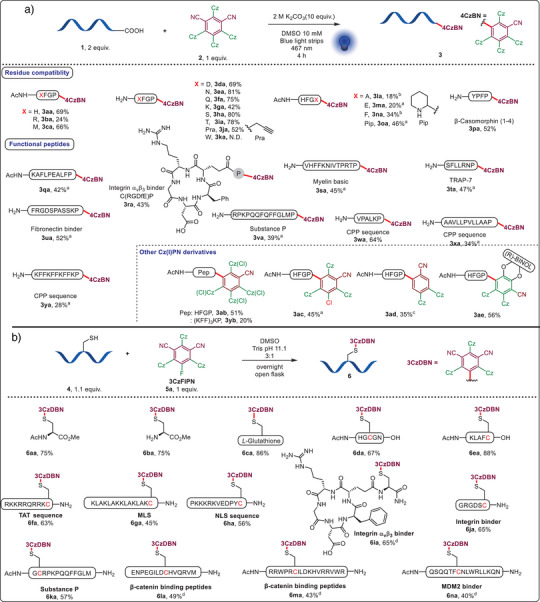
Scope of the C‐terminal decarboxylative substitution and Cys‐selective S_N_Ar. a. Decarboxylative arylation. b. Cys‐selective S_N_Ar. The reactions were performed on a 0.01 mmol scale, isolated yields are given. ^a^1.0 equiv. of peptide and 1.1 equiv. of 3CzFIPN were used. The d.r. of the product was not determined. The reactions were performed on a 0.01 mmol scale, isolated yields are provided. Cz(Cl): 3,6‐dichlorocarbazoyl. (R)‐BINOL: (R)‐1,1′‐Bi‐2‐naphthol. ^a^The reactions were conducted overnight. ^b^A 440 nm Kessil lamp (40 W, intensity: 25%) was used as the blue LED source for 1 h. ^c^A 390 nm Kessil lamp (40 W, intensity: 25%) was used as the light source for 2 h. ^d^1.0 equiv. of peptide and 1.1 equiv. of 3CzFIPN were used.

In 2019, König's group reported a decarboxylative photosubstitution of dicyanobenzene‐based PCs under blue light irradiation.^[^ ^27^, ^28^ ^]^ The monocyanobenzene products were still reducing PCs.^[^ ^29^, ^30^ ^]^ However, amino acids or peptides were not investigated as partners in this transformation. During our studies on the decarboxylative alkynylation of peptide C‐termini,^[^ ^31^, ^32^ ^]^ we observed similar decarboxylative photosubstitution products, which motivated us to optimize this transformation. Gratifyingly, the radical substitution could be achieved by shedding blue light on a mixture of peptide **1aa**, 4CzIPN, and (see Table S1 for reaction optimization). On tetrapeptides **1aa‐ap** bearing a Pro at the C‐terminus,^[^ ^31^ ^]^ Arg (**3ba**), Met (**3ca**), Asp (**3da**), amides (**3ea**, **3fa**), Lys (**3ga**), alcohols (**3ha**, **3ia**), a terminal alkyne (**3ja**), as well as Tyr (**3pa**) side chains were tolerated, resulting in 24%–81% yield of the corresponding products (Scheme 2a). Trp (**3ka**) was not compatible with the photoredox conditions. Concerning C‐terminal amino acids, Glu and pipecolic acid could be converted to the desired decarboxylation products **3ma** and **3oa** in 20% and 46% yield. For Ala (**3la**) and Phe (**3na**), it was necessary to use a stronger 440 nm Kessil lamp. More complex peptides were investigated next. β‐casomorphin (1–4),^[^ ^33^ ^]^ containing a free Tyr and N‐terminus, was transformed into conjugate **3pa** in 52% yield. Decamer **1q**, with a free Glu residue, was selectively functionalized at the C‐terminus, yielding **3qa**. Other modified bioactive peptides include an α_v_β_3_ integrin binding cyclic peptide (**3ra**);^[^ ^34^ ^]^ myelin basic protein fragment 87–99 (**3sa**),^[^ ^35^ ^]^ TRAP‐7 (**3ta**),^[^ ^36^ ^]^ a fibronectin binding inhibitor (**3ua**);^[^ ^37^ ^]^ and the neurotransmitter substance P (**3va**). We also synthesized conjugates **3wa**‐**3ya** containing three cell‐penetrating peptide sequences (VPALK,^[^ ^38^ ^]^ AAVLLPVLLAA,^[^ ^39^ ^]^ and (KFF)_3_K^[^ ^40^ ^]^). Finally, we extended this reaction to 4ClCzIPN (**2b**), 3CzClIPN^[^ ^41^ ^]^ (**2c**), 2CzPN (**2d**), and chiral BINOL‐derivative **2e**,^[^ ^42^ ^]^ to give **3ab‐e** and **3yb** in 20%–56% yield. Overall, the method could be applied to a large scope of peptides and Cz(I)PN derivatives. Nevertheless, the moderate incorporation efficiency for longer peptide sequences and the generation of diastereomeric mixtures can be a drawback for certain applications. Inspired by the reported synthesis of 4CzIPN,^[^ ^43^ ^]^ we attempted to use Cys as a nucleophile in an S_N_Ar reaction (Scheme 2b). To our delight, this reaction could be achieved in an open flask with DMSO and a Tris buffer as reaction solvents (see Table S2 for reaction optimization). From the amino acid cysteine to pentameric peptides, the introduction of 3CzIPN occurred in 67%–88% yield with exclusive formation of the desired products **6aa**‐**6ea**. Cys within a cationic cell penetrating peptide (CPP) TAT,^[^ ^44^ ^]^ a mitochondria localization sequence (MLS),^[^ ^45^ ^]^ a nucleus localization sequence (NLS),^[^ ^46^ ^]^ an αvβ3 integrin binder, Substance P and two α‐helical peptides binding to the protein β‐catenin^[^ ^47^ ^]^ and MDM2^[^ ^48^ ^]^ reacted efficiently to give conjugates **6fa‐6na** in 40%–65% isolated yield.

### Dyes Photophysical Properties and Photocatalytic Activity

**Scheme 3:**
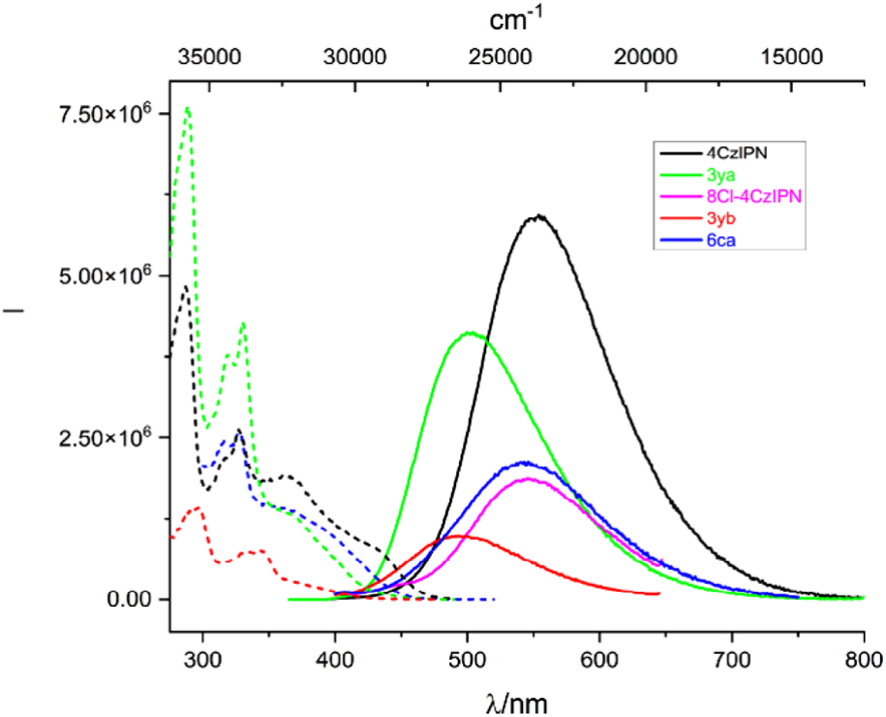
Corrected excitation and emission spectra of the different CzPN‐peptide conjugates at room temperature, in DMSO (10 µM). Solid lines: emission spectra with λ_ex_ = 330 nm Dashed lines: excitation spectra, λ_em_ corresponds to the maximum of the emission spectra; see Table S3 for exact values.

With various Cz(I)PN‐peptide conjugates in hand, we determined their photophysical properties. This was an essential step to assess if the conjugation to the peptide was not detrimental to their catalytic properties. In addition, there was precedence that the substitution of one cyanide with an alkyl group still led to functional catalysts,^[^ ^29^, ^30^ ^]^ the effect of replacing a carbazole substituent by a sulfur group had never been studied before. The absorption spectra feature several bands located below 400 nm, which are attributed to the donor–acceptor π→π* transition of the carbazole‐cyanobenzene.^[^ ^49^, ^50^ ^]^ An additional band extending to 450 nm is observed for all 3/4 carbazolyl conjugates, in DMSO or in water, matching with the excitation spectra (Scheme 3 and Figure S1). In DMSO, the excitation spectra of 4CzlPN and its peptide conjugates show three distinct peaks at 290, 318, and 330 nm, along with a broad band extending up to 350 nm. The spectra of 8Cl‐4CzlPN and its conjugates are blue‐shifted by approximately 1150–1450 cm⁻¹ (Scheme 3). Additionally, the emission maximum of 8Cl‐4CzlPN is blue‐shifted by around 334 cm⁻¹ compared to 4CzlPN. The emission of **6ca** is only slightly shifted (368 cm⁻¹) compared to its parent dye 4CzlPN, while **3ya** shows a more pronounced blue shift of 1884 cm⁻¹. Similarly, the shift observed between **3yb** and 8Cl‐4CzlPN is 1833 cm⁻¹. A slight red shift was noted for the **3yb** and **6ca** conjugates when the solvent was changed from DMSO to water (Figure S2), likely due to intramolecular charge transfer (ICT) influenced by the hydrophobic Cz(I)PN cores.^[^ ^49^ ^]^ Water, known to quench luminescence due to competition with non‐radiative deactivation pathways, caused a decrease in both emission and excitation intensities, although they remained relatively strong. The lifetimes of the different compounds were studied at 330 and 380 nm to broaden applicability for biological studies.

The results (Table S2) showed that decay curves fit best with a double exponential model. The first decay was minimally affected compared to the unsubstituted dyes (0.99–1.72 vs. 1.52 µs for 4CzIPN and its derivatives), while the second was shorter (8.60–8.81 vs. 11.6 µs for 4CzIPN and its derivatives). The conjugate lifetimes were consistent across different solvents and peptide conjugations. Notably, in water, **3yb** showed increased lifetimes, suggesting small aggregate formation. In DMSO, the quantum yield of unmodified 4CzIPN is 19.7% under excitation at 330 nm (Tables S3 and S4), aligning with the 10% yield previously reported in aerated toluene.^[^ ^19^ ^]^ The quantum yield of **3ya** in DMSO remained close, while other peptide conjugates showed yields around 7%–8%. Under 380 nm excitation, quantum yields were slightly lower. Notably, these compounds still exhibited good quantum yields in water, providing potential for biological applications.

**Scheme 4:**
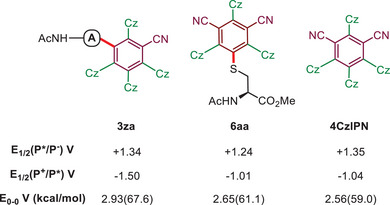
Structure and redox potentials of CzBN and CzIPN‐peptide conjugates. 4CzIPN and Ir PC are shown as a comparison. See Supporting Information for a full list of redox values (Table S5).

To further compare the redox potentials and excited‐state energy of the peptide conjugates with unmodified 4CzIPN and a well‐known Ir complex [Ir{dF(CF_3_)ppy}_2_(dtbpy)]PF_6_, cyclic voltammetry (CV) experiments were conducted (Scheme 4; Table S5), and excited state potentials were estimated using the Rehm–Weller equation. In the excited state, both conjugates exhibit similar or even stronger oxidative and reductive capabilities when compared to 4CzIPN and the Ir catalysts. They also possess high excited states energy (>60 kcal mol^−1^), with **3za** notably reaching 67.6 kcal mol^−1^.^[^ ^28^ ^]^

**Scheme 5:**
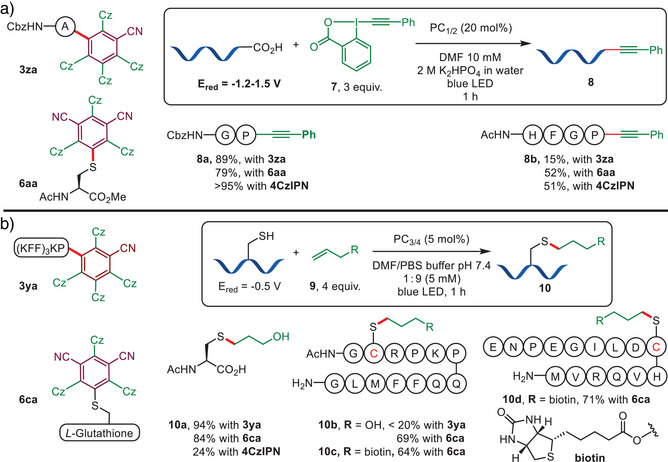
Application of CzBN‐ and CzIPN‐peptide conjugates in a) the photo‐mediated decarboxylative alkynylation and b) the thiol‐ene reactions. Yields were determined based on the integration of HPLC‐UV signals.

The broader redox‐potential window, long excited state lifetimes, and higher excited state energy of CzBN and CzIPN derivatives all indicated that they kept excellent photophysical properties to be used as PCs. Furthermore, their enhanced solubility in aqueous media while keeping sufficient photoactivity promised a high potential for the photo‐functionalization of biomolecules. First, we attempted the photo‐mediated C‐terminal decarboxylative alkynylation of peptides originally catalyzed by 4CzIPN (Scheme 5a). Compared to 4CzIPN, conjugates **3za** and **6aa** produced the alkynylated product **8a** with a slight decrease of yield for the protected dipeptide Cbz‐GP‐OH. We further tested the catalytic reactivity of the conjugates on a longer tetramer peptide, AcHFGP‐OH. The alkynylation reaction (**8b**) proceeded with both conjugates **3za** and **6aa**, achieving HPLC yields of 15% and 51%, respectively. The latter is comparable to the yield obtained with non‐modified 4CzIPN (52%). Encouraged by these results, we conducted this reaction in aqueous media using water‐soluble conjugates **3ya** and **6ca**. Unfortunately, no decarboxylation occurred with either catalyst using a simple dipeptide as the model substrate, likely due to differences in redox potential for the dyes in the two reaction solvents.

We next explored the feasibility of conducting a visible light‐mediated thiol‐ene reaction in aqueous media using CzBN and CzIPN conjugates as PCs (Scheme 5b). To date, there are only a few examples of thiol‐ene reactions carried out in aqueous media under visible light excitation, using either ruthenium or quinolinone‐based PCs,^[^ ^51^, ^52^ ^]^ and cyanoarene‐type dyes have not yet been used in this context. We were pleased to find that the desired thiol‐ene adduct could be efficiently synthesized from Ac‐Cys‐OH and allyl alcohol in aqueous media, using conjugates **3ya** and **6ca** as PCs. In contrast, 4CzIPN itself exhibited significantly lower catalytic reactivity due to its limited solubility in water. We further examined the compatibility of this reaction with longer peptide sequences. Conjugate **6ca** demonstrated superior catalytic activity compared to **3ya**, as the latter led to increased oxidation of the peptides during the photocatalytic process. Consequently, **6ca** was selected as the preferred PC for thiol‐ene reactions involving longer peptides. This reaction showed good tolerance for a variety of unprotected side‐chain residues, yielding the desired thiol‐ene adduct **10b** with high efficiency. Moreover, the reaction was not limited to allyl alcohol **9a**; a biotin‐containing alkene **9b** can also be incorporated into peptides, yielding the thiol‐ene product **10c** and **10d**.

### Proof of Concept Results on Proteins

**Scheme 6:**
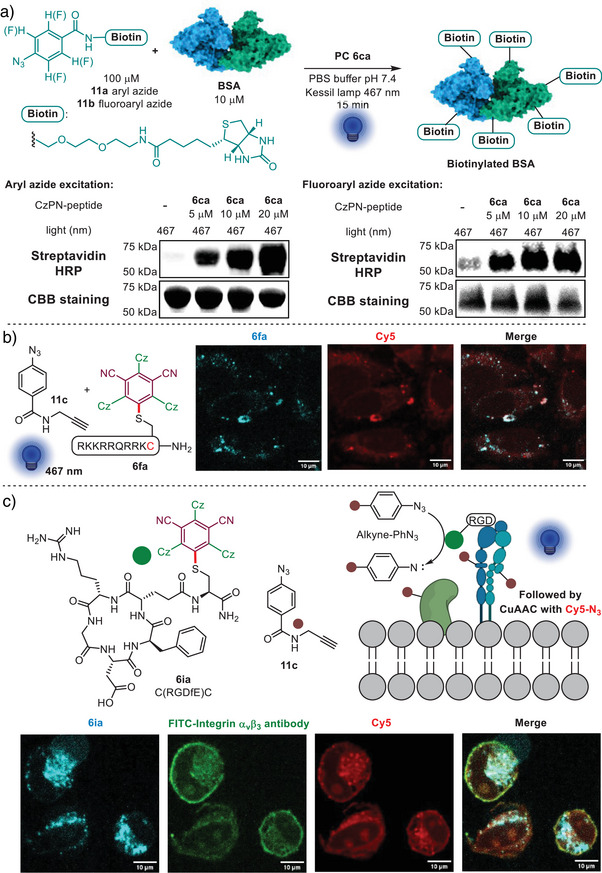
Aryl azide excitation for in vitro and cellular protein labeling. A Kessil lamp at 467 nm (40 W, intensity: 100%) was used as the light source. a) Protein labeling in vitro via aryl azide excitation using CzPN‐peptide conjugate **6ca**. b) Labeling using cell‐penetrating 3CzIPN conjugate **6fa** via nitrene formation. Cell line: HeLa. DIC imaging in Supporting Information Section 8.7.2. c) Peptide‐guided proximity‐driven labeling of integrin α_v_β_3_ with conjugate **6ia** (10 µM) via nitrene formation followed by IF analysis using FITC‐integrin α_v_β_3_ monoclonal antibody to confirm target specific labeling. Cell line: A549. DIC imaging in Supporting Information Section 8.7.3. Scale bar: 10 µm. For quantitative image analysis, the following thresholds were used: FITC‐integrin α_v_β_3_ monoclonal antibody channel: 741. Cy5 channel: 109. Pearson's coefficient for FITC‐integrin α_v_β_3_ monoclonal antibody and Cy5: 0.628.

Having successfully applied the new PCs for the functionalization of peptides, we then turned to the modification of more complex proteins. We chose the photocatalytic generation of nitrenes from aryl azides, which is an established method for the functionalization of biomolecules.^[^ ^7^, ^8^, ^10^, ^15^, ^53^ ^]^ Aryl azides **11** have a triplet state energy of 43.9 kcal mol^−1^, which is theoretically accessible through EnT using the peptide conjugates (excited energy above 60.0 kcal mol^−1^). This would further demonstrate that the new cyanoarene conjugates are not only competent for photoredox but also energy‐transfer catalysis. We were pleased to observe the successful generation of triplet nitrenes for both an aryl azide and a perfluorinated aryl azide using either 4CzBN **3za** or 3CzIPN conjugates **6aa** under 467 nm irradiation from a Kessil lamp (see Supporting Information Section 8.3.2 for the result and discussion of the photoconversion of aryl/fluoroaryl azides enabled by 4CzBN and 3CzIPN). Notably, 3CzIPN conjugates exhibited higher excitation efficiency. Encouraged by these results, we further explored the potential of in vitro protein labeling enabled by these dye conjugates (Scheme 6a). Considering the lower excitation efficiency when compared to Ir or Osmium catalysts, we conducted the reaction with higher catalyst concentration on bovine serum albumin (BSA) with a biotin‐conjugated aryl azide probe **11a** (100 µM) and 3CzIPN **6ca** (10 µM). The photolabeling reactivity of the conjugates was assessed via Western blotting of the reaction mixture with HRP‐conjugated streptavidin to detect the amount of biotinylated BSA. A notable biotinylation level was observed when the reaction was performed in a PBS buffer after only 15 min of irradiation with a 467 nm Kessil lamp (40 W, intensity: 100%) in the presence of **6ca**. The level of protein biotinylation correlated with the concentration of **6ca**. In contrast, direct excitation of BSA with 467 nm light alone resulted in minimal labeling. Upon applying perfluorinated aryl azide **11b**, a compound known to generate nitrene species with extended lifetimes for protein labeling with tunable labeling radii,^[^ ^10^ ^]^ we observed in contrast background reactivity, which is consistent with previous reports.^[^ ^10^, ^11^ ^]^ However, the addition of 3CzIPN **6ca** significantly enhanced BSA labeling efficiency in a dose‐dependent manner, supporting the capability of 3CzIPN conjugates for promoting fluoroaryl azide excitation.

### Proof of Concept Results for Proximity‐driven Labeling in Living Systems

With the success of in vitro protein labeling via aryl azide excitation, we extended this method to living cell systems with the goal of exploiting the potential for proximity‐driven labeling offered by the binding peptide. This approach would differ significantly compared to previous approaches based on localization‐specific small molecule dyes^[^ ^15^ ^]^ or antibody‐bound catalysts.^[^ ^7^, ^10^, ^17^ ^]^ Peptide sequences allow us potentially to target both intra‐ and extracellular targets.

To validate the potential of targeting intracellular targets, we used CPP‐3CzIPN conjugate **6fa** as the photosensitizer together with azide **11c**, which is small enough to cross the cell membrane (Scheme 6b; DIC imaging in Supporting Information Section 8.7.2). We observed significant fluorescent pattern overlap between the nitrene labeling region (revealed through CuAAC between a Cy5 azide reagent and the terminal alkyne on **11c**) and the CzIPN conjugate region inside HeLa cells (see Supporting Information Section 8.7 for experimental details).

As an extracellular target of high biological relevance, we chose integrins, which are a class of cell adhesion receptors playing a crucial role in mediating signal transduction between cells and the extracellular matrix (ECM). Notably, the αvβ3 integrin subtype is frequently overexpressed in different cancer cells. Consequently, it has emerged as a promising therapeutic target for the development of cancer treatments.^[^ ^54^, ^55^ ^]^ To further underscore the application of peptide sequences on 3CzIPN to realize protein specific labeling, we used the modified integrin αvβ3 binder **6ia** to probe the interactome of integrin αvβ3 (Scheme 6c; DIC imaging in Supporting Information Section 8.7.3). We selected the A549 cell line, known for its high expression of integrin αvβ3. Considering the influence of 3CzIPN conjugation on the binding affinity of RGD peptides, as well as previous reports on FITC‐labeled RGD peptides, ^[^ ^56^, ^57^ ^]^ a final concentration of 10 µM was chosen for the cell‐labeling experiments. The cells were first incubated with the integrin αvβ3 binder **6ia** for 1 h, followed by washing and subsequent incubation with the aryl azide probe **11c**. After the photoexcitation of aryl azide probe, the cells were fixed and subjected to immunofluorescence (IF) staining with FITC conjugated integrin αvβ3 antibody to determine the localization of the target protein. The labeling region of the aryl azide was visualized through CuAAC between a Cy5 azide reagent and the terminal alkyne on **11c**. We were pleased to observe a strong overlap between the antibody signal and the Cy5 signal, showing that aryl azide excitation had occurred in close proximity to integrin. After the photoreaction, the localization of the PC **6ia** also showed a good overlap with both the andibody and the Cy5 dye, although the signal on the membrane was weaker. This may be due to a fast photo‐bleaching of the dye on the membrane during irradiation. To further confirm the target engagement, we conducted a negative control experiment using HeLa cells, which express low levels of integrin αvβ3 (Figure S6) minimal fluorescence signal was observed in both the antibody and Cy5 channels, supporting the specificity of our probe for integrin αvβ3. In both the cases of intracellular and extracellular aryl azide excitation, cells remained in good viability before and after light irradiation, highlighting the potential of using 3CzIPN‐peptide conjugates in live systems (Figure S7).

## Conclusion

We have developed two efficient strategies to incorporate privileged cyanobenzene organic PCs into functional peptides. The first approach involved a photo‐mediated, C‐terminal selective decarboxylative arylation, enabling the attachment of various peptides to diverse CzBN cores. The second approach leveraged a cysteine‐selective S_N_Ar reaction with 3CzFIPN, which improved reaction efficiency and allowed the formation of a single bioconjugate at any position in the peptide sequence, resulting in unprecedented sulfur‐substituted cyanoarene dyes. The resulting peptide‐CzBN and CzIPN conjugates exhibited strong delayed fluorescence, broad redox potential windows, and high excited state energies. We demonstrated the potential of using these conjugates as PC for biomolecule functionalization through a photo‐mediated decarboxylative alkynylation in organic solvents and a thiol‐ene reaction in aqueous media. Additionally, the conjugated promoted aryl azide excitation, enabling protein labeling both in vitro and at the cellular level. Therefore, the conjugates could be used both for photoredox and energy‐transfer catalysis. Using a peptidic ligand of integrin αvβ3, proximity‐driven labeling of the protein and its interactome became possible via aryl azide excitation. We believe these conjugates will open new avenues for the development of novel photo‐mediated biomolecule functionalizations in biological contexts.^[^ ^58^ ^]^ After these proof of concept results, future work in our laboratory will focus on biological validation in other systems and further quantification of the efficiency and selectivity of the proximity‐driven labeling using proteomic approaches.

## Supporting Information

Supporting information is available as pdf file, including general procedures, HPLC methods, synthesis and characterization for compounds, photophysical properties, electrochemical measurements, biological results X‐ray data and copy of NMR spectra. Raw data for compound characterization will be available free access at https://doi.org/10.5281/zenodo.15572911 after final publication of the work. The authors have cited additional references within the Supporting Information.^[^ ^59^, ^60^, ^61^, ^62^, ^63^, ^64^, ^65^, ^66^ ^]^

## Conflict of Interests

The authors declare no conflict of interest.

## Supporting information



Supporting Information

## Data Availability

The data that support the findings of this study are available in the Supporting Information of this article.
