# Supplementary material for: Peptide‐Carbazolyl Cyanobenzene Conjugates: Enabling Biomolecule Functionalization via Photoredox and Energy Transfer Catalysis
Source: Angew Chem Int Ed Engl. 2025 Jun 30;64(32):e202507602. doi: 10.1002/anie.202507602 (PMC12322649; doi:10.1002/anie.202507602)
Supplement: Supplementary file 1 — Supporting Information [file ANIE-64-e202507602-s001.pdf]

Supporting information

Peptide-Carbazolyl Cyanobenzene Conjugates: Enabling Biomolecule Functionalization via Photoredox and Energy Transfer Catalysis

Xing-Yu Liu[1], Wei Cai[2], Anne-Sophie Chauvin[3], Beat Fierz[2], Jerome Waser*[1]

Table of content

1. General procedures 2
2. HPLC-MS and preparative HPLC information 4
    HPLC-MS analysis 4
    Preparative HPLC 4
    Solid-Phase Peptide Synthesis (SPPS): 4
    MS/MS fragmentation: 5
3. Synthesis of Cz(I)PN derivatives 5
4. Synthesis of CzBN-peptide conjugates via C-terminal decarboxylation 7
  4.1 Condition screenings for decarboxylative arylation of 4CzIPN 7
  4.2 Calibration of the cyclization reaction 8
  4.3 Scope of 4CzIPN-peptide conjugates 9
5. Synthesis of CzIPN-peptide conjugates via S_NAr 72
  5.1 General procedure for the synthesis of 3CzIPN-peptide conjugates 72
  5.2 Condition optimizations for S_NAr of 3CzFIPN 72
  5.3 Scope of 3CzIPN-peptide conjugates 73
6 Photophysical properties of CzPN-peptide conjugates 103
7. Electrochemical measurements of CzPN-peptide conjugates 107
8. Application of CzIPN-peptide conjugates on photo-mediated biomolecule functionalizations 108
  8.1 Decarboxylative alkynylation of peptide C-termini enabled by CzPN-peptide conjugates 108
  8.2 Thiol-ene of peptide enabled by CzPN-peptide conjugates 112

[1]Laboratory of Catalysis and Organic Synthesis (LCSO), École Polytechnique Fédérale de Lausanne, EPFL, 1015 Lausanne, Switzerland
[2] Laboratory of Biophysical Chemistry of Macromolecules (LCBM), Ecole Polytechnique Fédérale de Lausanne, EPFL, 1015 Lausanne, Switzerland
[3] Group of Coordination Chemistry, Institut des Sciences et Ingénierie Chimiques, École Polytechnique Fédérale de Lausanne, EPFL, 1015 Lausanne, Switzerland

8.3 Aryl azide excitation enabled by CzPN-peptide conjugates with blue light 120
8.3.1 Aryl azide excitation on small molecule 120
8.3.2 Fluoroaryl azide excitation on small molecule 122
8.3.3 Stern-volmer quenching between CzPN-peptide conjugates and aryl azide 124
8.4 Synthesis of aryl azide probes 126
8.5 Protein labeling in-vitro via aryl azide excitation 127
8.6 General Western blot procedure: 127
8.7 Live-cell protein labelling via aryl azide excitation 129
8.7.1 Cell line stocks 129
8.7.2 Workflow for aryl azide excitation 130
8.7.3 Labelling of integrin avb3 via aryl azide excitation 131
8.7.4 MTT assay 132
9. X-Ray crystallographic data of **3za** 134
10. NMR spectrum 150

1. General procedures

All reactions using anhydrous conditions were performed with oven-dried glasswares, under an atmosphere of nitrogen, unless stated otherwise. Tetrahydrofuran, acetonitrile, diethyl ether and dichloromethane (DCM) were dried by passage over activated alumina, under nitrogen atmosphere, on an Innovative Technology Solvent Delivery System (water content < 10 ppm, Karl-Fischer titration). Dichloroethane and ethanol were purchased from Acros and trifluoroethanol was purchased from Fluorochem. DMSO was purchased from Sigma-Aldrich. All the Fmoc-protected amino acids (including the non-canonical ones) and Rink Amide MBHA resin were purchased from GL Biochem or Bachem. 1-[Bis(dimethylamino)methylene]-1H-1,2,3-triazolo[4,5-b]pyridinium 3-oxide hexafluorophosphate (HATU, Bachem) and N,N-diisopropylethylamine (DIPEA, Iris Biotech GmbH) were used as received. All the other reagents were purchased from ABCR, Acros, AlfaAesar, Apollo Scientific, Fluorochem, Fluka, Roth, Sigma-Aldrich and TCI and were used as such. For flash chromatography, distilled technical grade solvents were used. Chromatographic purification was performed as flash chromatography using Macherey-Nagel silica 40-63, 60 Å, using the solvents indicated as eluent with 0.1 – 0.5 bar pressure. TLC was performed on Merck silica gel 60 F254 TLC aluminum or glass plates and visualized with UV light or permanganate stain. ^{1}H-NMR spectra were recorded on a Brucker DPX-400 400 MHz, 500 MHz and 600 MHz spectrometer in $CDCl_3$, DMSO-d_6, CD_3OD. All signals are reported in ppm with the internal $CHCl_3$ signal at 7.26 ppm, the internal DMSO signal at 2.50 ppm and CD_3OD as 3.35 ppm as standard. The data is being reported as: s = singlet, d = doublet, t = triplet, q = quadruplet, qi = quintet, m = multiplet or unresolved, br = broad signal, app = apparent, coupling constant(s) in Hz, integration, interpretation.^{13}C-NMR spectra were recorded with 1H-decoupling on a Brucker DPX-400 100 MHz, 126 MHz, 200 MHz spectrometer in $CDCl_3$, DMSO-d_6 or CD_3OD. All signals are reported in ppm with the internal $CHCl_3$ signal at 77.16 ppm or the internal DMSO signal at 39.52 ppm as standard. Spectra were fully assigned using COSY, HSQC, HMBC and ROESY. High-resolution mass spectrometric measurements were performed by the mass spectrometry service of ISIC at the EPFL on LTQ Orbitrap ELITE ETD (Thermo fisher), Xevo G2-S QTOF (Waters), or LTQ Orbitrap ELITE ETD (Thermo fisher). UV/Vis spectroscopy was performed on an Agilent Cary 60 UV-Vis and steady-state luminescence spectroscopy was recorded on a Varian Cary Eclipse spectrophotometer.

2. HPLC-MS and preparative HPLC information

HPLC-MS analysis

HPLC-MS measurements were performed on an Agilent 1290 Infinity HPLC system with a G4226a 1290 Autosampler, a G4220A 1290 Bin Pump and a G4212A 1290 DAD detector, connected to a 6130 Quadrupole LC/MS, coupled with a Waters XBridge C18 column (250 x 4.6 mm, 5 µm). Water:acetonitrile 95:5 (solvent A) and water:acetonitrile 5:95 (solvent B), each containing 0.1% formic acid, were used as the mobile phase, at a flow rate of 0.6 mL.min^{-1}. The gradient was programmed as follows:

Method 1: 100% A to 100% B in 20 minutes then isocratic for 5 minutes.

The column temperature was set up to 25 °C. Low-resolution mass spectrometric measurements were acquired using the following parameters: positive electrospray ionization (ESI), temperature of drying gas = 350 °C, flow rate of drying gas = 12 L. min^{-1}, pressure of nebulizer gas = 60 psi, capillary voltage = 2500 V and fragmentor voltage = 70 V.

Preparative HPLC

Preparative RP-HPLC were performed on an Agilent 1260 HPLC system with a G2260A 1260 Prep ALS Autosampler, a G1361a 1260 Prep Pump, a G1365C 1260 MWD detector and a G1364B 1260 FC-PS collector, coupled with a Waters XBridge semi-preparative C18 column (19 x 150 mm, 5 µm). Water (solvent A) and water:acetonitrile 5:95 (solvent B), each containing 0.1% TFA, were used as the mobile phase at a flow rate of 20 mL.min^{-1}.

Method 2: 100% A to 100% B in 20 minutes then isocratic for 5 minutes.

Method 3: 100% A to 100% B in 25 minutes then isocratic for 5 minutes.

Method 4: 100% A to 90% B in 5 minutes, then 90% B to 100%B in 5 minutes, then isocratic for 5 minutes.

Solid-Phase Peptide Synthesis (SPPS):

Peptides were synthesized on an MultiPep RSi parallel peptide synthesizer (Intavis) using standard Fmoc SPPS-chemistry, 2-chlorotrityl chloride resin (1.38 mmol/g, 100-200 mesh) and Rink-amide resin (0.33 mmol/g). For 2-chlorotrityl chloride resin, the first amino acid was loaded on the resin by incubation of the Fmoc-protected monomer (3 equiv of the number of active sites on the resin), DIPEA (4 equiv) in dichloromethane for 2 h. Each coupling cycle was initiated by Fmoc deprotection achieved by shaking the resin with 800 µL of 20% v/v piperidine in dimethylformamide (DMF) at 400 rpm, over 5 minutes twice. Then the resin was washed with DMF (6000 µL x7). The coupling was carried out by shaking resin with a Fmoc-protected monomer (4.0 equiv.), HATU (4.0 equiv.), *N*-Methylmorpholine (6.0 equiv.), in DMF (1.3 mL), at 400 rpm, over 30 minutes twice. Capping using Cap Mixture (5% v/v Ac_2O and 6% v/v 2,6-lutidine in DMF) was carried out at the end of each cycle, followed by a DMF wash (6000 µL x7). The synthesis was finished by deprotection of Fmoc using 20% v/v piperidine in dimethylformamide at 400 rpm, over 5 minutes two times. The N-terminus was either left unprotected or was acylated. Acetylation of the N-terminal was achieved by incubating the resin with Cap Mixture three times. Next, washing steps were performed with dimethylformamide (5 x 3 mL). Finally, resin was dried with dichloromethane (5 x 3 mL).

Peptide cleavage and deprotection:

Peptides without protecting groups

Peptides were deprotected and cleaved from the resin by treatment with 2.5% v/v water and 2.5% v/v Triisopropyl silane in neat trifluoroacetic acid (2 mL) (Note: For polyArg sequence, reagent R (TFA:thioanisole:EDT:anisole 90:5:3:2) was used). The resulting mixture was shaken for 2 hours, at room temperature. The resin was removed by filtration and peptides were precipitated in cold diethyl ether (50 mL), followed by a 2 hours incubation at -20 °C. Peptides were pelleted by centrifugation at 4000 rpm, for 5 minutes. Finally, the mother liquors were carefully removed.

The precipitations were further dissolved in water and acetonitrile, shell freeze and lyophilize to yield the desired crude peptides. If necessary, preparative HPLC purification was carried out.

Peptide analysis:

MS/MS fragmentation:

The regioselectivity of functionalization was confirmed using MS/MS analysis. The spectra were obtained by the mass spectrometry service of ISIC at the EPFL using Thermo Orbitrap Elite instrument. The desired ion was selected using mass filters and submitted to fragmentations. The obtained data was analyzed using fragment generation program on eln.epfl.ch.[2] For the calculations peak threshold for intensity was set to 0.5% and 0.03% for quantity, precision was set to 5 ppm and minimal similarity: 70%. The peaks were compared to theoretical peaks. The theoretical peak width was calculated from the mass of the ion by the formula provided in the script. The zone was set to -0.5 to 3.5 ppm. y and b fragments with and without linker were selected and reported. In the cases were fragmentation was low, c and z fragments and/or fragments arising from neutral losses were included.

3. Synthesis of Cz(I)PN derivatives

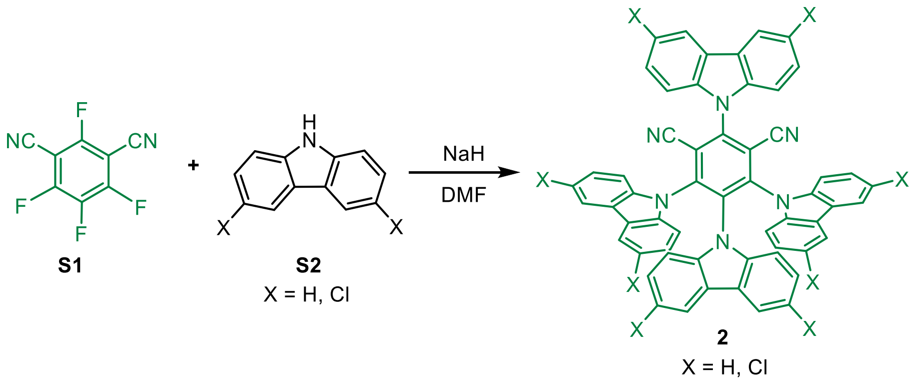

General procedure for the synthesis of **2a** and **2b**: Sodium hydride (60% suspension in mineral oil, 6.0 equiv) was added slowly to a stirred solution of substituted-carbazole **S2a,b** (4.4 equiv) in dry THF (0.05 M) under a nitrogen atmosphere at RT. After 30 min, 2,4,5,6-

[2] a) Desport, J.S., Frache, G., and Patiny, L. (2020), MSPolyCalc: A web-based App for polymer mass spectrometry data interpretation. The case study of a pharmaceutical excipient. Rapid Commun. Mass Spectrom. *34*, e8652.; b) Ortiz, D., Gasilova, N., Sepulveda, F., Patiny, L., Dyson, P.J., and Menin, L. (2020), Aom2S: A new web-based application for DNA/RNA tandem mass spectrometry data interpretation. Rapid Commun. Mass Spectrom. *34*, e8927.

tetrafluoroisophthalonitrile **S1** (1.0 equiv) was added. After stirring at RT for 15 h, 2 mL water was added to the reaction mixture to quench the excess of NaH. The resulting mixture was then concentrated under reduced pressure. The crude product was purified by recrystallization from hexane/CH_2Cl_2 then filtered.

4CzIPN (2a)

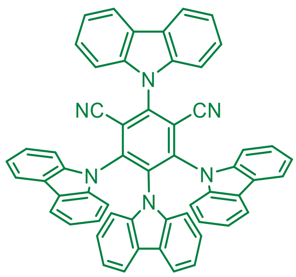

Following the **General procedure**, the reaction was conducted on a 2.5 mmol scale. The desired product **4CzIPN (2a)** was obtained as yellow solid (980 mg, 1.24 mmol, 50%).
^{1}H NMR (400 MHz, $CDCl_3$) δ 8.22 (d, J = 7.7 Hz, 2H), 7.70 (dt, J = 10.4, 4.8 Hz, 8H), 7.52 – 7.45 (m, 2H), 7.35 – 7.30 (m, 2H), 7.24 – 7.19 (m, 4H), 7.13 – 7.00 (m, 9H), 6.86 – 6.77 (m, 4H), 6.63 (t, J = 7.7 Hz, 2H).
The **^{1}H NMR** shift are consistent with reported data.[3]

8Cl-4CzIPN (2b)

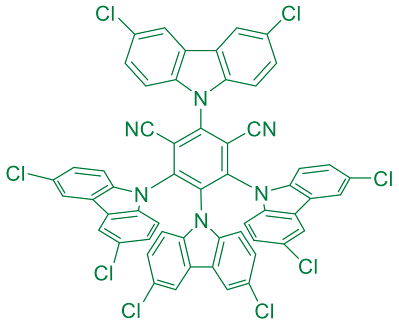

Following the **General procedure**, the reaction was conducted on a 1.0 mmol scale. The desired product **8Cl-4CzIPN (2b)** was obtained as yellow solid (689 mg, 0.650 mmol, 65%).
^{1}H NMR (400 MHz, $CDCl_3$) δ 8.16 (d, J = 1.9 Hz, 2H), 7.73 (d, J = 2.0 Hz, 4H), 7.68 (dd, J = 8.7, 2.1 Hz, 3H), 7.48 (d, J = 8.6 Hz, 2H), 7.42 (d, J = 2.0 Hz, 2H), 7.12 (dd, J = 8.7, 2.0 Hz, 4H), 7.02 (d, J = 8.8 Hz, 4H), 6.73 (dd, J = 8.7, 2.1 Hz, 2H), 6.66 (d, J = 8.7 Hz, 2H).
The **^{1}H NMR** shift are consistent with reported data.[4]

2CzPN (2d)

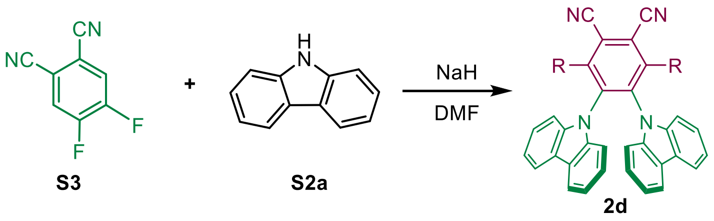

[3] Uoyama, H.; Goushi, K.; Shizu, K.; Nomura, H.; Adachi, C., *Nature* **2012,** *492* (7428), 234-238.
[4] Kretzschmar, A.; Patze, C.; Schwaebel, S. T.; Bunz, U. H. F., *J. Org. Chem.* **2015,** *80* (18), 9126-9131.

Sodium hydride (60% suspension in mineral oil, 3.0 equiv) was added slowly to a stirred solution of substituted-carbazole **S2a** (2.2 equiv) in dry THF (0.05 M) under a nitrogen atmosphere at RT. After 30 min, **S3** (1.0 equiv) was added. After stirring at RT for 15 h, 2 mL water was added to the reaction mixture to quench the excess of NaH. The resulting mixture was then concentrated under reduced pressure. The crude product **2d** was purified by recrystallization from hexane/CH_2Cl_2 then filtered as green solid (784 mg, 1.71 mmol, 57% yield).

^{1}H NMR (400 MHz, $CDCl_3$) δ 8.32 (s, 2H), 7.80 (d, J = 7.7, 1.1 Hz, 4H), 7.15 – 7.09 (m, 5H), 7.09 – 7.04 (m, 7H).

The **^{1}H NMR** shift are consistent with reported data [5]

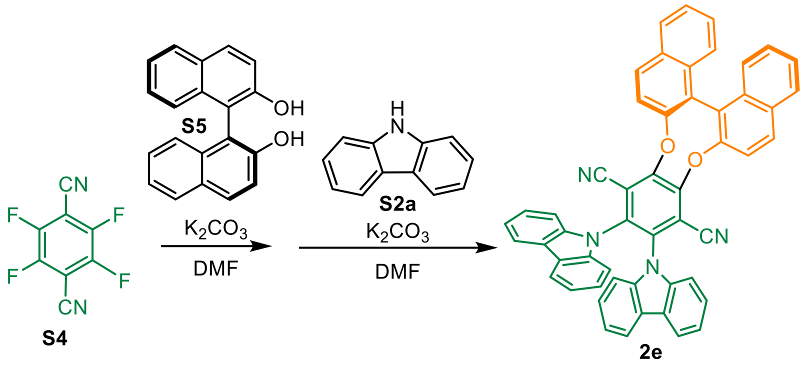

Following a reported procedure[6], to a 10 mL flask, dry DMF (2.5 mL) was added to dissolve **S4** (100 mg, 0.500 mmol, 1.00 equiv.). K_2CO_3 (138 mg, 1.00 mmol, 2.00 equiv.) and (S)-BINOL **S5** (143 mg, 0.500 mmol, 1.00 equiv.) were added. The reaction mixture was stirred for 12 h at room temperature under nitrogen. After completion of the reaction monitored by thin layer chromatography, K_2CO_3 (346 mg, 2.50 mmol, 5.00 equiv.) and carbazole **S2a** (184 mg, 1.10 mmol, 2.20 equiv.) were added to the medium. The reaction mixture was stirred for 3 hours at room temperature under nitrogen. Water (2 mL) and dichloromethane (5 mL) were then added and the organic layer was washed with dichloromethane (2 x 5 mL), dried over Na_2SO_4 and filtered. The solvent was then removed under vacuum and the solid was purified by flash chromatography to give **2e** as a yellow solid (251 mg, 0.339 mmol, 68% yield).

^{1}H NMR (400 MHz, $CDCl_3$) δ 8.05 (d, J = 8.9 Hz, 2H), 7.94 (d, J = 8.4 Hz, 2H), 7.64 (d, J = 8.8 Hz, 2H), 7.55 – 7.47 (m, 4H), 7.50 – 7.41 (m, 3H), 7.37 (d, J = 7.0 Hz, 2H), 7.11 (dd, J = 7.2, 1.3 Hz, 2H), 7.08 – 7.00 (m, 4H), 6.83 (t, J = 7.4 Hz, 2H), 6.64 (t, J = 8.4 Hz, 2H), 6.57 (d, J = 8.3 Hz, 2H).

The **^{1}H NMR** shift are consistent with reported data.[4]

4. Synthesis of CzBN-peptide conjugates via C-terminal decarboxylation

4.1 Condition screenings for decarboxylative arylation of 4CzIPN

General procedure:

[5] Rolka, A. B.; Koenig, B., *Org. Lett.* **2020,** *22* (13), 5035-5040.

[6] Feuillastre, S.; Pauton, M.; Gao, L.; Desmarchelier, A.; Riives, A. J.; Prim, D.; Tondelier, D.; Geffroy, B.; Muller, G.; Clavier, G.; Pieters, G., *J. Am. Chem. Soc.* **2016,** *138* (12), 3990-3993.

Peptide **1a** (2 μmol, 2 equiv.) and 4CzIPN **2a** (1 μmol, 1 equiv.) were weighed on the analytical balance and dissolved in 0.95 mL non-degassed DMSO in a 5 mL vial. Afterwards, 5 μL of 2 M K_2CO_3 (10 equiv.) in milli-Q purified water were placed into the vial, overall concentration: 10 mM. The vial was then capped and degassed by bubbling with N_2 for 20 min. The reaction was stirred under Blue LED strips (4 hours)/Kessil lamp (440 nm) (1 hour) irradiation at RT. The CzPN-peptides were isolated by Prep-RP-HPLC, followed by lyophilization.

For reaction used Kessil lamp: The reaction vials were placed on a stirring plate with Kessil lamps (390/440 nm, 40 W, 25% intensity) (the hood was free and coated with aluminum foil for personal protection). The distance between the Kessil lamps and the vials was approximatively 10 cm. A fan was used to cool down the reaction system.

Table S1 Condition optimizations for decarboxylative photosubstitution of 4CzIPN

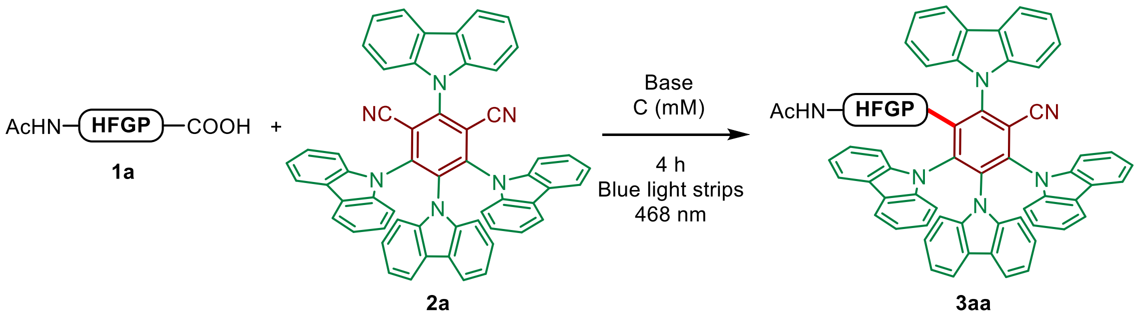

Entry	Base[a]	C(mM)	1a:2a	Solvent	Yield%
1[b]	K_2HPO_4	10	2	DMA	59
2	K_2HPO_4	10	2	DMF	75
3	K_2HPO_4	10	2	DMSO	79
4	Cs_2CO_3	10	2	DMSO	64
5[b]	Na_2HPO_4	10	2	DMSO	49
**6**	**K_2CO_3**	**10**	**2**	**DMSO**	**85(69%)[c]**
7	K_2CO_3	5	2	DMSO	80
8	K_2CO_3	15	2	DMSO	29
9	K_2CO_3	10	1.5	DMSO	66
10	K_2CO_3	10	1.2	DMSO	55
11	K_2CO_3	6.67	0.5	DMSO	76

The reactions were performed on a 1 μmol scale, the yield was determined based on the HPLC-UV calibration curve of **3aa**. [a]2 M of base solution in H_2O (10 equiv.). [b]The reaction was running for 16 h. [c]Isolated yield of **3aa** on 0.01 mmol scale. Isolated yield of **3aa** was presented on the parentheses.

4.2 Calibration of the cyclization reaction

Absorbance (mAU) versus concentration (mM) of **3aa**

Conc. (mM)	Absorbance (mAU)
0.1	1105.245
0.2	2220.271

0.3	3295.737
0.4	4562.553

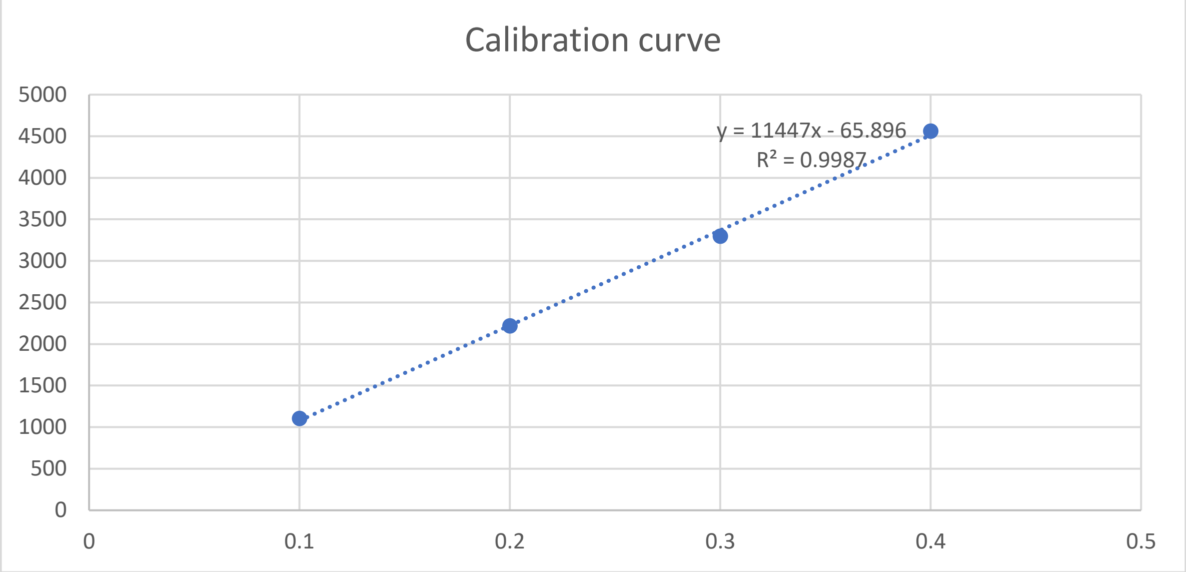

Linear equation of the absorbance (mAU) versus concentration (mM) of 3aa

4.3 Scope of 4CzBN-peptide conjugates

Cbz-Ala 4CzBN 3za

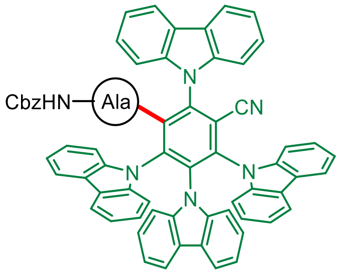

Following the general procedure, by switching the solvent from DMSO to DMA, the reaction was conducted on aa 0.02 mmol scale. The desired product **3za** (12.3 mg, 0.131 mmol, 65% yield) was isolated by **Method 4.**

^{1}H NMR (500 MHz, $CDCl_3$) δ 8.26 – 8.19 (m, 2H, ArH), 7.74 – 7.64 (m, 4H, ArH), 7.63 – 7.55 (m, 4H, ArH), 7.46 (t, *J* = 7.4 Hz, 1H), 7.44 – 7.38 (m, 2H), 7.35 – 7.26 (m, 5H, ArH(Bn)), 7.24 – 7.15 (m, 3H, ArH), 7.15 – 7.03 (m, 4H, ArH), 7.02 – 6.77 (m, 8H, ArH), 6.76 – 6.68 (m, 2H, ArH), 6.63 (t, *J* = 7.7 Hz, 1H, ArH), 6.57 (t, *J* = 7.9 Hz, 1H, ArH), 5.15 (p, *J* = 7.2 Hz, 1H, NCH), 4.78 (d, *J* = 11.9 Hz, 1H, NH), 4.44 (d, *J* = 11.9 Hz, 1H, OCH_2), 4.09 (d, *J* = 7.3 Hz, 1H, OCH_2), 1.17 (d, *J* = 7.2 Hz, 3H, CH_3).

^{13}C NMR (126 MHz, $CDCl_3$) δ 154.9, 148.6, 142.6, 142.0, 141.8, 141.1, 140.9, 139.8, 139.6, 139.1, 138.8, 138.4, 138.1, 136.0, 128.4, 128.3, 128.2, 127.0, 125.8, 125.5, 125.3, 125.0, 124.6, 124.4, 124.3, 124.1, 123.9, 123.8, 123.5, 123.5, 121.6, 121.4, 121.3, 121.3, 121.1, 121.1, 120.7, 120.4, 120.2, 120.2, 120.1, 120.0, 119.5, 119.3, 119.3, 66.7, 47.9, 21.6.

HRMS (nanochip-ESI/LTQ-Orbitrap) m/z: $[M + Na]^+$ Calcd for $C_{65}H_{44}N_6NaO_2^+$ 963.3418; Found 963.3400.

AcHFGP-OH (1a)

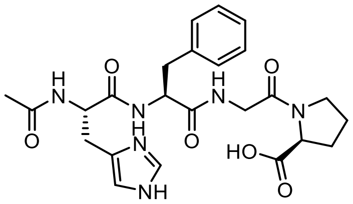

HPLC-UV chromatogram (210 nm) of AcHFGP (**1a**) by **Method 1**:

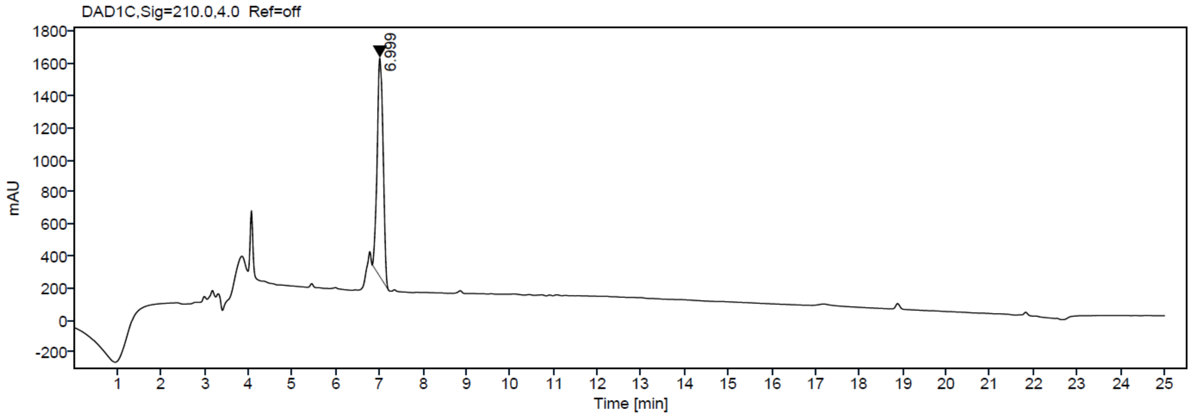

Retention time: 7.035 min **Area Percent:** 100%

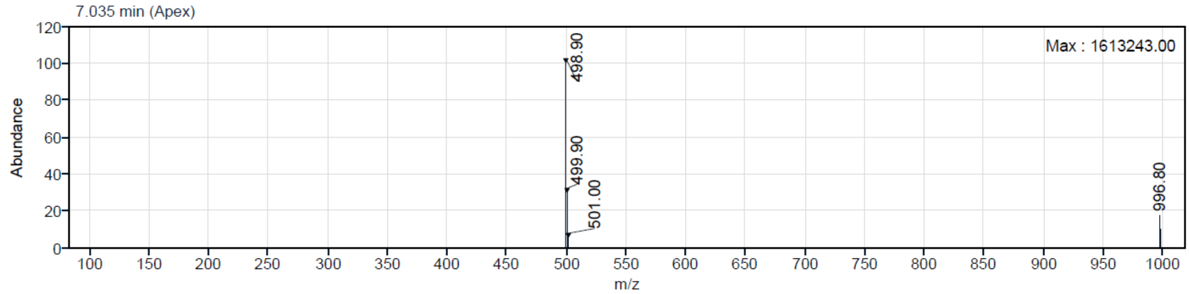

HRMS (nanochip-ESI/LTQ-Orbitrap) m/z: $[M + H]^+$ Calcd for $C_{24}H_{31}N_6O_6^+$ 499.2300; Found 499.2291.

AcHFGP 4CzBN (3aa)

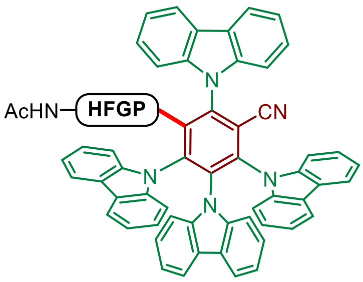

Following the general procedure, the reaction was conducted on aa 0.017 mmol scale. The desired product **3aa** (14.1 mg, 0.116 mmol, 69% yield) was isolated by **Method 2.**
HPLC-UV chromatogram (210 nm) of crude by **Method 1**:

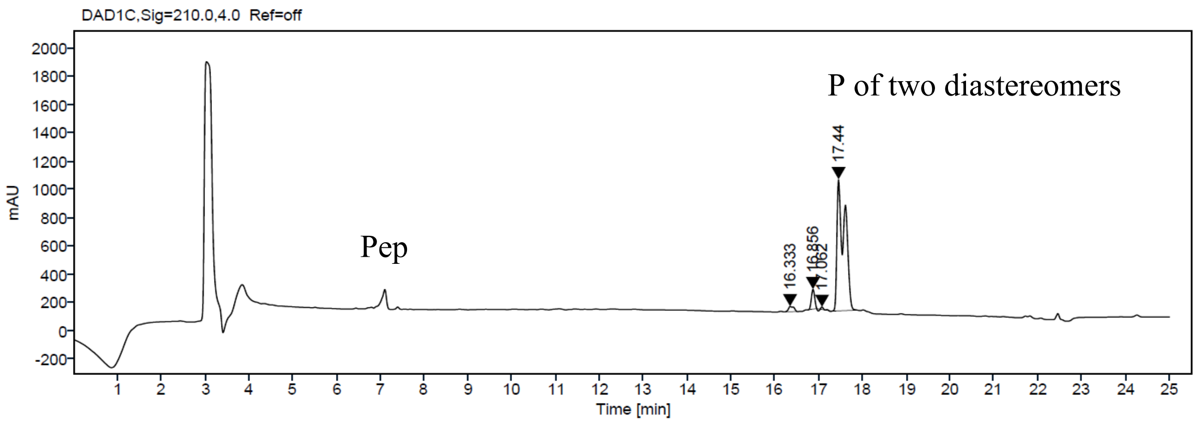

HPLC-UV chromatogram (210 nm) of product **3aa** by **Method 1**:

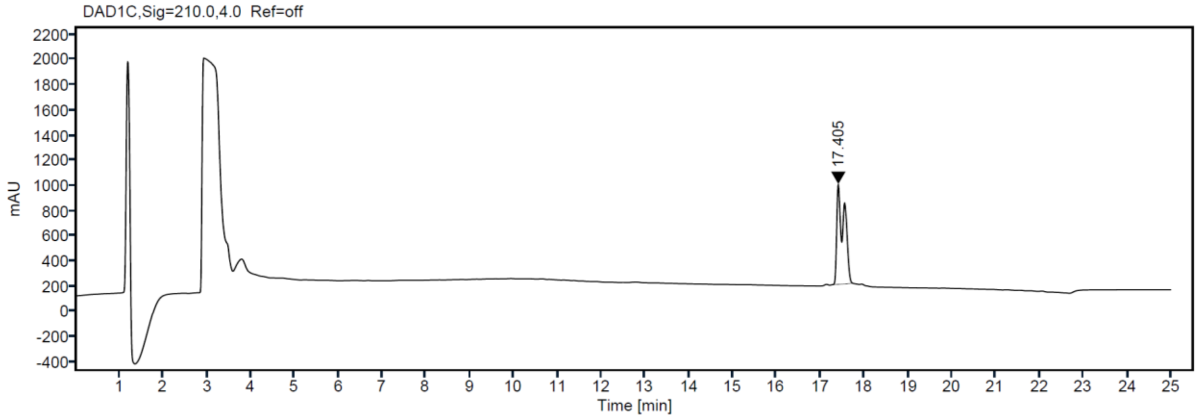

Retention time: 17.407 min **Area Percent:** 100%

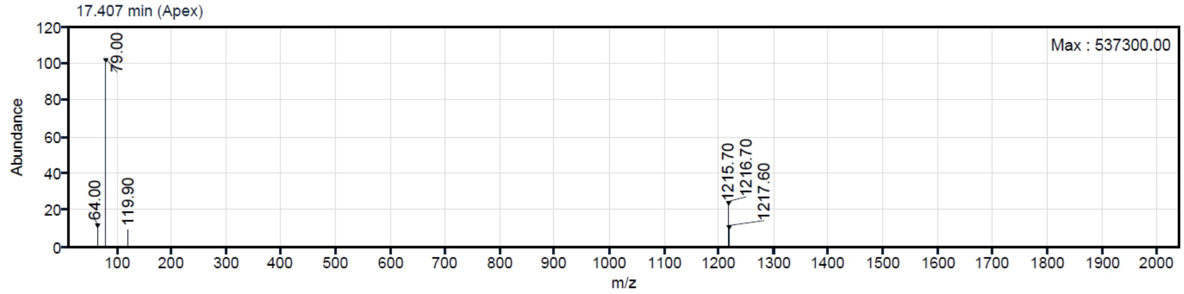

HRMS (nanochip-ESI/LTQ-Orbitrap) m/z: $[M + H]^+$ Calcd for $C_{78}H_{62}N_{11}O_4^+$ 1216.4981; Found 1216.4965.

^{1}H NMR (500 MHz, DMSO, **90 ºC**) δ 8.29 (d, *J* = 7.7 Hz, 2H), 8.10 (dd, *J* = 8.2, 2.9 Hz, 1H), 7.81 (q, *J* = 9.9, 6.9 Hz, 2H), 7.76 (d, *J* = 7.4 Hz, 2H), 7.72 (d, *J* = 6.6 Hz, 2H), 7.68 (d, *J* = 7.7 Hz, 2H), 7.64 (td, *J* = 11.7, 10.7, 6.4 Hz, 3H), 7.54 (d, *J* = 8.1 Hz, 2H), 7.51 (d, *J* = 2.9 Hz, 1H), 7.43 (d, *J* = 7.8 Hz, 2H), 7.42 – 7.35 (m, 3H), 7.31 (dq, *J* = 6.5, 3.7 Hz, 3H), 7.28 (d, *J* = 3.7 Hz, 2H), 7.23 (d, *J* = 9.0 Hz, 1H), 7.20 (s, 2H), 7.11 (q, *J* = 7.5 Hz, 2H), 6.97 – 6.89 (m, 2H), 6.85 (d, *J* = 6.3 Hz, 2H), 6.78 (d, *J* = 6.4 Hz, 1H), 6.73 – 6.63 (m, 4H), 4.86 (q, *J* = 9.3 Hz, 1H), 4.61 (dq, *J* = 27.6, 7.4 Hz, 1H), 4.50 (dt, *J* = 13.0, 6.5 Hz, 1H), 3.45 – 3.31 (m, 1H), 2.85 – 2.72 (m, 3H), 2.39 – 2.31 (m, 1H), 1.92 (dq, *J* = 8.3, 5.2, 3.2 Hz, 1H), 1.88 – 1.78 (m, 3H), 1.28 (d, *J* = 9.2 Hz, 1H), 1.23 – 1.09 (m, 1H), 0.86 (dtd, *J* = 12.7, 8.8, 7.8, 4.4 Hz, 1H).

^{13}C NMR (126 MHz, DMSO, **60 ºC**) (Only one diastereomer was resolved) δ 170.9, 170.4, 168.9, 166.9, 162.5, 156.0, 149.5, 141.2, 140.1, 139.1, 138.9, 138.4, 138.3, 137.5, 134.3, 129.0, 128.9, 127.8, 126.8, 126.4, 126.0, 125.4, 124.7, 124.1, 123.6, 123.3, 123.2, 123.0, 122.9, 122.6, 122.3, 122.2, 122.1, 120.7, 120.4, 120.4, 120.3, 120.1, 120.0, 119.9, 119.7, 119.5, 119.0, 118.8,

118.6, 112.9, 112.0, 111.7, 110.7, 110.4, 56.9, 53.5, 52.7, 45.0, 41.6, 37.6, 37.4, 30.6, 23.5, 22.3.

MS/MS fragmentation of **3aa**

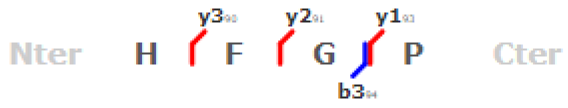

Nter = C2H3O
Cter = C54H32O-1N5

Sequence	Type	MF	MF Mass	m/z	Intensity	Similarity
HFG	b3	C19H22N5O4(+1)	384.1672	384.1666	0.87	93.79%
P	y1	C59H41N6(+1)	833.3393	833.3387	100.59	92.60%
GP	y2	C61H44N7O(+1)	890.3607	890.3602	48.72	91.15%
FGP	y3	C70H53N8O2(+1)	1037.429	1037.429	21.04	90.18%

AcRFGP 1b

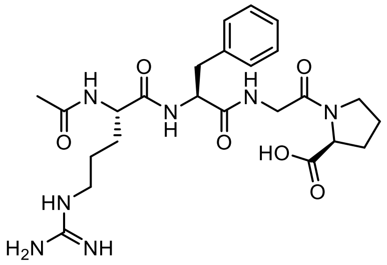

HPLC-UV chromatogram (210 nm) of Ac-RFGP **1b** by **Method 1**:

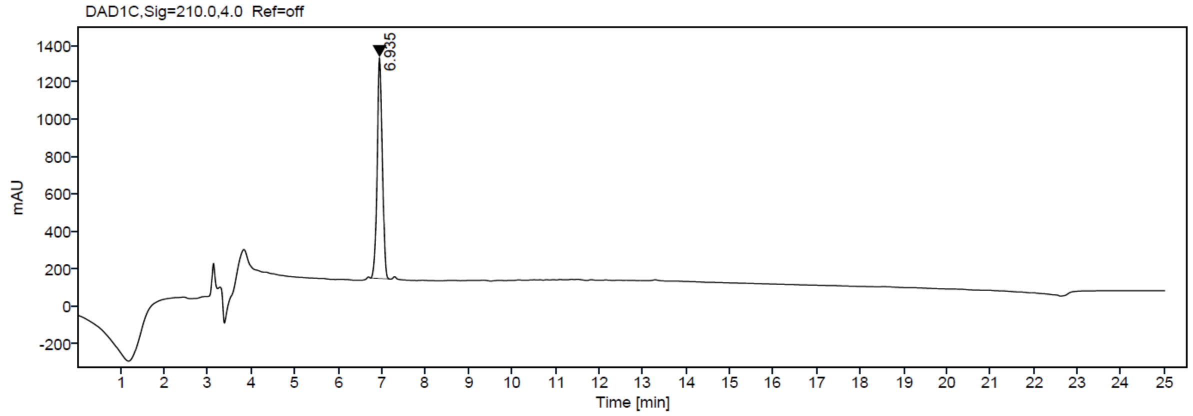

Retention time: 6.942 min **Area Percent:** 100%

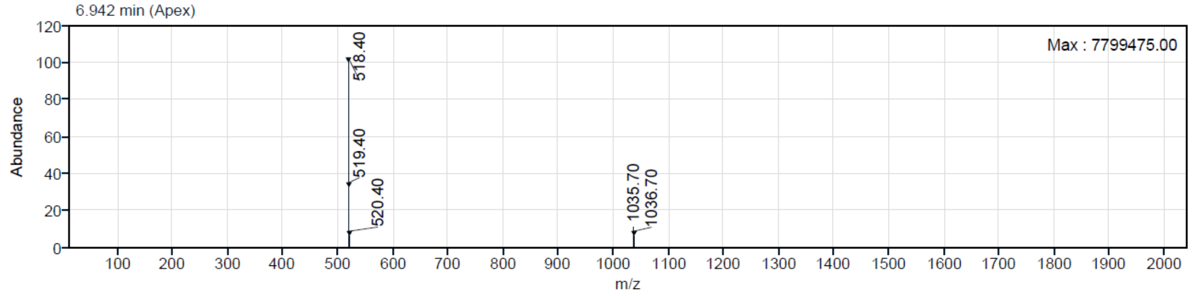

HRMS (nanochip-ESI/LTQ-Orbitrap) m/z: $[M + H]^+$ Calcd for $C_{24}H_{36}N_7O_6^+$ 518.2722; Found 518.2715.

AcRFGP 4CzBN (3ba)

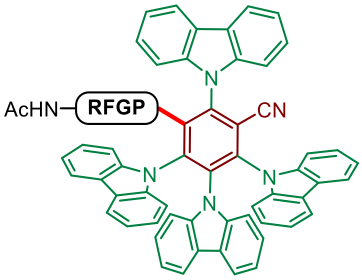

Following the general procedure, the reaction was conducted on a10 µmol scale. The desired product **3ba** (3.0 mg, 2.4 µmol, 24% yield) was isolated by **Method 3.**

HPLC-UV chromatogram (210 nm) of the crude by **Method 1**:

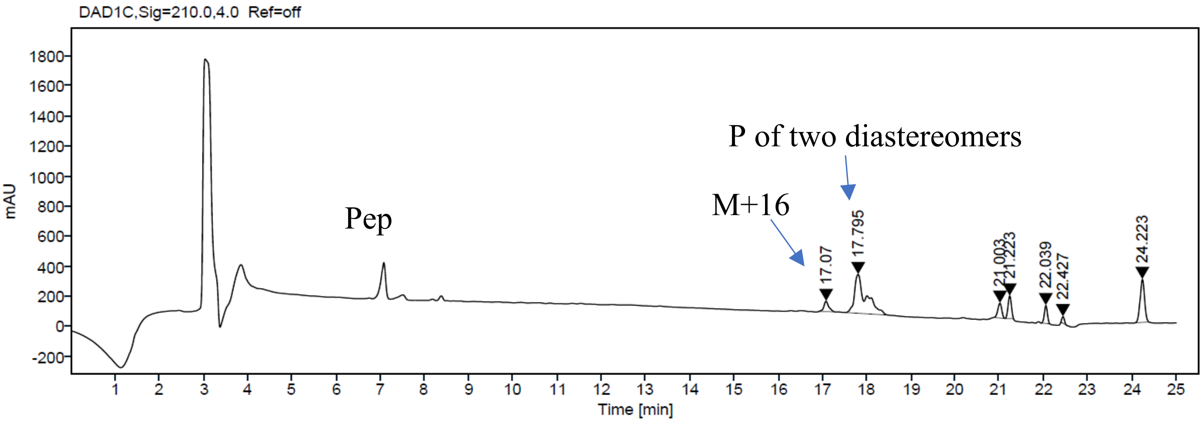

HPLC-UV chromatogram (210 nm) of **3ba** by **Method 1**:

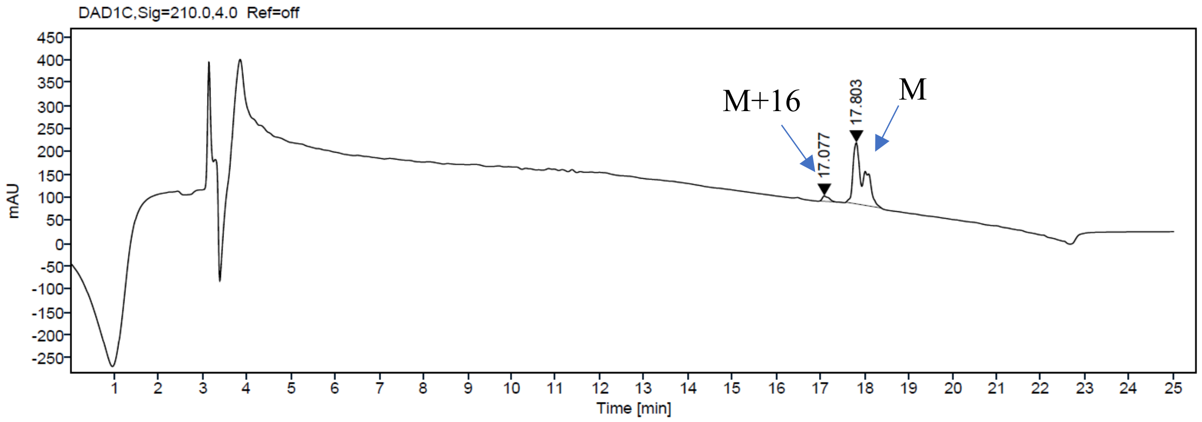

Retention time: 17.842 min **Area Percent:** 100%

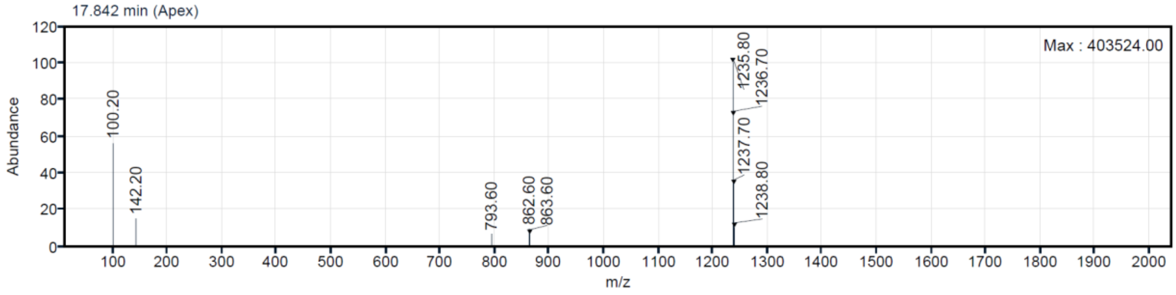

HRMS (nanochip-ESI/LTQ-Orbitrap) m/z: $[M + H]^+$ Calcd for $C_{78}H_{67}N_{12}O_4^+$ 1235.5403; Found 1235.5422.

MS/MS fragmentation of **3ba**

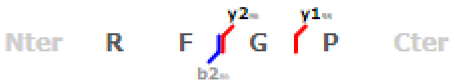

Nter = C2H3O
Cter = C54H32N5O-1

Sequence	Type	MF	MF Mass	m/z	Intensity	Similarity
P	y1	C59H41N6(+1)	833.3393	833.3387	96.59	98.04%
GP	y2	C61H44N7O(+1)	890.3607	890.3602	101.59	96.22%
RF	b2	C17H24N5O3(+1)	346.1879	346.1874	0.85	85.72%

AcMFGP 1c

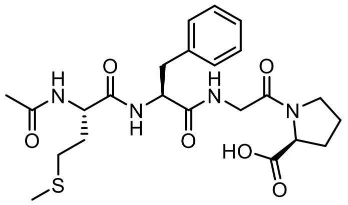

HPLC-UV chromatogram (210 nm) of AcMFGP **1c** by **Method 1**:

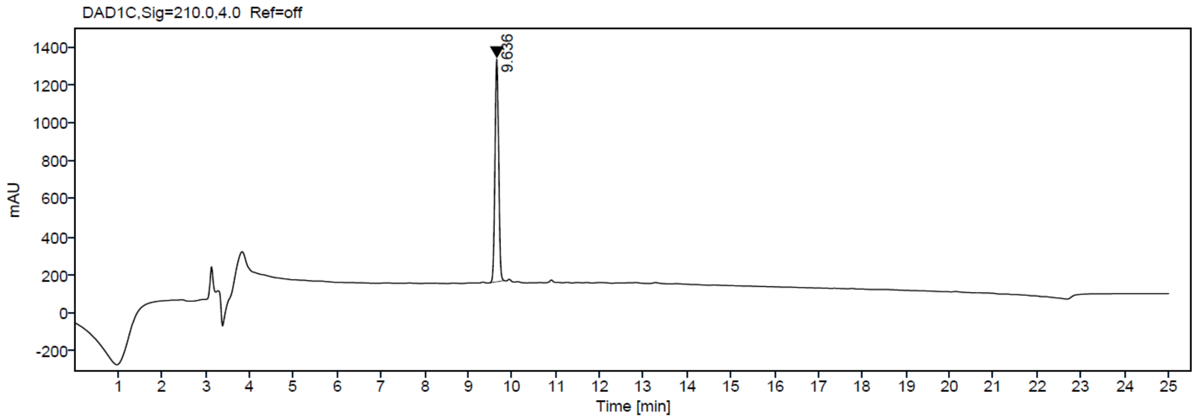

Retention time: 9.667 min **Area Percent:** 100%

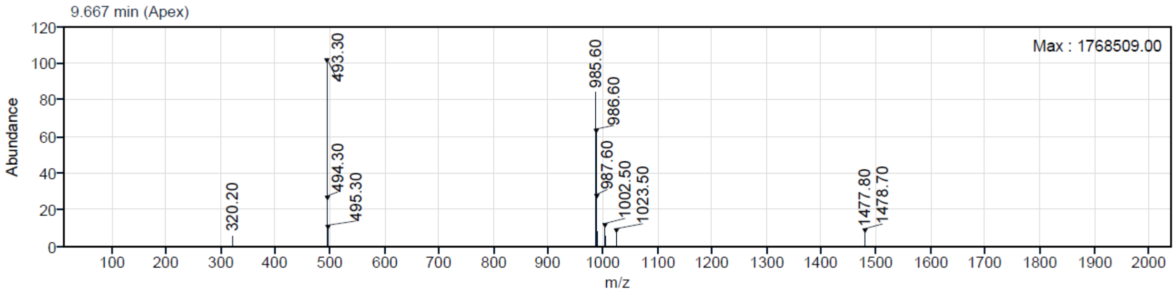

HRMS (nanochip-ESI/LTQ-Orbitrap) m/z: $[M + H]^+$ Calcd for $C_{23}H_{33}N_4O_6S^+$ 493.2115; Found 493.2106.

AcMFGP 3ca

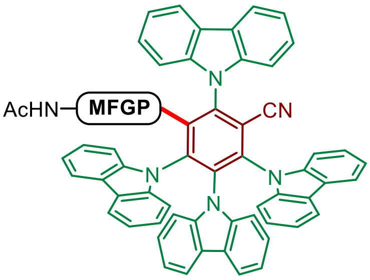

Following the general procedure, the reaction was conducted on a10 μmol scale. The desired product **3ca** (8.0 mg, 6.6 μmol, 66% yield) was isolated by **Method 4.**

HPLC-UV chromatogram (210 nm) of the crude by **Method 1**:

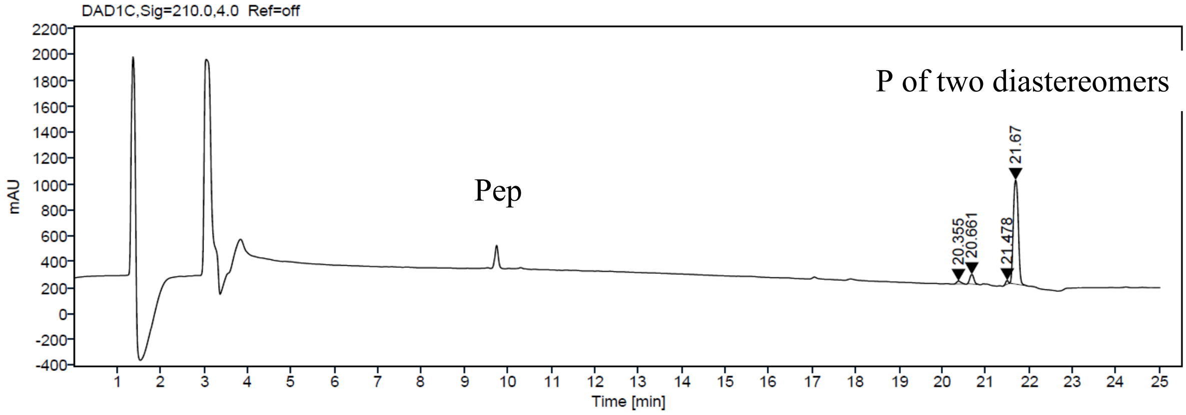

HPLC-UV chromatogram (210 nm) of **3ca** by **Method 1**:

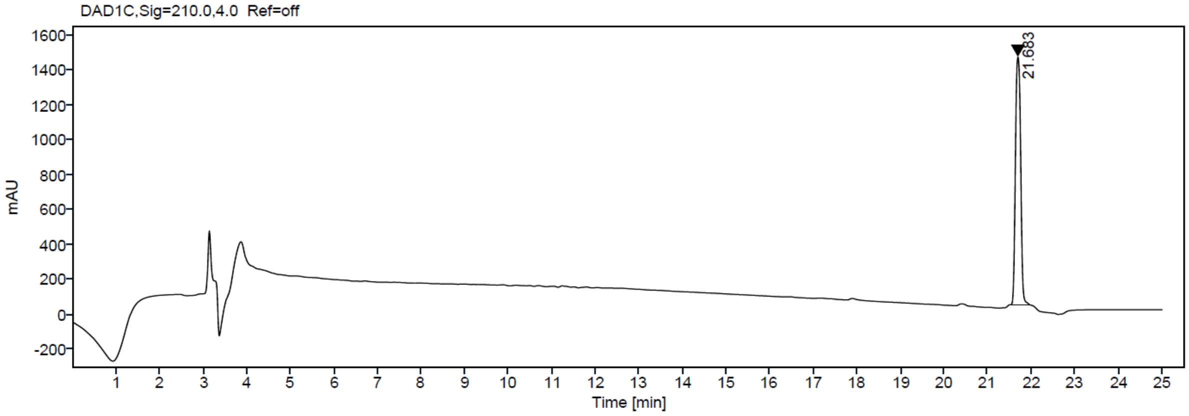

Retention time: 21.555 min **Area Percent:** 100%

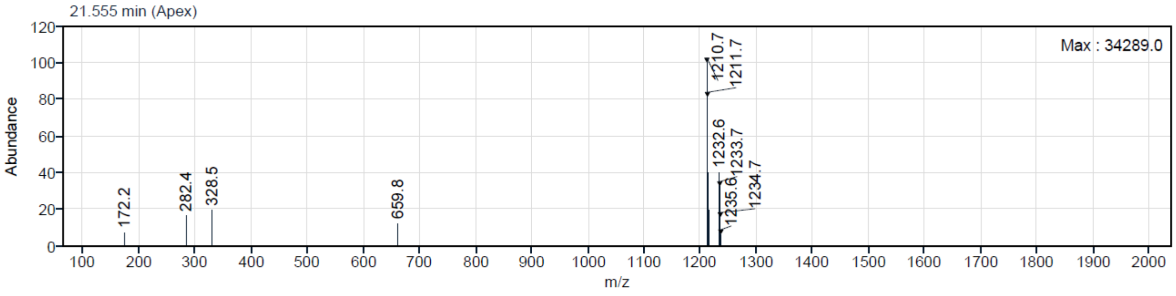

HRMS (ESI/QTOF) m/z: $[M + Na]^+$ Calcd for $C_{77}H_{63}N_9NaO_4S^+$ 1232.4616; Found 1232.4662.
MS/MS fragmentation of **3ca**

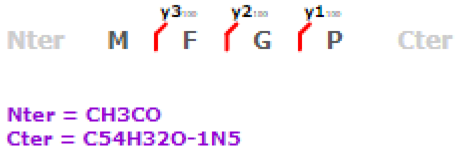

Sequence	Type	MF	MF Mass	m/z	Intensity	Similarity
P	y1	C59H41N6(+1)	833.3393	833.3387	31.61	99.97%
FGP	y3	C70H53N8O2(+1)	1037.429	1037.429	69.35	99.95%
GP	y2	C61H44N7O(+1)	890.3607	890.3602	102.07	99.94%

DFGP (1d)

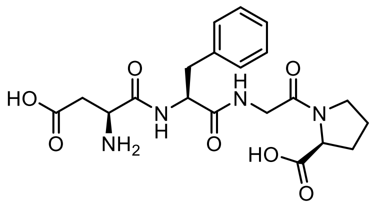

HPLC-UV chromatogram (210 nm) of DFGP (**1d**) by **Method 1**:

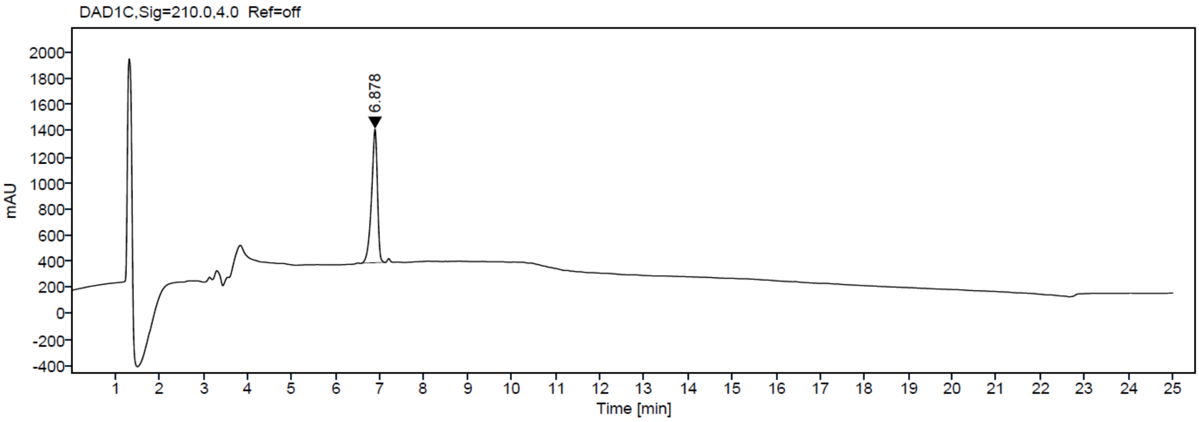

Retention time: 6.904 min **Area Percent:** 100%

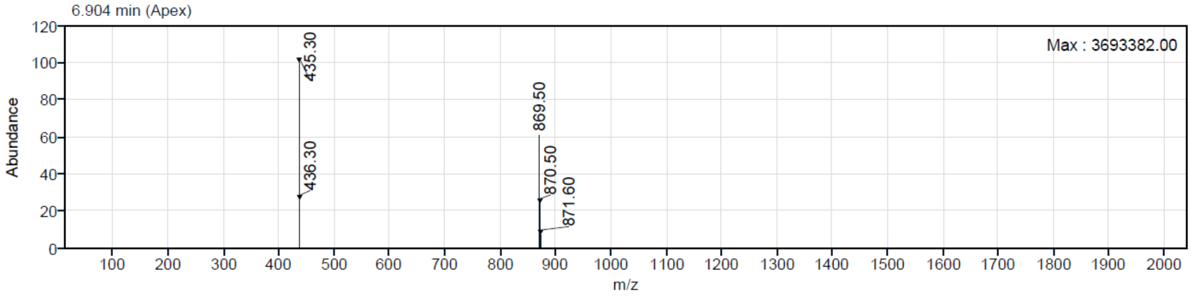

HRMS (nanochip-ESI/LTQ-Orbitrap) m/z: $[M + H]^+$ Calcd for $C_{20}H_{27}N_4O_7^+$ 435.1874; Found 435.1857.

DFGP 4CzBN (3da)

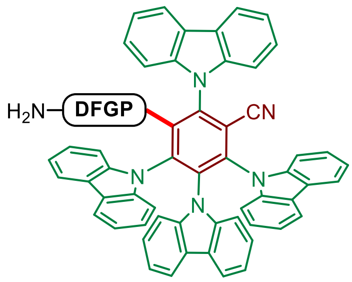

Following the general procedure, the reaction was conducted on a10 μmol scale. The desired product **3da** (8.0 mg, 6.9 μmol, 69% yield) was isolated by **Method 2**

HPLC-UV chromatogram (210 nm) of crude by **Method 1**:

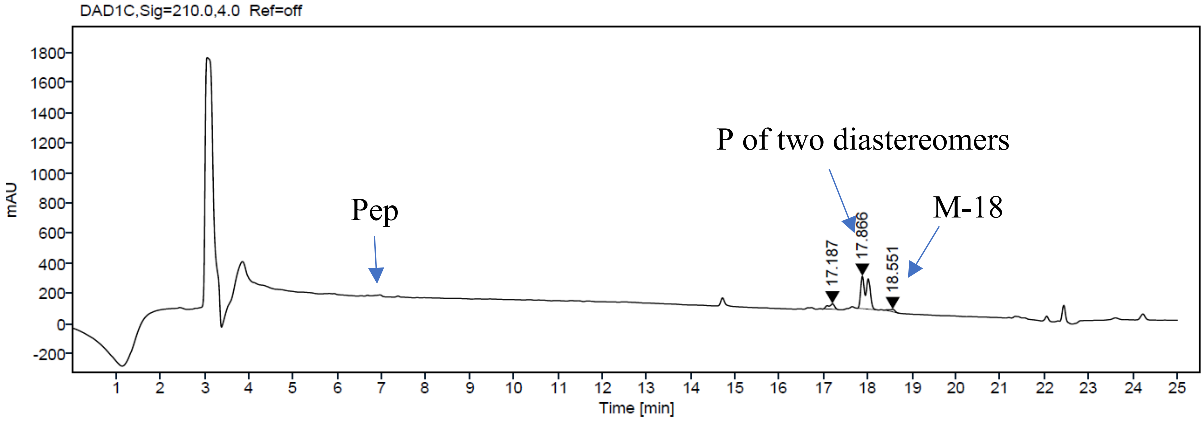

HPLC-UV chromatogram (210 nm) of product **3da** by **Method 1**:

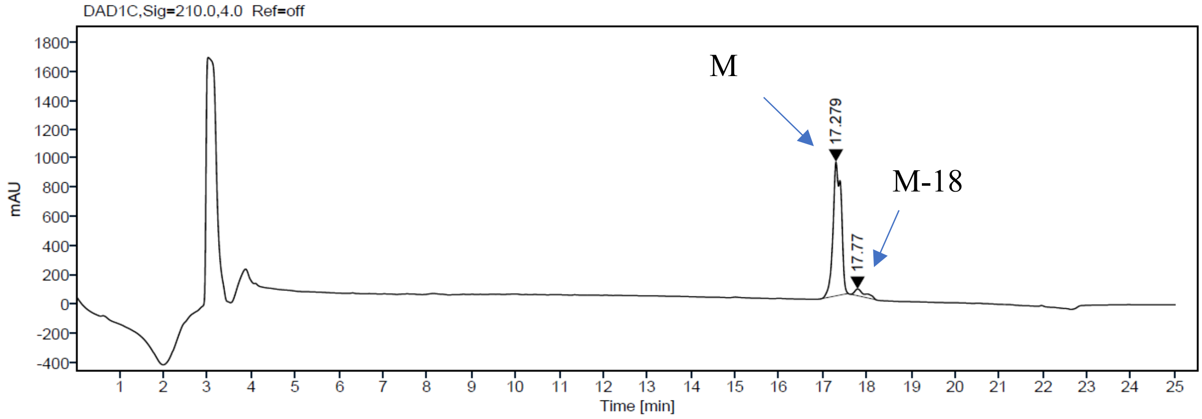

Retention time: 17.312 min **Area Percent:** 100%

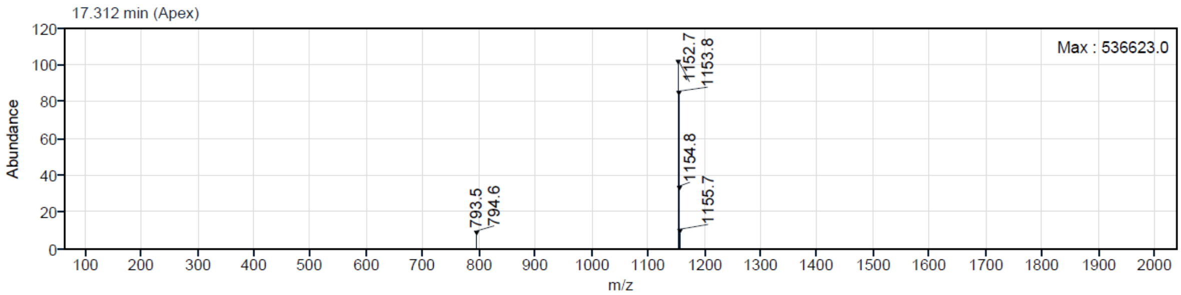

HRMS (nanochip-ESI/LTQ-Orbitrap) m/z: $[M + H]^+$ Calcd for $C_{74}H_{58}N_9O_5^+$ 1152.4555; Found 1152.4541

MS/MS fragmentation of **3da**

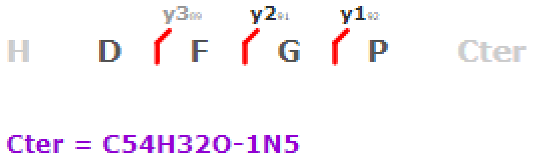

Sequence	Type	MF	MF Mass	m/z	Intensity	Similarity
P	y1	C59H41N6(+1)	833.3393	833.3387	18.34	92.34%
GP	y2	C61H44N7O(+1)	890.3607	890.3602	102.17	90.62%
FGP	y3	C70H53N8O2(+1)	1037.429	1037.429	1.53	88.52%

NFGP (1e)

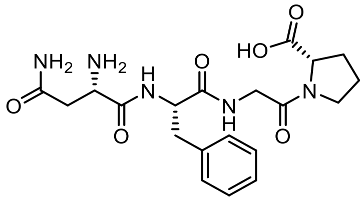

HPLC-UV chromatogram (210 nm) of NFGP (**1e**) by **Method 1**:

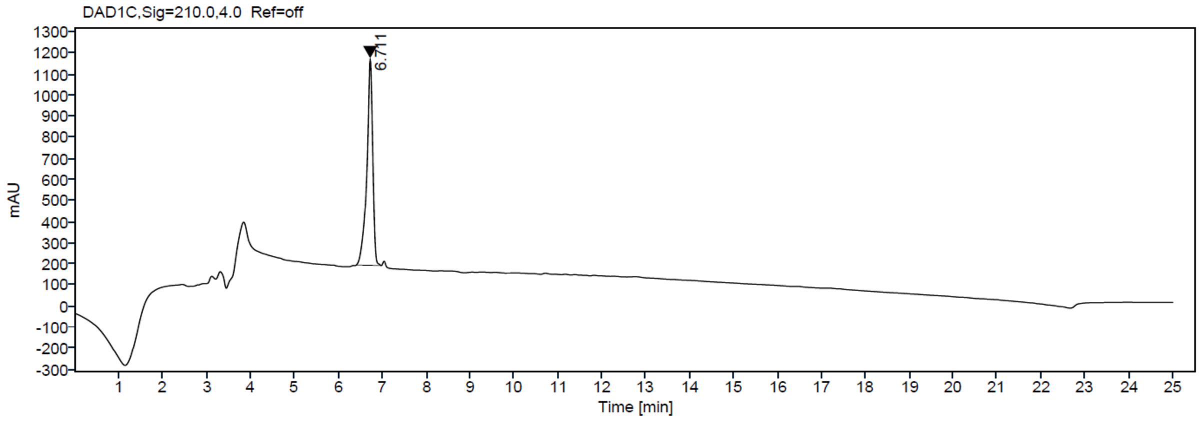

Retention time: 6.725 min **Area Percent:** 100%

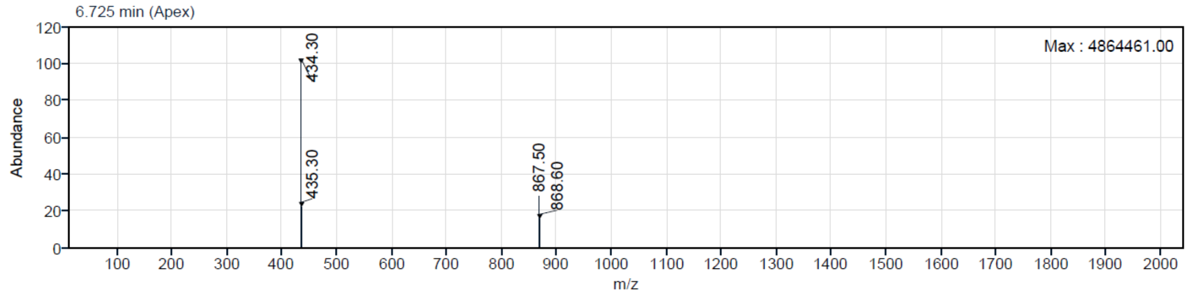

HRMS (nanochip-ESI/LTQ-Orbitrap) m/z: $[M + H]^+$ Calcd for $C_{20}H_{28}N_5O_6^+$ 434.2034; Found 434.2028.

NFGP 4CzBN 3ea

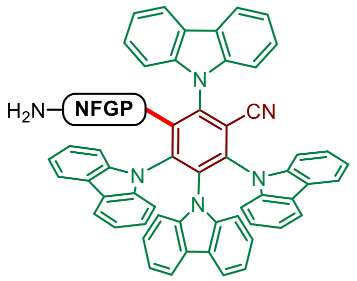

Following the general procedure, the reaction was conducted on a10 μmol scale. The desired product **3ea** (9.3 mg, 8.1 μmol, 81% yield) was isolated by **Method 3**.

HPLC-UV chromatogram (210 nm) of the crude by **Method 1**:

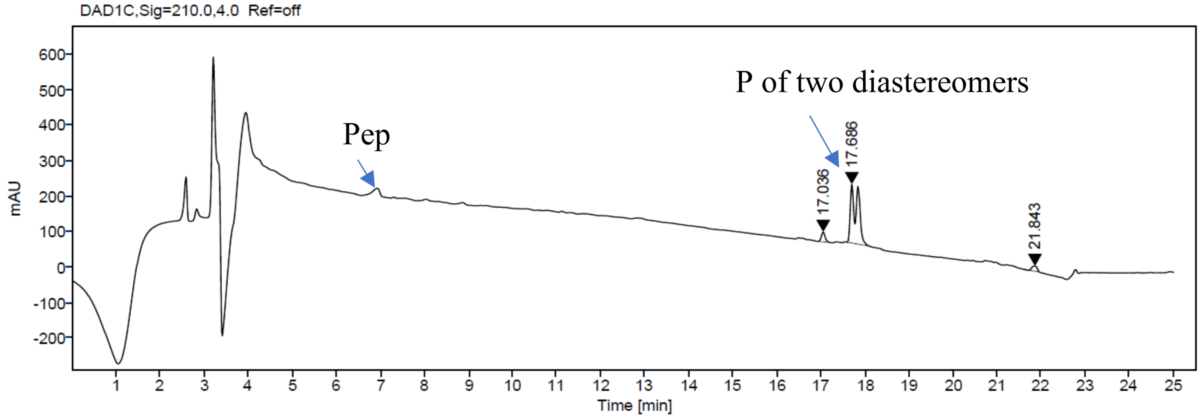

HPLC-UV chromatogram (210 nm) of **3ea** by **Method 1**:

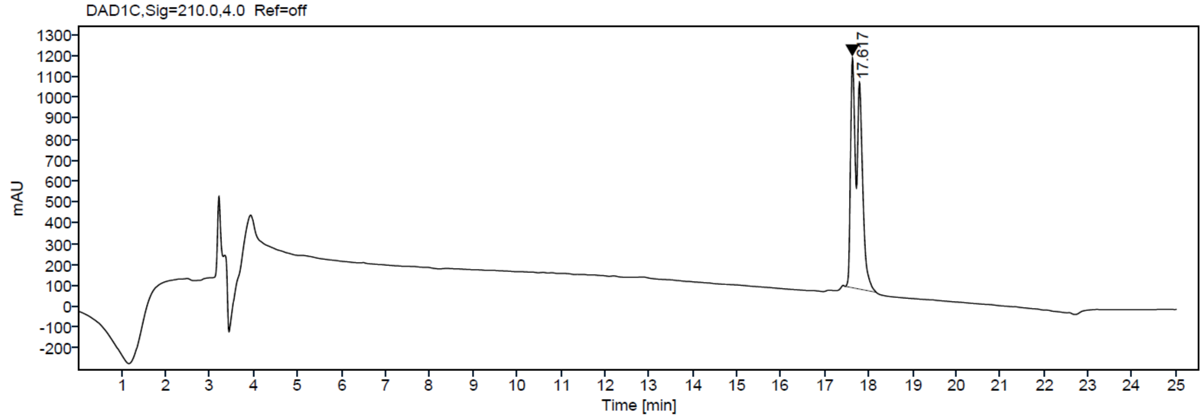

Retention time: 17.824 min **Area Percent:** 100%

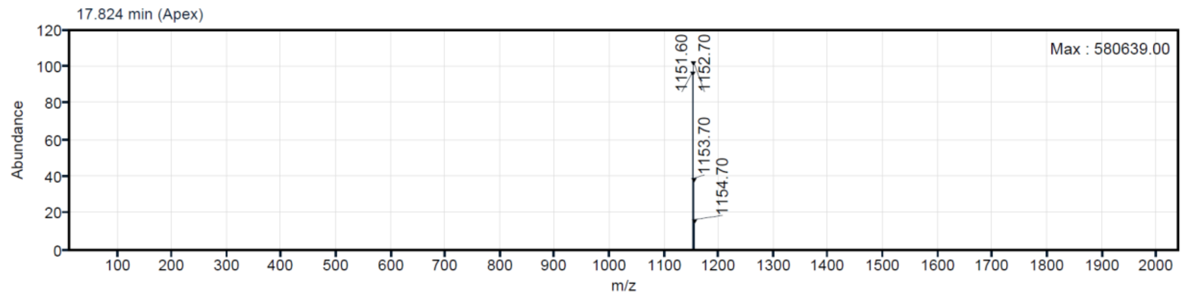

HRMS (nanochip-ESI/LTQ-Orbitrap) m/z: $[M + H]^+$ Calcd for $C_{74}H_{59}N_{10}O_4^+$ 1151.4715; Found 1151.4721.

^{1}H NMR (800 MHz, DMSO, 90 °C) δ 8.31 (t, *J* = 6.5 Hz, 2H), 8.22 – 8.10 (m, 3H), 7.91 – 7.76 (m, 3H), 7.73 (d, *J* = 7.4 Hz, 1H), 7.67 (t, *J* = 7.6 Hz, 2H), 7.63 – 7.57 (m, 2H), 7.55 – 7.46 (m, 3H), 7.41 (dq, *J* = 10.6, 6.4, 5.1 Hz, 4H), 7.33 (q, *J* = 7.4, 6.7 Hz, 5H), 7.20 (q, *J* = 7.7 Hz, 3H), 7.17 – 7.11 (m, 2H), 6.88 (d, *J* = 13.4 Hz, 2H), 6.73 – 6.67 (m, 3H), 6.65 (t, *J* = 7.8 Hz, 2H), 4.81 – 4.59 (m, 2H), 3.50 – 3.44 (m, 1H), 3.07 – 2.98 (m, 1H), 2.88 (d, *J* = 11.3 Hz, 1H), 2.80 – 2.73 (m, 1H), 2.70 (s, 1H), 2.43 – 2.35 (m, 2H), 2.29 (d, *J* = 15.7 Hz, 1H), 2.13 – 2.05 (m, 2H), 1.99 – 1.88 (m, 2H), 1.18 (s, 1H), 1.14 – 1.06 (m, 1H), 0.85 – 0.77 (m, 1H).
MS/MS fragmentation of **3ea:**

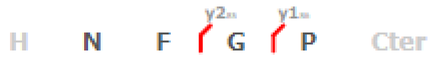

Cter = C54H32N5O-1

Sequence	Type	MF	MF Mass	m/z	Intensity	Similarity
P	y1	C59H41N6(+1)	833.3393	833.3387	102.78	86.31%
GP	y2	C61H44N7O(+1)	890.3607	890.3602	66.91	85.46%

QFGP 1f

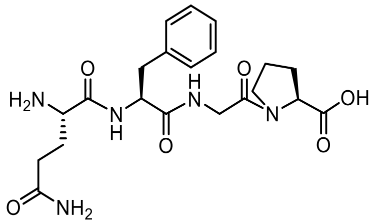

HPLC-UV chromatogram (210 nm) of QFGP (**1f**) by **Method 1**:

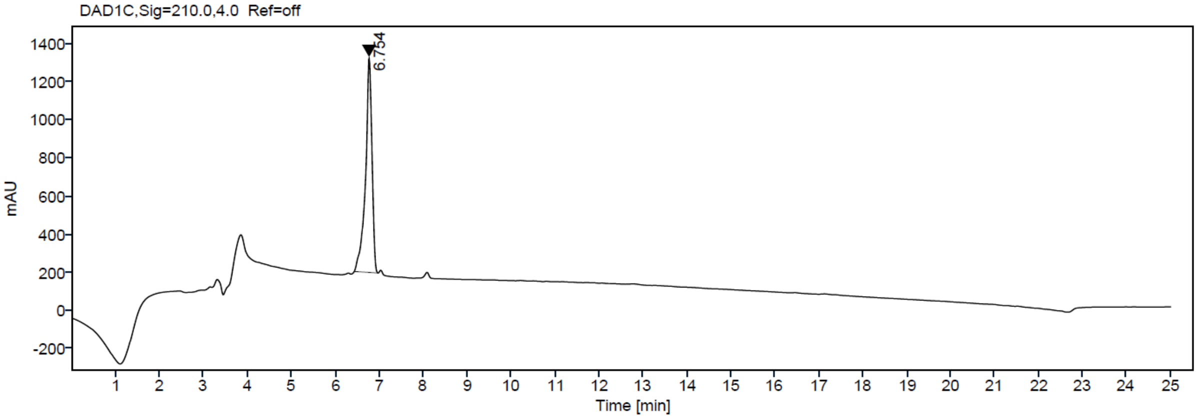

Retention time: 6.79 min **Area Percent:** 100%

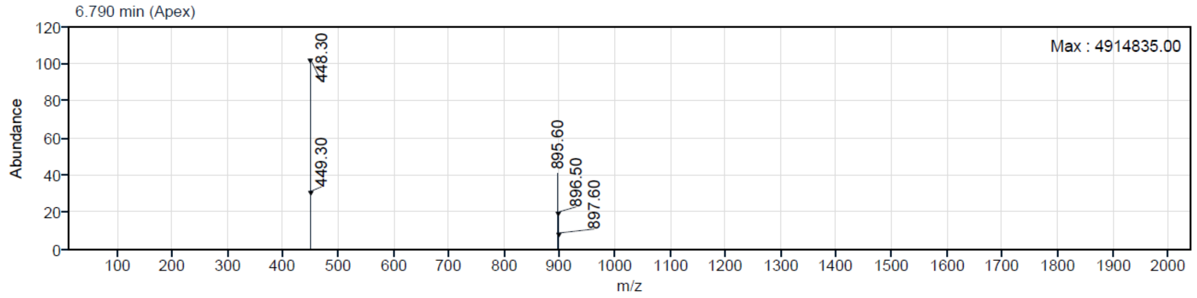

HRMS (nanochip-ESI/LTQ-Orbitrap) m/z: $[M + H]^+$ Calcd for $C_{21}H_{30}N_5O_6^+$ 448.2191; Found 448.2182.

QFGP 4CzBN 3fa

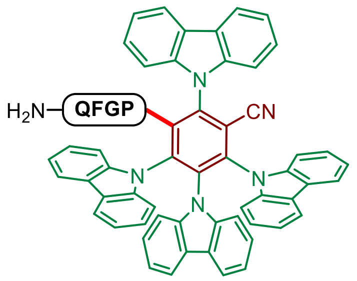

Following the general procedure, the reaction was conducted on a10 μmol scale. The desired product **3fa** (8.8 mg, 7.5 μmol, 75% yield) was isolated by **Method 3.**

HPLC-UV chromatogram (210 nm) of the crude by **Method 1**:

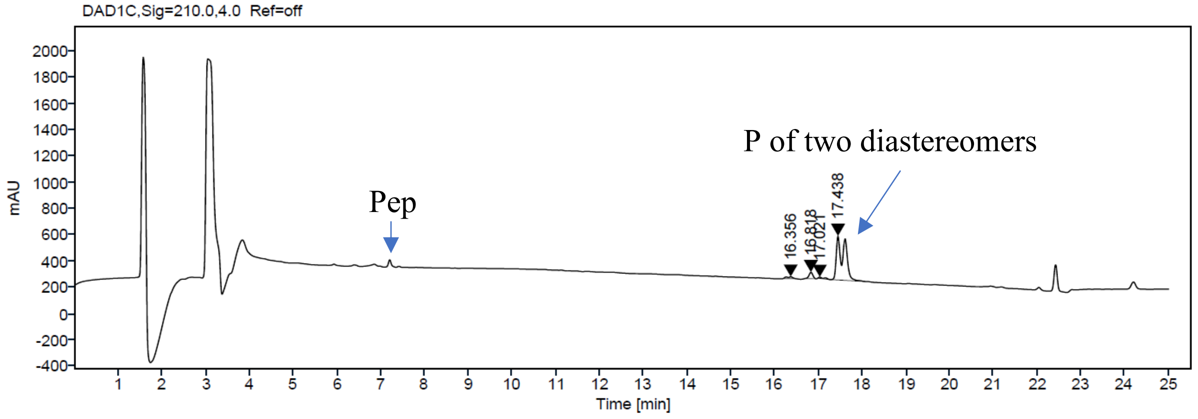

HPLC-UV chromatogram (210 nm) of **3fa** by **Method 1**:

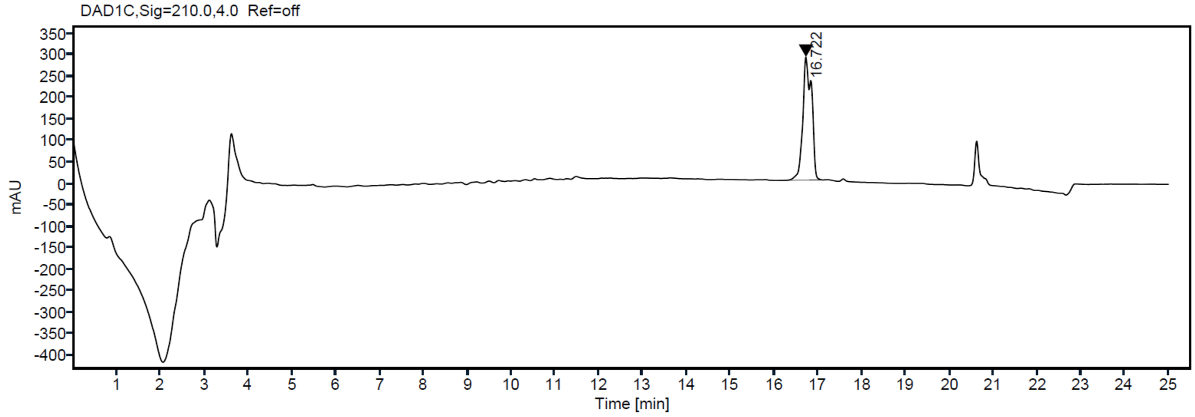

Retention time: 16.863 min **Area Percent:** 100%

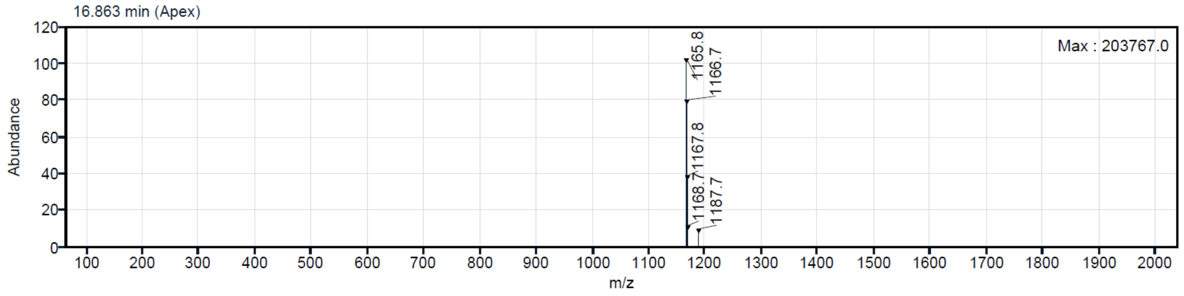

HRMS (nanochip-ESI/LTQ-Orbitrap) m/z: $[M + H]^+$ Calcd for $C_{75}H_{61}N_{10}O_4^+$ 1165.4872; Found 1165.4877.
MS/MS fragmentation of **3fa**

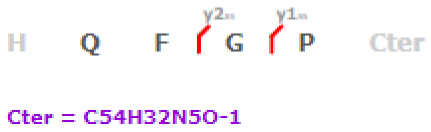

Sequence	Type	MF	MF Mass	m/z	Intensity	Similarity
GP	y2	C61H44N7O(+1)	890.3607	890.3602	74.1	85.32%
P	y1	C59H41N6(+1)	833.3393	833.3387	101.55	84.92%

KFGP 1g

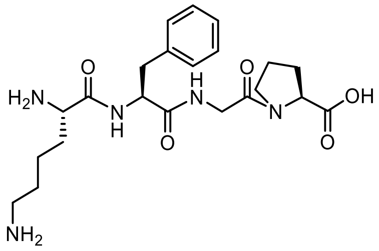

HPLC-UV chromatogram (210 nm) of KFGP (**1g**) by **Method 1**:

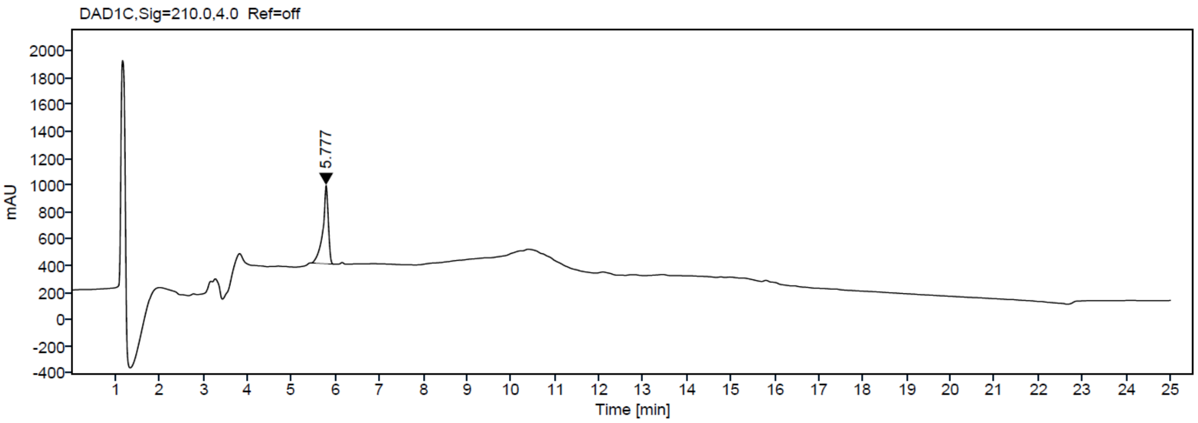

Retention time: 5.793 min **Area Percent:** 100%

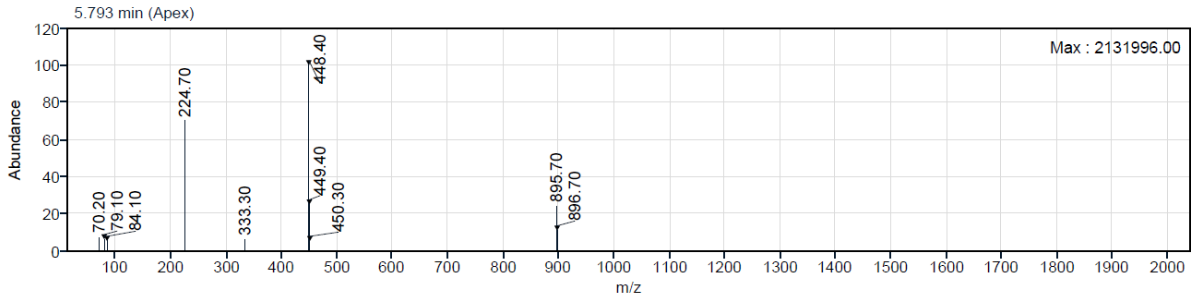

HRMS (nanochip-ESI/LTQ-Orbitrap) m/z: $[M + H]^+$ Calcd for $C_{22}H_{34}N_5O_5^+$ 448.2554; Found 448.2547.

KFGP 4CzBN 3ga

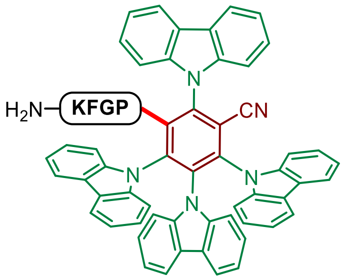

Following the general procedure, the reaction was conducted on a10 μmol scale. The desired product **3ga** (4.9 mg, 4.2 μmol, 42% yield) was isolated by **Method 3.**

HPLC-UV chromatogram (210 nm) of the crude by **Method 1**:

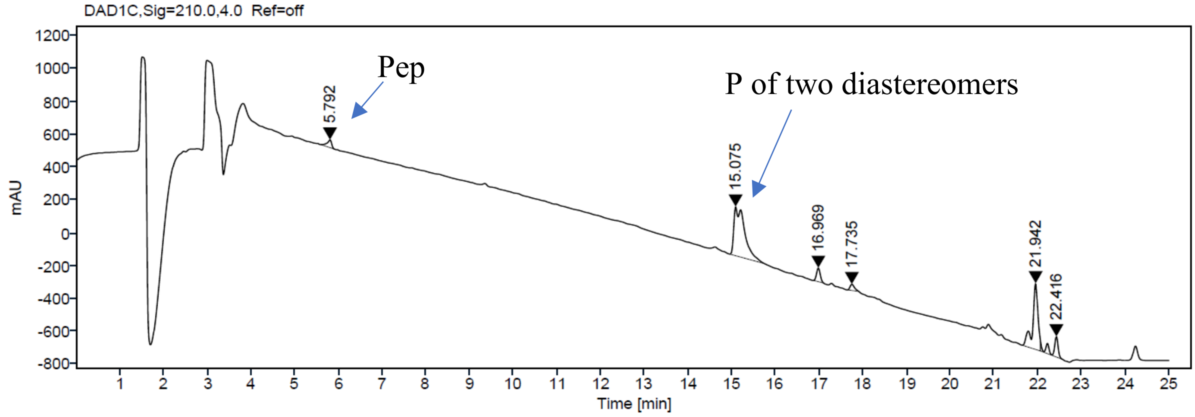

HPLC-UV chromatogram (210 nm) of **3ga** by **Method 1**:

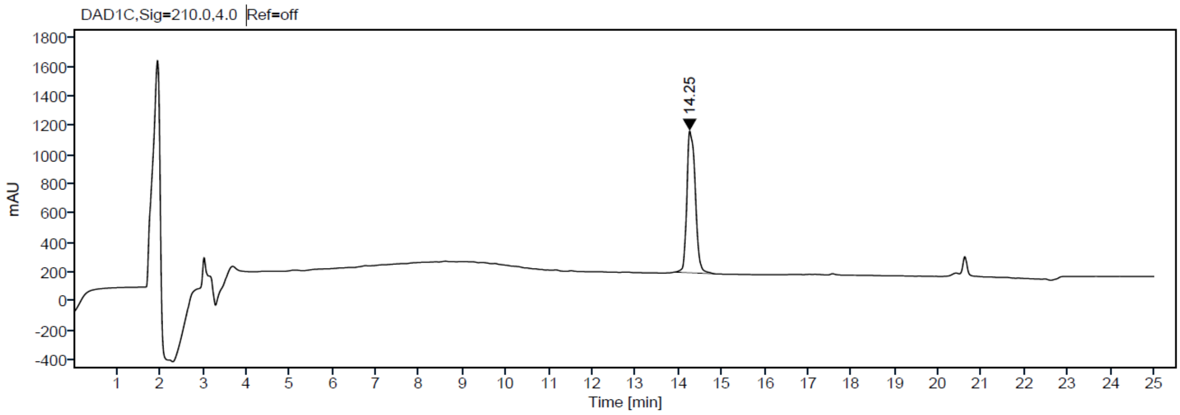

Retention time: 14.347 min **Area Percent:** 100%

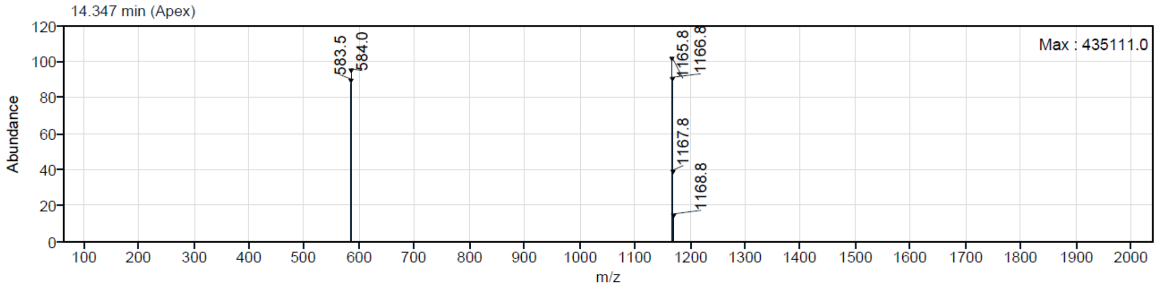

HRMS (nanochip-ESI/LTQ-Orbitrap) m/z: $[M + H]^+$ Calcd for $C_{76}H_{65}N_{10}O_3^+$ 1165.5236; Found 1165.5243.

MS/MS fragmentation of **3ga**

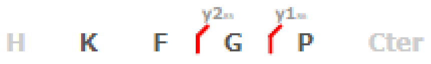

Cter = C54H32O-1N5

Sequence	Type	MF	MF Mass	m/z	Intensity	Similarity
P	y1	C59H41N6(+1)	833.3393	833.3387	91.09	86.31%
GP	y2	C61H44N7O(+1)	890.3607	890.3602	102.15	85.32%

SFGP 1h

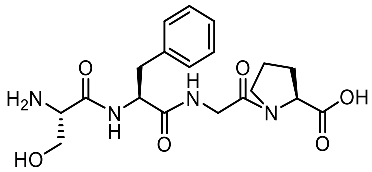

HPLC-UV chromatogram (210 nm) of SFGP (**1h**) by **Method 1**:

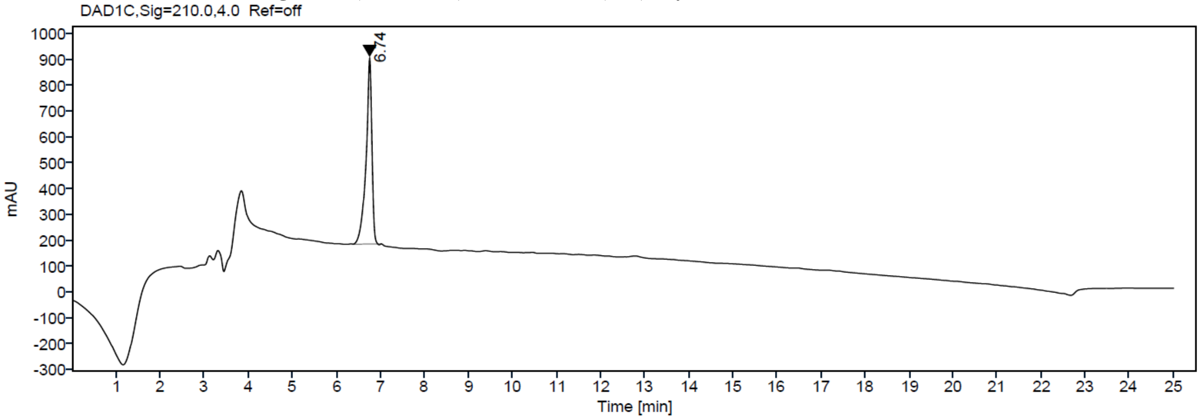

Retention time: 6.765 min **Area Percent:** 100%

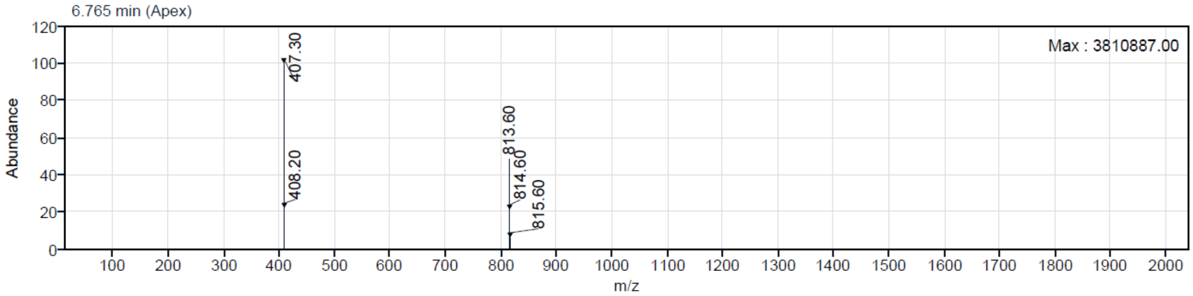

HRMS (ESI/QTOF) m/z: $[M + Na]^+$ Calcd for $C_{21}H_{28}N_4NaO_7^+$ 471.1850; Found 471.1862.

SFGP 4CzBN 3ha

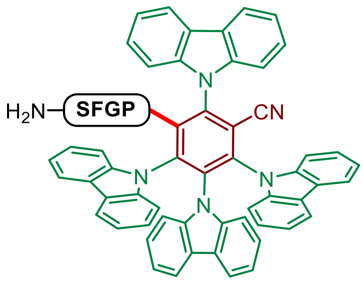

Following the general procedure, the reaction was conducted on a10 μmol scale. The desired product **3ha** (8.9 mg, 8.0 μmol, 80% yield) was isolated by **Method 3.**

HPLC-UV chromatogram (210 nm) of the crude by **Method 1**:

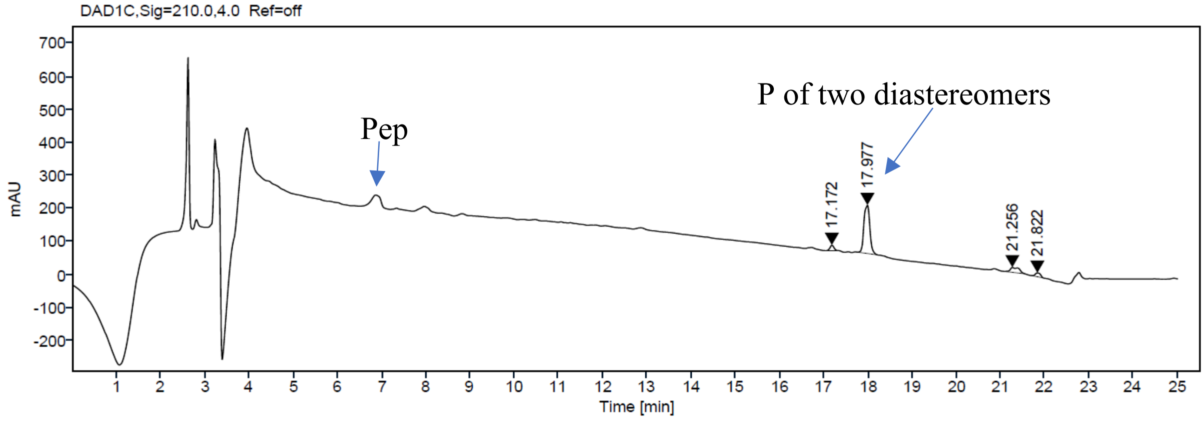

HPLC-UV chromatogram (210 nm) of **3ha** by **Method 1**:

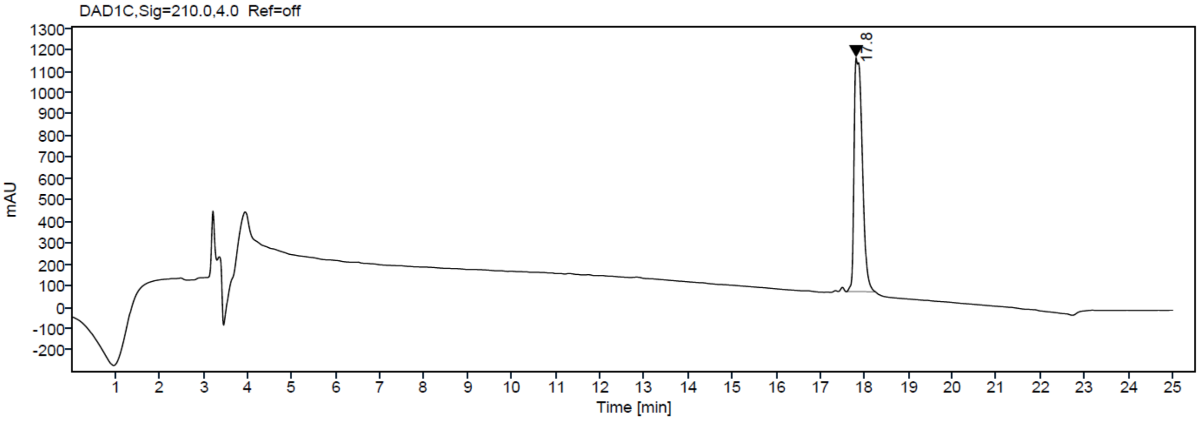

Retention time: 17.94 min **Area Percent:** 100%

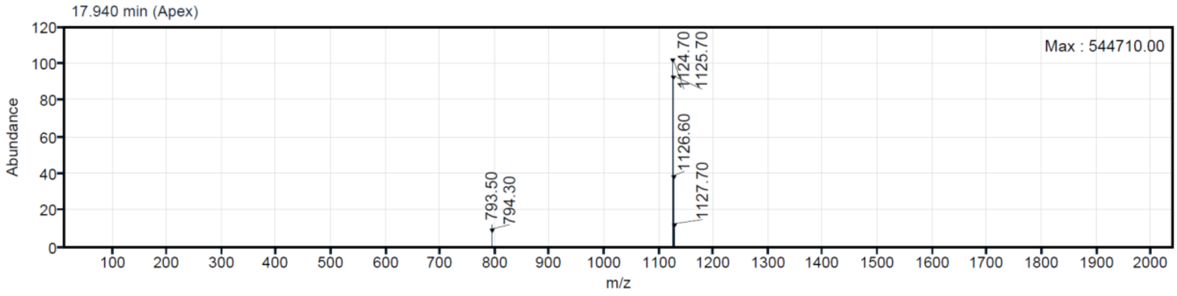

HRMS (ESI/QTOF) m/z: $[M + H]^+$ Calcd for $C_{73}H_{58}N_9O_4^+$ 1124.4606; Found 1124.4590.

MS/MS fragmentation of **3ha**:

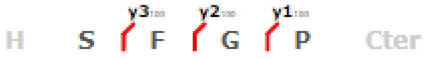

Cter = C54H32N5O-1

Sequence	Type	MF	MF Mass	m/z	Intensity	Similarity
FGP	y3	C70H53N8O2(+1)	1037.429	1037.429	0.74	99.99%
P	y1	C59H41N6(+1)	833.3393	833.3387	10.7	99.97%
SFGP		C73H57N9O4	1123.453	1124.461	33.23	99.95%
GP	y2	C61H44N7O(+1)	890.3607	890.3602	104.08	99.95%

TFGP 1i

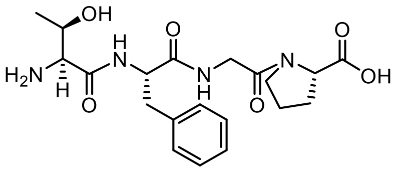

HPLC-UV chromatogram (210 nm) of TFGP (**1i**) by **Method 1**:

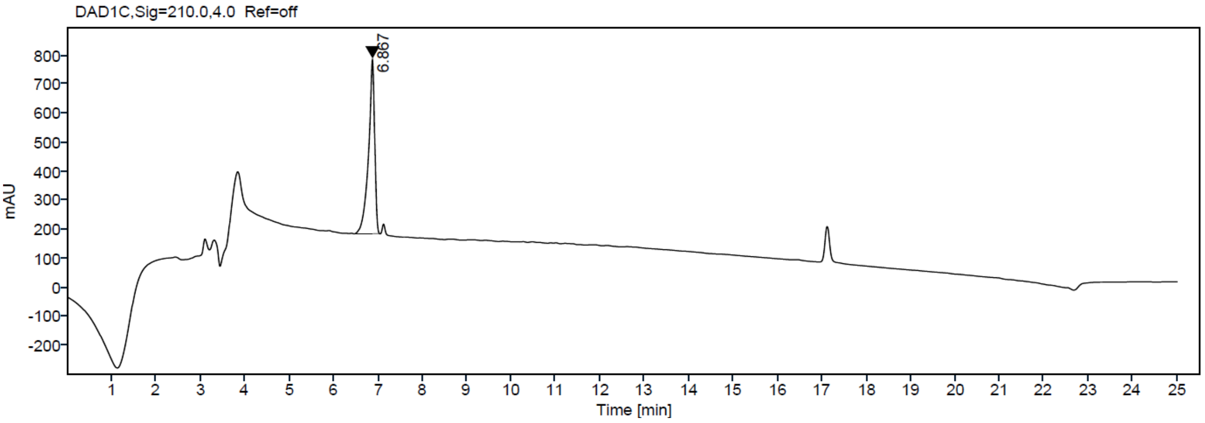

Retention time: 6.899 min **Area Percent:** 100%

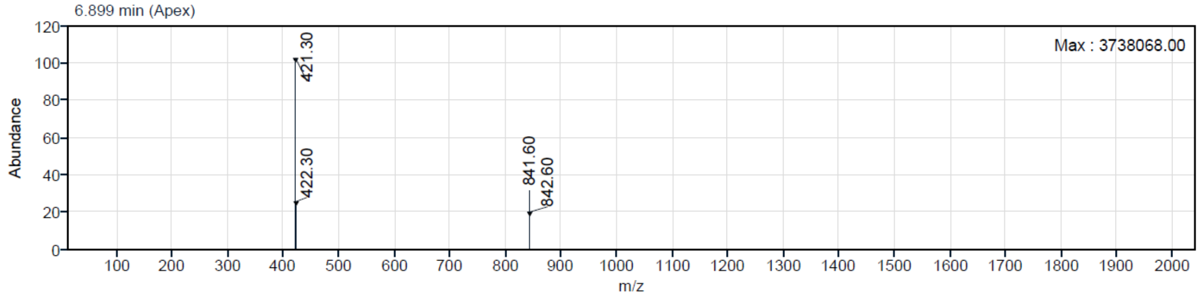

HRMS (nanochip-ESI/LTQ-Orbitrap) m/z: $[M + H]^+$ Calcd for $C_{20}H_{29}N_4O_6^+$ 421.2082; Found 421.2076.

TFGP 4CzBN 1i

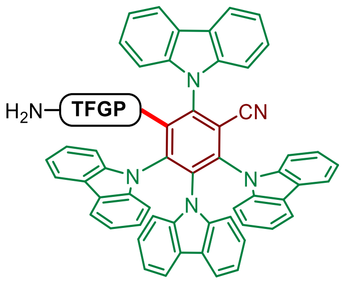

Following the general procedure, the reaction was conducted on a10 μmol scale. The desired product **3ia** (8.9 mg, 7.8 μmol, 78% yield) was isolated by **Method 3.**

HPLC-UV chromatogram (210 nm) of the crude by **Method 1**:

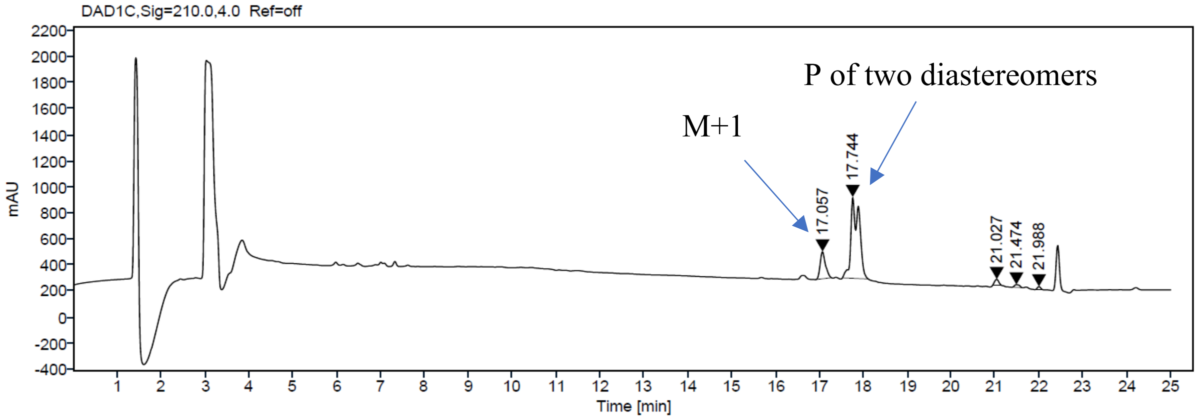

HPLC-UV chromatogram (210 nm) of **3ia** by **Method 1**:

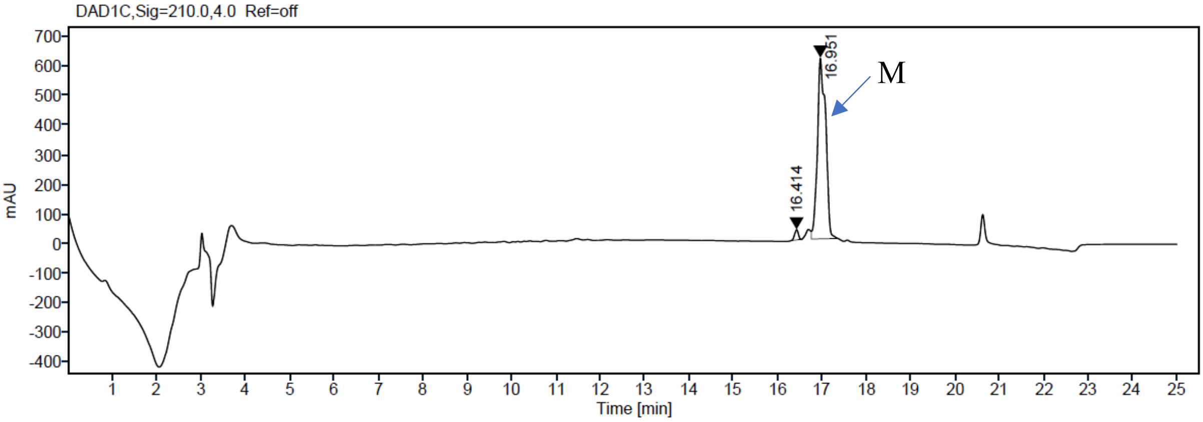

Retention time: 17.925 min **Area Percent:** 100%

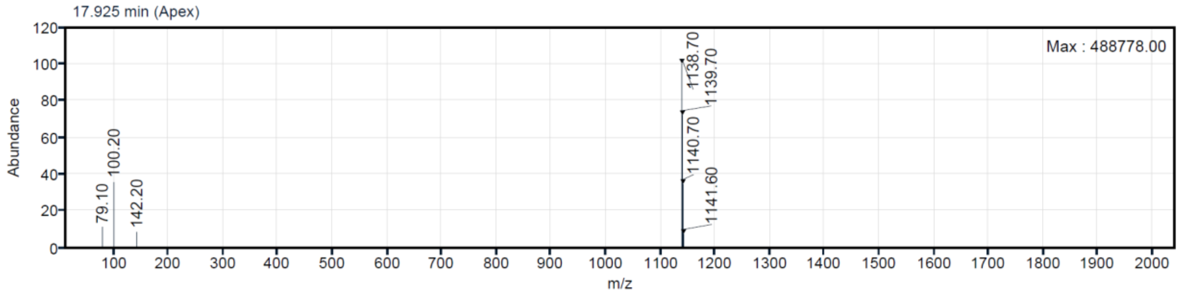

HRMS (nanochip-ESI/LTQ-Orbitrap) m/z: $[M + H]^+$ Calcd for $C_{74}H_{60}N_9O_4^+$ 1138.4763; Found 1138.4768.

MS/MS fragmentation of **3ia**:

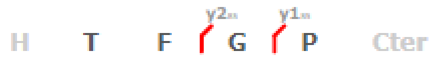

Cter = C54H32N5O-1

Sequence	Type	MF	MF Mass	m/z	Intensity	Similarity
GP	y2	C61H44N7O(+1)	890.3607	890.3602	102.25	85.36%
P	y1	C59H41N6(+1)	833.3393	833.3387	81.67	84.86%

PraFGP 1j

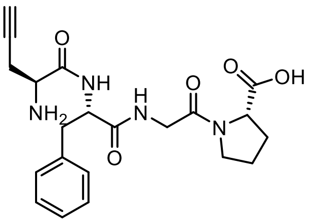

HPLC-UV chromatogram (210 nm) of PraFGP (**1j**) by **Method 1**:

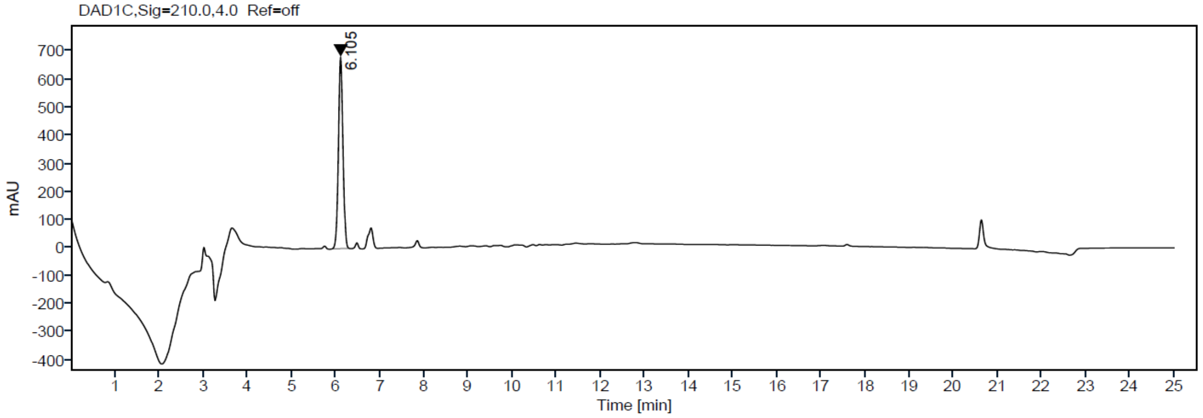

Retention time: 6.126 min **Area Percent:** 100%

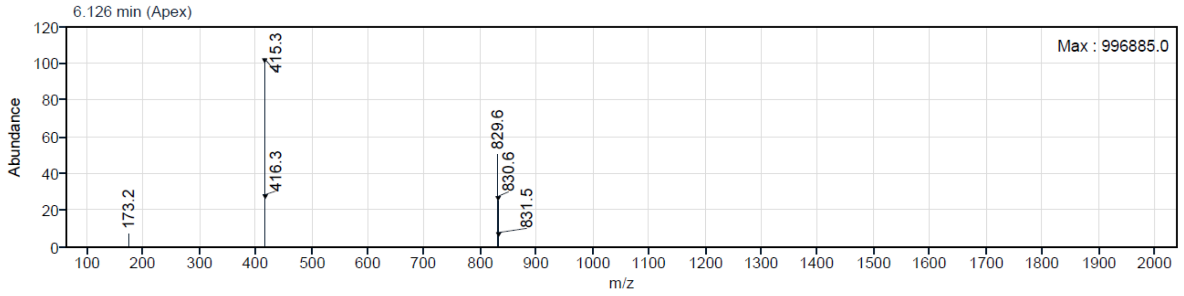

HRMS (LTQ-Orbitrap) m/z: $[M + H]^+$ Calcd for $C_{21}H_{27}N_4O_5^+$ 415.1976; Found 415.1970.

PraFGP 4CzBN 3ja

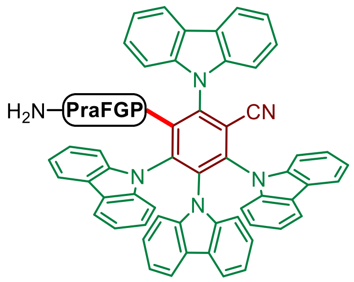

Following the general procedure, the reaction was conducted on a10 µmol scale. The desired product **3ja** (5.9 mg, 5.2 µmol, 52% yield) was isolated by **Method 3.**

HPLC-UV chromatogram (210 nm) of the crude by **Method 1**:

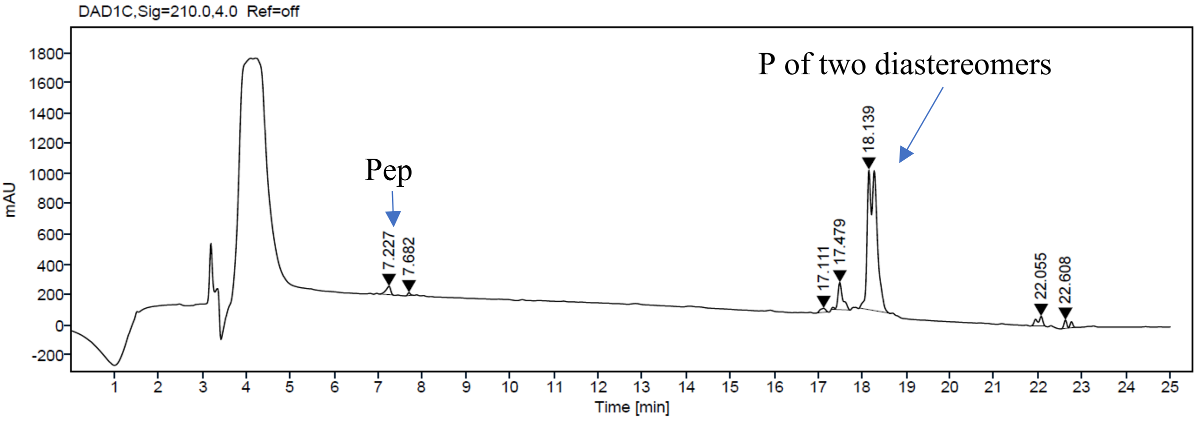

HPLC-UV chromatogram (210 nm) of **3ja** by **Method 1**:

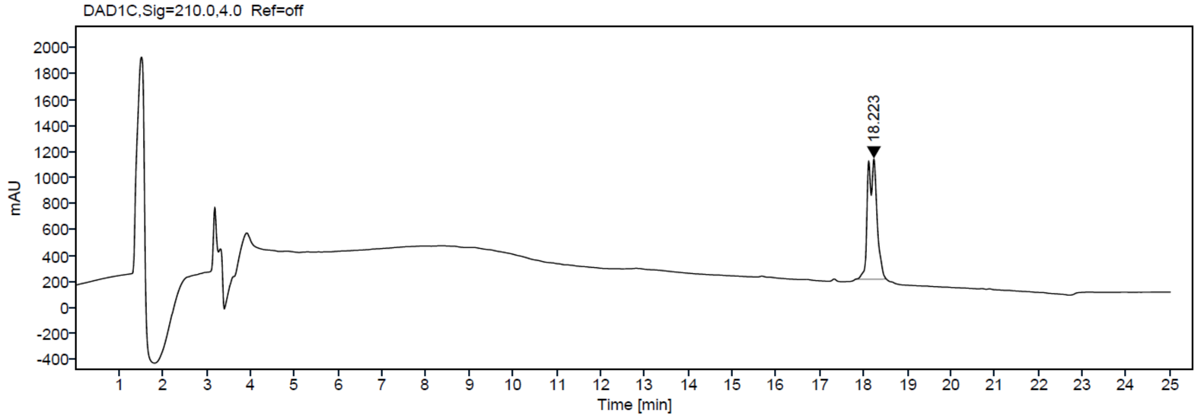

Retention time: 18.249 min **Area Percent:** 100%

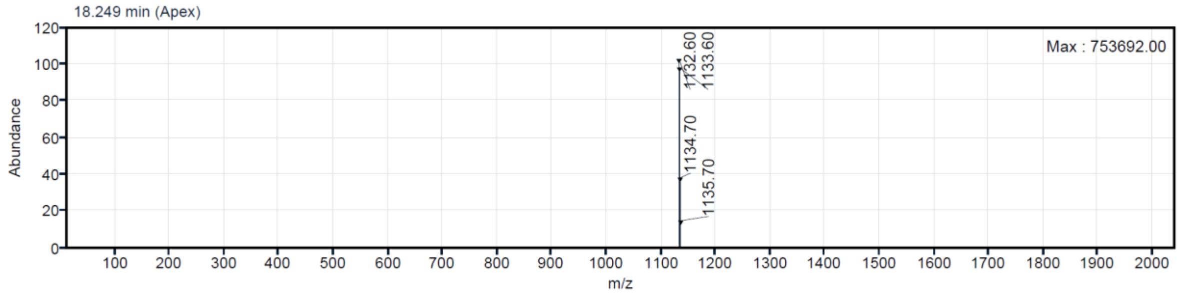

HRMS (nanochip-ESI/LTQ-Orbitrap) m/z: $[M + H]^+$ Calcd for $C_{75}H_{58}N_9O_3^+$ 1132.4657; Found 1132.4622.

MS/MS fragmentation of **3ja**:

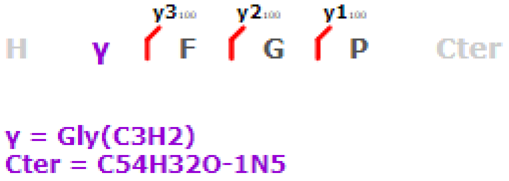

Sequence	Type	MF	MF Mass	m/z	Intensity	Similarity
FGP	y3	C70H53N8O2(+1)	1037.429	1037.429	0.82	100.00%
P	y1	C59H41N6(+1)	833.3393	833.3387	11.88	99.97%
GP	y2	C61H44N7O(+1)	890.3607	890.3602	101.73	99.97%

AcHFGA 1l

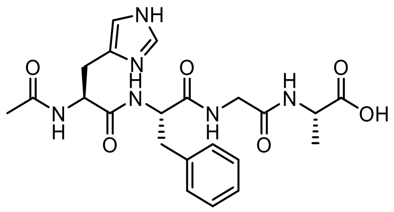

HPLC-UV chromatogram (210 nm) of AcHFGA (**1l**) by **Method 3**:

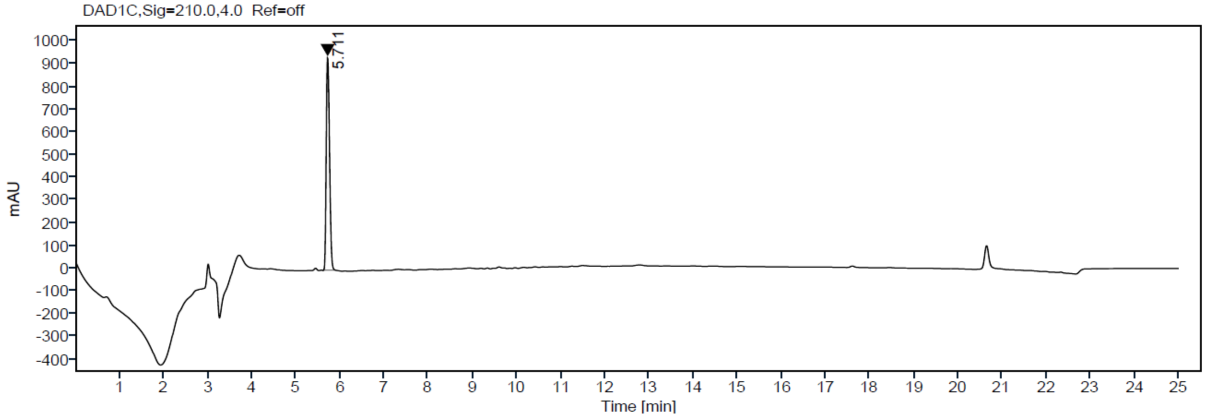

Retention time: 5.737 min **Area Percent:** 100%

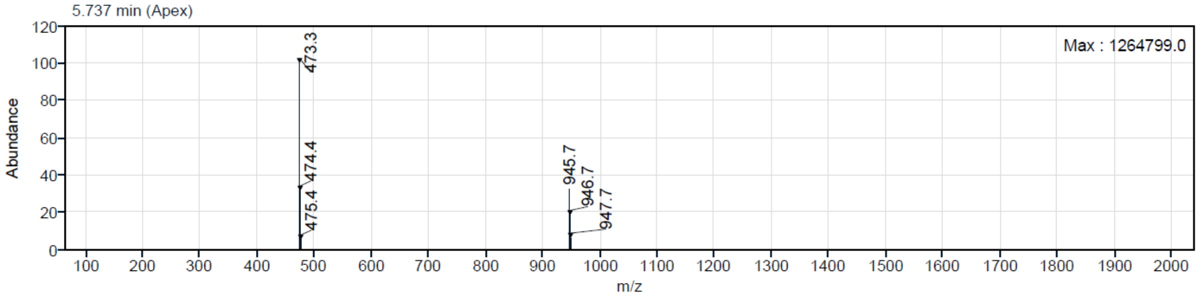

HRMS (nanochip-ESI/LTQ-Orbitrap) m/z: $[M + H]^+$ Calcd for $C_{22}H_{29}N_6O_6^+$ 473.2143; Found 473.2134.

AcHFGA 4CzBN 3la

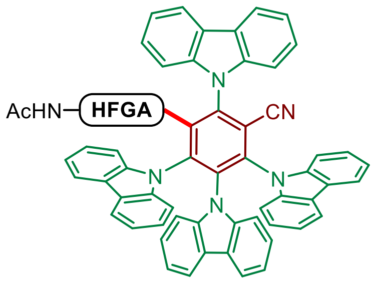

Following the general procedure (Kessil lamp 440 nm), the reaction was conducted on a10 μmol scale with **Kessil lamp (440 nm)** irradiation for 1 hour. The desired product **3la** (2.1 mg, 1.8 μmol, 18% yield) was isolated by **Method 2.**

HPLC-UV chromatogram (210 nm) of the crude by **Method 1**:

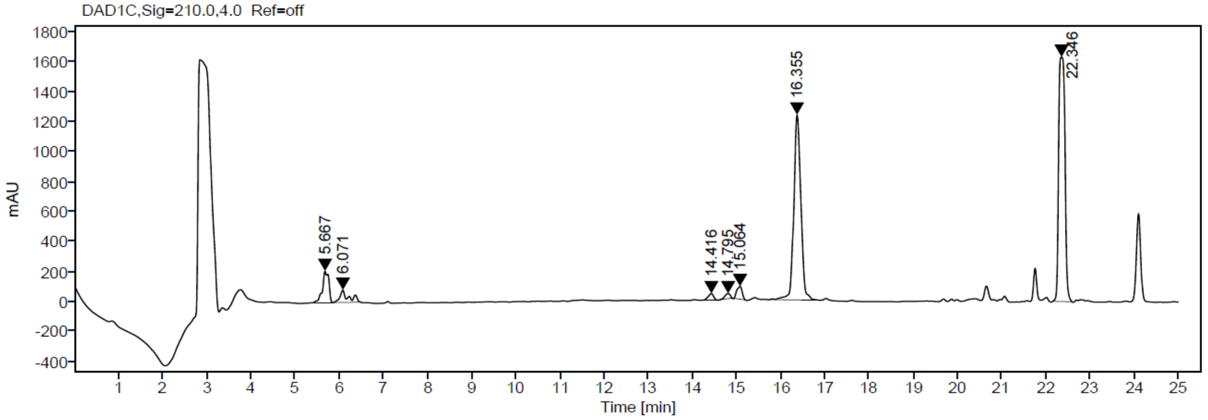

HPLC-UV chromatogram (210 nm) of **3la** by **Method 1**:

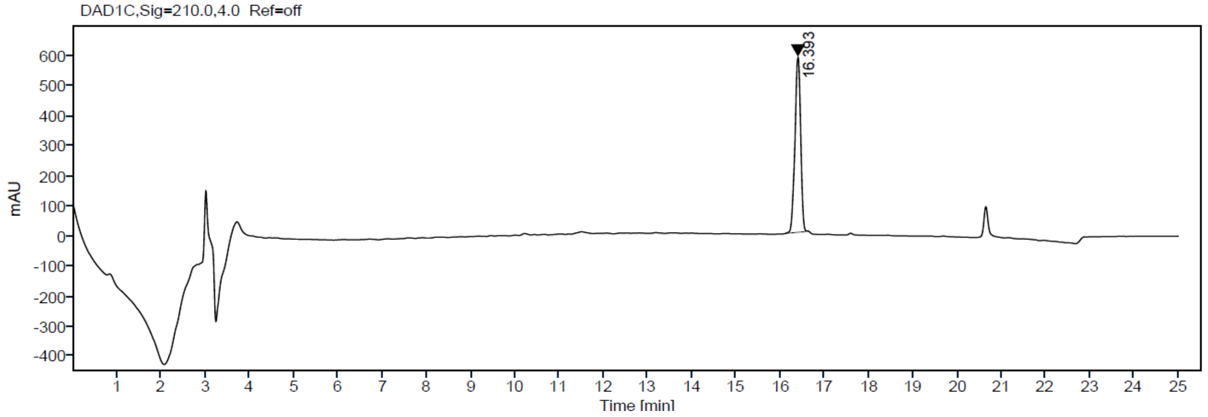

Retention time: 16.438 min **Area Percent:** 100%

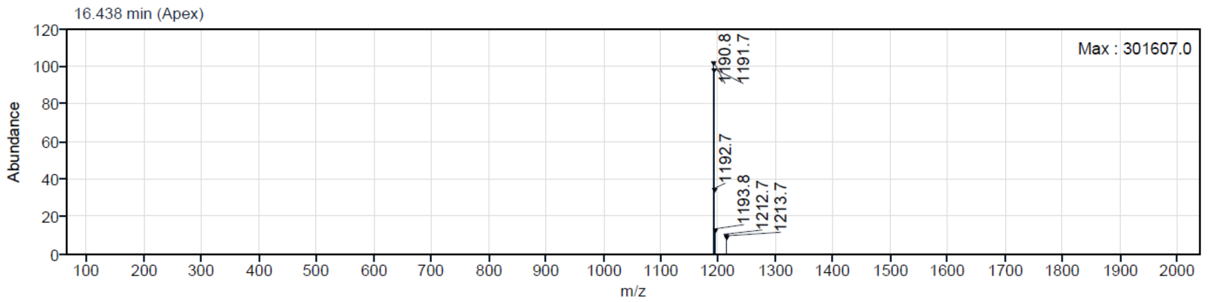

MS/MS fragmentation of **3la**:

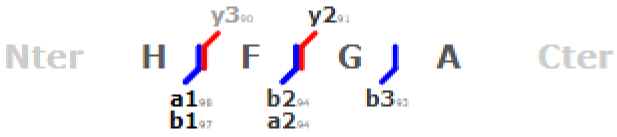

Nter = C2H3O
Cter = C54H32N5O-1

Sequence	Type	MF	MF Mass	m/z	Intensity	Similarity
H	a1	C7H10N3O(+1)	152.08	152.08	24.44	97.60%
H	b1	C8H10N3O2(+1)	180.08	180.08	100.14	97.41%
HF	b2	C17H19N4O3(+1)	327.15	327.15	42.24	94.45%
HF	a2	C16H19N4O2(+1)	299.15	299.15	2.68	93.83%
HFG	b3	C19H22N5O4(+1)	384.17	384.17	11.84	92.78%
GA	y2	C59H42N7O(+1)	864.35	864.34	14.34	90.93%
FGA	y3	C68H51N8O2(+1)	1011.4	1011.4	6.81	89.95%
HFGA		C76H59N11O4	1189.5	1190.5	62.36	88.84%

AcHFGE 1m

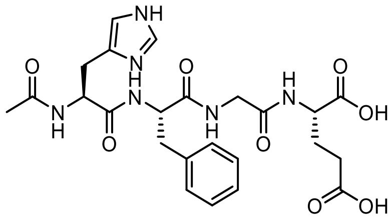

HPLC-UV chromatogram (210 nm) of AcHFGE (**1m**) by **Method 1**:

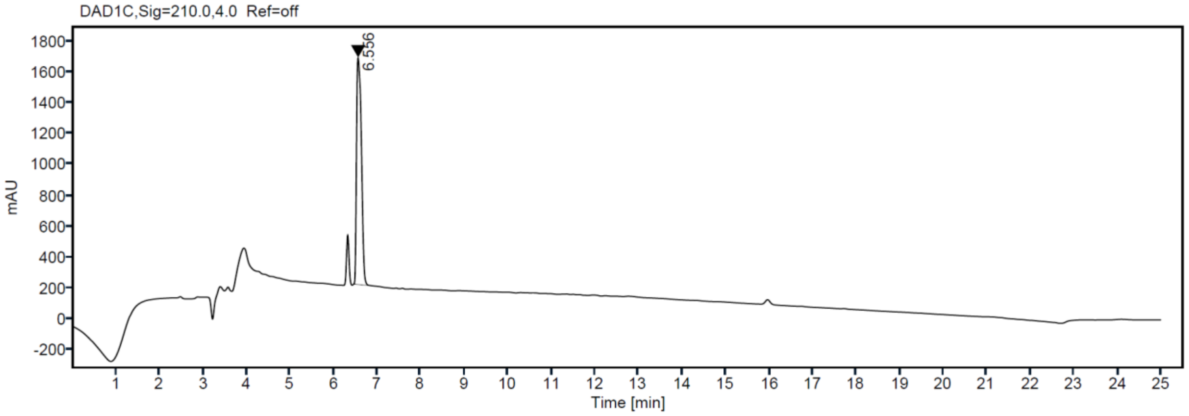

Retention time: 6.58 min **Area Percent:** 100%

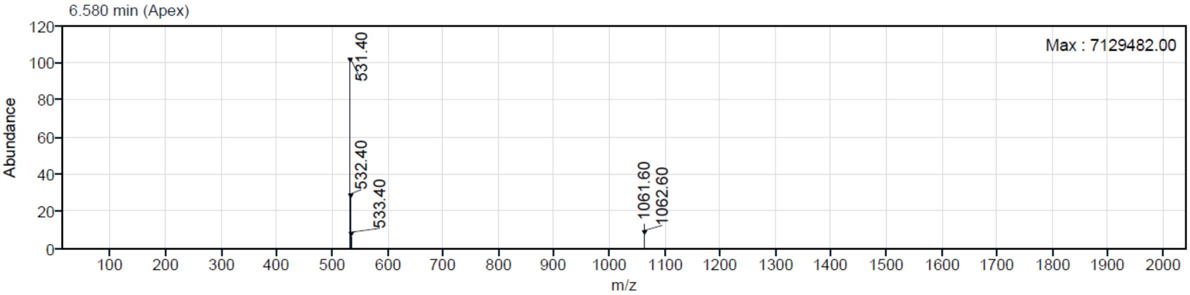

HRMS (nanochip-ESI/LTQ-Orbitrap) m/z: $[M + H]^+$ Calcd for $C_{24}H_{31}N_6O_8^+$ 531.2198; Found 531.2188.

AcHFGE 4CzBN 3ma

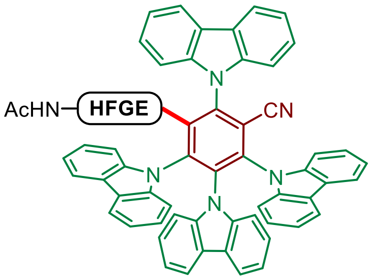

Following the general procedure (reaction time: overnight), the reaction was conducted on a10 µmol scale. The desired product **3ma** (2.5 mg, 2.0 µmol, 20% yield) was isolated by **Method 2.**

HPLC-UV chromatogram (210 nm) of the crude by **Method 1**:

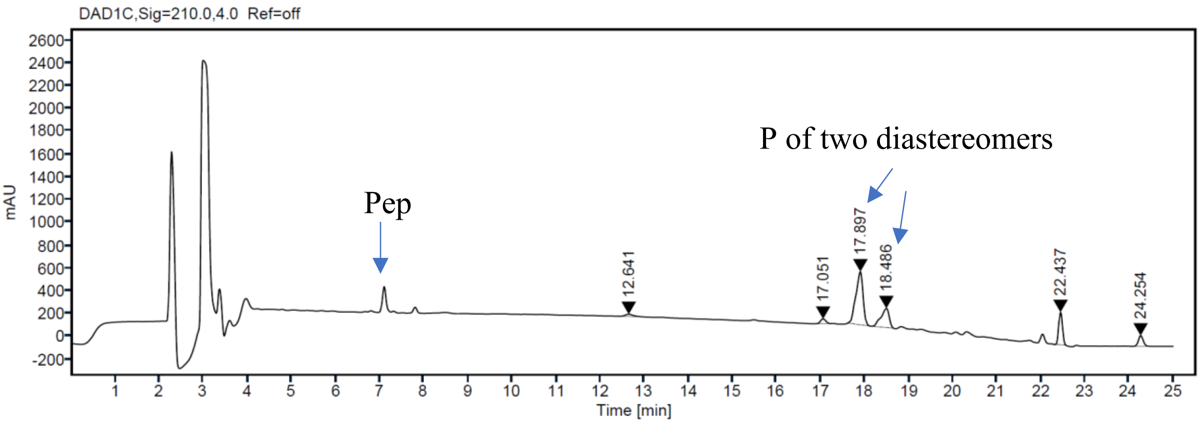

HPLC-UV chromatogram (210 nm) of **3ma** by **Method 1**:

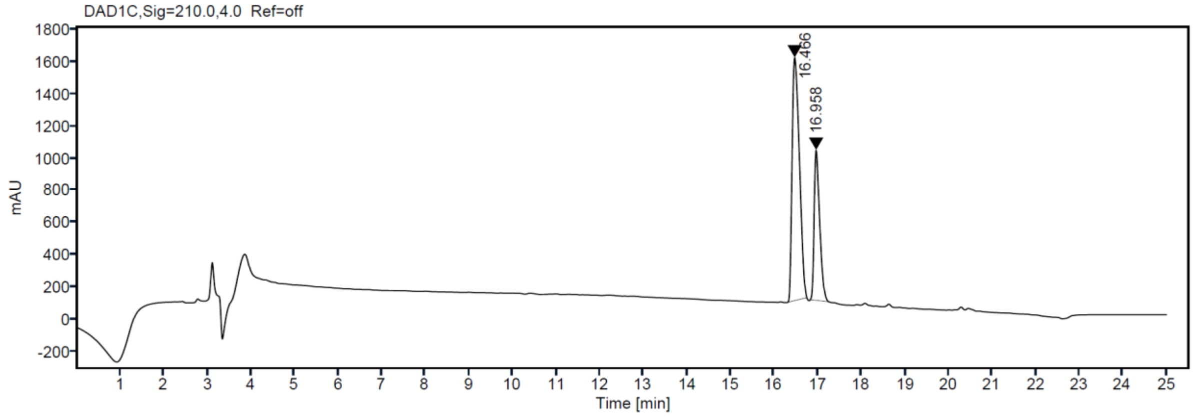

Retention time: 16.993 min **Area Percent:** 71%

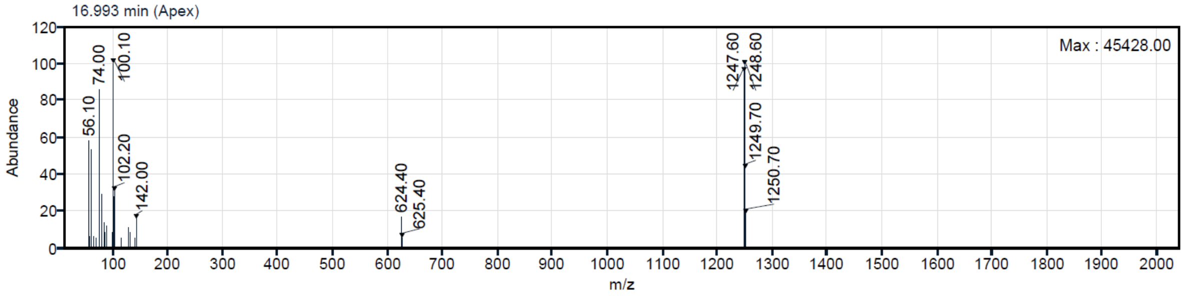

Retention time: 16.518 min **Area Percent:** 29%

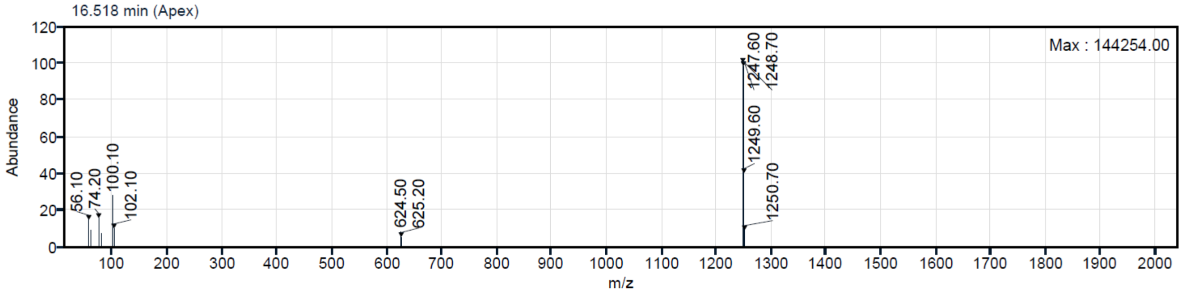

HRMS (nanochip-ESI/LTQ-Orbitrap) m/z: $[M + H]^+$ Calcd for $C_{78}H_{62}N_{11}O_6^+$ 1248.4879; Found 1248.4911.

MS/MS fragmentation of **3ma**:

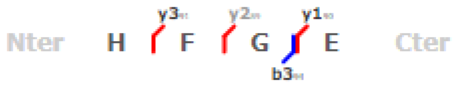

Nter = C2H3O
Cter = C54H32N5O-1

Sequence	Type	MF	MF Mass	m/z	Intensity	Similarity
HFG	b3	C19H22N5O4(+1)	384.1672	384.1666	1.36	94.07%
FGE	y3	C70H53N8O4(+1)	1069.419	1069.418	71.15	90.83%
E	y1	C59H41N6O2(+1)	865.3291	865.3286	8.97	90.05%
GE	y2	C61H44N7O3(+1)	922.3506	922.35	102.35	88.76%

AcHFGF 1n

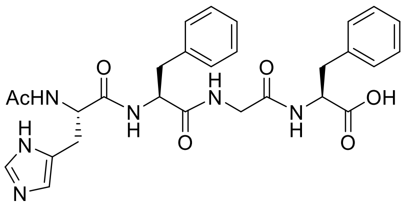

HPLC-UV chromatogram (210 nm) of **1n** by **Method 1**:

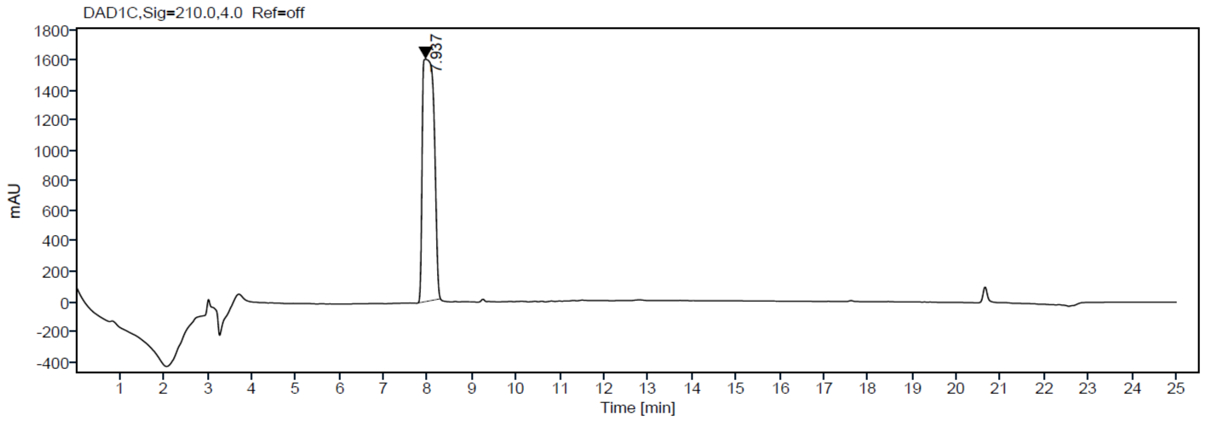

Retention time: 8.083 min **Area Percent:** 100%

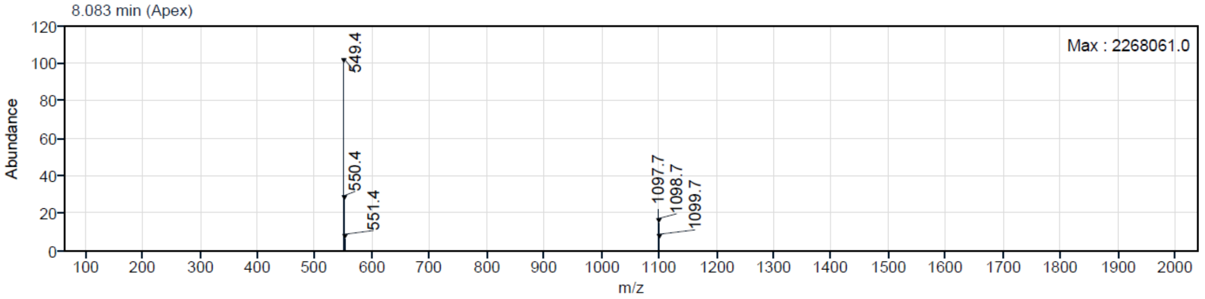

HRMS (nanochip-ESI/LTQ-Orbitrap) m/z: $[M + H]^+$ Calcd for $C_{28}H_{33}N_6O_6^+$ 549.2456; Found 549.2437.

AcHFGF 4CzBN 3na

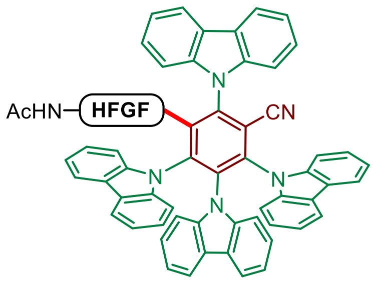

Following the general procedure (Kessil lamp 440 nm), the reaction was conducted on a10 μmol scale with **Kessil lamp (440 nm)** irradiation for 1 hour.. The desired product **3na** (4.3 mg, 0.0034 mmol, 34% yield) was isolated by **Method 2.**
HPLC-UV chromatogram (210 nm) of the crude by **Method 1**:

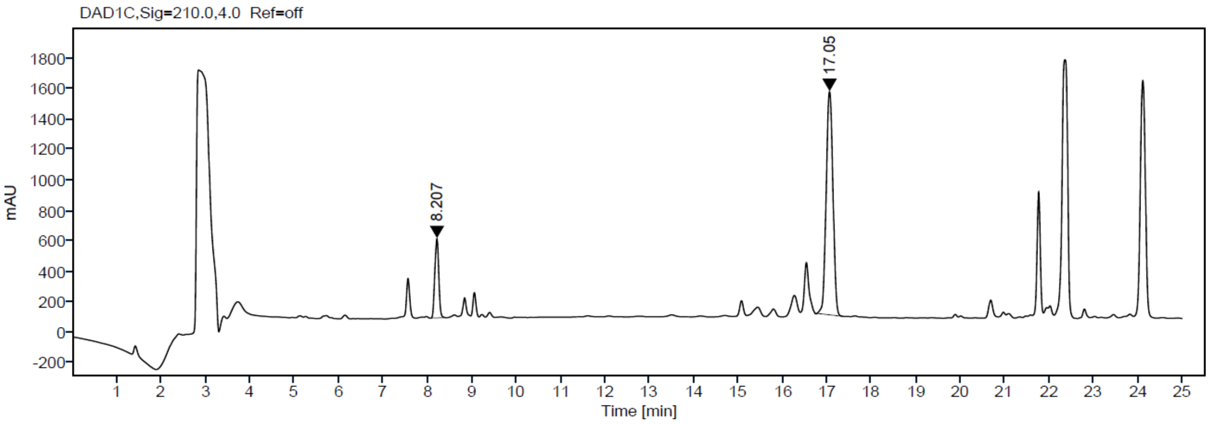

HPLC-UV chromatogram (210 nm) of **3na** by **Method 1**:

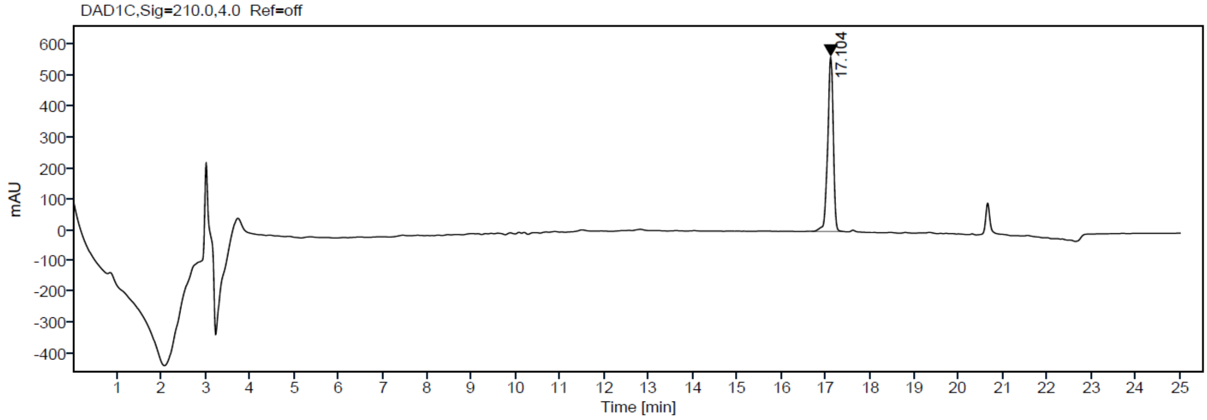

Retention time: 17.134 min **Area Percent:** 100%

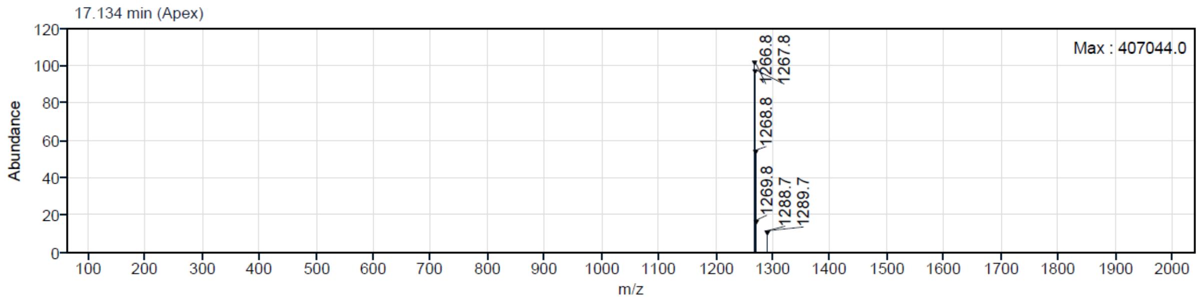

HRMS (nanochip-ESI/LTQ-Orbitrap) m/z: $[M + H]^+$ Calcd for $C_{82}H_{64}N_{11}O_4^+$ 1266.5137; Found 1266.5132.

MS/MS fragmentation of **3na**:

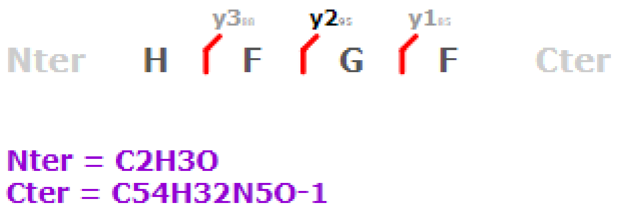

Sequence	Type	MF	MF Mass	m/z	Intensity	Similarity
GF	y2	C65H46N7O(+1)	940.38	940.38	100.2	95.23%
FGF	y3	C74H55N8O2(+1)	1087.4	1087.4	53.11	87.58%

F	y1	C63H43N6(+1)	883.35	883.35	4.26	84.91%

Ac-HFGPip 1o

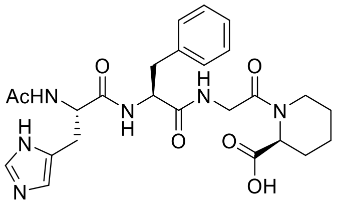

HPLC-UV chromatogram (210 nm) of HFGPip (**1o**) by **Method 1**:

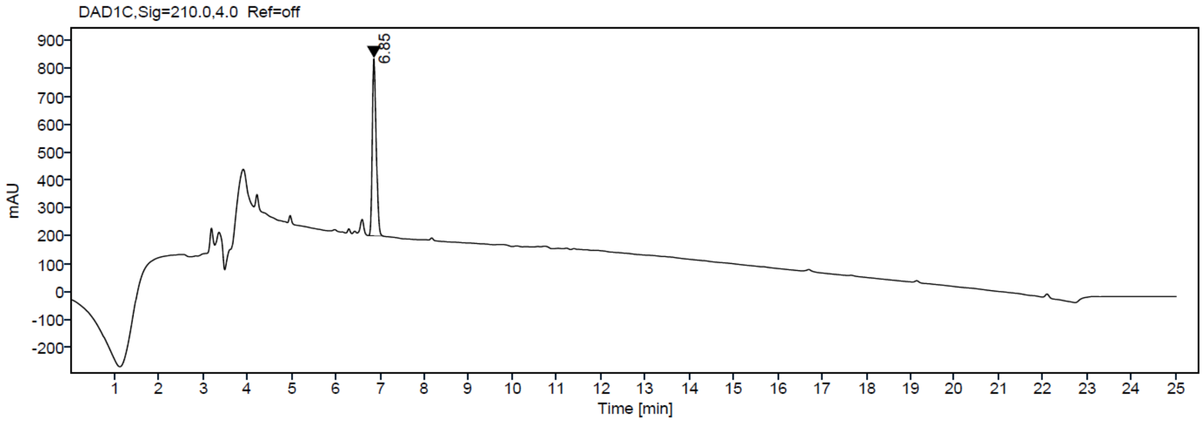

Retention time: 6.864 min **Area Percent:** 100%

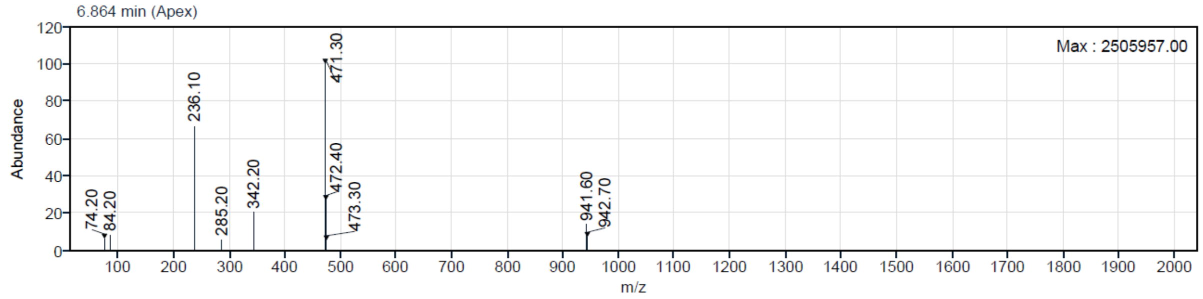

HRMS (LTQ-Orbitrap) m/z: $[M + H]^+$ Calcd for $C_{23}H_{31}N_6O_5^+$ 471.2350; Found 471.2350.

AcHFGPip 4CzBN 3oa

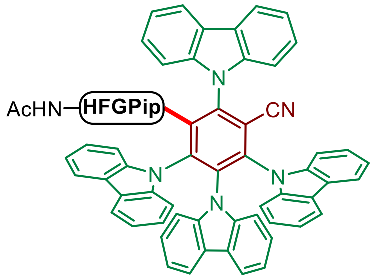

Following the general procedure, the reaction was conducted on a10 μmol scale. The desired product **3oa** (5.5 mg, 4.6 μmol, 46% yield) was isolated by **Method 2.**
HPLC-UV chromatogram (210 nm) of the crude by **Method 1**:

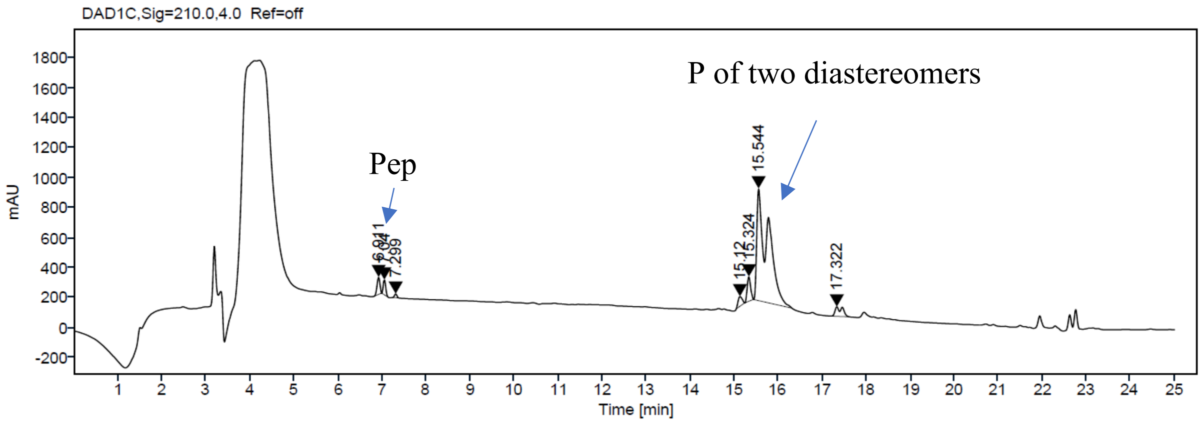

HPLC-UV chromatogram (210 nm) of **3oa** by **Method 1**:

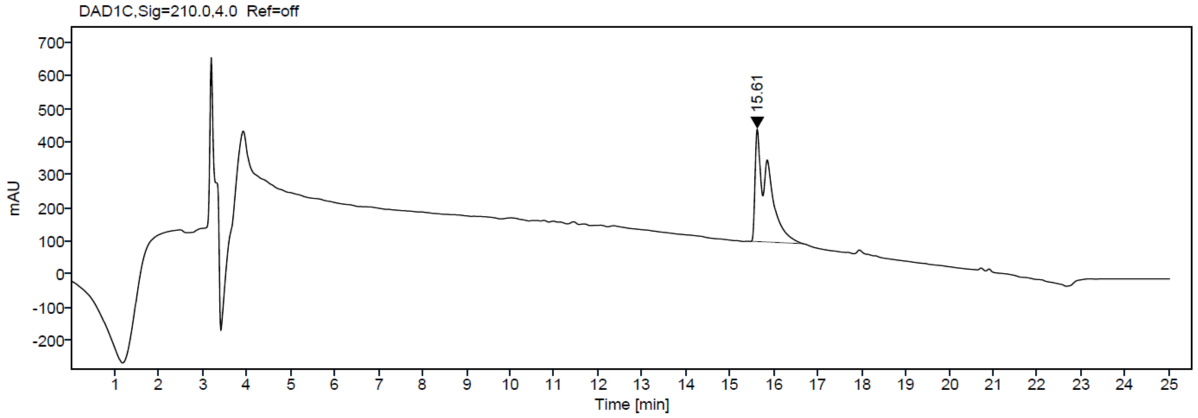

Retention time: 15.912 min **Area Percent:** 100%

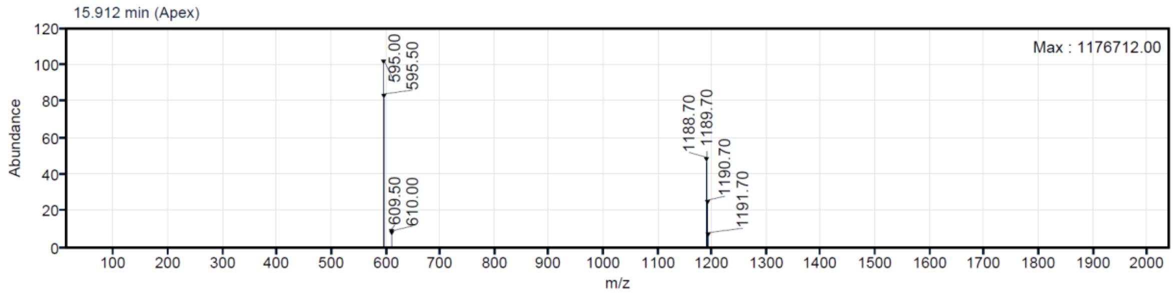

HRMS (nanochip-ESI/LTQ-Orbitrap) m/z: $[M + H]^+$ Calcd for $C_{77}H_{62}N_{11}O_3^+$ 1188.5032; Found 1188.5004.

MS/MS fragmentation of **3oa**

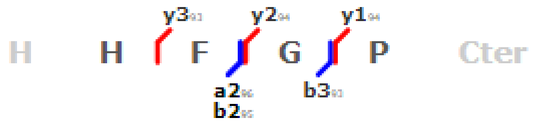

Cter = CH2)(C54H32N5O-1

Sequence	Type	MF	MF Mass	m/z	Intensity	Similarity
HF	a2	C14H17N4O(+1)	257.1402	257.1397	16.24	95.55%

HF	b2	C15H17N4O2(+1)	285.1352	285.1346	48.26	95.20%
Pip	y1	C60H43N6(+1)	847.3549	847.3544	15.95	94.50%
FGPip	y3	C71H55N8O2(+1)	1051.445	1051.444	26.9	94.40%
GPip	y2	C62H46N7O(+1)	904.3764	904.3758	100.15	94.25%
HFG	b3	C17H20N5O3(+1)	342.1566	342.1561	4.31	93.43%
FGPip	y3	C71H55N8O2(+1)	1051.445	526.2258	2.02	92.57%

YPFP 1p

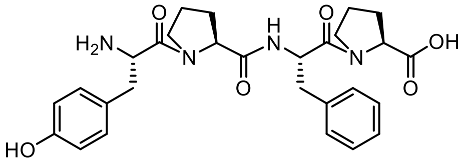

HPLC-UV chromatogram (210 nm) of YPFP (**1p**) by **Method 1**:

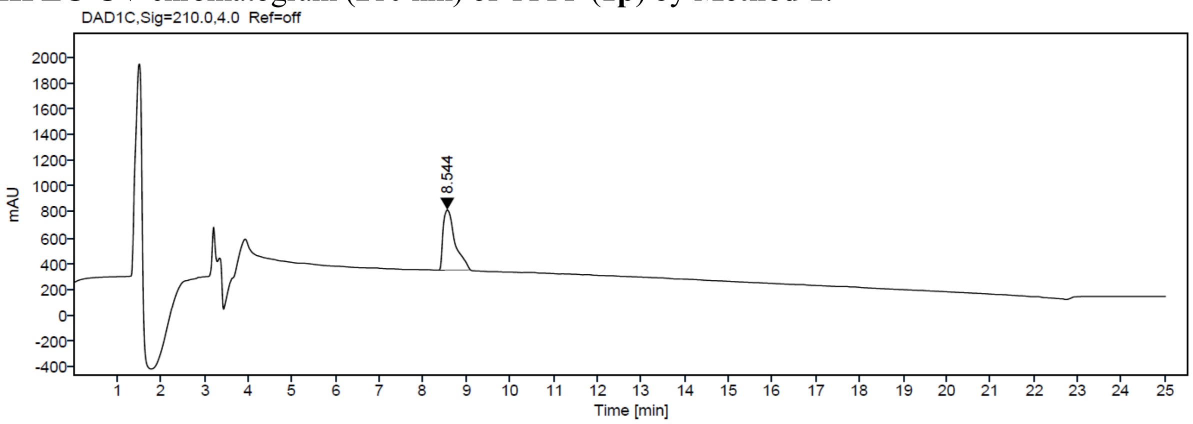

Retention time: 8.586 min **Area Percent:** 100%

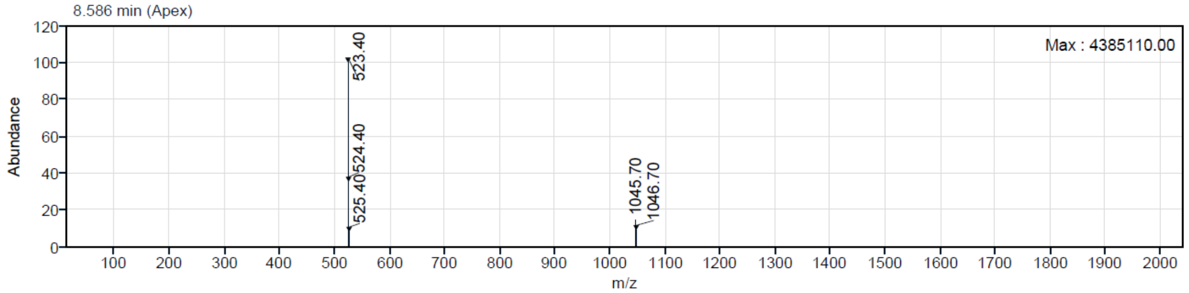

HRMS (ESI/QTOF) m/z: $[M + H_{-1}]^-$ Calcd for $C_{28}H_{33}N_4O_6^-$ 521.2406; Found 521.2411

YPFP 4CzBN 3pa

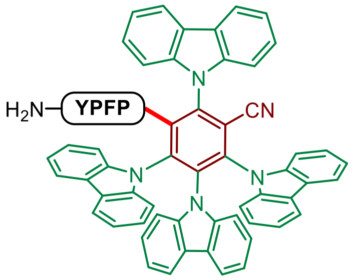

Following the general procedure (reaction time: overnight), the reaction was conducted on a10 μmol scale. The desired product **3pa** (6.5 mg, 5.2 μmol, 52% yield) was isolated by **Method 3**.

More diastereomers derives from atropisomers due to the steric hindrance between 4CzBN and the terminal proline.

HPLC-UV chromatogram (210 nm) of the crude by **Method 1**:

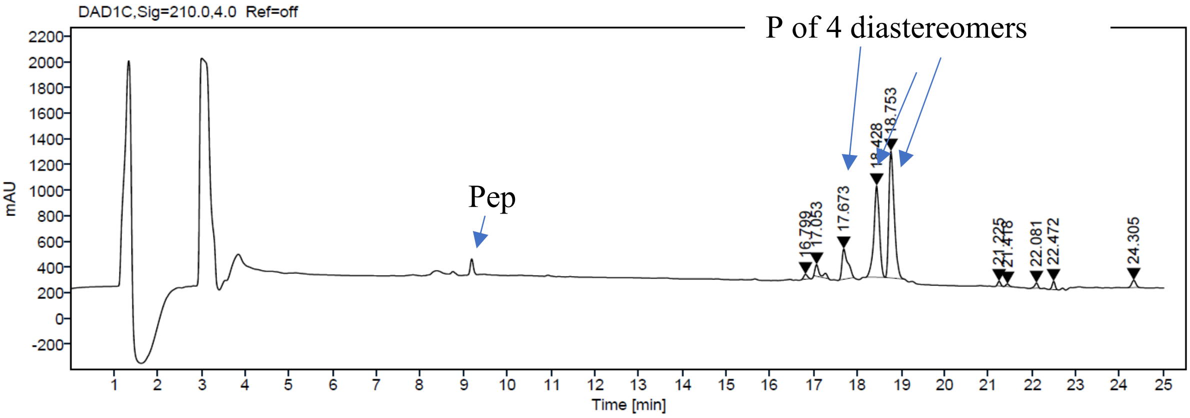

HPLC-UV chromatogram (210 nm) of **3pa** by **Method 1**:

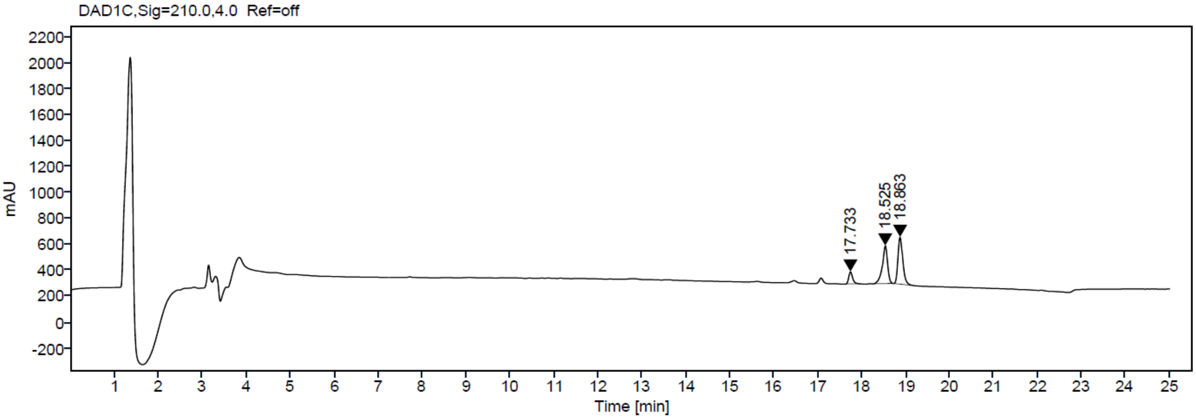

Retention time: 17.757 min **Area Percent:** 21%

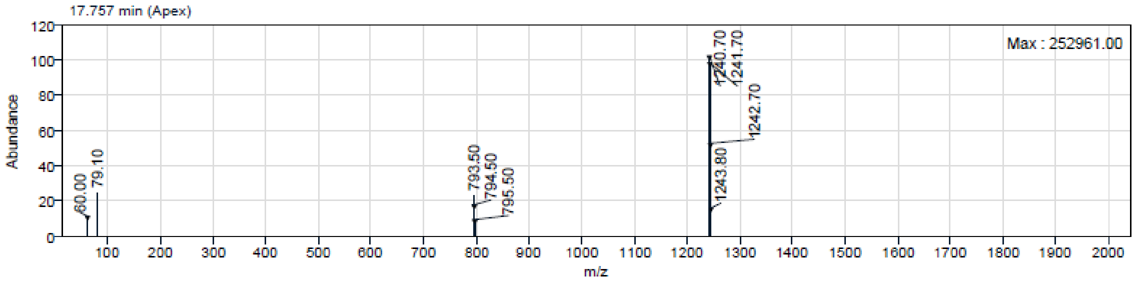

Retention time: 18.55 min **Area Percent:** 23%

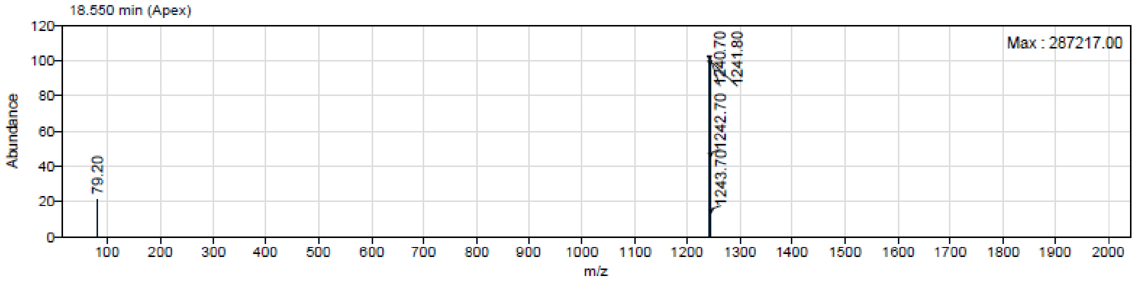

Retention time: 18.901 min **Area Percent:** 56%

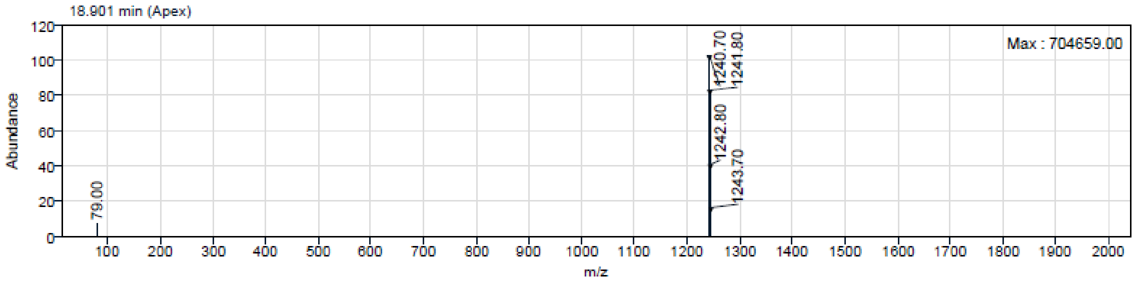

HRMS (nanochip-ESI/LTQ-Orbitrap) m/z: $[M + H]^+$ Calcd for $C_{82}H_{66}N_9O_4^+$ 1240.5232; Found 1240.5229.
MS/MS fragmentation of **3pa**

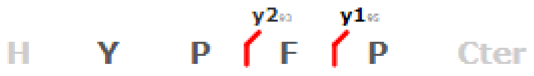

Cter = C54H32N5O-1

Sequence	Type	MF	MF Mass	m/z	Intensity	Similarity
P	y1	C59H41N6(+1)	833.339	833.34	100.33	95.11%
FP	y2	C68H50N7O(+1)	980.408	980.41	55.45	92.99%

AcKAFLPEAFLP 1q

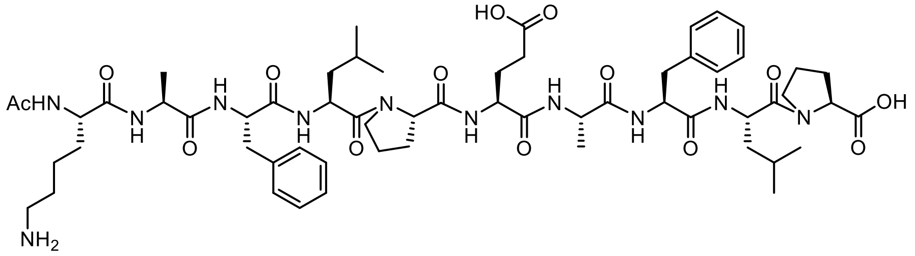

HPLC-UV chromatogram (210 nm) of AcKAFLPEAFLP (**1q**) by **Method 1**:

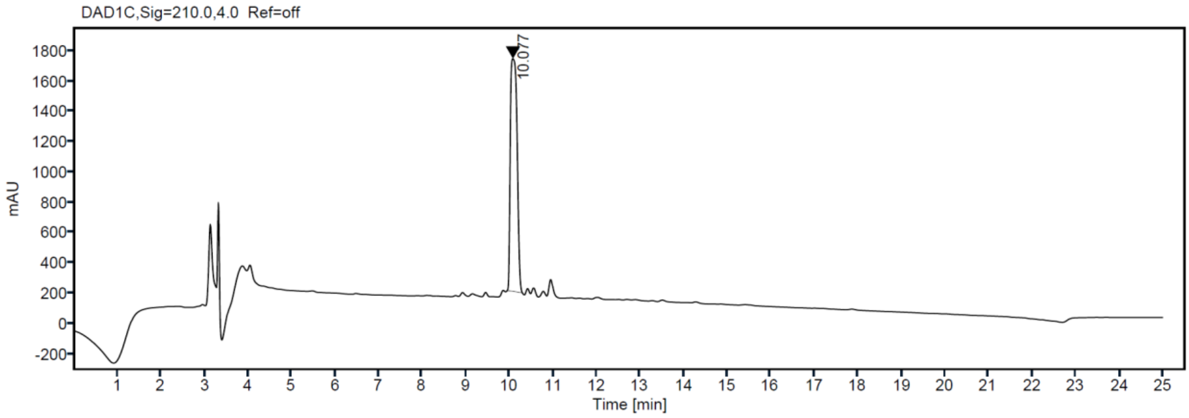

Retention time: 10.192 min **Area Percent:** 100%

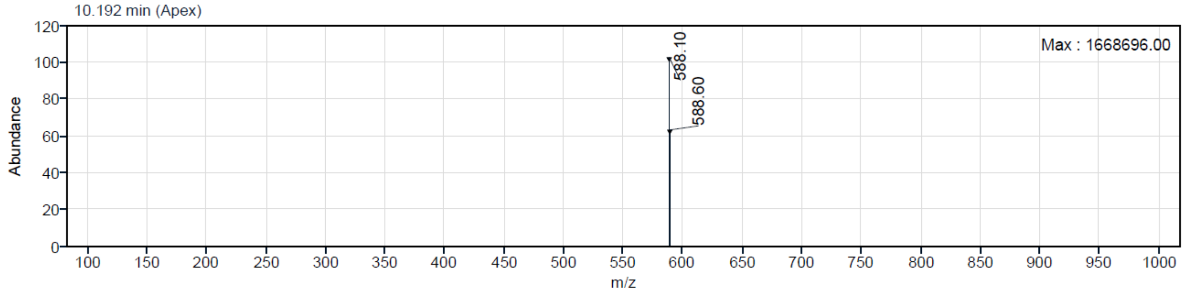

HRMS (ESI/QTOF) m/z: $[M + H]^+$ Calcd for $C_{59}H_{88}N_{11}O_{14}^+$ 1174.6507; Found 1174.6472

AcKAFLPEALFP 4CzBN 3qa

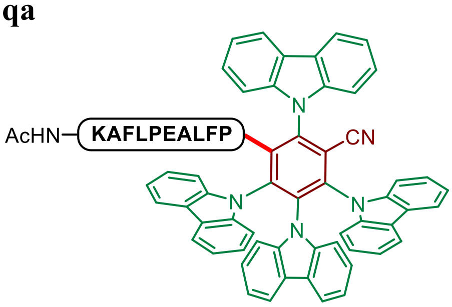

Following the general procedure (reaction time: overnight), the reaction was conducted on a7.1 μmol scale. The desired product **3qa** (3.8 mg for major isomer, 1.8 mg for minor isomer, 5.6 mg in total, 3.0 μmol, 42% yield) was isolated by **Method 2.**

HPLC-UV chromatogram (210 nm) of the crude by **Method 1**:

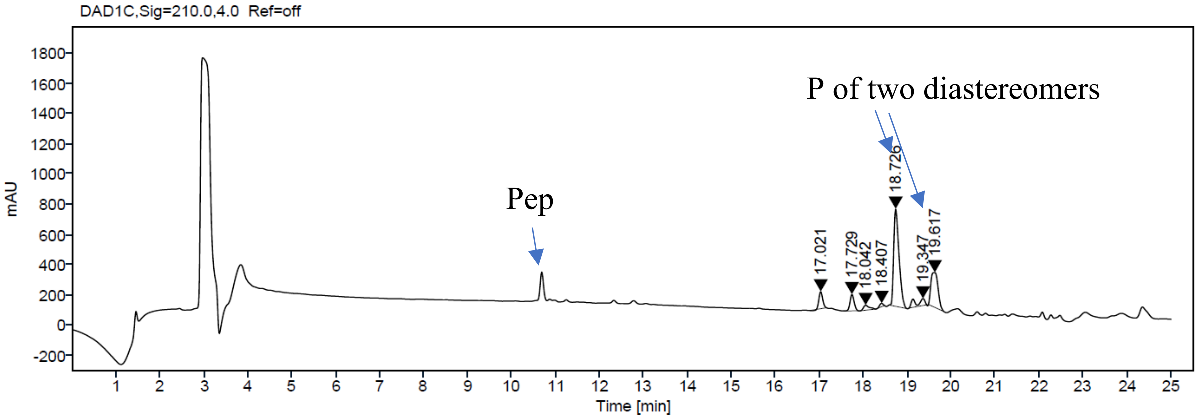

HPLC-UV chromatogram (210 nm) of **3qa** by **Method 1**:

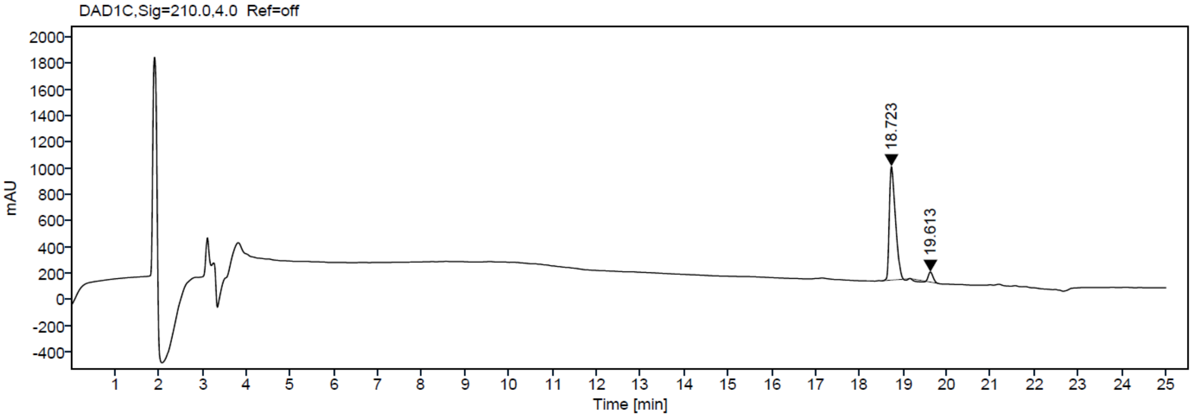

HPLC-UV chromatogram (210 nm) of **3qa’** by **Method 1**:

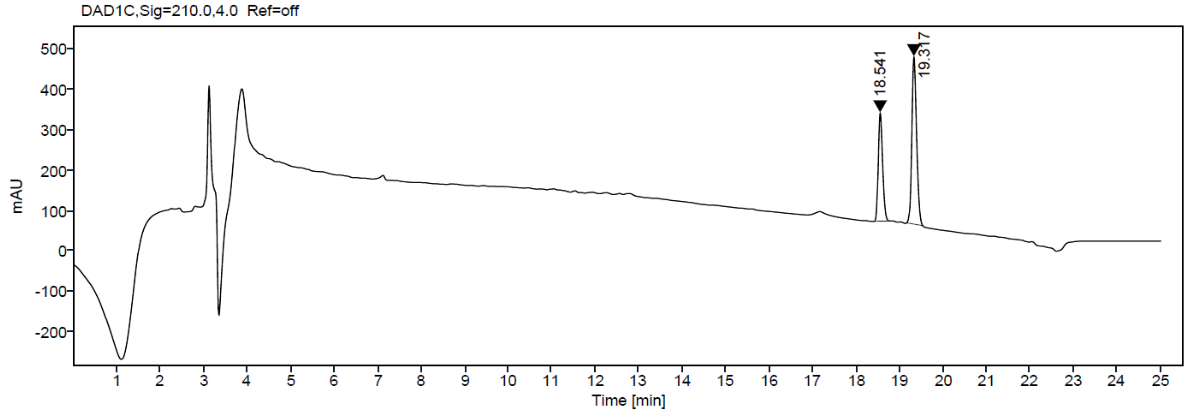

Retention time: 18.611 min **Area Percent:** 35%

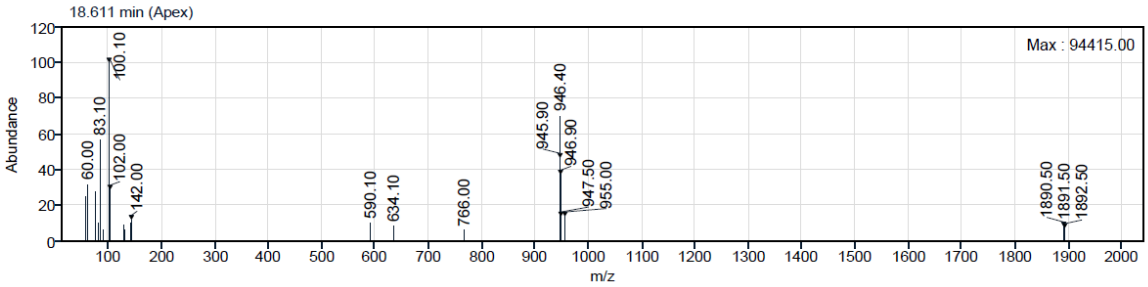

Retention time: 19.301 min **Area Percent:** 65%

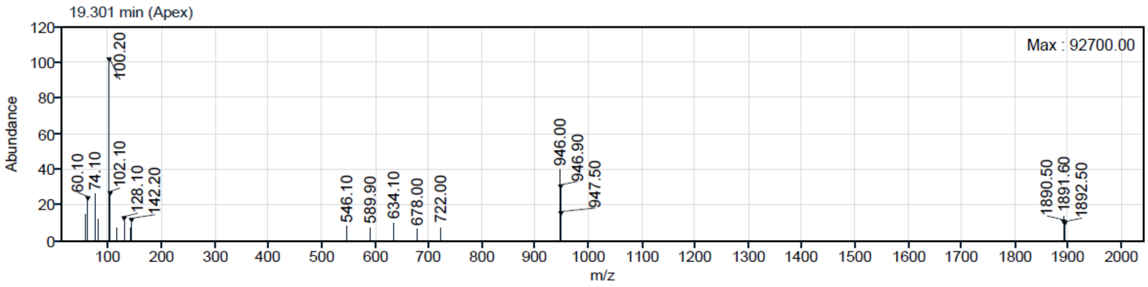

HRMS (nanochip-ESI/LTQ-Orbitrap) m/z: $[M + H]^+$ Calcd for $C_{113}H_{119}N_{16}O_{12}^+$ 1891.9188; Found 1891.9154.

MS/MS fragmentation of **3qa**

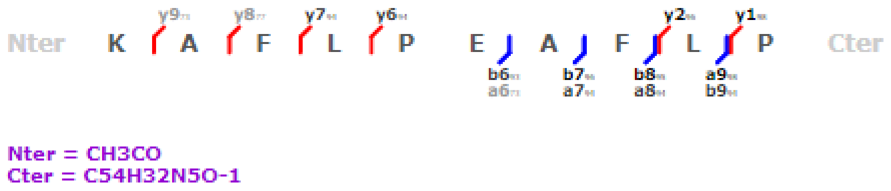

Sequence	Type	MF	MF Mass	m/z	Intensity	Similarity
P	y1	C59H41N6(+1)	833.3393	833.3387	70.16	98.07%
KAFLPEAFL	a9	C53H79N10O11(+1)	1031.593	1031.592	13.89	97.68%
KAFLPEA	b7	C39H59N8O10(+1)	799.4354	799.4349	19.93	95.81%
LP	y2	C65H52N7O(+1)	946.4233	946.4228	9.13	95.75%
KAFLPEAF	b8	C48H68N9O11(+1)	946.5038	946.5033	55.49	95.49%
KAFLPEA	a7	C38H59N8O9(+1)	771.4405	771.44	3.28	94.22%
KAFLPEAFL	b9	C54H79N10O12(+1)	1059.5879	1059.587	103.18	94.18%
PEAFLP	y6	C87H80N11O7(+1)	1390.6242	1390.624	97.36	94.18%
KAFLPEAF	a8	C47H68N9O10(+1)	918.5089	918.5084	8.17	94.11%
LPEAFLP	y7	C93H91N12O8(+1)	1503.7083	1503.708	8.41	93.79%
KAFLPE	b6	C36H54N7O9(+1)	728.3983	728.3978	15.81	93.29%
FLPEAFLP	y8	C102H100N13O9(+1)	1650.7767	1650.776	1.54	76.92%
KAFLPE	a6	C35H54N7O8(+1)	700.4034	700.4028	1.58	73.00%
AFLPEAFLP	y9	C105H105N14O10(+1)	1721.8138	1721.813	2.63	72.61%

C(RGDfE)P 1r

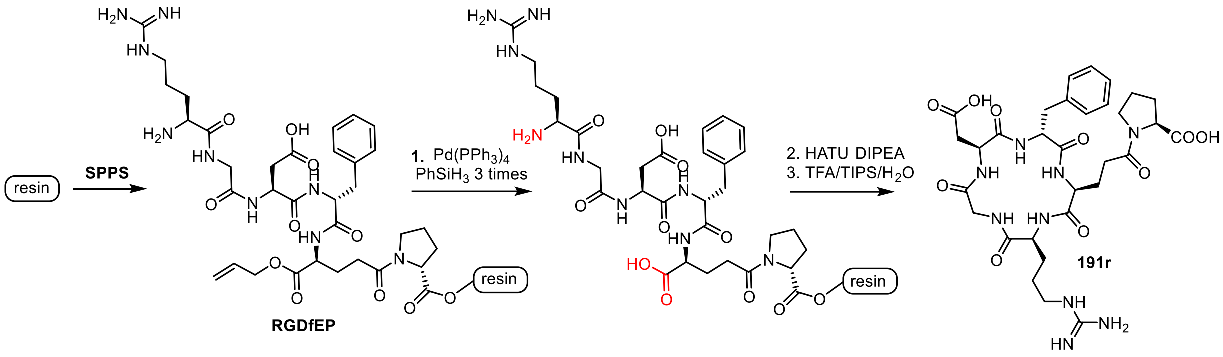

The cyclic peptide **1r** was prepared on 50 μmol scale following a reported procedure[7]: The linear peptide RGDfEP was prepared with general SPPS procedure with Rink amide resin (0.23 g/mmol). The resin with allyl protecting group was mixed with $Pd(PPh_3)_4$ (0.1 equiv.) and $PhSiH_3$ (20 equiv.) in DCM under N_2 atmosphere and shaking for 1 hour. This step was repeated twice. The on-resin cyclization was conducted by treating the resin with HATU (4 equiv.) and DIPEA (4 equiv.) in DMF (4 mL/0.1 mmol) for 3 hours. The resin was cleaved with standard cleavage condition and purified by RP-HPLC, yielding cyclic peptide **1r**.

HPLC-UV chromatogram (210 nm) of C(RGDfE)P (**1r**) by **Method 1**:

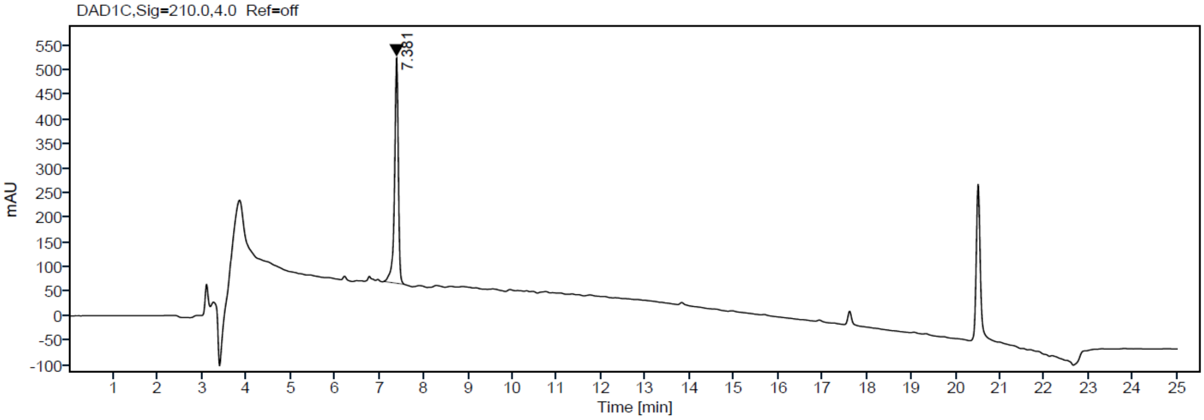

Retention time: 7.416 min **Area Percent:** 100%

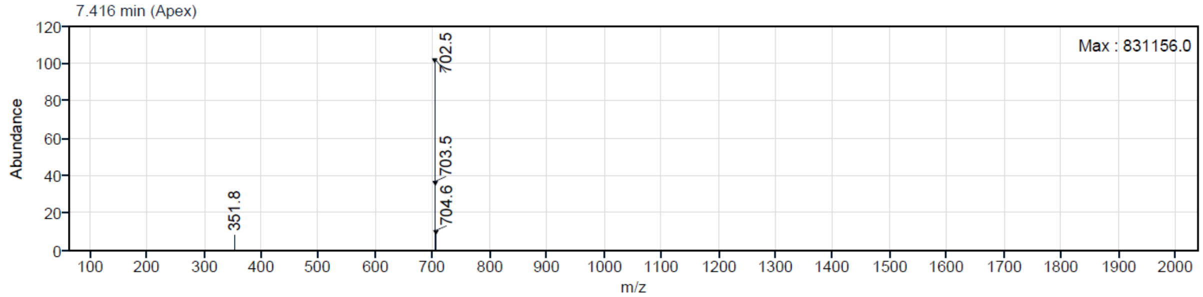

HRMS (ESI/QTOF) m/z: $[M + H]^+$ Calcd for $C_{31}H_{44}N_9O_{10}^+$ 702.3206; Found 702.3221.

C(RGDfE)P 4CzBN 3ra

[7] Wu, Y.; Chau, H.-F.; Yeung, Y.-H.; Thor, W.; Kai, H.-Y.; Chan, W.-L.; Wong, K.-L., *Angew. Chem., Int. Ed.* **2022,** *61* (34), e202207532.

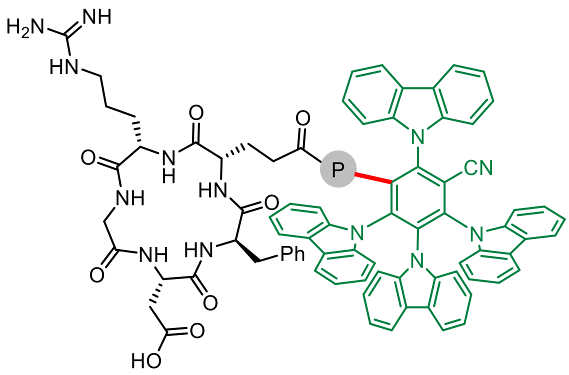

Following the general procedure, the reaction was conducted on a2.9 μmol scale. The desired product **3ra** (1.2 mg, 0.83 μmol, 43% yield) was isolated by **Method 1**.

HPLC-UV chromatogram (210 nm) of the crude by **Method 1**:

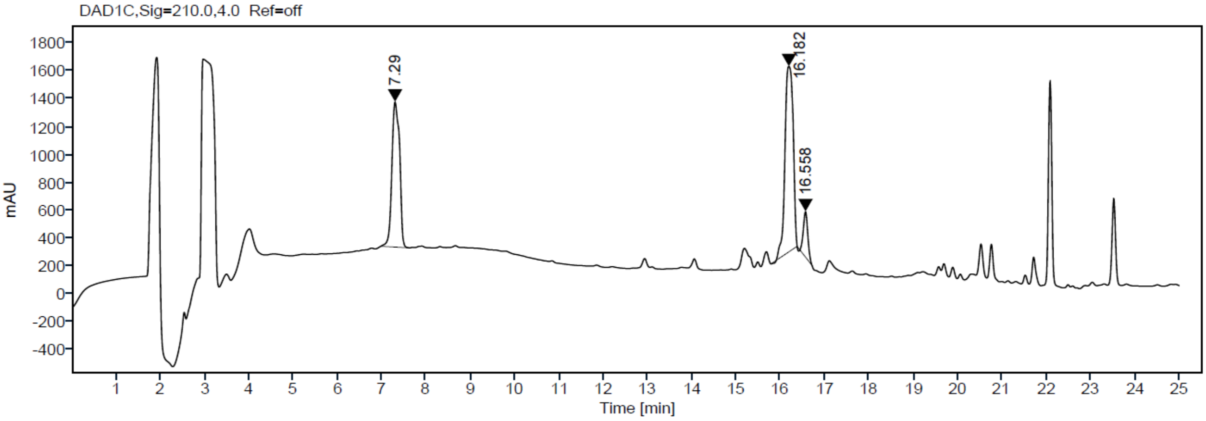

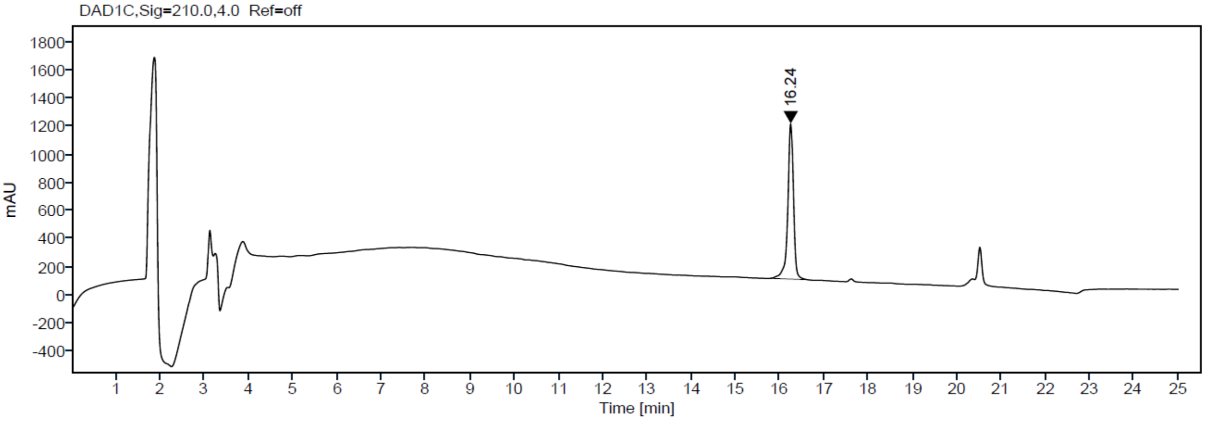

Retention time: 16.197 min **Area Percent:** 100%

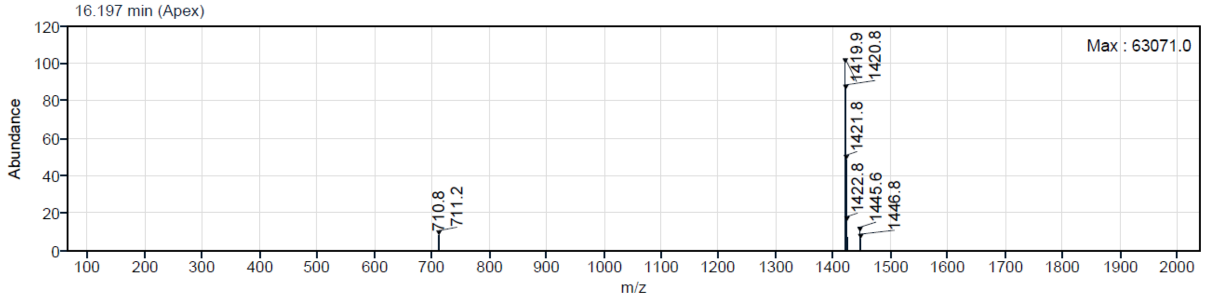

HRMS (Nanochip-based ESI/LTQ-Orbitrap) m/z: $[M + H]^+$ Calcd for $C_{85}H_{75}N_{14}O_8^+$ 1419.5887; Found 1419.5896.

SFLLRNP 1s

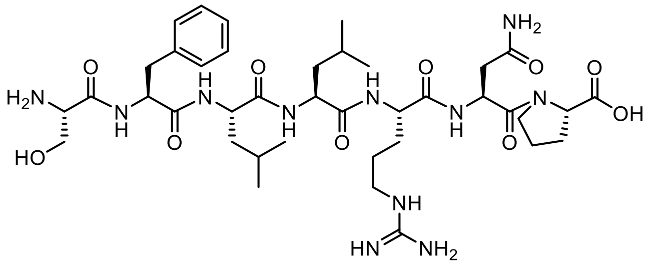

HPLC-UV chromatogram (210 nm) of TRAP-7 peptide SFLLRNP (**1s**) by **Method 1**:

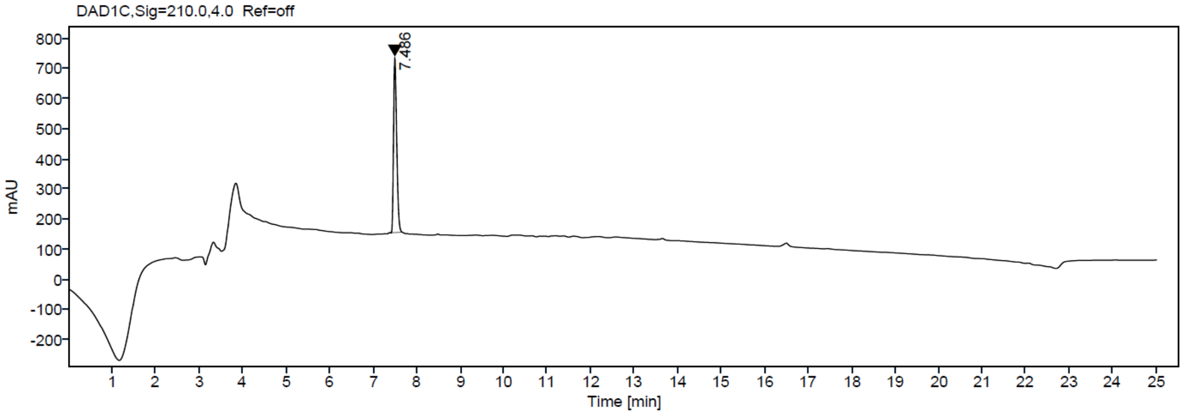

Retention time: 7.522 min **Area Percent:** 100%

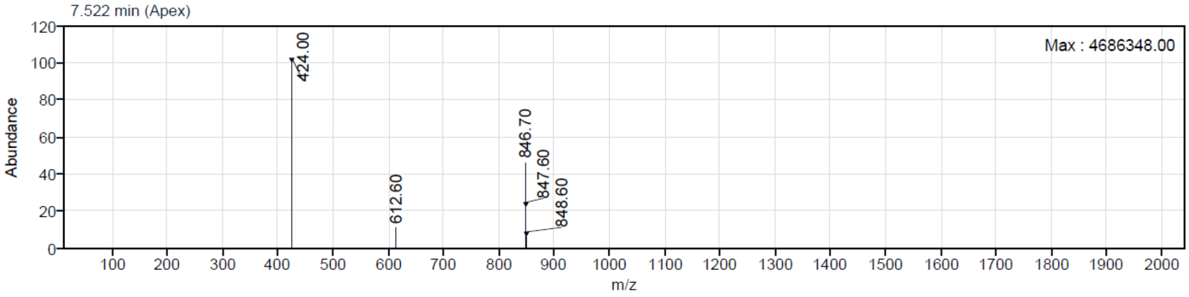

HRMS (nanochip-ESI/LTQ-Orbitrap) m/z: $[M + H_2]^{+2}$ Calcd for $C_{39}H_{65}N_{11}O_{10}{}^{+2}$ 423.7452; Found 423.7463.

VHFFKNIVTPRTP 1t

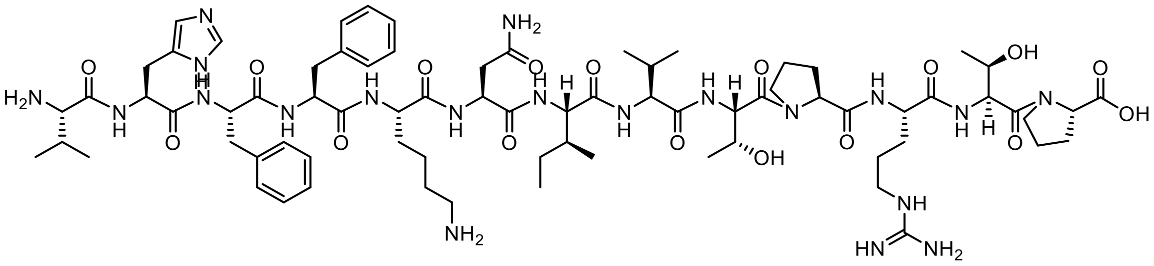

HPLC-UV chromatogram (210 nm) of **Myelin basic (87-99) (1t)** by **Method 1**:

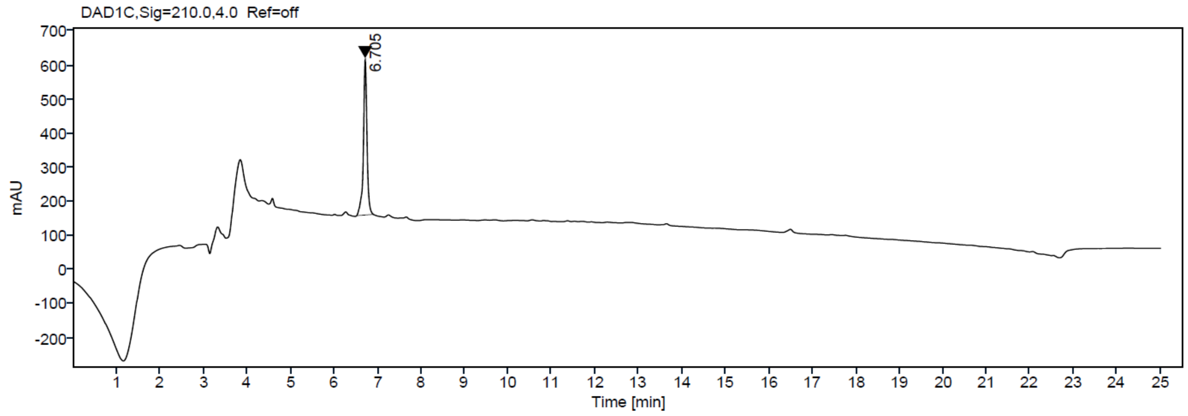

Retention time: 6.734 min **Area Percent:** 100%

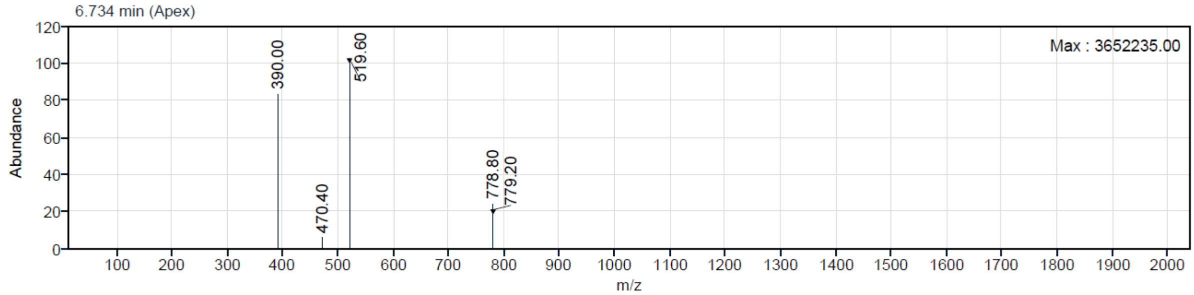

HRMS (nanochip-ESI/LTQ-Orbitrap) m/z: $[M + H_3]^{+3}$ Calcd for $C_{74}H_{117}N_{20}O_{17}^{+3}$ 519.2963; Found 519.2961.

VHFFKNIVTPRTP 4CzBN 3sa

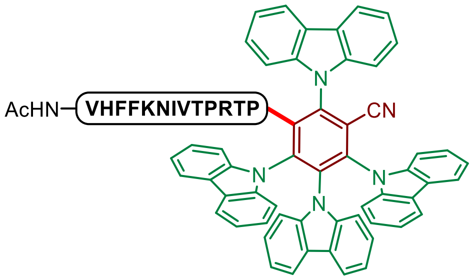

Following the general procedure (reaction time: overnight), the reaction was conducted on a10 μmol scale. The desired product **3sa** (5.1mg, 4.5 μmol, 45% yield) was isolated by **Method 1.**

HPLC-UV chromatogram (210 nm) of the crude by **Method 1**:

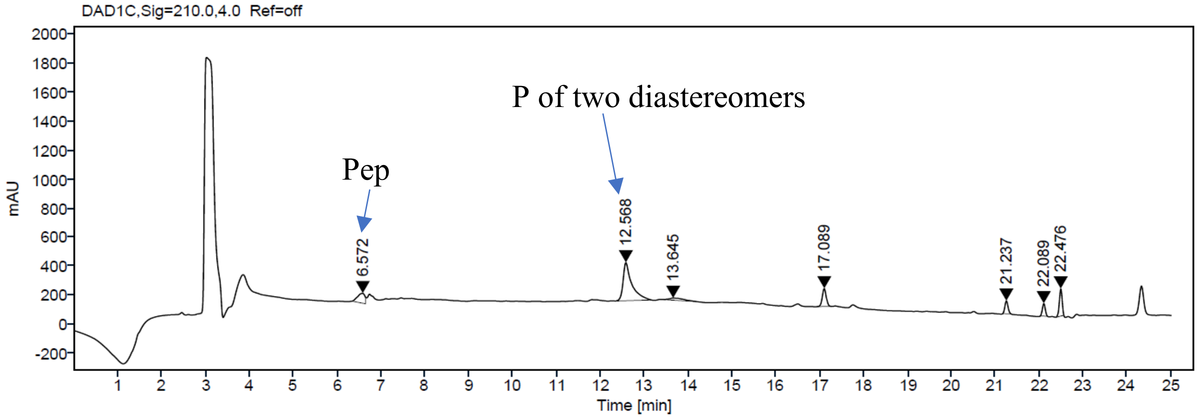

HPLC-UV chromatogram (210 nm) of **3sa** by **Method 1**:

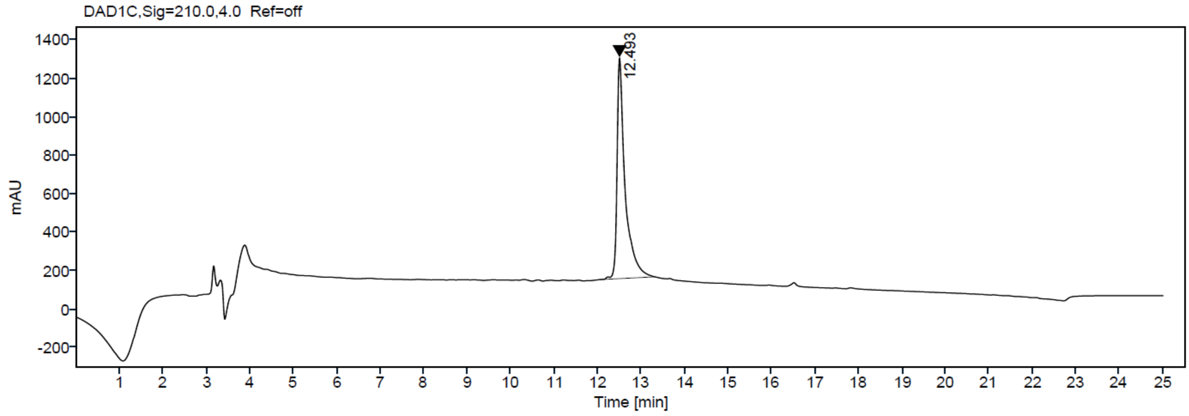

Retention time: 12.519 min **Area Percent:** 100%

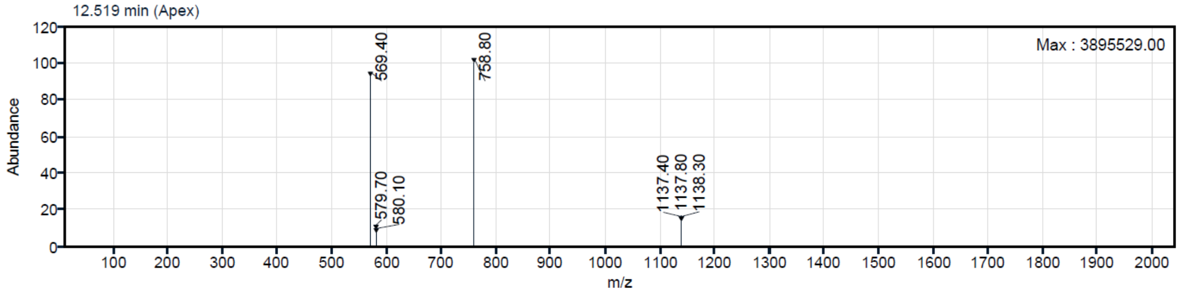

HRMS (nanochip-ESI/LTQ-Orbitrap) m/z: $[M + H_2]^{+2}$ Calcd for $C_{128}H_{147}N_{25}O_{15}^{+2}$ 1137.0749; Found 1137.0790.

MS/MS fragmentation of **3sa**:

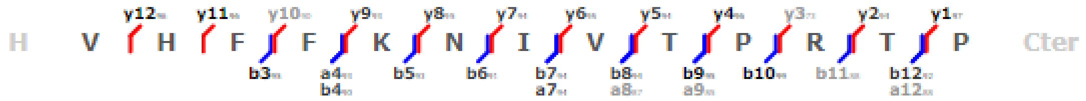

Cter = C54H32N5O-1

Sequence	Type	MF	MF Mass	m/z	Intensity	Similarity
VHFFKNIVTP	b10	C59H87N14O12(+1)	1183.663	1183.662	9.08	98.90%
P	y1	C59H41N6(+1)	833.3393	833.3387	26.38	96.55%
HFFKNIVTPRTP	y12	C123H137N24O14(+1)	2174.075	1087.541	29.09	95.82%
FFKNIVTPRTP	y11	C117H130N21O13(+1)	2037.016	2037.015	43.79	95.70%
PRTP	y4	C74H67N12O4(+1)	1187.541	1187.54	86.71	95.54%
FKNIVTPRTP	y10	C108H121N20O12(+1)	1889.947	1889.947	35.86	95.50%
KNIVTPRTP	y9	C99H112N19O11(+1)	1742.879	1742.878	25.24	95.50%
FFKNIVTPRTP	y11	C117H130N21O13(+1)	2037.016	1019.011	31.93	95.48%
VHFFKNIVT	b9	C54H80N13O11(+1)	1086.61	1086.61	48.78	95.34%
VHF	b3	C20H26N5O3(+1)	384.2036	384.203	27.55	95.21%
NIVTPRTP	y8	C93H100N17O10(+1)	1614.784	1614.783	66.01	94.67%
VTPRTP	y6	C83H83N14O7(+1)	1387.657	1387.656	56.21	94.66%
VHFFKNI	b7	C45H64N11O8(+1)	886.4939	886.4934	30.06	94.50%
VHFFKNIV	b8	C50H73N12O9(+1)	985.5623	985.5618	49.83	94.43%

VHFFKNI	a7	C44H64N11O7(+1)	858.499	858.4985	3.95	94.37%
TPRTP	y5	C78H74N13O6(+1)	1288.589	1288.588	77.03	94.09%
IVTPRTP	y7	C89H94N15O8(+1)	1500.741	1500.74	28.8	93.96%
TP	y2	C63H48N7O2(+1)	934.3869	934.3864	6.74	93.56%
VHFFKNIVTPRT	b12	C69H106N19O15(+1)	1440.812	1440.811	6.17	93.14%
VHFF	a4	C28H35N6O3(+1)	503.2771	503.2765	3.65	93.01%
VHFFK	b5	C35H47N8O5(+1)	659.3669	659.3664	7.56	92.98%
VHFFKNIVTPRT	b12	C69H106N19O15(+1)	1440.812	720.9092	0.53	91.13%
VHFFKN	b6	C39H53N10O7(+1)	773.4099	773.4093	4.25	91.08%
KNIVTPRTP	y9	C99H112N19O11(+1)	1742.879	871.9428	3.35	91.00%
VHFF	b4	C29H35N6O4(+1)	531.272	531.2714	10.82	90.17%
VHFFKNIVTPRT	a12	C68H106N19O14(+1)	1412.817	1412.816	0.73	88.09%
VHFFKNIVTPR	b11	C65H99N18O13(+1)	1339.764	670.3853	1.02	87.69%
VHFFKNIVTPR	b11	C65H99N18O13(+1)	1339.764	1339.763	4.69	87.62%
VHFFKNIV	a8	C49H73N12O8(+1)	957.5674	957.5669	2.46	87.15%
VHFFKNIVT	a9	C53H80N13O10(+1)	1058.615	1058.615	1.74	85.02%
FKNIVTPRTP	y10	C108H121N20O12(+1)	1889.947	945.477	1.66	84.13%
RTP	y3	C69H60N11O3(+1)	1090.488	1090.488	2.44	73.22%

SFLLRNP 4CzBN 3ta

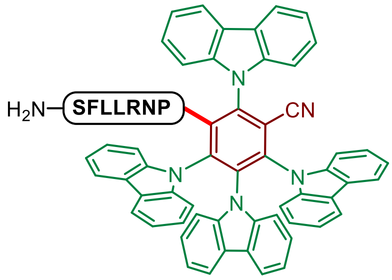

Following the general procedure (reaction time: overnight), the reaction was conducted on a5.3 μmol scale. The desired product **3ta** (3.9 mg, 2.5 μmol, 47% yield) was isolated by **Method 1**

HPLC-UV chromatogram (210 nm) of the crude by **Method 1**:

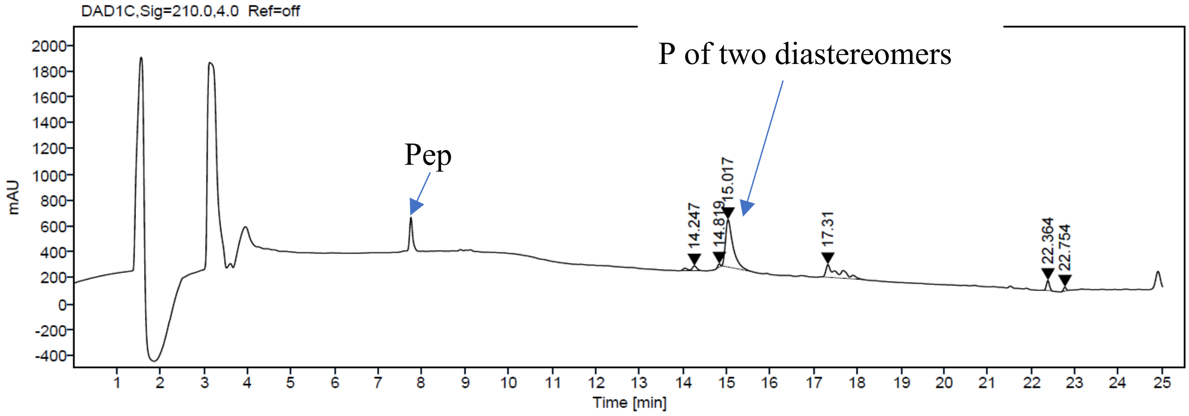

HPLC-UV chromatogram (210 nm) of **3ta** by **Method 1**:

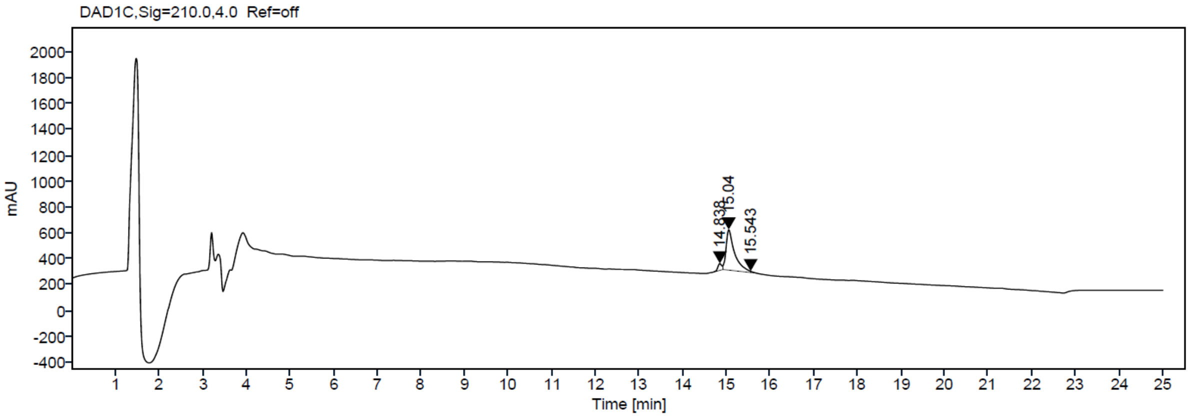

Retention time: 15.047 min **Area Percent:** 100%

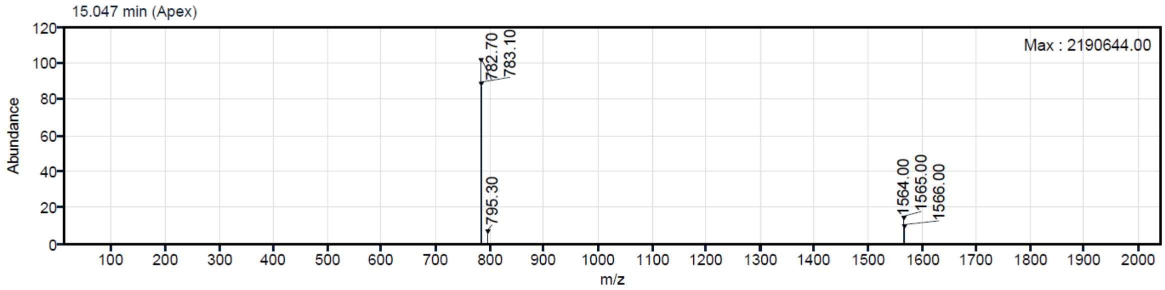

HRMS (nanochip-ESI/LTQ-Orbitrap) m/z: $[M + H_2]^{+2}$ Calcd for $C_{93}H_{96}N_{16}O_8^{+2}$ 782.3793; Found 782.3773.

MS/MS fragmentation of **3sa**

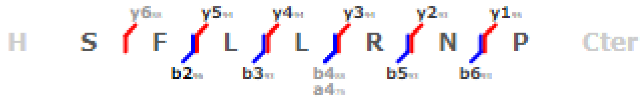

Cter = C54H32N5O-1

Sequence	Type	MF	MF Mass	m/z	Intensity	Similarity
SF	b2	C12H15N2O3(+1)	235.1083	235.1077	5.87	95.97%
P	y1	C59H41N6(+1)	833.3393	833.3387	40.9	94.71%
LLRNP	y5	C81H81N14O5(+1)	1329.651	1329.651	100.2	93.92%
LLRNP	y5	C81H81N14O5(+1)	1329.651	665.3291	57.63	93.87%
LRNP	y4	C75H70N13O4(+1)	1216.567	1216.567	35.91	93.58%
RNP	y3	C69H59N12O3(+1)	1103.483	1103.483	30.71	93.57%
NP	y2	C63H47N8O2(+1)	947.3822	947.3816	21.93	93.38%
SFLLRN	b6	C34H55N10O8(+1)	731.4204	731.4199	34.66	93.01%
SFLLR	b5	C30H49N8O6(+1)	617.3775	617.377	52.91	92.76%
SFL	b3	C18H26N3O4(+1)	348.1923	348.1918	13.05	92.68%
FLLRNP	y6	C90H90N15O6(+1)	1476.72	738.8633	4.15	90.85%
SFLL	b4	C24H37N4O5(+1)	461.2764	461.2758	3.25	88.15%
FLLRNP	y6	C90H90N15O6(+1)	1476.72	1476.719	0.59	84.82%
SFLL	a4	C23H37N4O4(+1)	433.2815	433.2809	0.98	75.91%

FRGDSPASSKP 1u

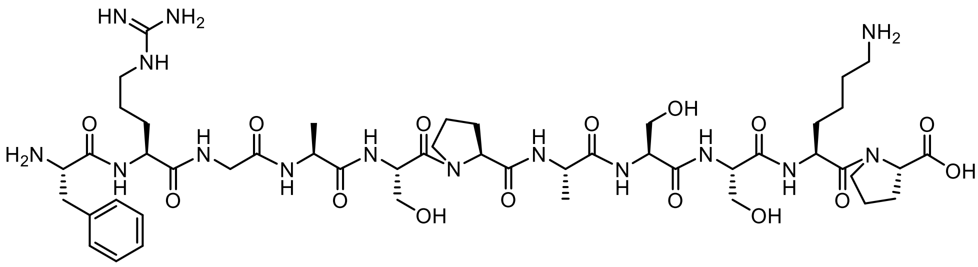

HPLC-UV chromatogram (210 nm) of Fibronectin binding inhibitor peptide (**191u**) by **Method 1**:

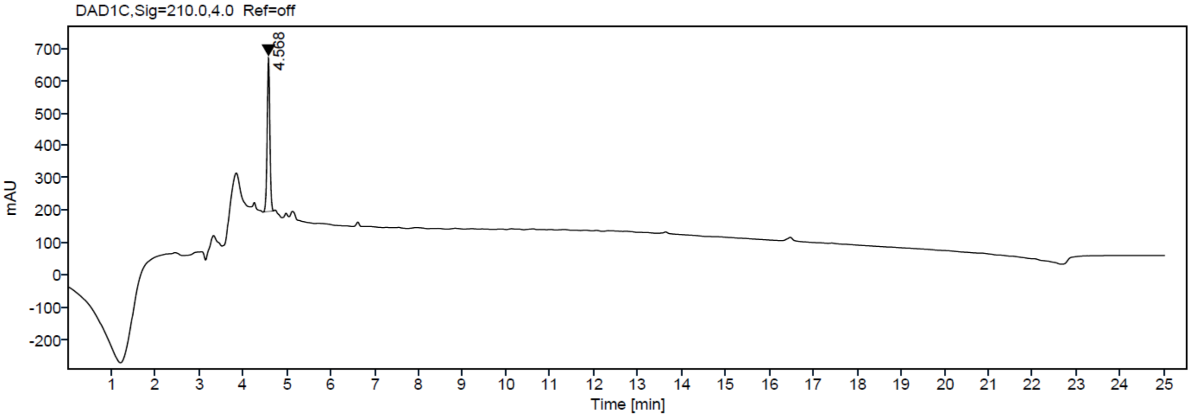

Retention time: 4.587 min **Area Percent:** 43%

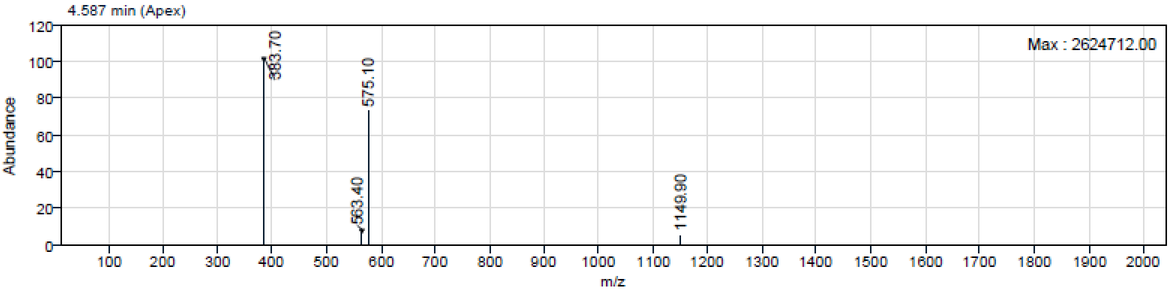

HRMS (nanochip-ESI/LTQ-Orbitrap) m/z: $[M + H_2]^{+2}$ Calcd for $C_{49}H_{79}N_{15}O_{17}{}^{+2}$ 574.7884; Found 574.7877.

FRGDSPASSKP 4CzBN 3ua

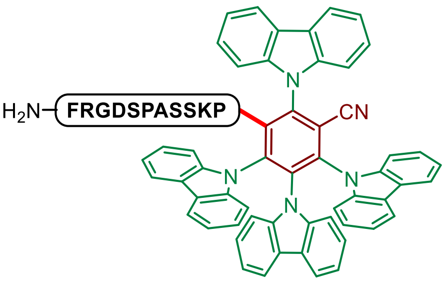

Following the general procedure (reaction time: overnight), the reaction was conducted on a8.1 μmol scale. The desired product **3ua** (5.7 mg, 3.6 μmol, 52% yield) was isolated by **Method 1**
HPLC-UV chromatogram (210 nm) of the crude by **Method 1**:

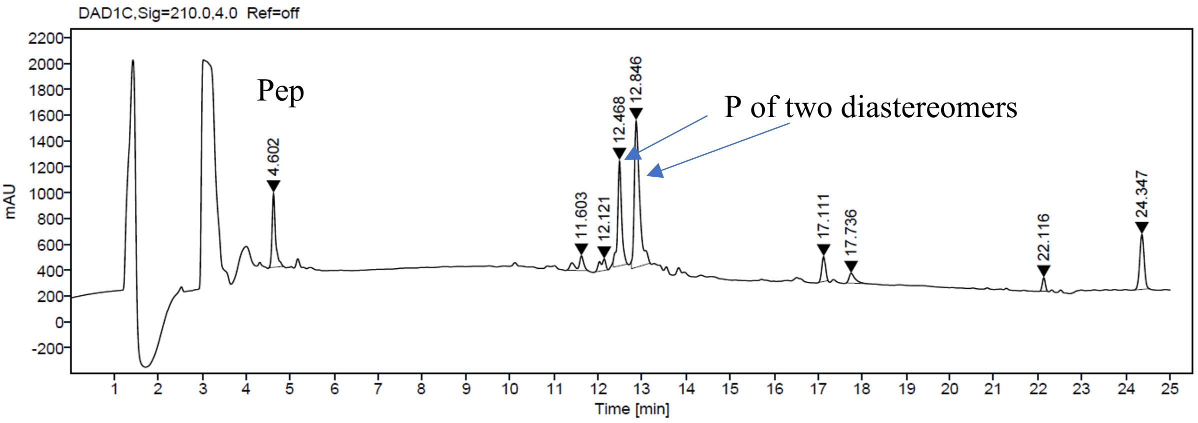

HPLC-UV chromatogram (210 nm) of **3ua** by **Method 1**:

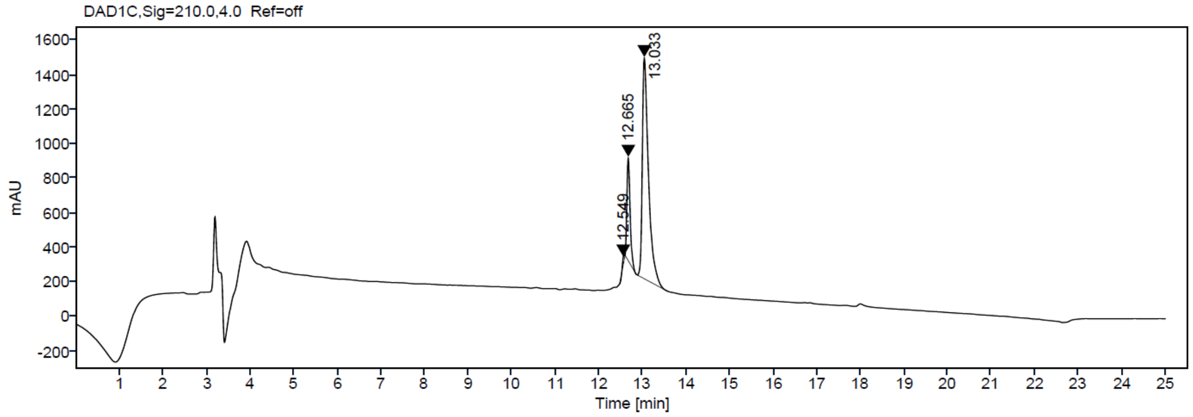

Retention time: 13.046 min **Area Percent:** 100%

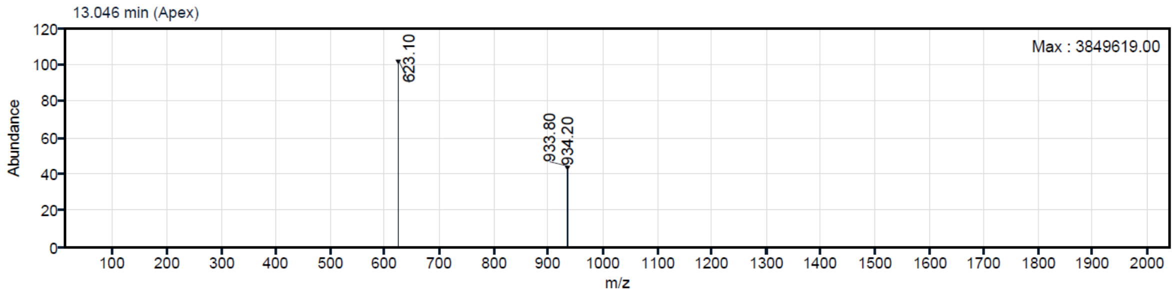

HRMS (nanochip-ESI/LTQ-Orbitrap) m/z: $[M + H_2]^{+2}$ Calcd for $C_{103}H_{110}N_{20}O_{15}{}^{+2}$ 933.4224; Found 933.4221.

MS/MS fragmentation of **3ua**:

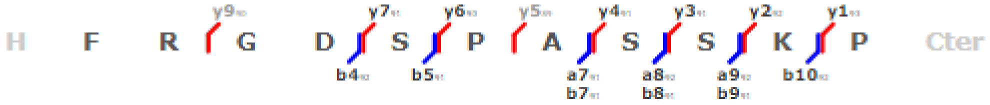

Cter = C54H32N5O-1

Sequence	Type	MF	MF Mass	m/z	Intensity	Similarity
P	y1	C59H41N6(+1)	833.3393	833.3387	100.43	92.58%
KP	y2	C65H53N8O(+1)	961.4342	961.4337	10.82	92.48%

FRGD	b4	C21H30N7O6(+1)	476.2258	476.2252	14.01	92.28%
FRGDSPASS	a9	C37H57N12O13(+1)	877.4168	877.4163	2.2	92.07%
FRGDSPAS	a8	C34H52N11O11(+1)	790.3848	790.3842	0.96	91.95%
FRGDSPASSK	b10	C44H69N14O15(+1)	1033.507	1033.506	80.03	91.55%
FRGDSPA	a7	C31H47N10O9(+1)	703.3527	703.3522	2.44	91.42%
FRGDSPA	b7	C32H47N10O10(+1)	731.3477	731.3471	8.85	90.89%
SSKP	y4	C71H63N10O5(+1)	1135.498	1135.498	3.73	90.85%
SPASSKP	y7	C82H80N13O9(+1)	1390.62	1390.62	8.04	90.84%
SKP	y3	C68H58N9O3(+1)	1048.466	1048.466	2.78	90.82%
FRGDSPAS	b8	C35H52N11O12(+1)	818.3797	818.3791	8.03	90.79%
FRGDSPASS	b9	C38H57N12O14(+1)	905.4117	905.4112	15.35	90.62%
FRGDS	b5	C24H35N8O8(+1)	563.2578	563.2572	2.98	90.55%
PASSKP	y6	C79H75N12O7(+1)	1303.588	1303.588	2.51	90.06%
GDSPASSKP	y9	C88H88N15O13(+1)	1562.669	1562.668	1.2	89.65%
ASSKP	y5	C74H68N11O6(+1)	1206.535	1206.535	0.52	88.57%

RPKPQQFQFFGLMP 1v

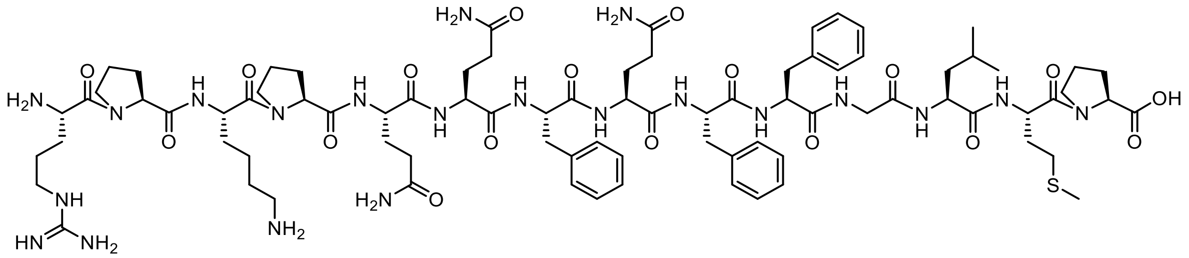

HPLC-UV chromatogram (210 nm) of RPKPQQFQFFGLMP (**1v**) by **Method 1**:

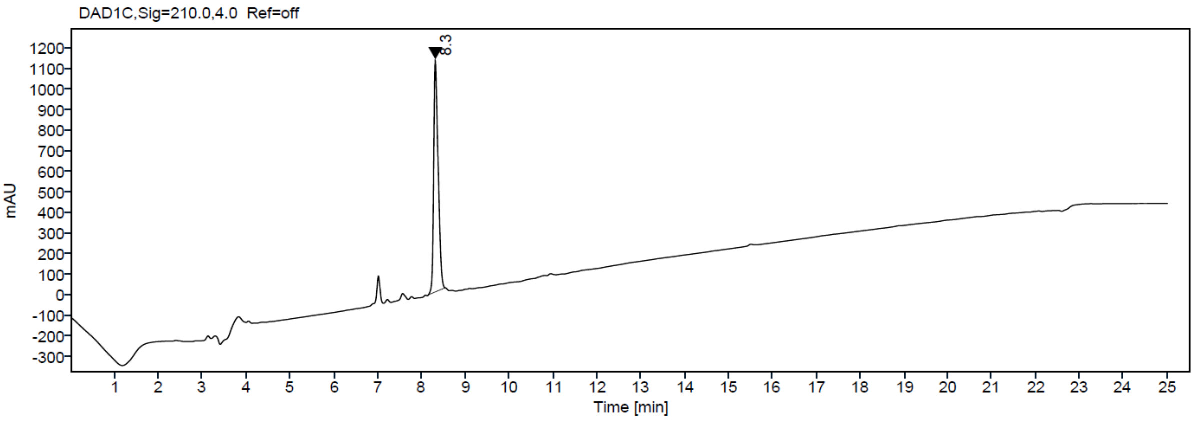

Retention time: 8.353 min **Area Percent:** 100%

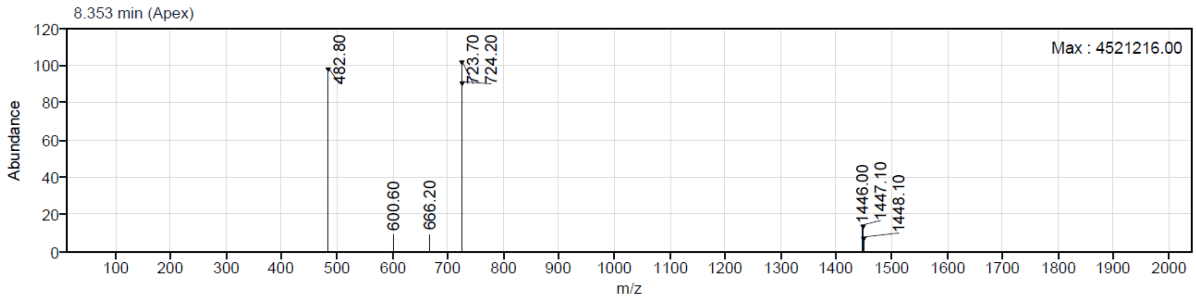

HRMS (nanochip-ESI/LTQ-Orbitrap) m/z: $[M + H_2]^{+2}$ Calcd for $C_{68}H_{106}N_{18}O_{15}S^{+2}$ 723.3897; Found 723.3892.

RPKPQQFQFFGLMP 4CzBN 3va

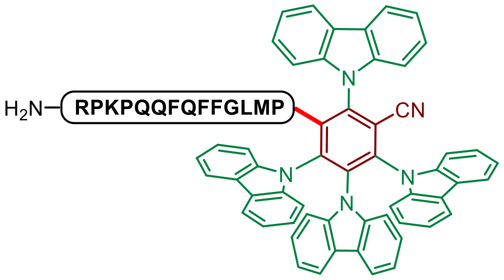

Following the general procedure (reaction time: overnight), the reaction was conducted on a 10 μmol scale. The desired product **3va** (8.4 mg, 3.9 μmol, 39% yield) was isolated by **Method 3.**

HPLC-UV chromatogram (210 nm) of the crude by **Method 1**:

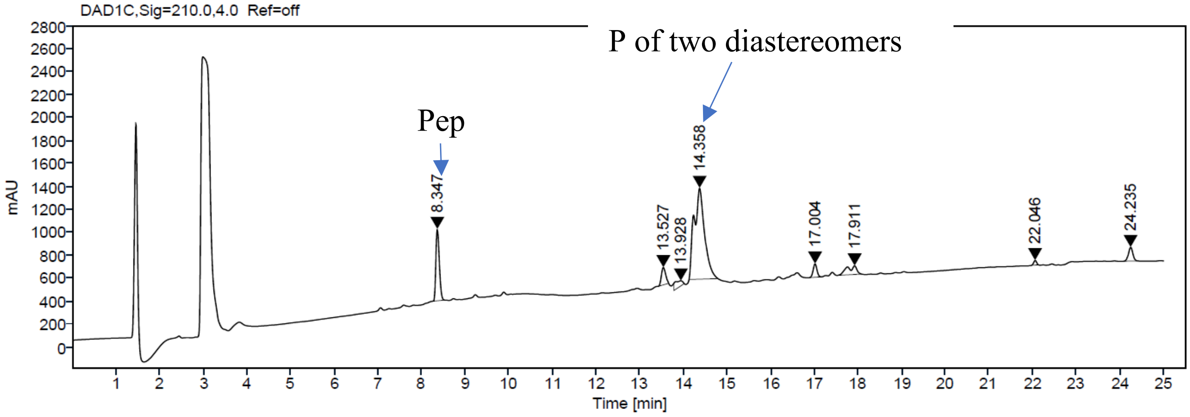

HPLC-UV chromatogram (210 nm) of **3va** by **Method 1**:

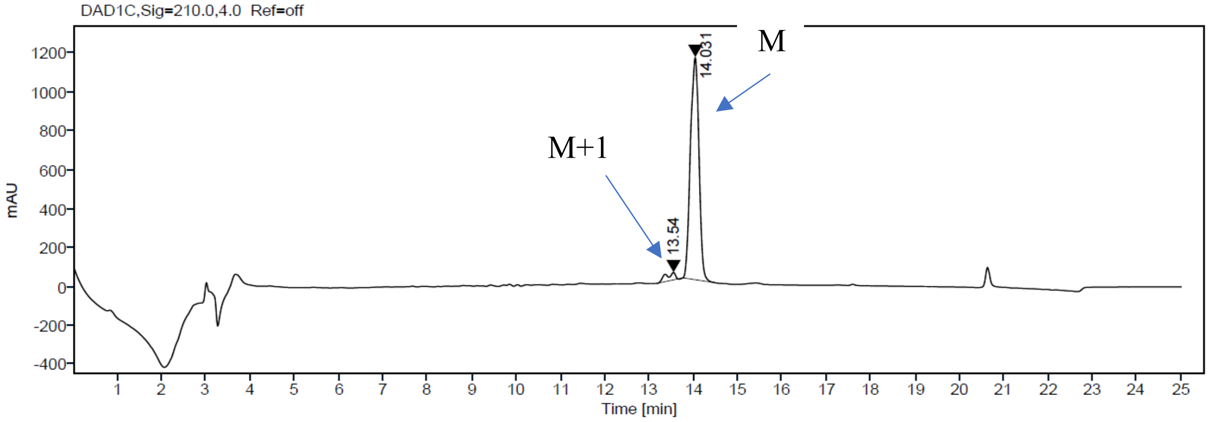

Retention time: 14.643 min **Area Percent:** 100%

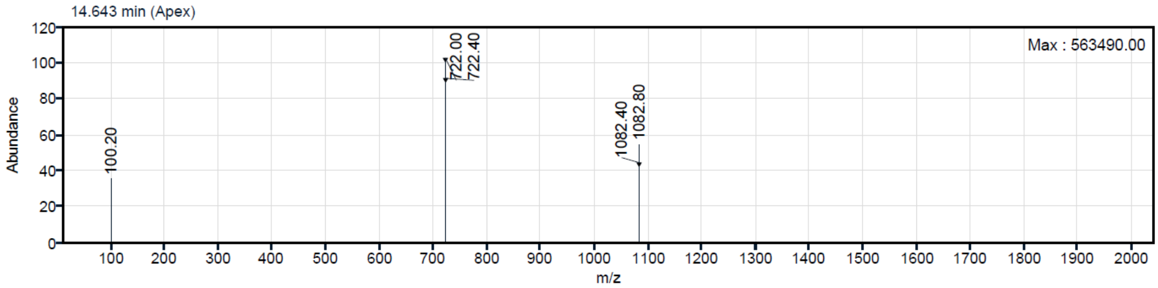

HRMS (nanochip-ESI/LTQ-Orbitrap) m/z: $[M + H_2]^{+2}$ Calcd for $C_{122}H_{137}N_{23}O_{13}S^{+2}$ 1082.0238; Found 1082.0245.

MS/MS fragmentation of **3va**:

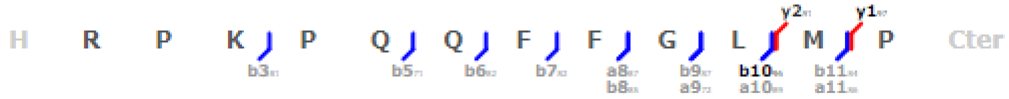

Sequence	Type	MF	MF Mass	m/z	Intensity	Similarity
P	y1	C59H41N6(+1)	833.3393	833.3387	102.31	97.05%
RPKPQQFFGL	b10	C58H87N16O12(+1)	1199.6689	1199.668	66.74	93.51%
RPKPQ	b5	C27H47N10O6(+1)	607.368	607.3675	0.82	92.70%
MP	y2	C64H50N7OS(+1)	964.3798	964.3792	53.21	92.36%
RPKPQQFFGLM	b11	C63H96N17O13S(+1)	1330.7094	1330.709	62.62	92.01%
RPKPQQFF	b8	C50H73N14O10(+1)	1029.5634	1029.563	16.39	90.80%
RPKPQQFFGL	a10	C57H87N16O11(+1)	1171.674	1171.674	5.81	89.47%
KPQQFFGLMP	y10	C111H117N18O11S(+1)	1909.887	1909.886	2.92	84.08%
RPKPQQ	b6	C32H55N12O8(+1)	735.4266	735.426	2.42	83.69%
RPKPQQF	b7	C41H64N13O9(+1)	882.495	882.4944	6.19	82.03%
RPKPQQFFGLM	b11	C63H96N17O13S(+1)	1330.7094	665.8581	5.45	81.37%
RPKPQQFFGLM	a11	C62H96N17O12S(+1)	1302.7145	1302.714	2.43	77.10%
RPKPQQFF	a8	C49H73N14O9(+1)	1001.5685	1001.568	1.13	73.12%
RPKPQQF	a7	C40H64N13O8(+1)	854.5001	854.4995	1.16	71.42%

VPALKP 1w

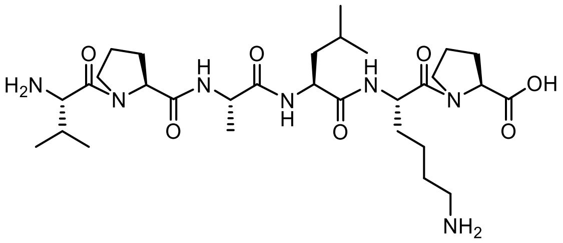

HPLC-UV chromatogram (210 nm) of VPALKP (**1w**) by **Method 1**:

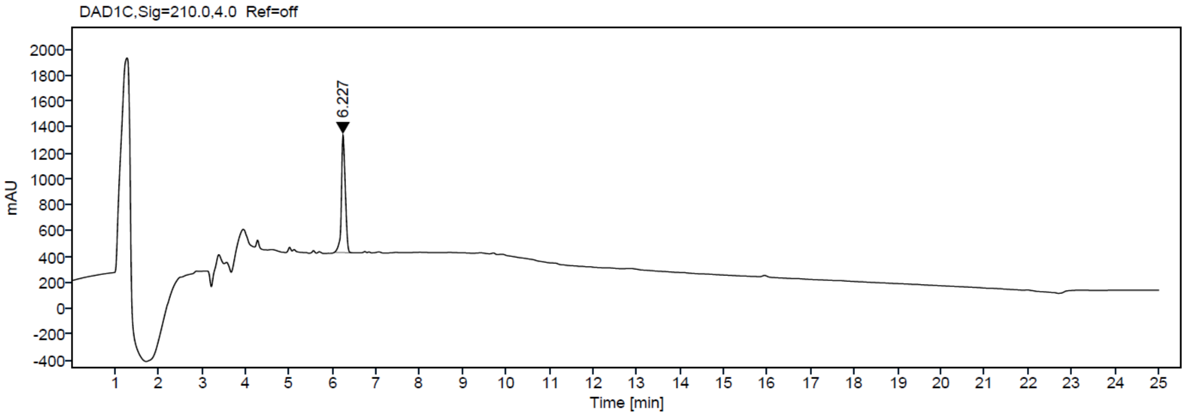

Retention time: 6.259 min **Area Percent:** 100%

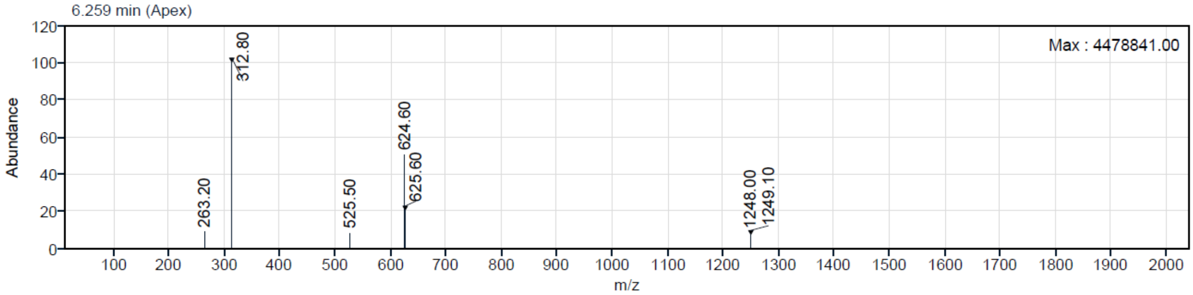

HRMS (nanochip-ESI/LTQ-Orbitrap) m/z: $[M + H]^+$ Calcd for $C_{30}H_{54}N_7O_7^+$ 624.4079; Found 624.4075.

VPALKP 4CzBN 3wa

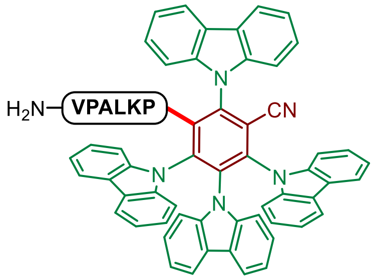

Following the general procedure, the reaction was conducted on a 10 μmol scale. The desired product **3wa** (8.6 mg, 6.4 μmol, 64% yield) was isolated by **Method 3.** More diastereomers derives from atropisomers due to the steric hindrance between 4CzBN and the terminal proline.
HPLC-UV chromatogram (210 nm) of crude by **Method 1**:

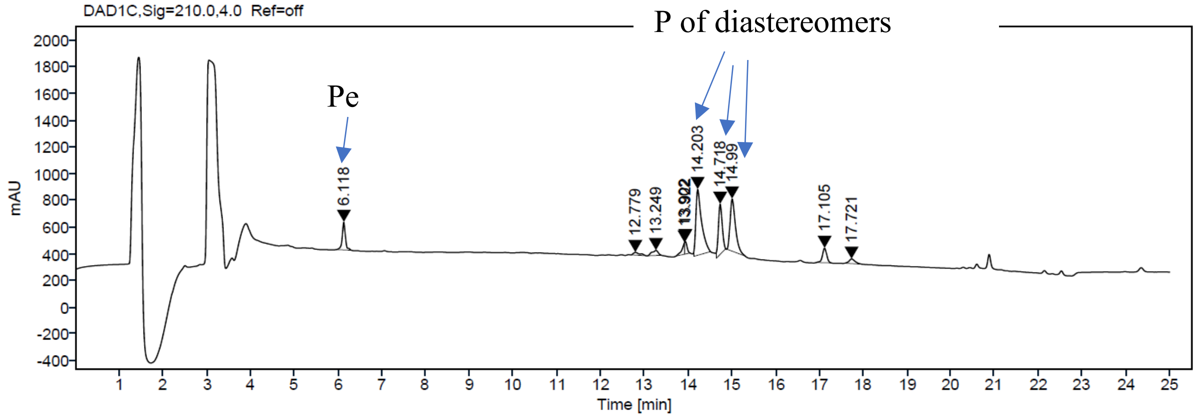

HPLC-UV chromatogram (210 nm) of product by **Method 1**:

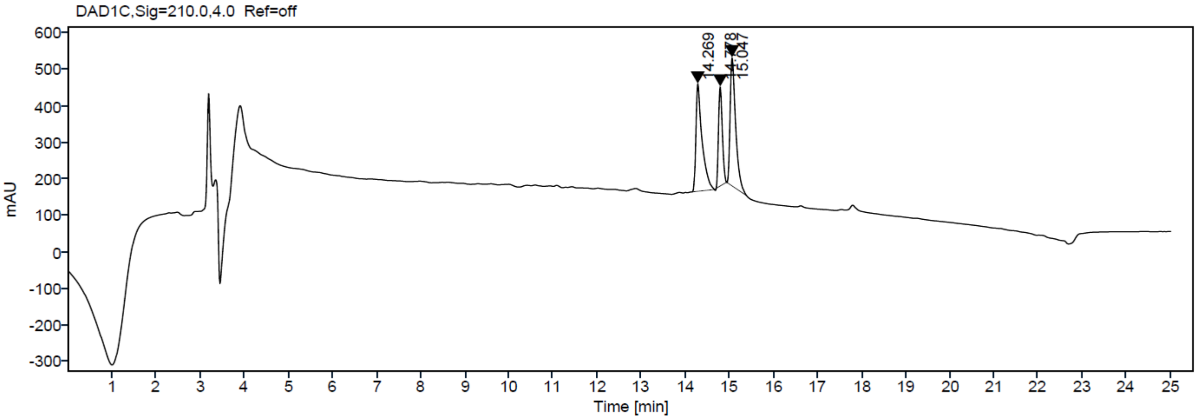

Retention time: 14.307 min **Area Percent:** 46%

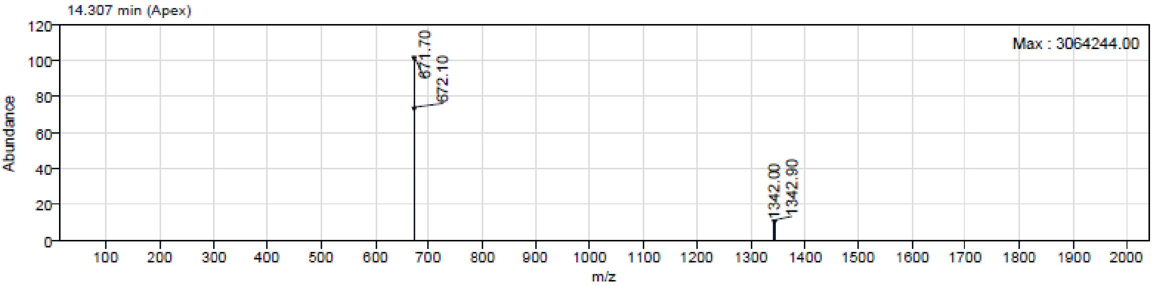

Retention time: 14.783 min **Area Percent:** 19%

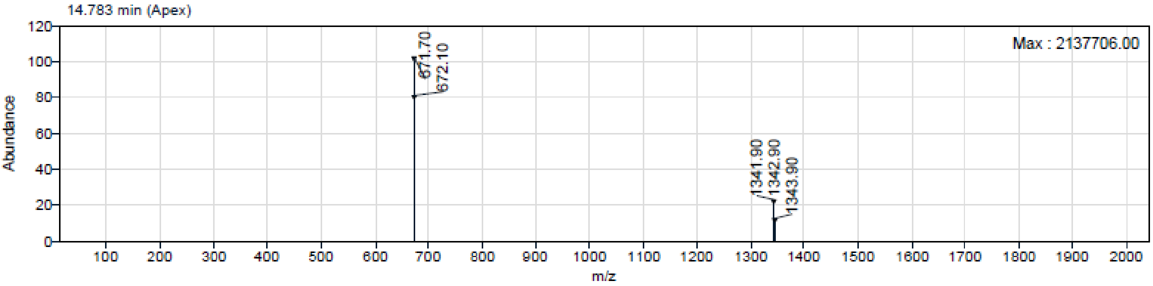

Retention time: 15.173 min **Area Percent:** 35%

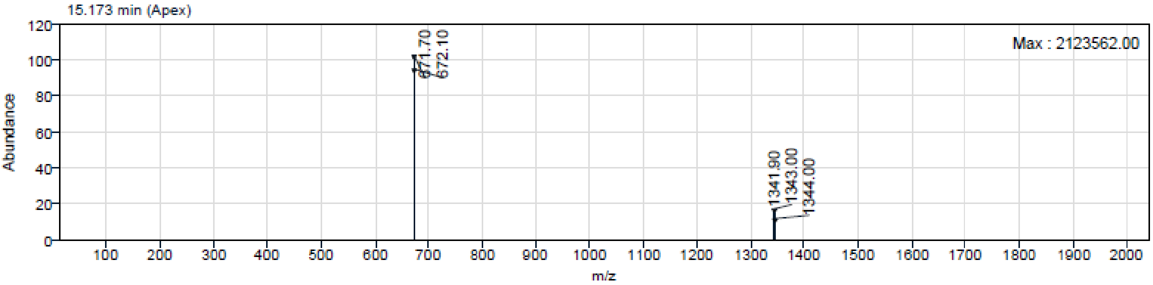

HRMS (nanochip-ESI/LTQ-Orbitrap) m/z: $[M + H]^+$ Calcd for $C_{84}H_{85}N_{12}O_5^+$ 1341.6760; Found 1341.6747.

MS/MS fragmentation of **3wa**:

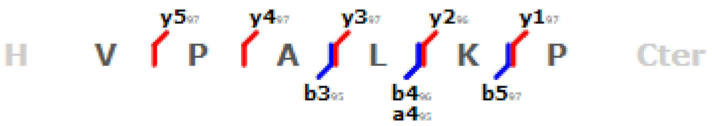

Cter = C54H32N5O-1

Sequence	Type	MF	MF Mass	m/z	Intensity	Similarity
PALKP	y5	C79H76N11O4(+1)	1242.608	1242.608	32.48	97.77%
ALKP	y4	C74H69N10O3(+1)	1145.555	1145.555	19.15	97.69%
P	y1	C59H41N6(+1)	833.3393	833.3387	34.12	97.05%
LKP	y3	C71H64N9O2(+1)	1074.518	1074.518	2.57	96.96%
PALKP	y5	C79H76N11O4(+1)	1242.608	621.8075	100.54	96.96%
VPALK	b5	C25H45N6O5(+1)	509.3451	509.3446	15.84	96.74%
KP	y2	C65H53N8O(+1)	961.4342	961.4337	3.86	96.10%
VPAL	b4	C19H33N4O4(+1)	381.2502	381.2496	5.76	96.07%
ALKP	y4	C74H69N10O3(+1)	1145.555	573.2811	1.29	95.77%
VPAL	a4	C18H33N4O3(+1)	353.2553	353.2547	3.89	95.35%
VPA	b3	C13H22N3O3(+1)	268.1661	268.1656	1.32	95.15%

AAVLLPVLLAAP 1x

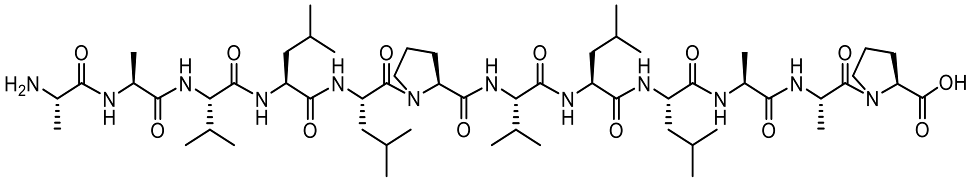

HPLC-UV chromatogram (210 nm) of AAVLLPVLLAAP (**1x**) by **Method 1**:

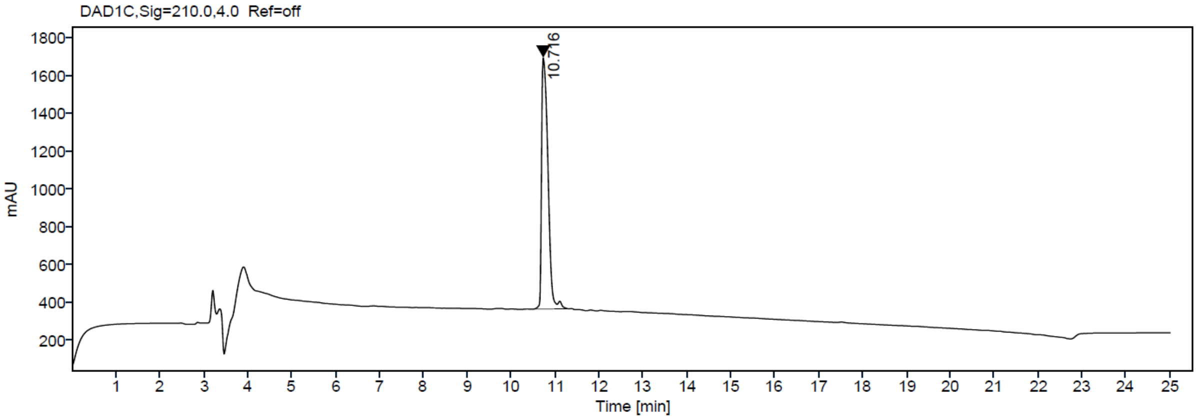

Retention time: 10.791 min **Area Percent:** 100%

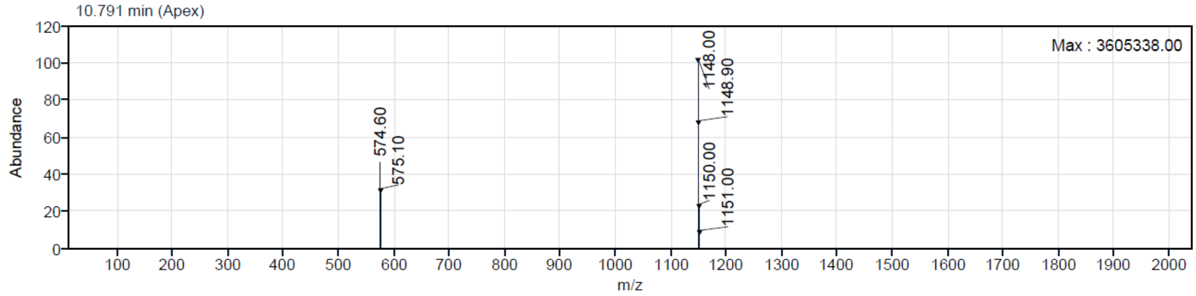

HRMS (nanochip-ESI/LTQ-Orbitrap) m/z: $[M + H]^+$ Calcd for $C_{56}H_{99}N_{12}O_{13}^+$ 1147.7449; Found 1147.7422.

AAVLLPVLLAAP 4CzBN 3xa

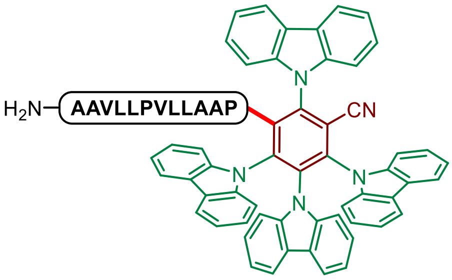

Following the general procedure (reaction time: overnight), the reaction was conducted on a10 μmol scale. The desired product **3xa** (6.4 mg, 3.4 μmol, 34% yield) was isolated by **Method 1**. More diastereomers derives from atropisomers due to the steric hindrance between 4CzBN and the terminal proline.

HPLC-UV chromatogram (210 nm) of crude by **Method 1**:

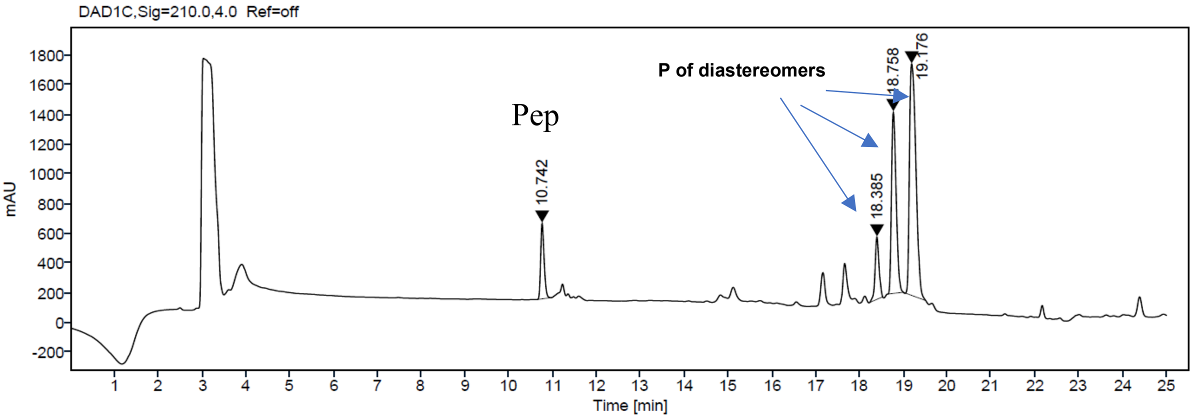

HPLC-UV chromatogram (210 nm) of **3xa** by **Method 1**:

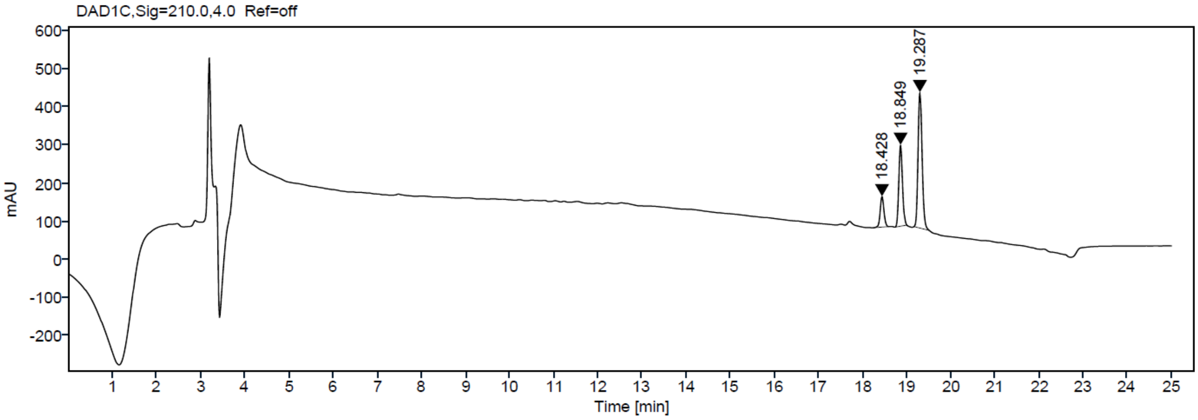

Retention time: 18.46 min **Area Percent:** 24%

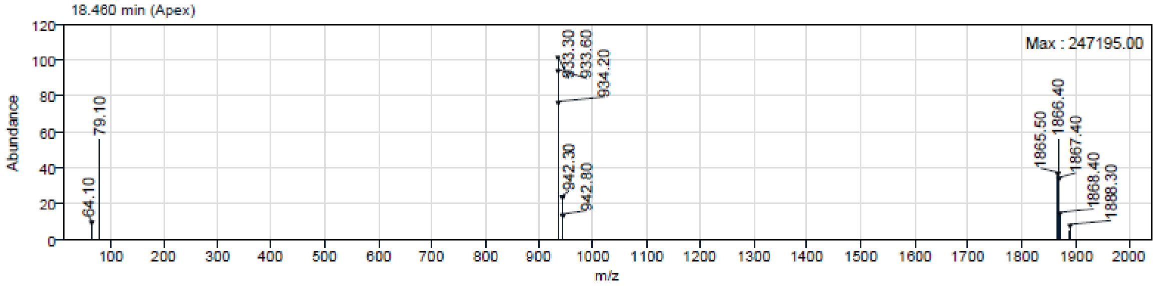

Retention time: 18.875 min **Area Percent:** 24%

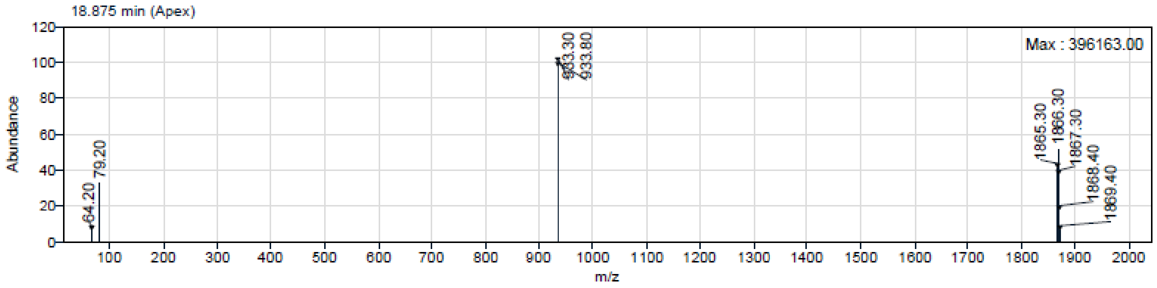

Retention time: 19.354 min **Area Percent:** 52%

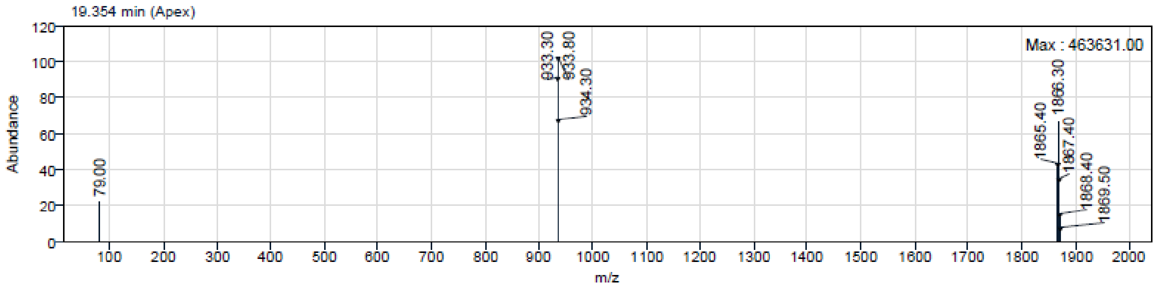

HRMS (nanochip-ESI/LTQ-Orbitrap) m/z: $[M + H_2]^{+2}$ Calcd for $C_{110}H_{131}N_{17}O_{11}^{+2}$ 933.0102; Found 933.0090.

MS/MS fragmentation of **3xa**:

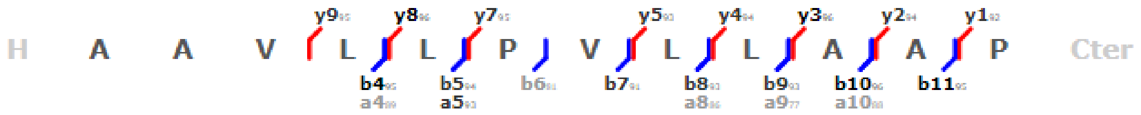

Cter = C54H32N5O-1

Sequence	Type	MF	MF Mass	m/z	Intensity	Similarity
LPVLLAAP	y8	C93H100N13O7(+1)	1510.79	1510.8	5.31	96.07%
AAP	y3	C65H51N8O2(+1)	975.414	975.41	12.29	96.02%
AAVLLPVLLA	b10	C48H85N10O10(+1)	961.645	961.64	75.45	95.84%
AAVLLPVLLAA	b11	C51H90N11O11(+1)	1032.68	1032.7	21.48	95.42%
AAVL	b4	C17H31N4O4(+1)	355.235	355.23	2.61	95.36%
LLPVLLAAP	y9	C99H111N14O8(+1)	1623.87	1623.9	1.23	94.95%
PVLLAAP	y7	C87H89N12O6(+1)	1397.7	1397.7	18.58	94.79%

AP	y2	C62H46N7O(+1)	904.376	904.38	101.05	94.13%
LAAP	y4	C71H62N9O3(+1)	1088.5	1088.5	12.01	94.01%
AAVLL	b5	C23H42N5O5(+1)	468.319	468.32	8.56	93.82%
AAVLLPVLL	b9	C45H80N9O9(+1)	890.608	890.61	27.33	93.29%
AAVLL	a5	C22H42N5O4(+1)	440.324	440.32	1.78	93.14%
AAVLLPVL	b8	C39H69N8O8(+1)	777.524	777.52	16.19	92.80%
LLAAP	y5	C77H73N10O4(+1)	1201.58	1201.6	3.23	92.60%
P	y1	C59H41N6(+1)	833.339	833.34	50.24	92.30%
AAVLLPV	b7	C33H58N7O7(+1)	664.44	664.44	4.82	91.06%
AAVL	a4	C16H31N4O3(+1)	327.24	327.24	0.62	88.84%
AAVLLPVLLA	a10	C47H85N10O9(+1)	933.65	933.65	0.7	88.20%
AAVLLPVL	a8	C38H69N8O7(+1)	749.529	749.53	0.53	85.74%
AAVLLP	b6	C28H49N6O6(+1)	565.371	565.37	1.02	81.50%
AAVLLPVLL	a9	C44H80N9O8(+1)	862.613	862.61	0.62	76.76%

KFFKFFKFFKP 1y

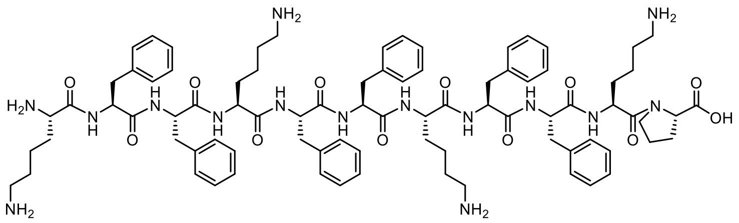

HPLC-UV chromatogram (210 nm) of (KFF)$_3$KP (**1y**) by **Method 1**:

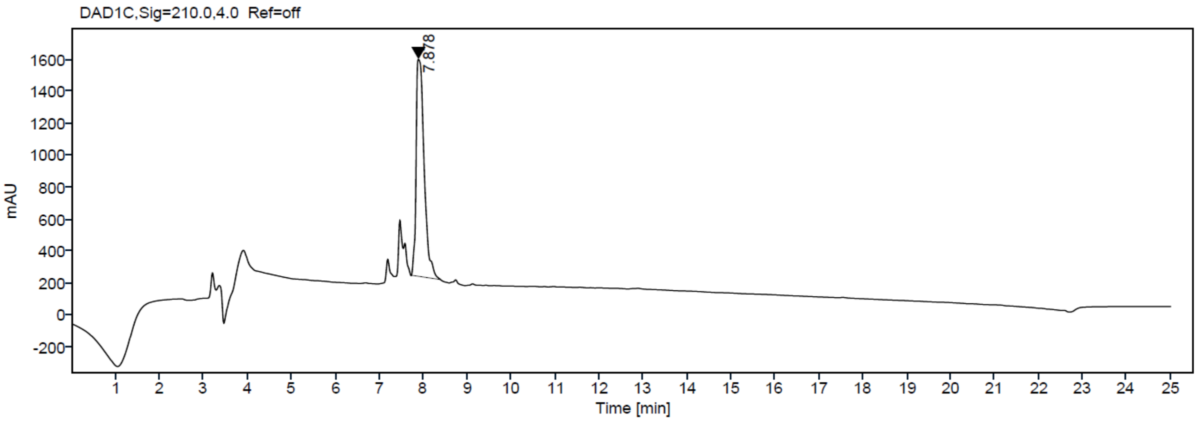

Retention time: 7.916 min **Area Percent:** 100%

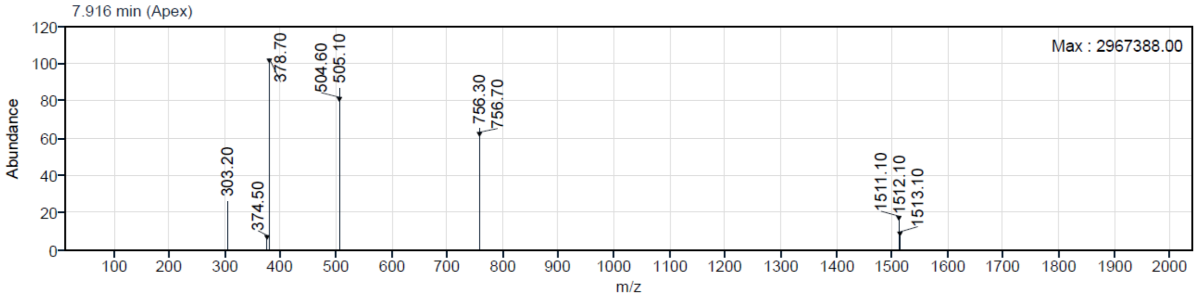

HRMS (nanochip-ESI/LTQ-Orbitrap) m/z: $[M + H_2]^{+2}$ Calcd for $C_{83}H_{113}N_{15}O_{12}^{+2}$ 755.9341; Found 755.9321.

KFFKFFKFFKP 4CzBN 3ya

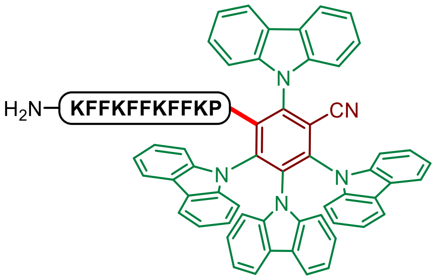

Following the general procedure (reaction time: overnight), the reaction was conducted on a 10 μmol scale. The desired product **3ya** (6.3 mg, 2.8 μmol, 28% yield) was isolated by **Method 3.**

HPLC-UV chromatogram (210 nm) of the crude by **Method 1**:

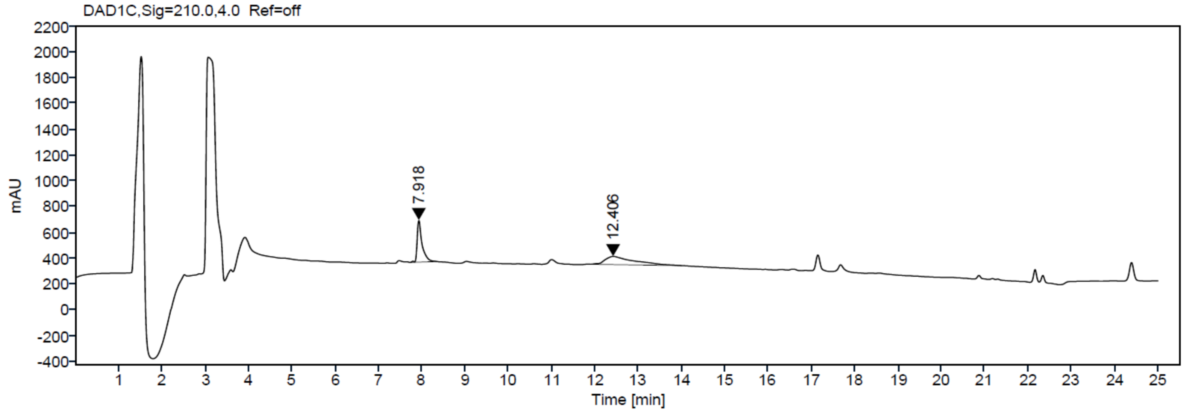

HPLC-UV chromatogram (210 nm) of product by **Method 1**:

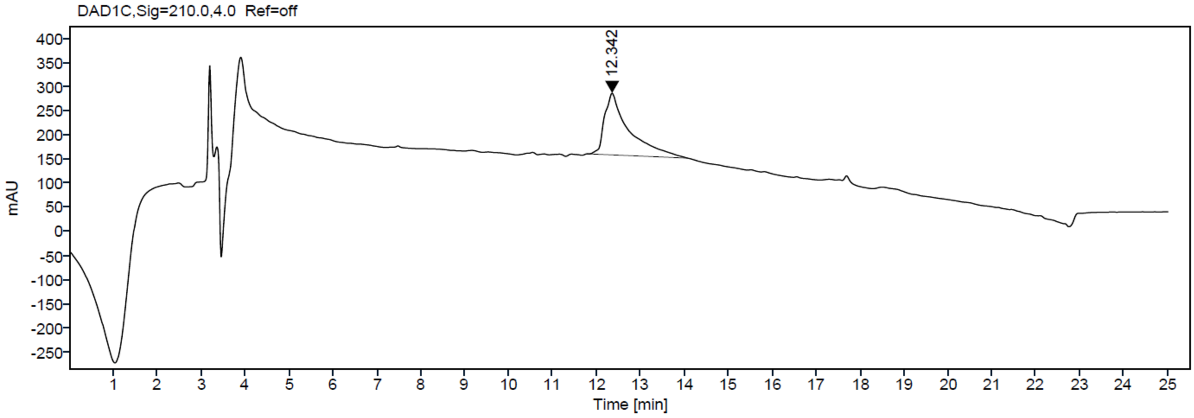

Retention time: 12.363 min **Area Percent:** 100%

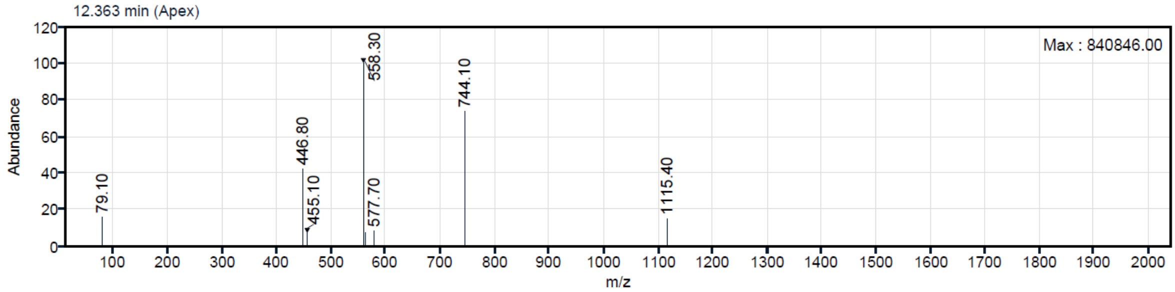

HRMS (nanochip-ESI/LTQ-Orbitrap) m/z: $[M + H_3]^{+3}$ Calcd for $C_{137}H_{145}N_{20}O_{10}^{+3}$ 743.3812; Found 743.3816.

MS/MS fragmentation of **3ya**:

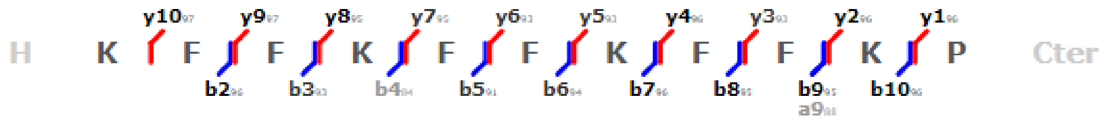

Cter = C54H32N5O-1

Sequence	Type	MF	MF Mass	m/z	Intensity	Similarity
FFKFFKFFKP	y10	C131H131N18O9(+1)	2100	1050.5	70.52	99.42%
KFFKFFK	b7	C54H73N10O7(+1)	973.57	487.29	1.67	97.35%
FKFFKFFKP	y9	C122H122N17O8(+1)	1953	976.99	26.47	97.05%
FFKP	y4	C83H71N10O3(+1)	1255.6	1255.6	4.11	96.45%
KFFKFFKFFK	b10	C78H103N14O10(+1)	1395.8	698.4	61.45	96.36%
KP	y2	C65H53N8O(+1)	961.43	961.43	7.16	96.31%
P	y1	C59H41N6(+1)	833.34	833.34	95.18	96.27%
KF	b2	C15H22N3O2(+1)	276.17	276.17	2.6	95.58%
KFFKFFKFF	b9	C72H91N12O9(+1)	1267.7	1267.7	8.98	95.51%
KFFKFFKP	y8	C113H113N16O7(+1)	1805.9	903.45	4.67	95.41%
KFFKFFK	b7	C54H73N10O7(+1)	973.57	973.57	3.02	95.30%
KFFKFFKF	b8	C63H82N11O8(+1)	1120.6	1120.6	5.1	95.28%
KFFKP	y5	C89H83N12O4(+1)	1383.7	1383.7	1.14	95.28%
FKFFKP	y6	C98H92N13O5(+1)	1530.7	765.87	1.36	95.27%
FFKFFKFFKP	y10	C131H131N18O9(+1)	2100	700.68	2.2	95.25%
FFKFFKP	y7	C107H101N14O6(+1)	1677.8	1677.8	1.49	95.21%
KFFKFFKFF	b9	C72H91N12O9(+1)	1267.7	634.36	10.86	95.20%
KFFKFFKF	b8	C63H82N11O8(+1)	1120.6	560.82	1.38	94.92%
KFFKFF	b6	C48H61N8O6(+1)	845.47	845.47	7.34	93.94%
FFKFFKP	y7	C107H101N14O6(+1)	1677.8	839.4	4.65	93.81%
FKP	y3	C74H62N9O2(+1)	1108.5	1108.5	2.16	93.33%
KFFK	b4	C30H43N6O4(+1)	551.33	551.33	4.3	92.97%
KFF	b3	C24H31N4O3(+1)	423.24	423.24	2.94	92.52%
FKFFKP	y6	C98H92N13O5(+1)	1530.7	1530.7	1.03	91.46%
KFFKF	b5	C39H52N7O5(+1)	698.4	698.4	61.45	91.45%
KFFKP	y5	C89H83N12O4(+1)	1383.7	692.34	0.92	90.85%
KFFKFFKFF	a9	C71H91N12O8(+1)	1239.7	620.36	0.75	88.25%
KFFK	b4	C30H43N6O4(+1)	551.33	276.17	2.6	74.67%

AcHFGP 8Cl-4CzBN 3ab

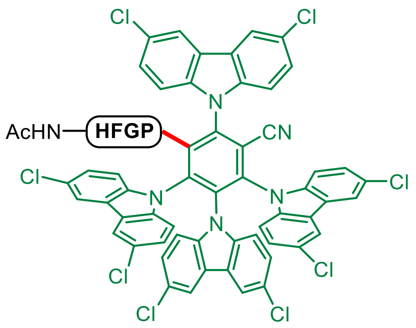

Following the general procedure, the reaction was conducted on a 10 μmol scale. The desired product **3ab** (5.8 mg, 5.1 μmol, 51% yield) was isolated by **Method 2.**

HPLC-UV chromatogram (210 nm) of the crude by **Method 1**:

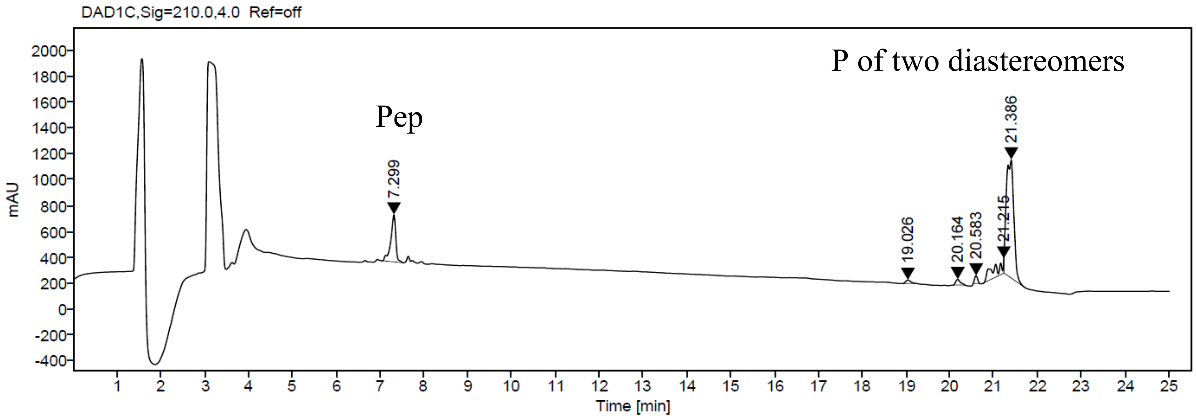

HPLC-UV chromatogram (210 nm) of **3ab** by **Method 1**:

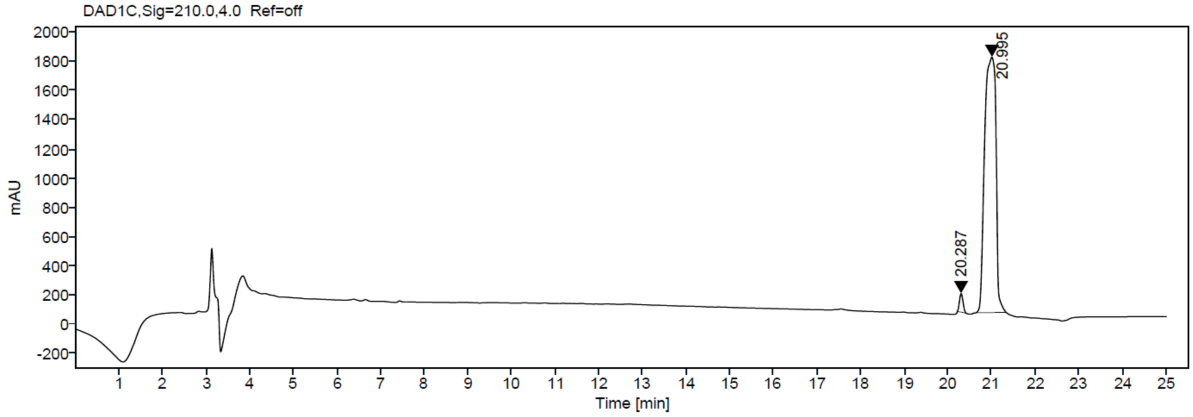

Retention time: 20.988 min **Area Percent:** 100%

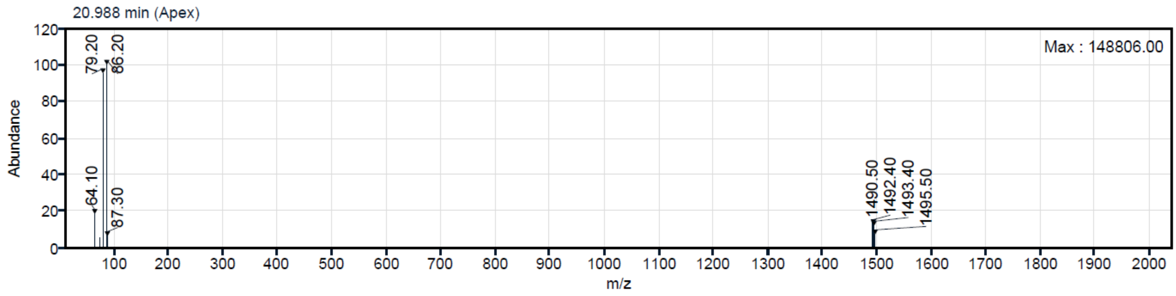

HRMS (nanochip-ESI/LTQ-Orbitrap) m/z: $[M + H]^+$ Calcd for $C_{78}H_{54}Cl_8N_{11}O_4^+$ 1488.1863; Found 1488.1841.

MS/MS fragmentation of **3ab**

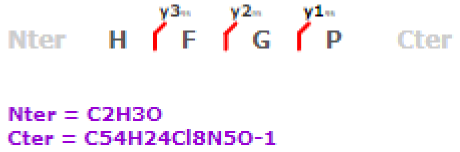

Sequence	Type	MF	MF Mass	m/z	Intensity	Similarity
P	y1	C59H33Cl8N6(+1)	1105.0275	1105.027	1.15	95.13%
GP	y2	C61H36Cl8N7O(+1)	1162.049	1162.048	0.76	94.76%
FGP	y3	C70H45Cl8N8O2(+1)	1309.1174	1309.117	4.11	94.56%

KFFKFFKFFKP 8Cl-4CzBN 3yb

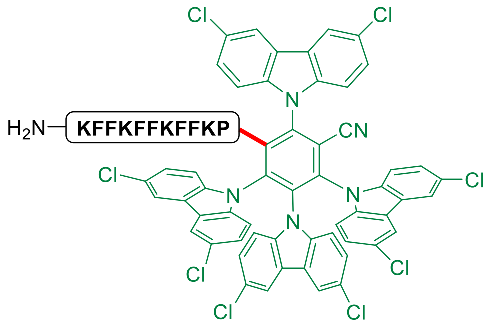

Following the general procedure (reaction time: overnight), the reaction was conducted on a 10 µmol scale. The desired product **3yb** (2.5 mg, 2.0 µmol, 20% yield) was isolated by **Method 3.**

HPLC-UV chromatogram (210 nm) of the crude by **Method 1**:

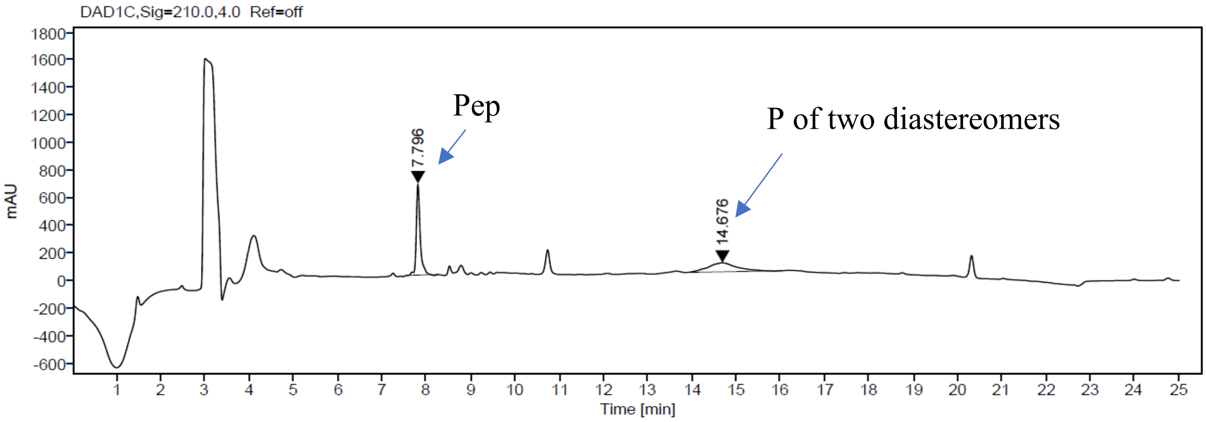

HPLC-UV chromatogram (210 nm) of **3yb** by **Method 1**:

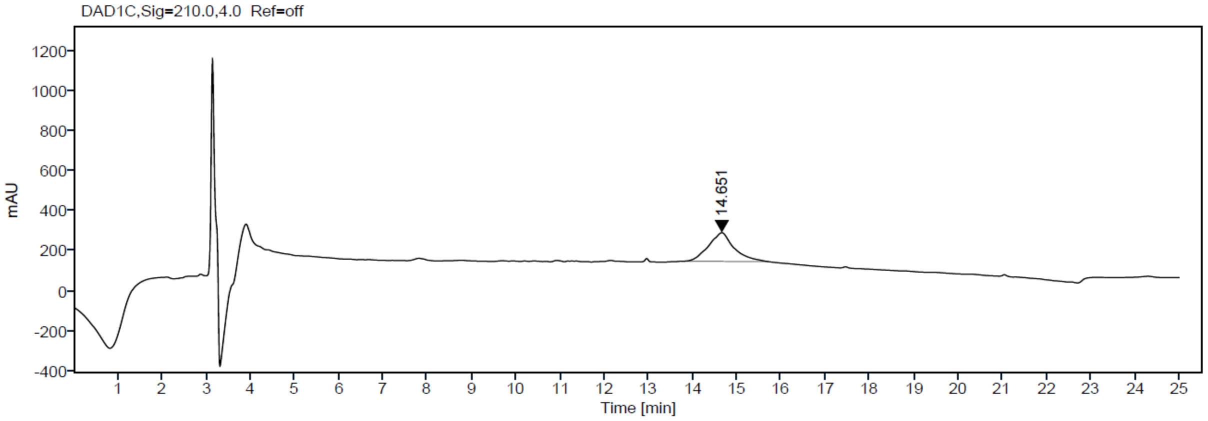

Retention time: 14.698 min **Area Percent:** 100%

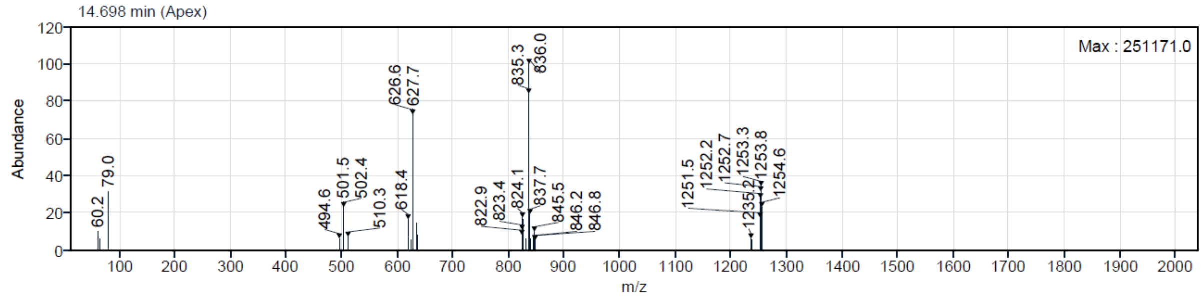

HRMS (standard-ESI/LTQ-Orbitrap) m/z: $[M + H_2]^{+2}$ Calcd for $C_{137}H_{136}Cl_8N_{20}O_{10}^{+2}$ 1250.4123; Found 1250.4073.

MS/MS fragmentation of **3yb:**

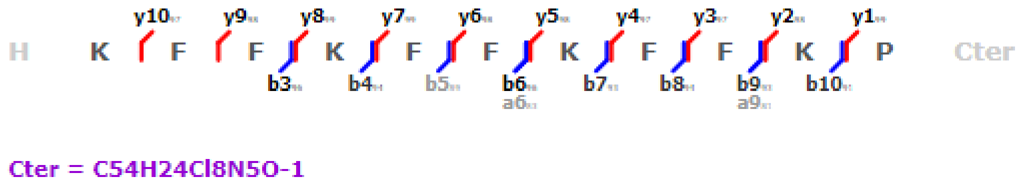

Sequence	Type	MF	MF Mass	m/z	Intensity	Similarity
P	y1	C59H33Cl8N6(+1)	1105	1105	10.74	99.06%
KFFKFFKP	y8	C113H105Cl8N16O7(+1)	2077.6	2077.6	2.32	99.02%
FKFFKFFKP	y9	C122H114Cl8N17O8(+1)	2224.7	2224.7	4.32	98.94%
FFKFFKP	y7	C107H93Cl8N14O6(+1)	1949.5	1949.5	4.9	98.72%
KFFKP	y5	C89H75Cl8N12O4(+1)	1655.4	1655.4	2.33	98.09%
FKFFKP	y6	C98H84Cl8N13O5(+1)	1802.4	1802.4	3.05	97.79%
FFKFFKFFKP	y10	C131H123Cl8N18O9(+1)	2371.7	2371.7	4.62	97.73%
KP	y2	C65H45Cl8N8O(+1)	1233.1	1233.1	17.19	97.70%
FFKP	y4	C83H63Cl8N10O3(+1)	1527.3	1527.3	7.01	96.89%
FKFFKFFKP	y9	C122H114Cl8N17O8(+1)	2224.7	1112.8	1.53	96.83%
FKP	y3	C74H54Cl8N9O2(+1)	1380.2	1380.2	3.36	96.82%
KFF	b3	C24H31N4O3(+1)	423.24	423.24	8.68	96.18%
KFFKFF	b6	C48H61N8O6(+1)	845.47	845.47	38.67	95.96%
FFKFFKFFKP	y10	C131H123Cl8N18O9(+1)	2371.7	1186.4	7.53	95.86%
KFFKFFKFFK	b10	C78H103N14O10(+1)	1395.8	1395.8	20.69	94.56%

KFFKFFKF	b8	C63H82N11O8(+1)	1120.6	1120.6	39.41	94.14%
KFFK	b4	C30H43N6O4(+1)	551.33	551.33	16.54	94.03%
KFFKFFKFF	b9	C72H91N12O9(+1)	1267.7	1267.7	103.59	94.00%
KFFKFFK	b7	C54H73N10O7(+1)	973.57	973.57	38.92	93.18%
KFFKFFKFF	b9	C72H91N12O9(+1)	1267.7	634.36	10.84	92.83%
KFFKF	b5	C39H52N7O5(+1)	698.4	698.4	63.93	88.55%
KFFKFFKFFK	b10	C78H103N14O10(+1)	1395.8	698.4	63.93	87.36%
KFFKFFKFF	a9	C71H91N12O8(+1)	1239.7	1239.7	2.27	83.68%
KFFKFF	a6	C47H61N8O5(+1)	817.48	817.48	1.92	83.25%
KFFKFFKFF	a9	C71H91N12O8(+1)	1239.7	620.36	1.12	79.01%

AcHFGP 3CzClIPN 3ac

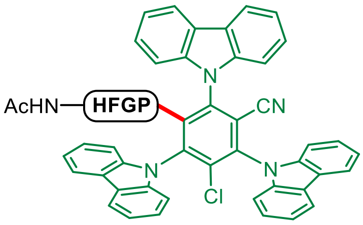

Following the general procedure, the reaction was conducted on a10 μmol scale. The desired product **3ac** (4.9 mg, 4.5 μmol, 45% yield) was isolated by **Method 3.**
HPLC-UV chromatogram (210 nm) of the crude by **Method 1**:

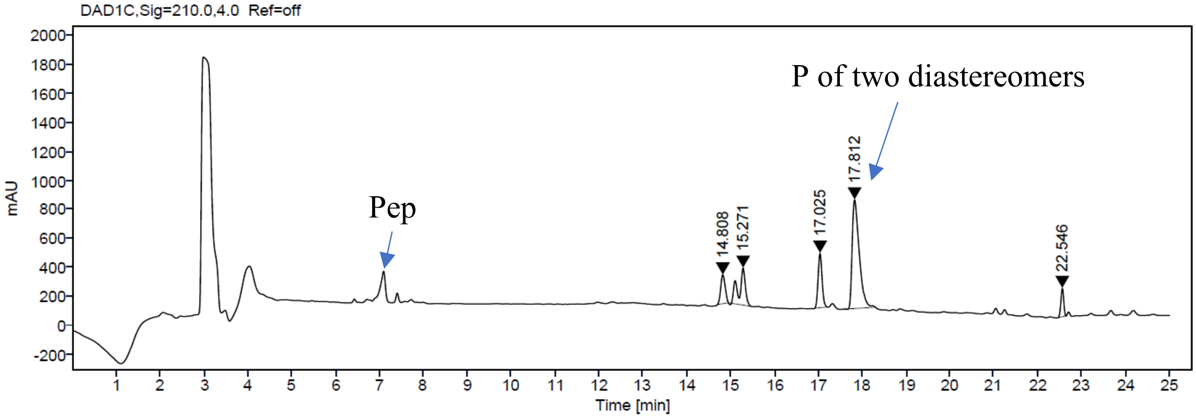

HPLC-UV chromatogram (210 nm) of **3ac** by **Method 1**:

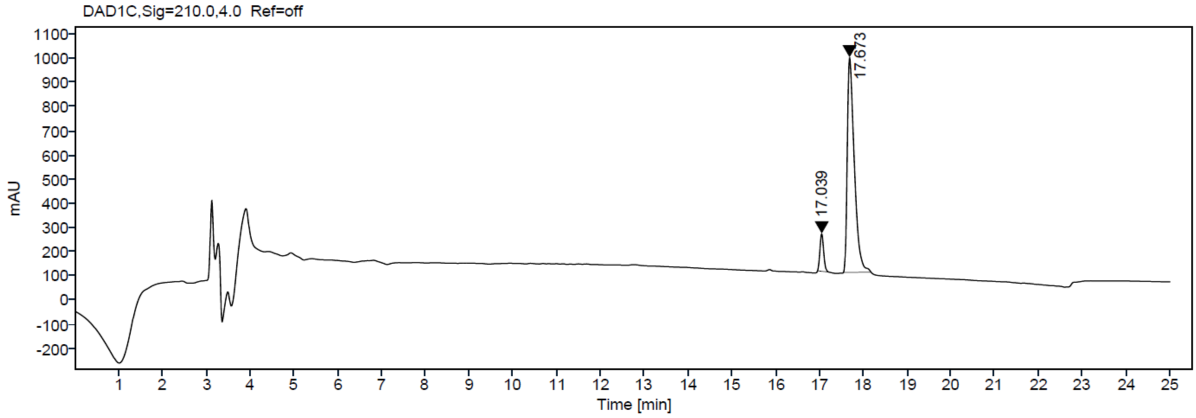

Retention time: 17.85 min Area Percent: 100%

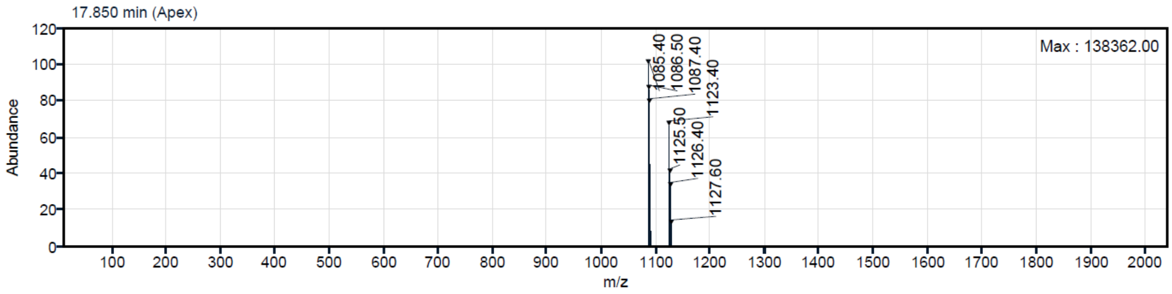

HRMS (nanochip-ESI/LTQ-Orbitrap) m/z: $[M + H]^+$ Calcd for $C_{66}H_{54}ClN_{10}O_4^+$ 1085.4013; Found 1085.4011.

MS/MS fragmentation of **3ac**

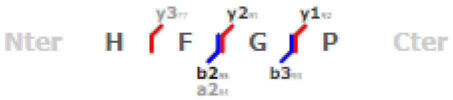

Nter = CH3CO
Cter = C42H24N4ClO-1

Sequence	Type	MF	MF Mass	m/z	Intensity	Similarity
HF	b2	C17H19N4O3(+1)	327.1457	327.1452	91.2	95.06%
HFG	b3	C19H22N5O4(+1)	384.1672	384.1666	18.5	93.14%
P	y1	C47H33ClN5(+1)	702.2424	702.2419	100.28	92.07%
HFGP		C66H53ClN10O4	1084.394	1085.401	17.31	90.84%
GP	y2	C49H36ClN6O(+1)	759.2639	759.2634	80.76	90.64%
HF	a2	C16H19N4O2(+1)	299.1508	299.1503	9.94	84.28%
FGP	y3	C58H45ClN7O2(+1)	906.3323	906.3318	7.83	76.94%

AcHFGP 2CzPN 3ad

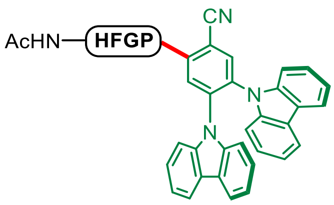

Following the general procedure (Kessil lamp 390 nm), the reaction was conducted on a10 μmol scale. The desired product **3ad** (3.1 mg, 3.5 μmol, 35% yield) was isolated by **Method 3.**

HPLC-UV chromatogram (210 nm) of the crude by **Method 1**:

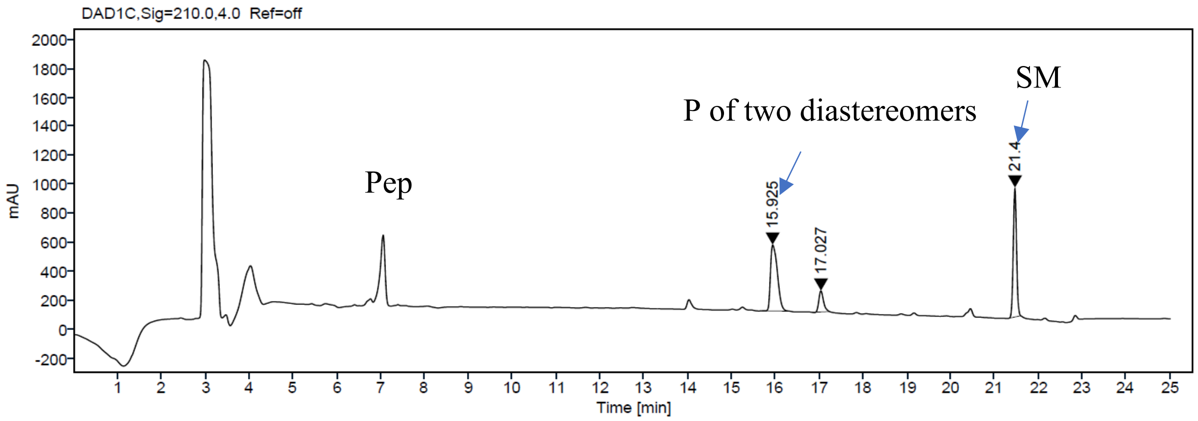

HPLC-UV chromatogram (210 nm) of **190ad** by **Method 1**:

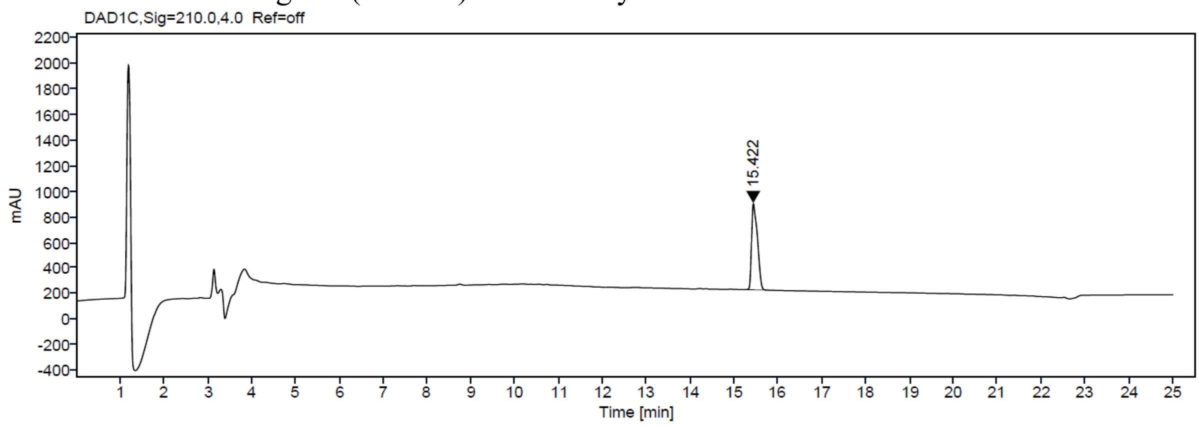

Retention time: 15.458 min **Area Percent:** 100%

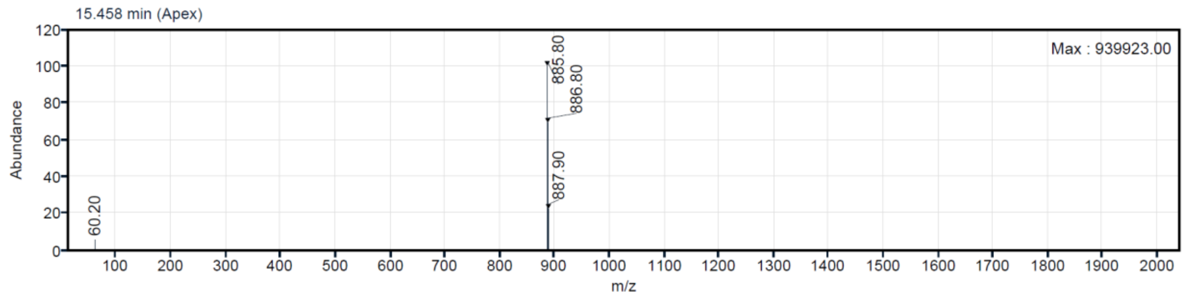

HRMS (nanochip-ESI/LTQ-Orbitrap) m/z: $[M + H]^+$ Calcd for $C_{54}H_{48}N_9O_4^+$ 886.3824; Found 886.3828.

MS/MS fragmentation of **3ad**

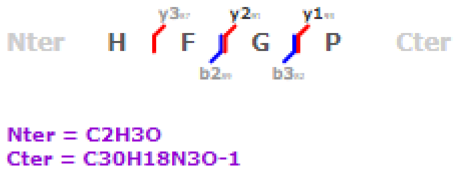

Sequence	Type	MF	MF Mass	m/z	Intensity	Similarity
P	y1	C35H27N4(+1)	503.2236	503.223	36.96	92.68%
GP	y2	C37H30N5O(+1)	560.245	560.2445	18.6	91.26%
HF	b2	C17H19N4O3(+1)	327.1457	327.1452	14.46	89.34%
FGP	y3	C46H39N6O2(+1)	707.3134	707.3129	100.56	87.04%

HFG	b3	C19H22N5O4(+1)	384.1672	384.1666	8.58	82.10%

AcHFGP BINOL-2CzPN 3ae

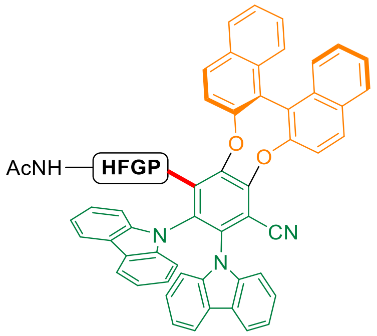

Following the general procedure, the reaction was conducted on a10 μmol scale. The desired product **3ae** (6.5 mg, 5.6 μmol, 56% yield) was isolated by **Method 3.**

HPLC-UV chromatogram (210 nm) of the crude by **Method 1**:

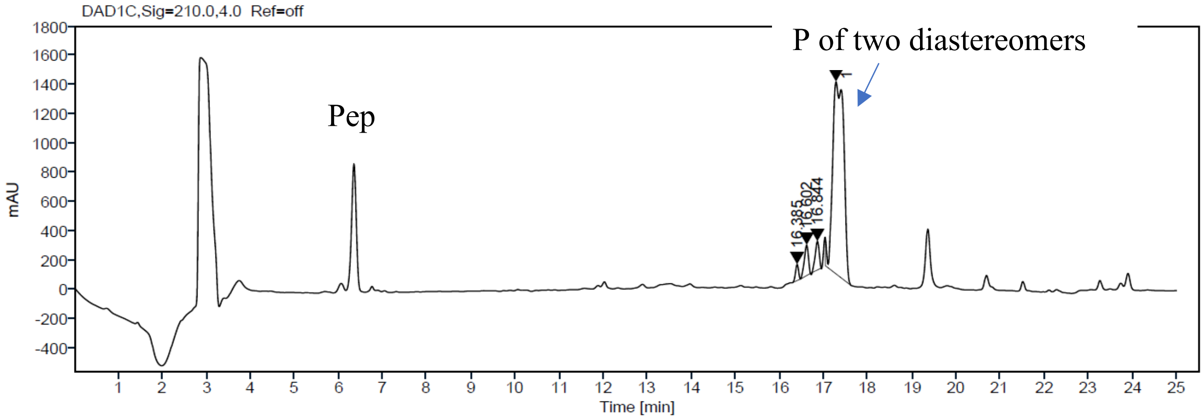

HPLC-UV chromatogram (210 nm) of **3ae** by **Method 1**:

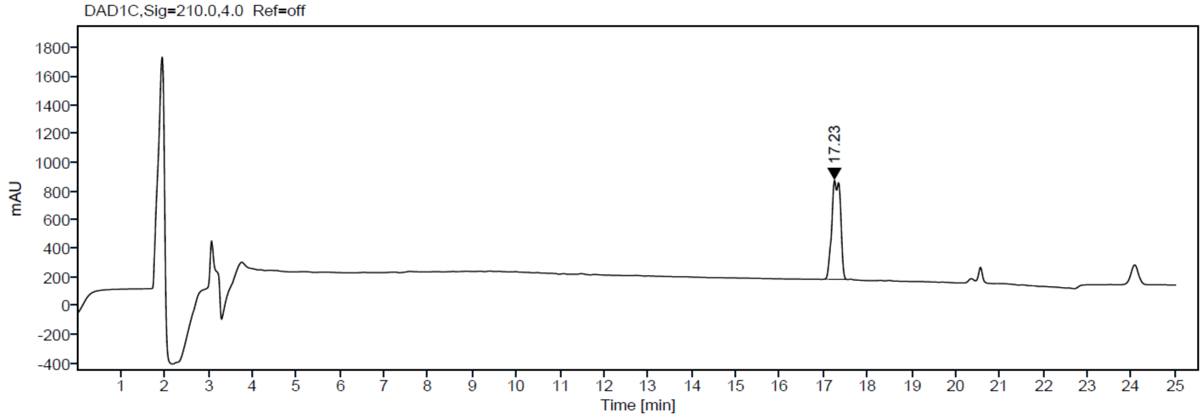

Retention time: 17.294 min **Area Percent:** 100%

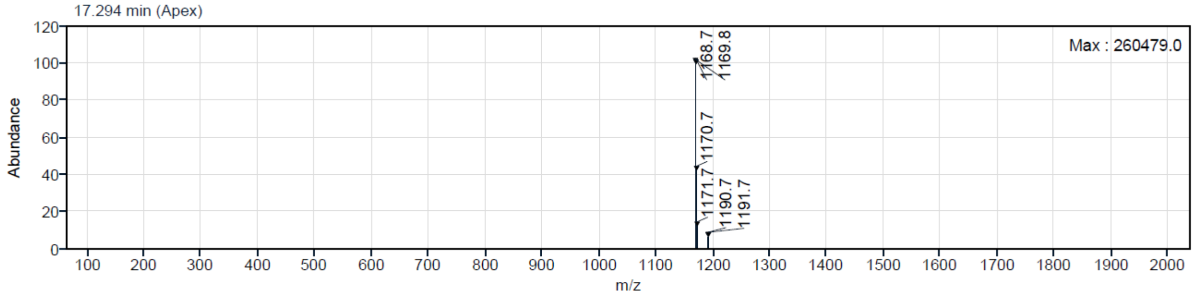

HRMS (nanochip-ESI/LTQ-Orbitrap) m/z: $[M + H]^+$ Calcd for $C_{74}H_{58}N_9O_6^+$ 1168.4505; Found 1168.4530.

MS/MS fragmentation of **3ae**:

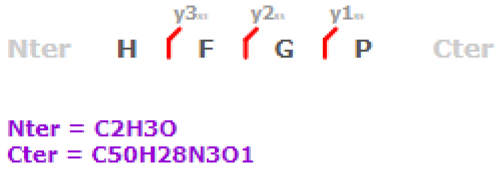

Sequence	Type	MF	MF Mass	m/z	Intensity	Similarity
GP	y2	C57H40N5O3(+1)	842.31	842.31	41.07	85.17%
P	y1	C55H37N4O2(+1)	785.29	785.29	101.46	85.15%
FGP	y3	C66H49N6O4(+1)	989.38	989.38	16.03	82.90%

5. Synthesis of CzIPN-peptide conjugates via S_NAr

5.1 General procedure for the synthesis of 3CzIPN-peptide conjugates

For peptide smaller than pentamers: Cys containing peptide **4** (0.011 mmol, 1.1 equiv.) and 3CzFIPN **5a** (10 µmol, 1 equiv.) were weighed on the analytical balance and dissolved in 0.75 mL non-degassed DMSO and 0.25 mL 100 mM Tris buffer pH 11.1 in a 5 mL vial, overall concentration: 5 mM. The reaction was stirred overnight. The 3CzPN-conjugates were isolated by Prep-RP-HPLC, followed by lyophilization.

For peptide larger than pentamers: Cys containing peptide **4** (0.010 mmol, 1.0 equiv.) and 3CzFIPN **5a** (0.011 mmol, 1.1 equiv.) were weighed on the analytical balance and dissolved in 0.75 mL non-degassed DMSO and 0.25 mL 100 mM Tris buffer pH 11.1 in a 5 mL vial, overall concentration: 5 mM. The reaction was stirred overnight. The 3CzPN-conjugates were isolated by Prep-RP-HPLC, followed by lyophilization.

5.2 Condition optimizations for S_NAr of 3CzFIPN

Table S2 Condition optimizations for S_NAr of 3CzFIPN

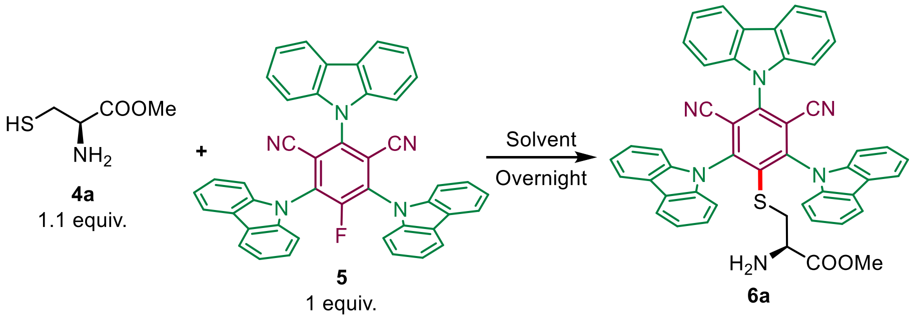

Entry	Temp (°C)	Base (2 equiv.)	Solvent	C(mM)	Yield%
1	rt	Et_3N	DMSO	10	45
2	rt	Et_3N	DMF	10	27
3	rt	Et_3N	MeCN/H_2O 9:1	10	38
4	rt	K_2HPO_4	DMSO	10	36
5	rt	K_2CO_3	DMSO	10	-

6	rt	-	DMSO/Tris pH 9.0 (4:1)[a]	10	70
7	rt	-	DMSO/Tris pH 8.2 (4:1)[a]	10	20
8	rt	-	DMSO/PB pH 9.0 (4:1)[b]	10	38
9	rt	-	DMSO/Tris pH 11.1 (4:1)[a]	10	88
10	37	-	DMSO/Tris pH 9.0 (4:1)[a]	10	60
11	37	-	DMSO/Tris pH 8.2 (4:1)[a]	10	52
12	37	-	DMSO/Tris pH 11.1 (4:1)[a]	10	83
13	rt	-	DMSO/Tris pH 11.1 (1:2)[a]	10	10
14	rt	-	DMSO/Tris pH 11.1 (1:1)[a]	10	16
15	rt	-	DMSO/Tris pH 11.1 (2:1)[a]	10	87
**16**	**rt**	**-**	**DMSO/Tris pH 11.1 (3:1)[a]**	**10**	**92**
17	rt	-	DMSO/Tris pH 11.1 (3:1)[a]	5	91
18	rt	-	DMSO/Tris pH 11.1 (3:1)[a]	20	73

The reactions were performed on a 1 μmol scale, the yield was determined based on the HPLC-UV ratio between 3CzFIPN (**5**) and **6a**. [a]100 mM Tris buffer was used. [b]PB: Phosphate buffer.

5.3 Scope of 3CzIPN-peptide conjugates

Ac-Cys-OMe 3CzIPN 6aa

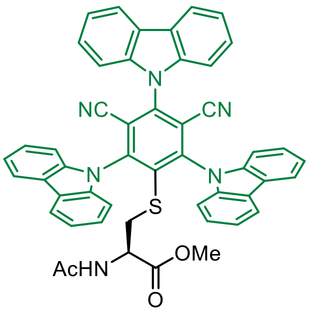

Following the general procedure, the reaction was conducted on a 0.1 mmol scale. The desired product **6aa** (54.1 mg, 0.0677 mmol, 75% yield) was isolated by column chromatography.

^{1}H NMR (500 MHz, $CDCl_3$) δ 8.16 (t, J = 7.5 Hz, 6H, ArH), 7.56 (t, J = 7.7 Hz, 6H, ArH), 7.47 – 7.37 (m, 8H, ArH), 7.32 (d, J = 8.2 Hz, 4H, ArH), 5.11 (d, J = 7.6 Hz, 1H, NH), 4.07 – 4.00 (m, 1H, α-amino CH), 3.15 (s, 3H, OCH_3), 2.09 – 2.01 (m, 2H, CH_2), 1.52 (s, 3H, CH_3CO).

^{13}C NMR (126 MHz, $CDCl_3$) δ 169.8, 169.4, 148.7, 145.5, 140.9, 140.1, 139.6, 139.5, 127.0, 124.8, 124.6, 124.5, 122.3, 122.3, 122.2, 121.3, 121.2, 121.2, 117.2, 111.3, 110.2, 110.0, 109.7, 52.5, 50.9, 36.5, 22.7.

HRMS (ESI/QTOF) m/z: $[M + Na]^+$ Calcd for $C_{50}H_{34}N_6NaO_3S^+$ 821.2305; Found 821.2289.

NH_2-Cys-OMe 3CzIPN 6ba

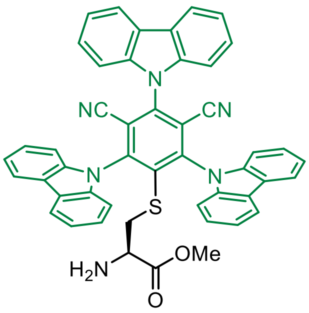

Following the **General procedure**, the reaction was conducted on a 10 µmol scale. The desired product **6ba** (5.7 mg, 7.5 µmol, 75% yield) was isolated by **Method 3.**

HPLC-UV chromatogram (210 nm) of the crude by **Method 1**:

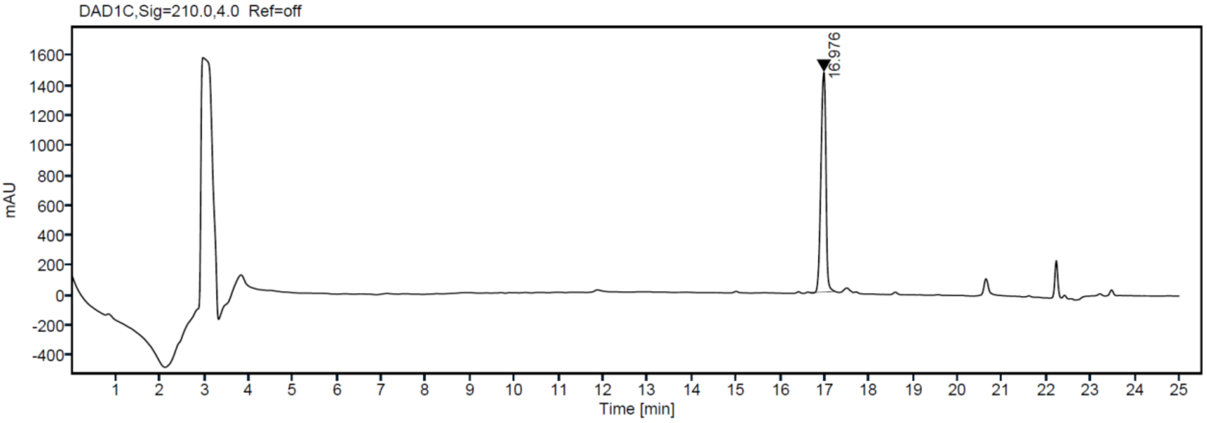

HPLC-UV chromatogram (210 nm) of **6ba** by **Method 1**:

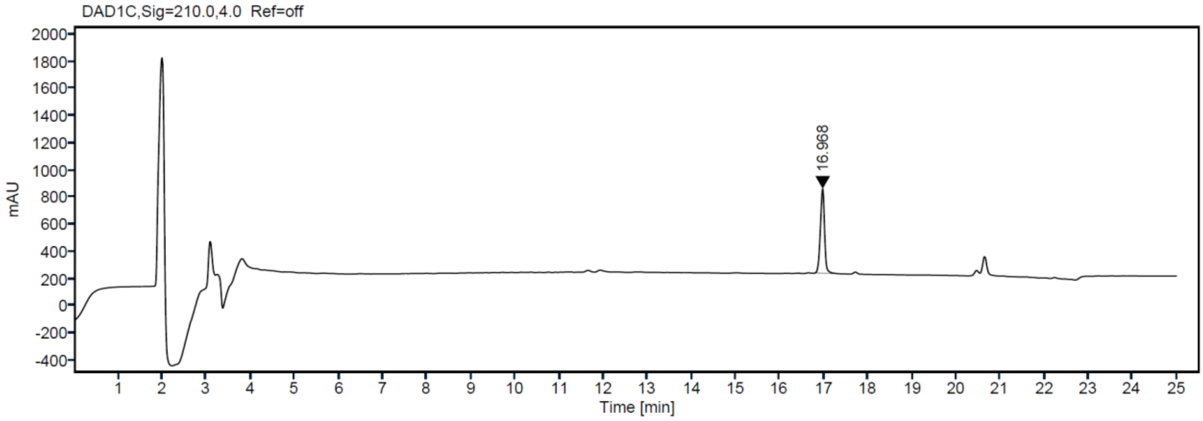

Retention time: 17.011 min **Area Percent:** 100%

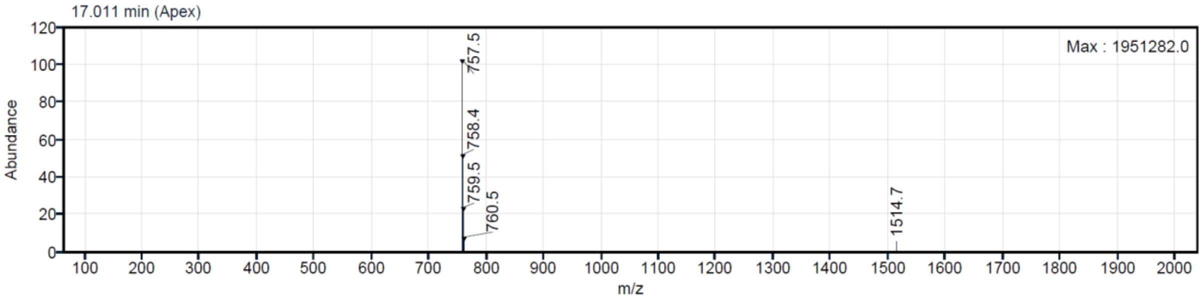

HRMS (nanochip-ESI/LTQ-Orbitrap) m/z: $[M + H]^+$ Calcd for $C_{48}H_{33}N_6O_2S^+$ 757.2380; Found 757.2393.

GSH 3CzIPN (6ca)

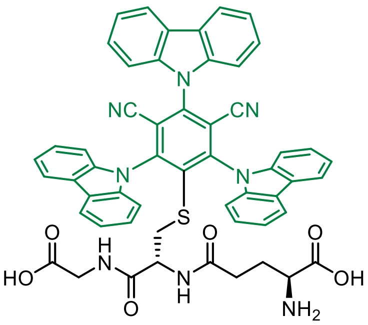

Following the **General procedure**, the reaction was conducted on a 10 µmol scale. The desired product **6ca** (8.0 mg, 8.6 µmol, 86% yield) was isolated by **Method 3.**
^{1}H NMR (500 MHz, DMSO) δ 8.29 (d, J = 7.8 Hz, 2H, ArH), 8.26 (d, J = 7.7 Hz, 3H, ArH), 7.83 (d, J = 8.2 Hz, 2H, ArH), 7.76 (d, J = 8.2 Hz, 2H, ArH), 7.69 (d, J = 8.2 Hz, 2H, ArH), 7.63 – 7.59 (m, 2H, ArH), 7.58 (d, J = 1.5 Hz, 2H, ArH), 7.57 – 7.53 (m, 2H, ArH), 7.50 (d, J = 8.2 Hz, 1H, ArH), 7.43 – 7.35 (m, 6H, ArH), 3.58 (q, J = 7.4 Hz, 2H), 3.14 (dd, J = 17.5, 5.2 Hz, 2H), 2.22 (dd, J = 12.7, 6.3 Hz, 1H), 1.95 (dd, J = 12.7, 7.6 Hz, 1H), 1.85 – 1.78 (m, 1H), 1.73 – 1.59 (m, 3H).
^{13}C NMR (126 MHz, DMSO) δ 171.0, 171.0, 169.4, 148.8, 144.8, 142.9, 140.2, 140.0, 140.0, 127.2, 127.1, 123.9, 123.8, 123.8, 122.2, 121.8, 121.5, 121.3, 117.4, 112.4, 111.4, 111.3, 111.1, 52.5, 52.3, 41.1, 36.4, 30.9, 26.4, 1.6.
HPLC-UV chromatogram (210 nm) of the crude by **Method 1**:

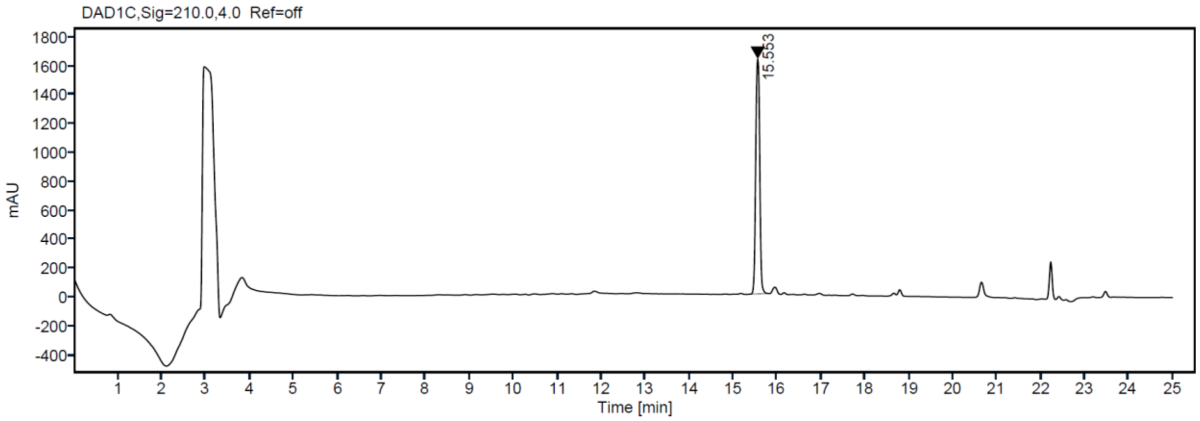

HPLC-UV chromatogram (210 nm) of **6ca** by **Method 1**:

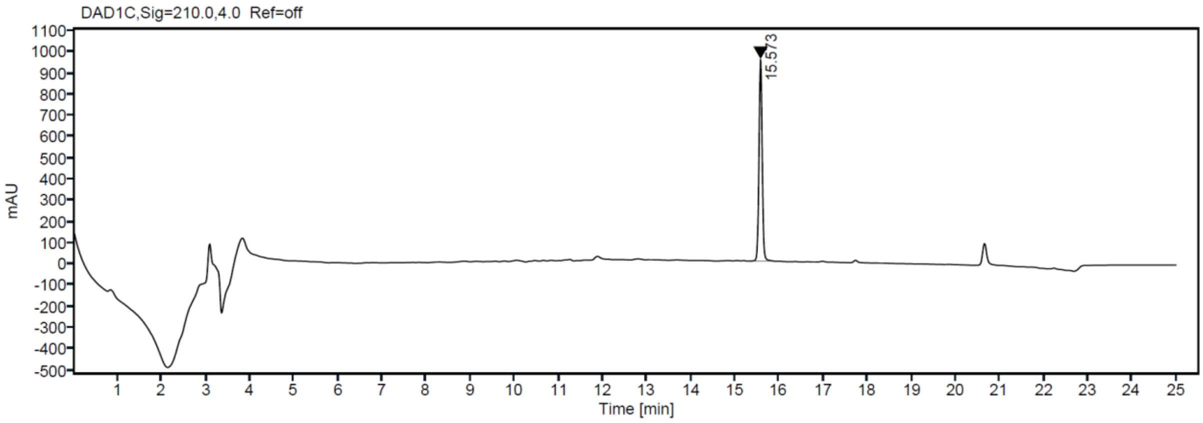

Retention time: 15.6 min **Area Percent:** 100%

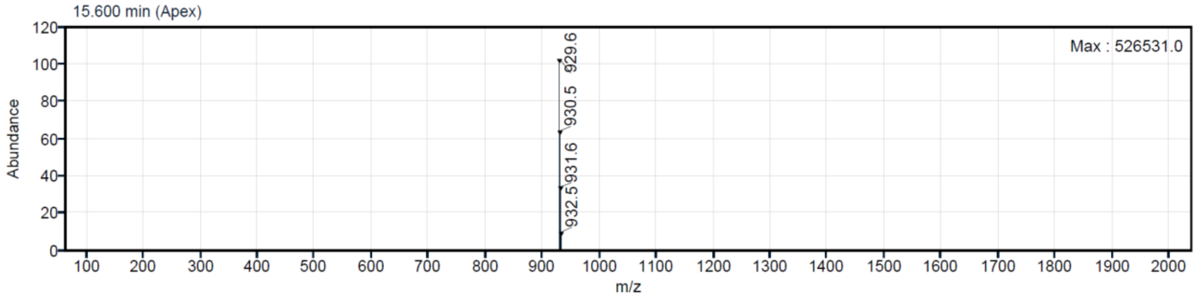

HRMS (nanochip-ESI/LTQ-Orbitrap) m/z: $[M + H]^+$ Calcd for $C_{54}H_{41}N_8O_6S^+$ 929.2864; Found 929.2886.

Ac-HGCGN-NH$_2$ (4d)

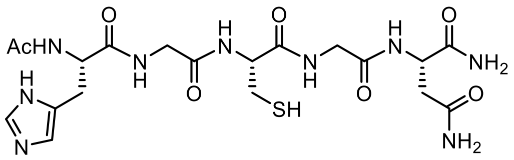

HPLC-UV chromatogram (210 nm) of **4d** by **Method 1**:

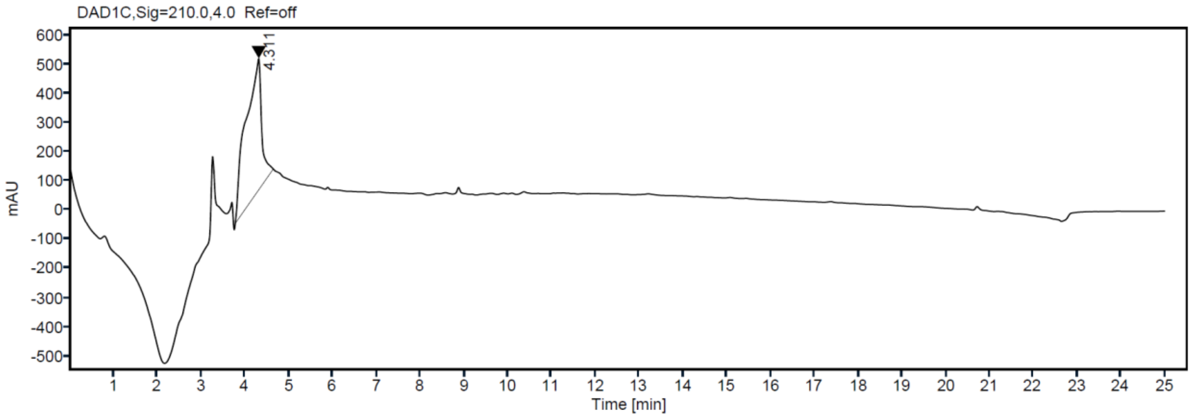

Retention time: 3.935 min **Area Percent:** 100%

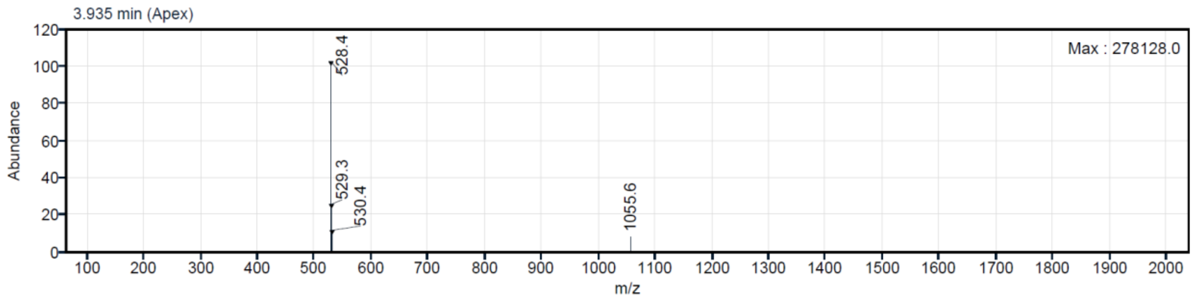

HRMS (nanochip-ESI/LTQ-Orbitrap) m/z: $[M + Na]^+$ Calcd for $C_{19}H_{29}N_9NaO_7S^+$ 550.1803; Found 550.1807.

Ac-HGCGN-NH$_2$ 3CzIPN (6da)

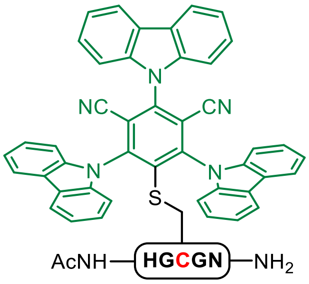

Following the **General procedure**, the reaction was conducted on a 10 µmol scale. The desired product **6da** (7.7 mg, 6.7 µmol, 67% yield) was isolated by **Method 3.**
HPLC-UV chromatogram (210 nm) of the crude by **Method 1**:

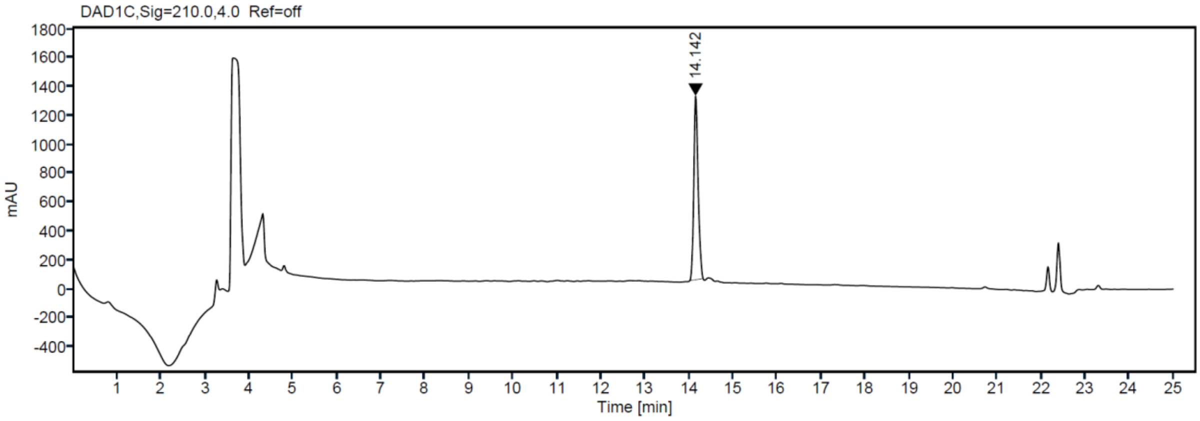

HPLC-UV chromatogram (210 nm) of **6da** by **Method 1**:

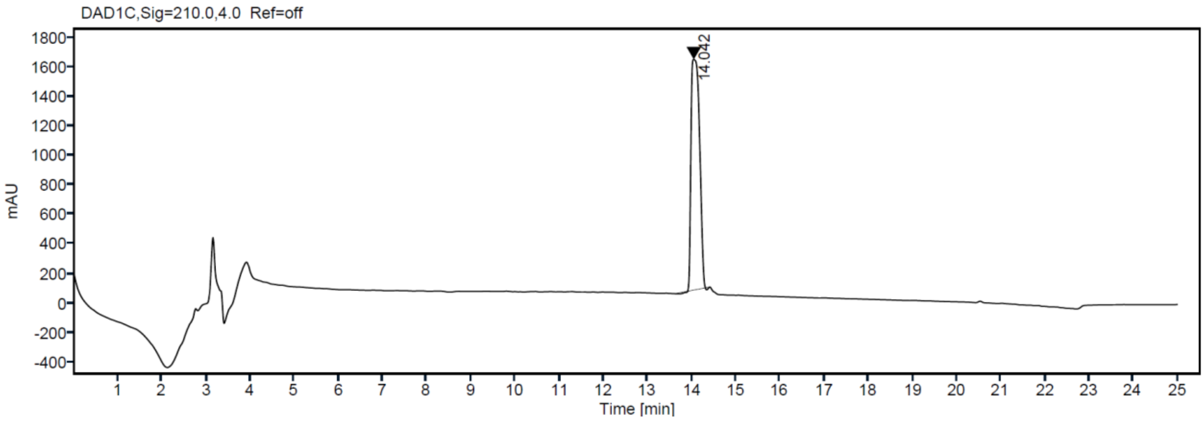

Retention time: 14.068 min **Area Percent:** 100%

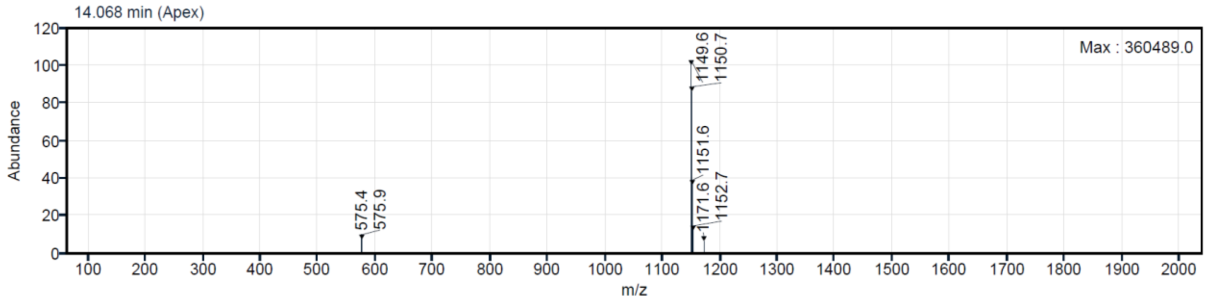

HRMS (nanochip-ESI/LTQ-Orbitrap) m/z: $[M + H]^+$ Calcd for $C_{63}H_{53}N_{14}O_7S^+$ 1149.3937; Found 1149.3940.

MS/MS fragmentation of **6da**:

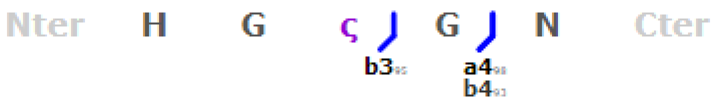

ç = Cys(C44H23N5)
Nter = C2H3O
Cter = NH2

Sequence	Type	MF	MF Mass	m/z	Intensity	Similarity
HGCG	a4	C58H44N11O4S(+1)	990.33	495.67	14.24	98.36%
HGC	b3	C57H41N10O4S(+1)	961.3	961.3	7.29	95.50%
HGCG	b4	C59H44N11O5S(+1)	1018.3	1018.3	35.12	94.88%
HGCG	b4	C59H44N11O5S(+1)	1018.3	509.67	12.62	92.04%

AcKLAFC-OH (4e)

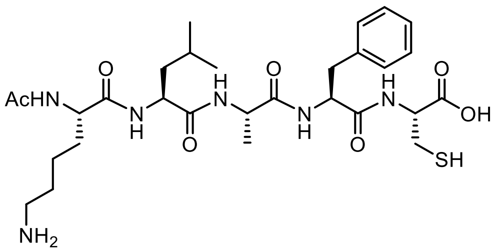

HPLC-UV chromatogram (210 nm) of **4e** by **Method 1**:

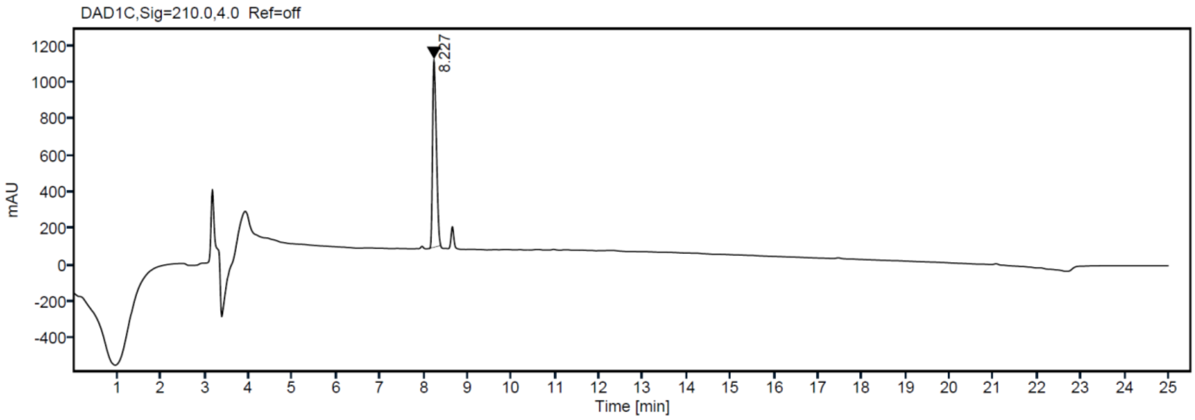

Retention time: 8.253 min **Area Percent:** 100%

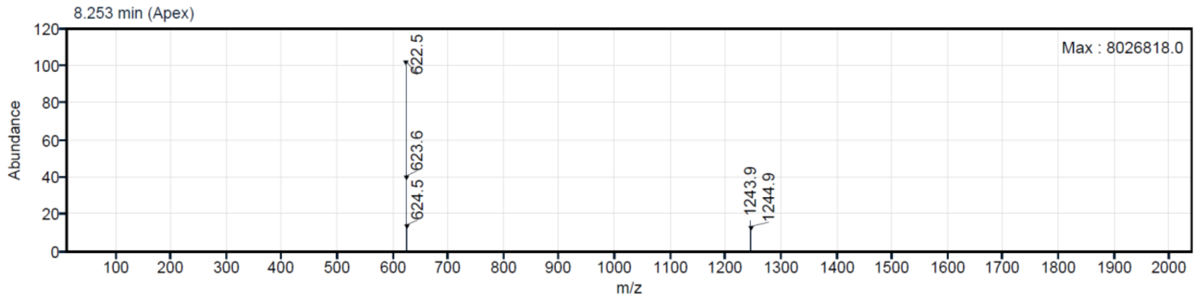

HRMS (ESI/QTOF) m/z: $[M + H]^+$ Calcd for $C_{29}H_{47}N_6O_7S^+$ 623.3221; Found 622.3371.

AcKLAFC-OH 3CzIPN (6ea)

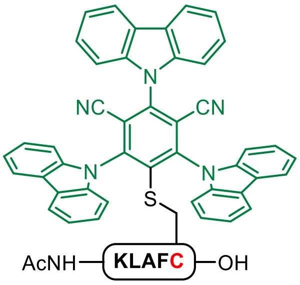

Following the **General procedure**, the reaction was conducted on a 5.1 μmol scale. The desired product **6ea** (5.1 mg, 4.1 μmol, 88% yield) was isolated by **Method 3.**
HPLC-UV chromatogram (210 nm) of the crude by **Method 1**:

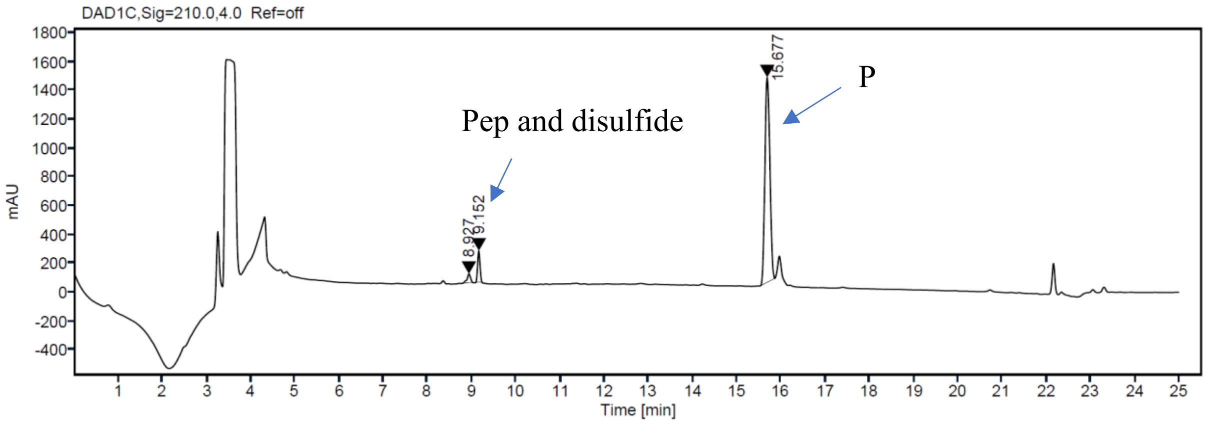

HPLC-UV chromatogram (210 nm) of **6ea** by **Method 1**:

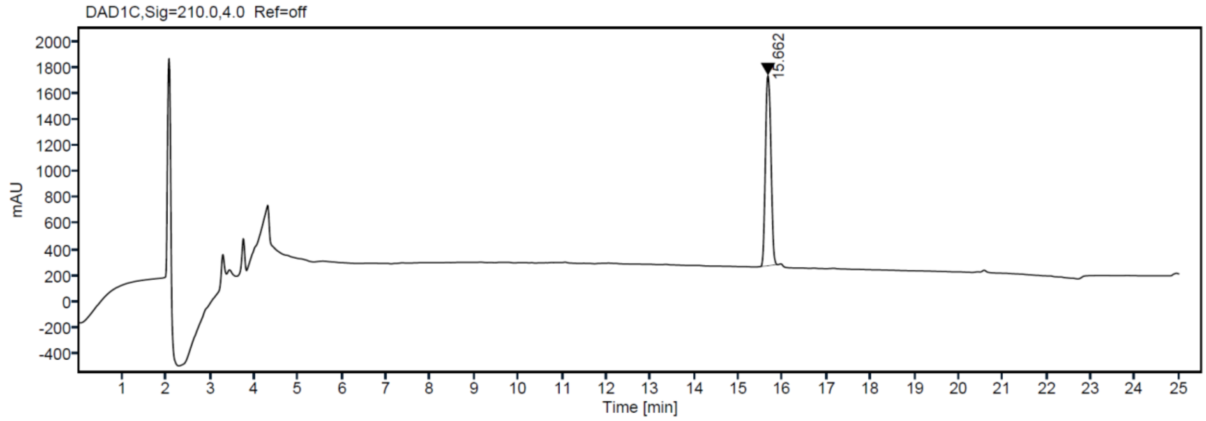

Retention time: 15.698 min **Area Percent:** 100%

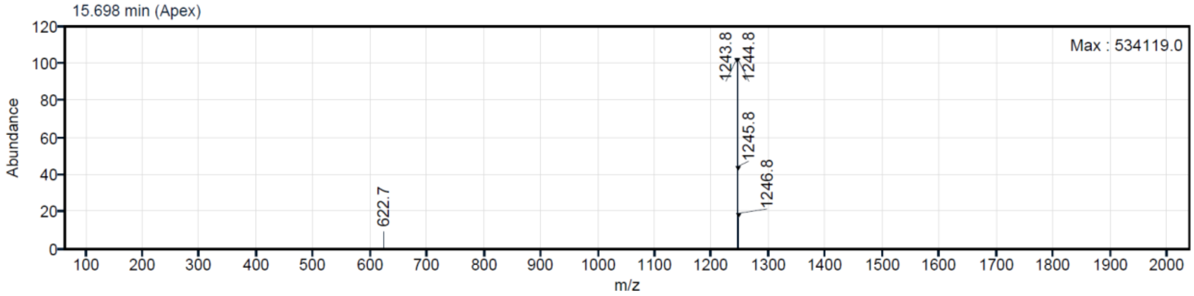

HRMS (nanochip-ESI/LTQ-Orbitrap) m/z: $[M]^+$ Calcd for $C_{73}H_{69}N_{11}O_7S^+$ 1243.5097; Found 1243.5333.

MS/MS fragmentation of **6ea**:

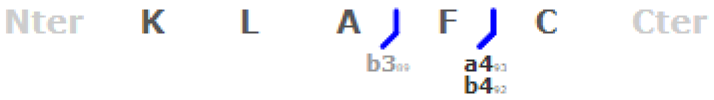

Nter = C2H3O
Cter = C44H24N5O

Sequence	Type	MF	MF Mass	m/z	Intensity	Similarity
KLAF	a4	C25H40N5O4(+1)	474.308	474.3075	21.22	92.79%

KLAF	b4	C26H40N5O5(+1)	502.3029	502.3024	84.53	92.39%
KLA	b3	C17H31N4O4(+1)	355.2345	355.234	6.08	88.63%

RKKRRQRRKC-NH2 (4f)

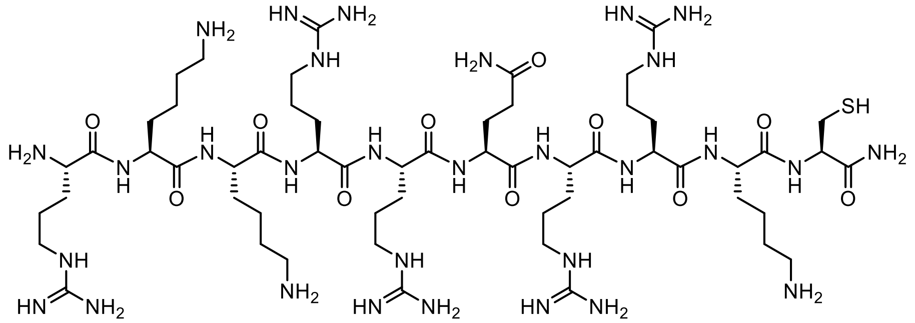

HPLC-UV chromatogram (210 nm) of **4f** by **Method 1**:

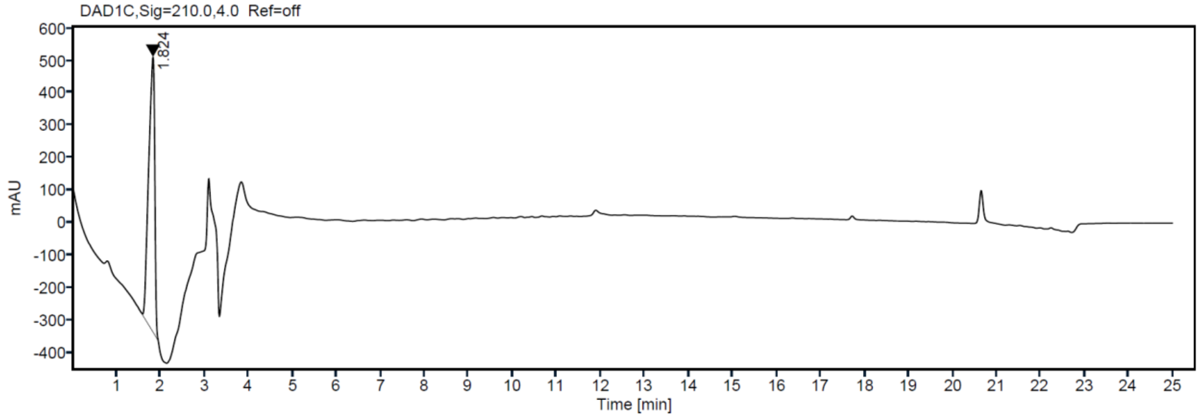

Retention time: 1.868 min **Area Percent:** 100%

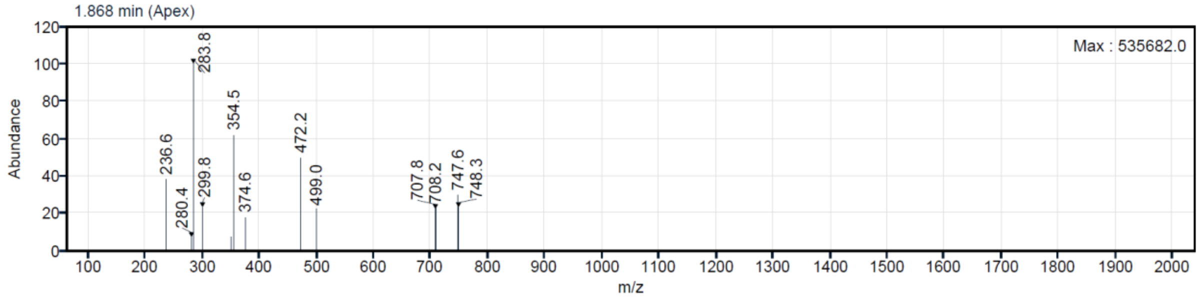

HRMS (Nanochip-based ESI/LTQ-Orbitrap) m/z: [M + H4]$^{+4}$ Calcd for $C_{56}H_{116}N_{30}O_{11}S^{+4}$ 354.2285; Found 354.2285.

RKKRRQRRKC-NH2 3CzIPN (6fa)

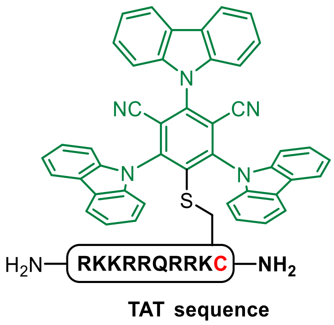

Following the **General procedure**, the reaction was conducted on a 5.0 μmol scale. The desired product **6fa** (6.4 mg, 3.1 μmol, 63% yield) was isolated by **Method 3.**

HPLC-UV chromatogram (210 nm) of the crude by **Method 1**:

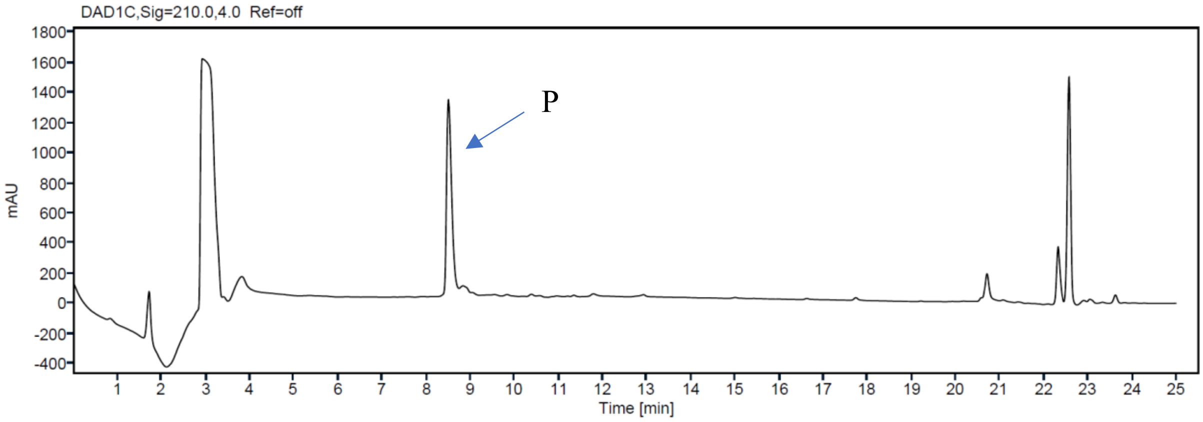

HPLC-UV chromatogram (210 nm) of **6fa** by **Method 1**:

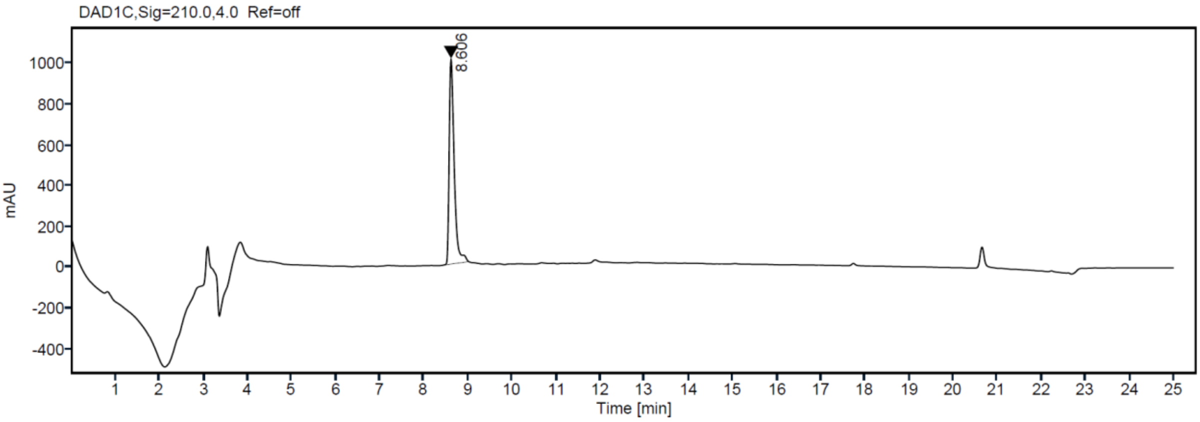

Retention time: 8.615 min **Area Percent:** 100%

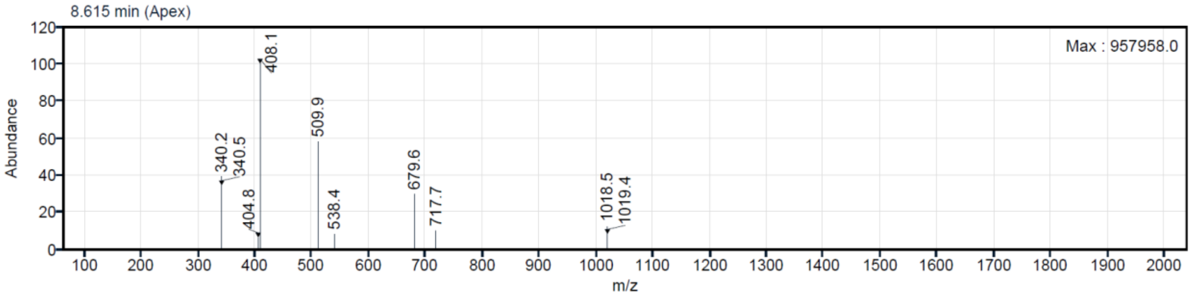

HRMS (LTQ-Orbitrap) m/z: $[M + H_4]^{+4}$ Calcd for $C_{100}H_{139}N_{35}O_{11}S^{+4}$ 509.5273; Found 509.5273.

MS/MS fragmentation of **6fa**:

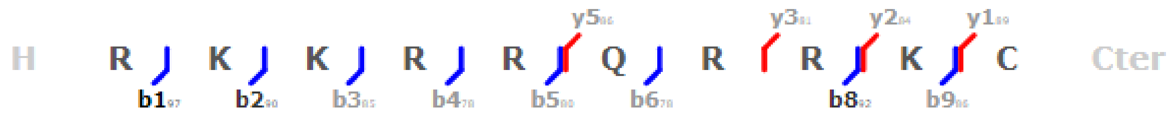

Cter = C44H23N5NH2

Sequence	Type	MF	MF Mass	m/z	Intensity	Similarity
RKKRRQRRKC		C100H135N35O11S	2034.1	509.53	47.23	98.43%
R	b1	C6H13N4O(+1)	157.11	157.11	26.55	96.92%
RKKRRQRR	b8	C47H93N26O9(+1)	1165.8	292.2	1.95	94.75%
RK	b2	C12H25N6O2(+1)	285.2	285.2	11.83	90.04%
C	y1	C47H32N7OS(+1)	742.24	742.24	8.76	89.09%
RKKRRQRR	b8	C47H93N26O9(+1)	1165.8	389.26	4.61	88.76%
RKKRRQRRK	b9	C53H105N28O10(+1)	1293.9	324.22	3.24	86.47%
QRRKC	y5	C70H76N19O6S(+1)	1310.6	655.8	1.78	85.75%
RKK	b3	C18H37N8O3(+1)	413.3	413.3	4.99	84.90%
KC	y2	C53H44N9O2S(+1)	870.33	870.33	2.2	83.72%
RKKRR	b5	C30H61N16O5(+1)	725.5	242.51	1.88	82.36%
RKKR	b4	C24H49N12O4(+1)	569.4	285.2	11.83	80.69%
RKC	y3	C59H56N13O3S(+1)	1026.4	1026.4	1.28	80.52%
RKKRR	b5	C30H61N16O5(+1)	725.5	363.25	1.9	78.34%
RKKRRQ	b6	C35H69N18O7(+1)	853.56	427.28	4.14	78.15%
RKKR	b4	C24H49N12O4(+1)	569.4	569.4	1.14	74.65%

KLAKLAKKLAKLAKC-NH₂ (4g)

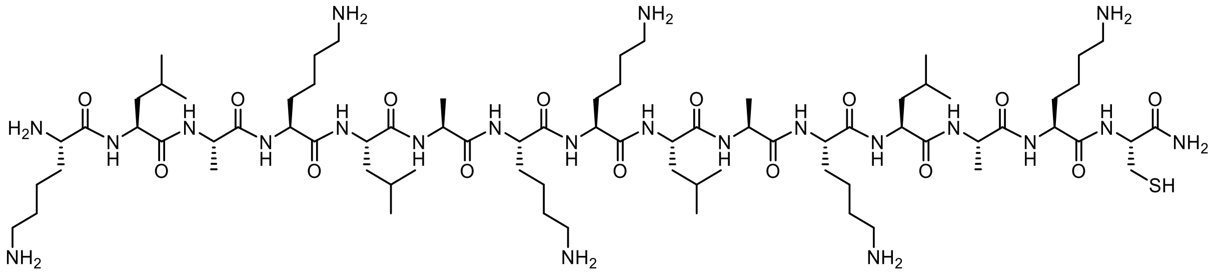

HPLC-UV chromatogram (210 nm) of **4g** by **Method 1**:

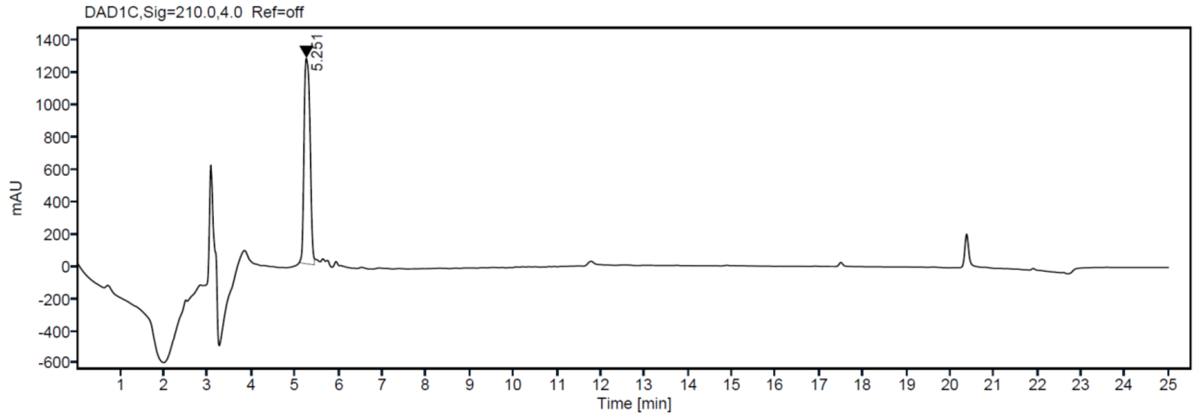

Retention time: 5.292 min **Area Percent:** 100%

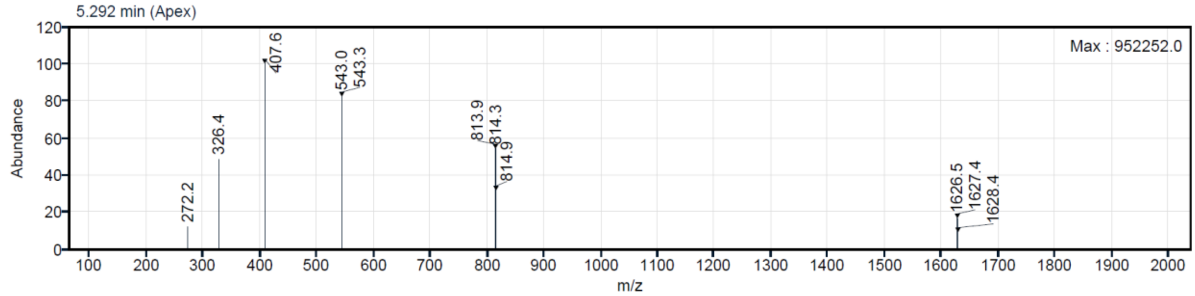

HRMS (nanochip-ESI/LTQ-Orbitrap) m/z: $[M + H_3]^{+3}$ Calcd for $C_{75}H_{147}N_{22}O_{15}S^{+3}$ 542.7040; Found 542.7058.

KLAKLAKKLAKLAKC-NH2 3CzIPN (6ga)

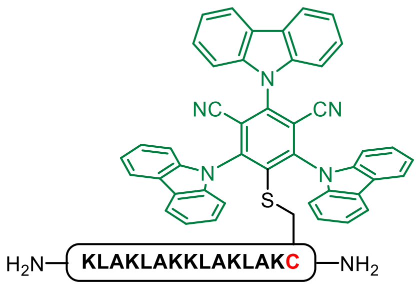

Following the **General procedure**, the reaction was conducted on a 5.0 µmol scale. The desired product **6ga** (5.1 mg, 2.3 µmol, 45% yield) was isolated by **Method 3.**
HPLC-UV chromatogram (210 nm) of the crude by **Method 1**:

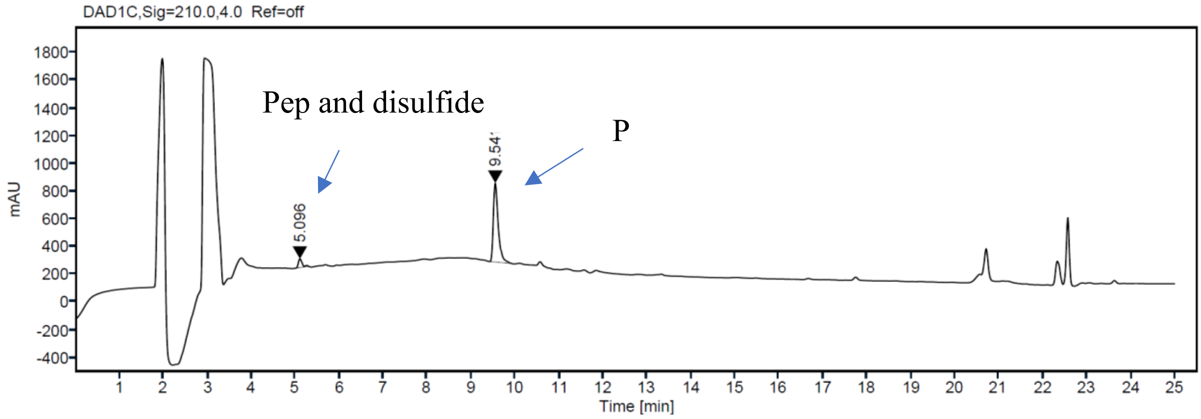

HPLC-UV chromatogram (210 nm) of **6ga** by **Method 1**:

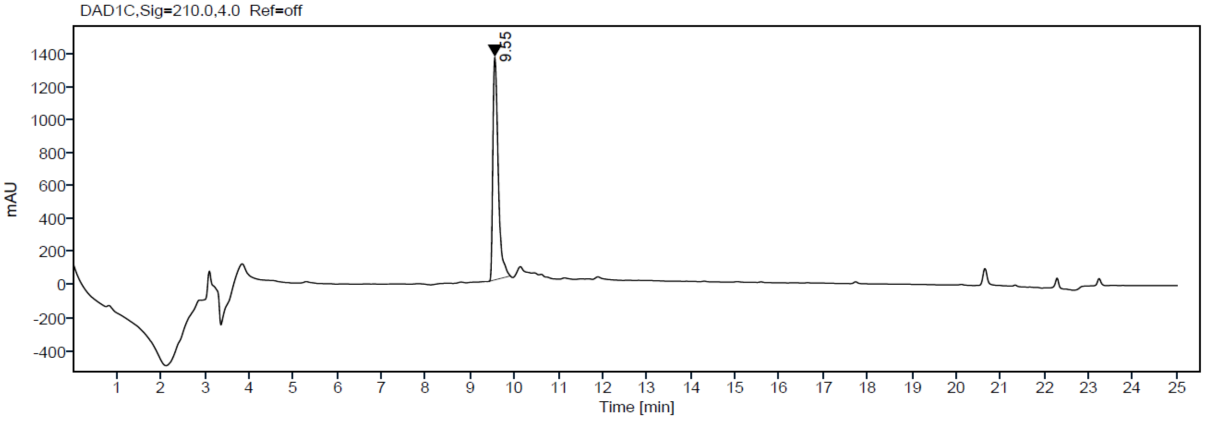

Retention time: 9.586 min **Area Percent:** 100%

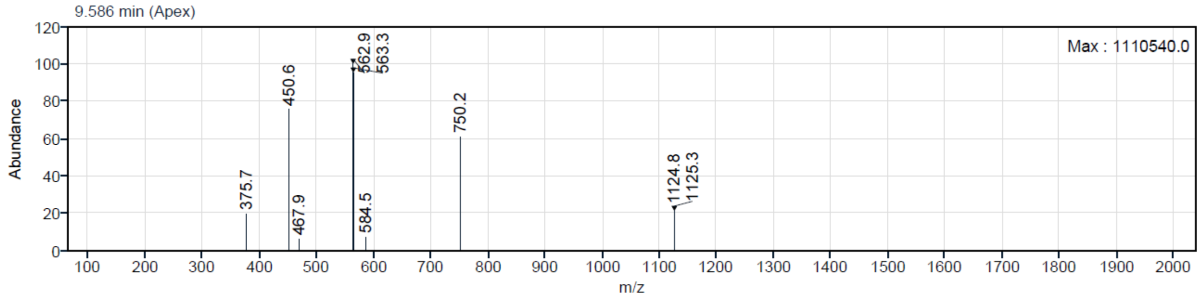

HRMS (nanochip-ESI/LTQ-Orbitrap) m/z: $[M + H_3]^{+3}$ Calcd for $C_{119}H_{170}N_{27}O_{15}S^{+3}$ 749.7691; Found 749.7719.

MS/MS fragmentation of **6ga**:

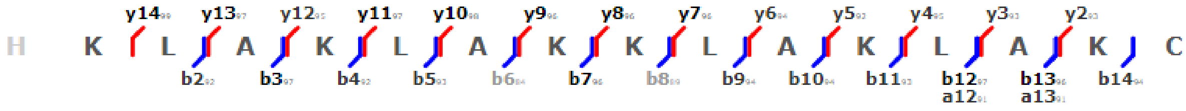

Cter

Cter = C44H25N6

Sequence	Type	MF	MF Mass	m/z	Intensity	Similarity
LAKLAKKLAKLAKC	y14	C113H156N25O14S(+1)	2119.2	1060.1	76.5	99.36%
AKLAKKLAKLAKC	y13	C107H145N24O13S(+1)	2006.1	1003.6	73.44	98.50%
LAKLAKKLAKLAKC	y14	C113H156N25O14S(+1)	2119.2	707.07	24.47	98.40%
KLAKLAKKLAKLAK	b14	C72H137N20O14(+1)	1506.1	502.69	15.24	98.31%
KLAKKLAKLAKC	y12	C104H140N23O12S(+1)	1935.1	968.04	15.67	97.99%
AKKLAKLAKC	y10	C92H117N20O10S(+1)	1693.9	847.45	10.8	97.67%
KLAKLAKKLAKL	b12	C63H120N17O12(+1)	1306.9	653.97	26.76	97.46%
KLA	b3	C15H29N4O3(+1)	313.22	313.22	6.88	97.46%
KLAKLAKKLAK	b11	C57H109N16O11(+1)	1193.8	597.43	29.14	97.34%
LAKKLAKLAKC	y11	C98H128N21O11S(+1)	1807	903.99	24.47	97.14%
LAKLAKC	y7	C77H88N15O7S(+1)	1366.7	1366.7	11.17	96.87%

KLAKLAKKLAKLA	b13	C66H125N18O13(+1)	1378	689.49	64.33	96.39%
KLAKLAKC	y8	C83H100N17O8S(+1)	1494.8	1494.8	3.54	96.25%
AKLAKC	y6	C71H77N14O6S(+1)	1253.6	1253.6	13.38	96.19%
KLAKLAK	b7	C36H69N10O7(+1)	753.54	377.27	4.59	96.12%
KKLAKLAKC	y9	C89H112N19O9S(+1)	1622.9	811.93	3.9	96.11%
KLAKLAK	b7	C36H69N10O7(+1)	753.54	753.53	61.42	95.99%
AKLAKKLAKLAKC	y13	C107H145N24O13S(+1)	2006.1	669.38	5.56	95.75%
KLAKLAKKL	b9	C48H92N13O9(+1)	994.71	994.71	4.86	95.46%
LAKLAKC	y7	C77H88N15O7S(+1)	1366.7	683.84	8.8	95.18%
KLAKLAKKLA	b10	C51H97N14O10(+1)	1065.8	533.38	23.81	94.54%
KLAKC	y5	C68H72N13O5S(+1)	1182.6	1182.5	11.54	94.51%
LAKC	y4	C62H60N11O4S(+1)	1054.5	1054.5	17.46	94.51%
KLAKLAKKLA	b10	C51H97N14O10(+1)	1065.8	1065.8	14.31	93.99%
KLAKLAKK	b8	C42H81N12O8(+1)	881.63	881.63	9.18	93.41%
KLAKL	b5	C27H52N7O5(+1)	554.4	554.4	7.74	93.07%
AKC	y3	C56H49N10O3S(+1)	941.37	941.37	15.99	92.98%
KC	y2	C53H44N9O2S(+1)	870.33	870.33	11.88	92.98%
KLAKLA	b6	C30H57N8O6(+1)	625.44	625.44	26.06	92.92%
KL	b2	C12H24N3O2(+1)	242.19	242.19	1.75	92.50%
KLAKLAKKL	b9	C48H92N13O9(+1)	994.71	497.86	13.63	92.25%
AKLAKC	y6	C71H77N14O6S(+1)	1253.6	627.3	3.2	91.96%
KLAK	b4	C21H41N6O4(+1)	441.32	441.32	25.45	91.51%
KLAKKLAKLAKC	y12	C104H140N23O12S(+1)	1935.1	645.7	1.43	91.14%
KLAKLAKKLAKL	a12	C62H120N17O11(+1)	1278.9	639.97	1.04	90.90%
KLAKLAKKLAKLA	a13	C65H125N18O12(+1)	1350	675.49	1.72	90.58%
KLAKC	y5	C68H72N13O5S(+1)	1182.6	591.78	2.82	88.91%
KLAKLAKKLAKLAK	b14	C72H137N20O14(+1)	1506.1	753.53	61.42	88.73%
KLAKLAKKLAK	b11	C57H109N16O11(+1)	1193.8	1193.8	1.7	88.01%
KLAKLAKK	b8	C42H81N12O8(+1)	881.63	441.32	25.45	84.47%
KLAKLA	b6	C30H57N8O6(+1)	625.44	313.22	6.88	74.62%

PKKKRKVEDPYC-NH$_2$ (4h)

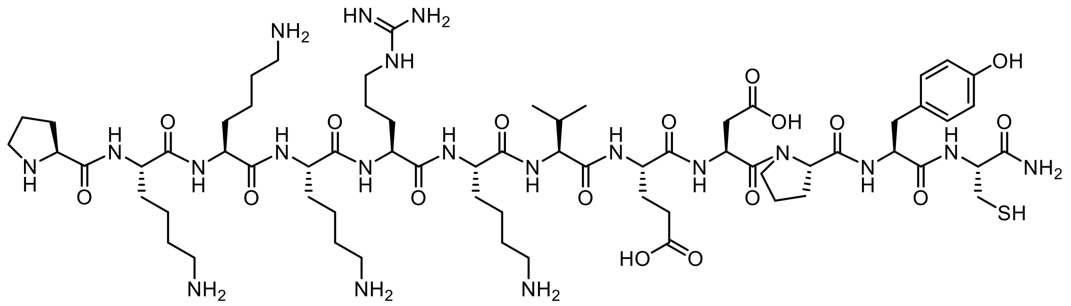

HPLC-UV chromatogram (210 nm) of **4h** by **Method 1**:

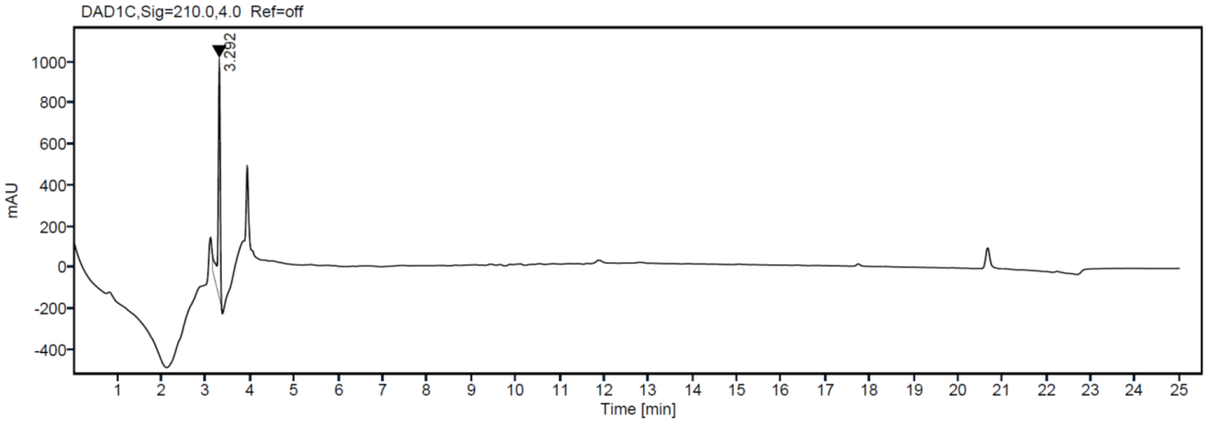

Retention time: 3.318 min **Area Percent:** 100%

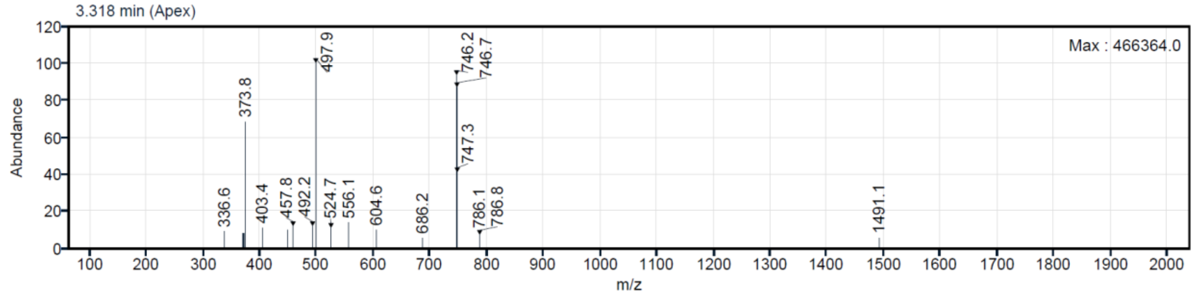

HRMS (nanochip-ESI/LTQ-Orbitrap) m/z: $[M + H_3]^{+3}$ Calcd for $C_{66}H_{115}N_{20}O_{17}S^{+3}$ 497.2818; Found 497.2819.

PKKRKVEDPYC-NH2 3CzIPN (6ha)

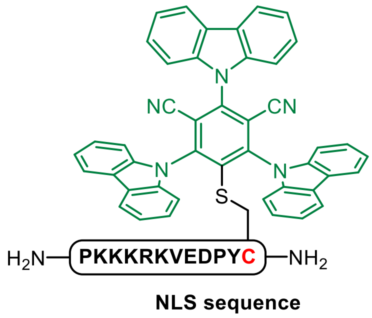

Following the **General procedure**, the reaction was conducted on a 5.0 μmol scale. The desired product **6ha** (5.9 mg, 2.8 μmol, 56% yield) was isolated by **Method 3.**
HPLC-UV chromatogram (210 nm) of the crude by **Method 1**:

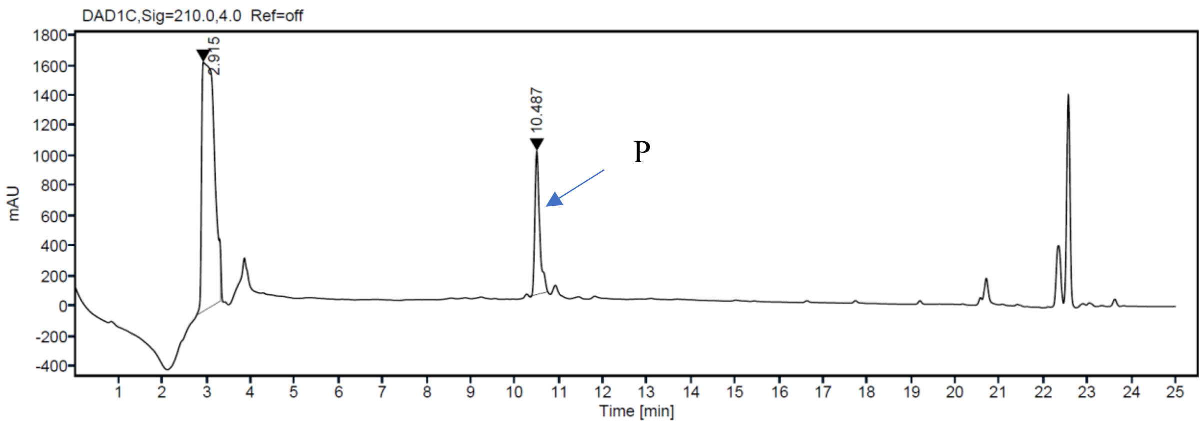

HPLC-UV chromatogram (210 nm) of **6ha** by **Method 1**:

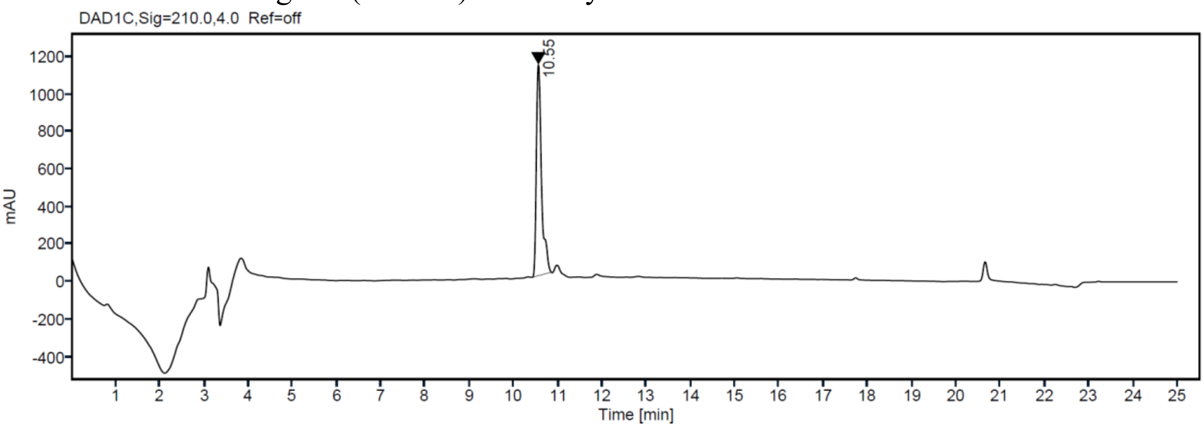

Retention time: 10.606 min **Area Percent:** 100%

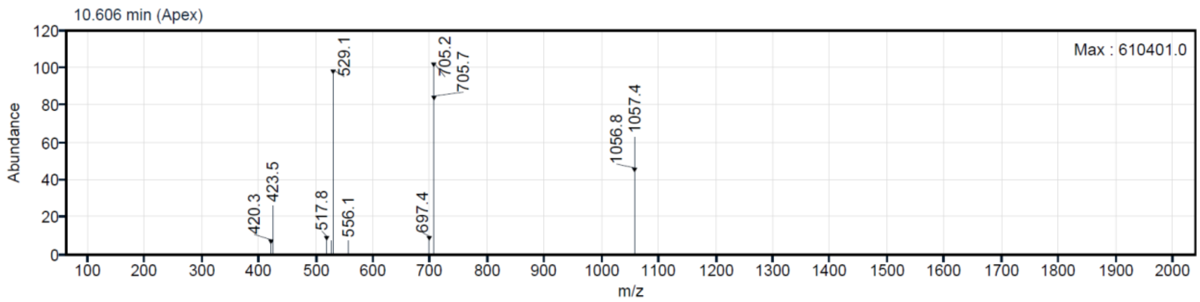

HRMS (nanochip-ESI/LTQ-Orbitrap) m/z: $[M + H_3]^{+3}$ Calcd for $C_{110}H_{138}N_{25}O_{17}S^{+3}$ 704.3469; Found 704.3488.

MS/MS fragmentation of **6ha**:

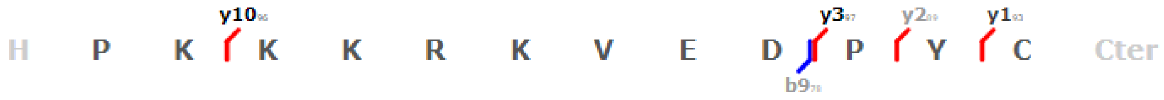

Cter = C44H25N6

Sequence	Type	MF	MF Mass	m/z	Intensity	Similarity
PYC	y3	C61H48N9O4S(+1)	1002.4	1002.4	30.9	97.15%
KKRKVEDPYC	y10	C99H117N22O15S(+1)	1885.9	943.44	1.29	96.47%
C	y1	C47H32N7OS(+1)	742.24	742.24	37.59	93.49%

YC	y2	C56H41N8O3S(+1)	905.3	905.3	1.18	89.31%
PKKKRKVED	b9	C49H89N16O13(+1)	1109.7	555.34	14.55	78.10%

C(RGDfE)C-NH$_2$ (4i)

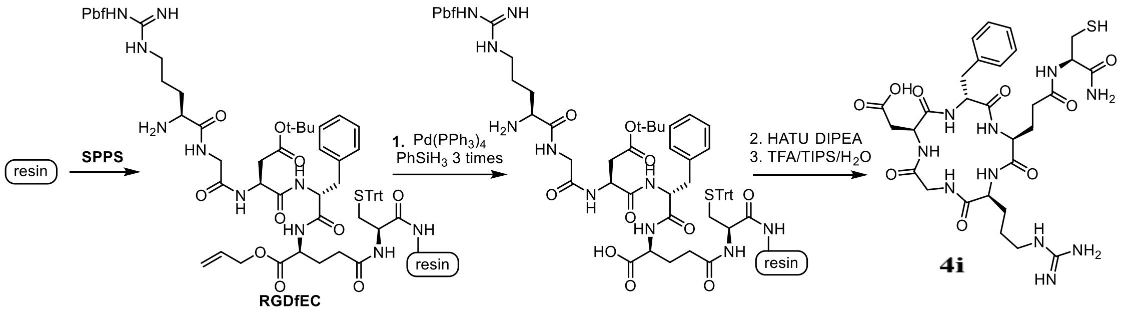

The cyclic peptide **4i** was prepared following a reported procedure[8]: The linear peptide RGDfEP was prepared with general SPPS procedure with Rink amide resin (0.23 g/mmol). The resin with allyl protecting group was mixed with Pd(PPh$_3$)$_4$ (0.1 equiv.) and PhSiH$_3$ (20 equiv.) in DCM under N$_2$ atmosphere and shaken for 1 hour. This step was repeated twice. The on-resin cyclization was conducted by treating the resin with HATU (4 equiv.) and DIPEA (4 equiv.) in DMF (4 mL/0.1 mmol) for 3 hours. The resin was cleaved with standard cleavage condition and purified by RP-HPLC, yielding cyclic peptide **4i**.

HPLC-UV chromatogram (210 nm) of **4i** by **Method 1**:

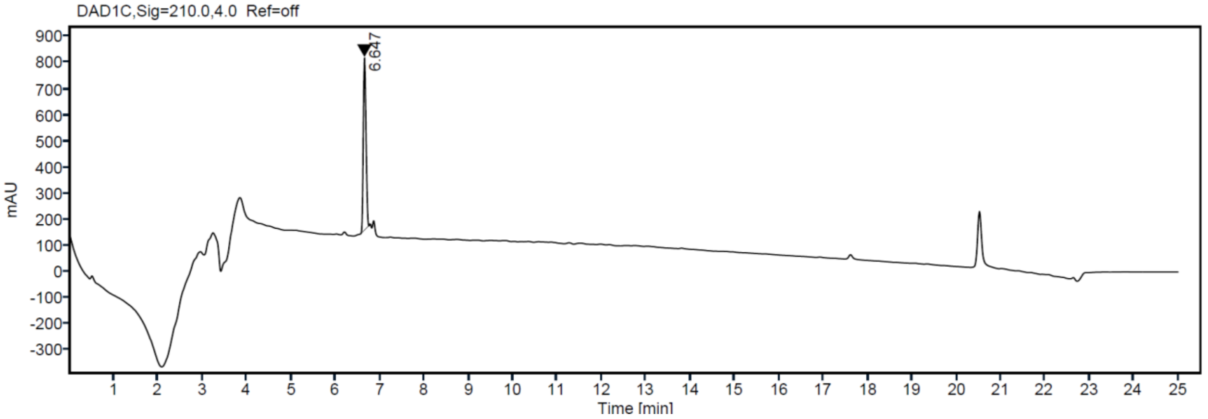

Retention time: 6.667 min **Area Percent:** 100%

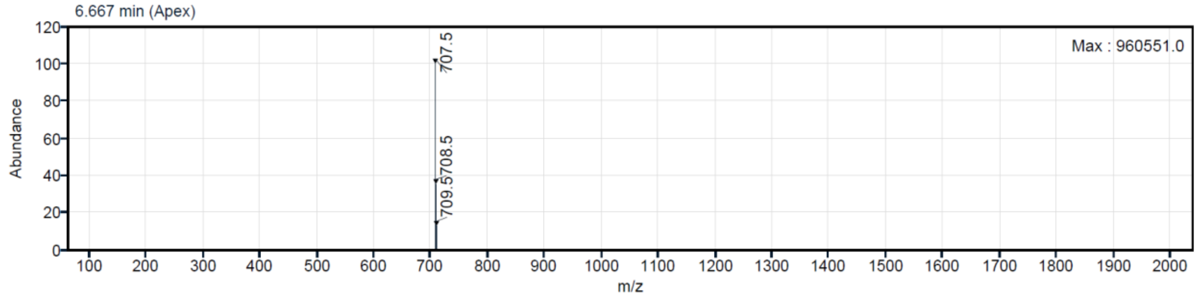

HRMS (ESI/QTOF) m/z: $[M + H]^+$ Calcd for $C_{29}H_{43}N_{10}O_9S^+$ 707.2930; Found 707.2938.

[8] Wu, Y.; Chau, H.-F.; Yeung, Y.-H.; Thor, W.; Kai, H.-Y.; Chan, W.-L.; Wong, K.-L., *Angew. Chem., Int. Ed.* **2022,** *61* (34), e202207532.

C(RGDfE)C-NH2 3CzIPN (6ia)

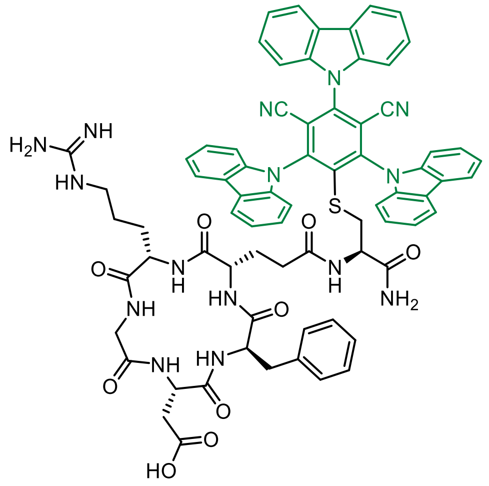

Following the **General procedure**, the reaction was conducted on a 3.8 μmol scale. The desired product **6ia** (3.3 mg, 2.5 μmol, 65% yield) was isolated by **Method 3.**

HPLC-UV chromatogram (210 nm) of the crude by **Method 1**:

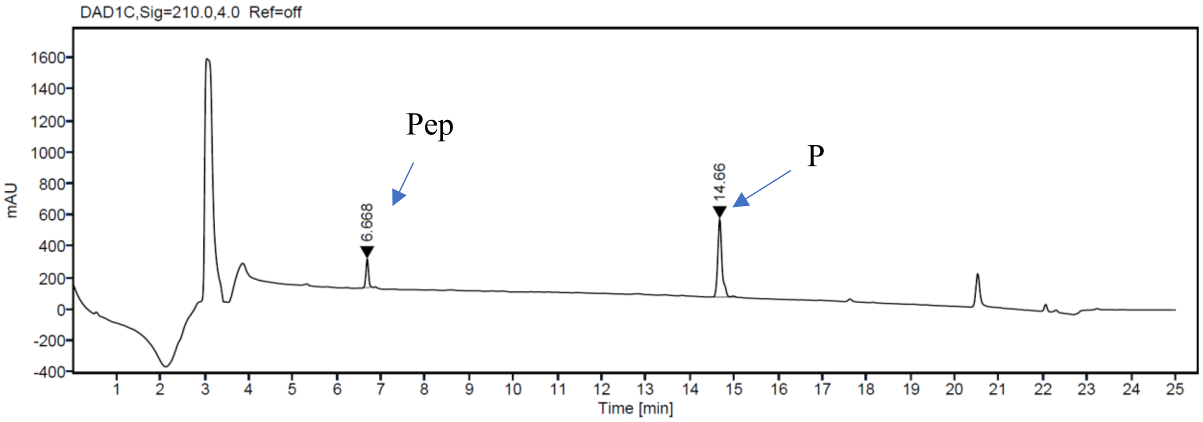

HPLC-UV chromatogram (210 nm) of **6ia** by **Method 1**:

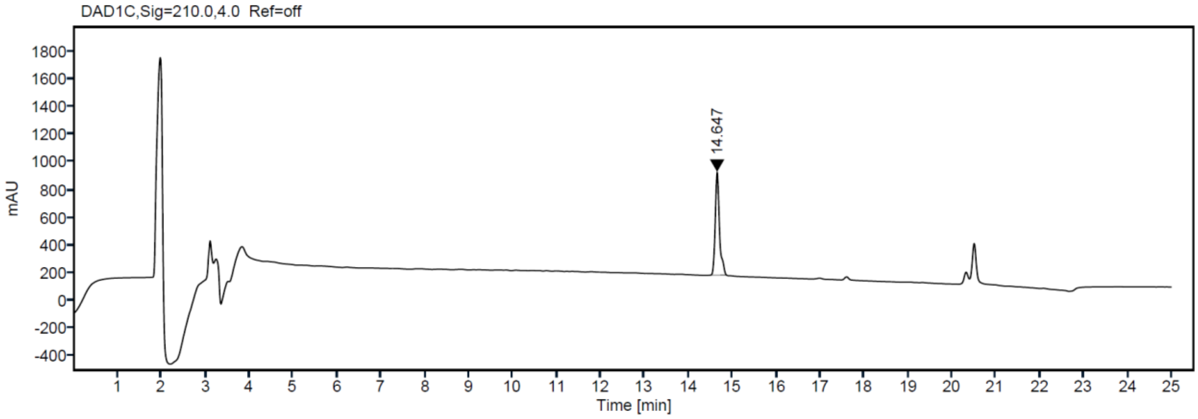

Retention time: 14.694 min **Area Percent:** 100%

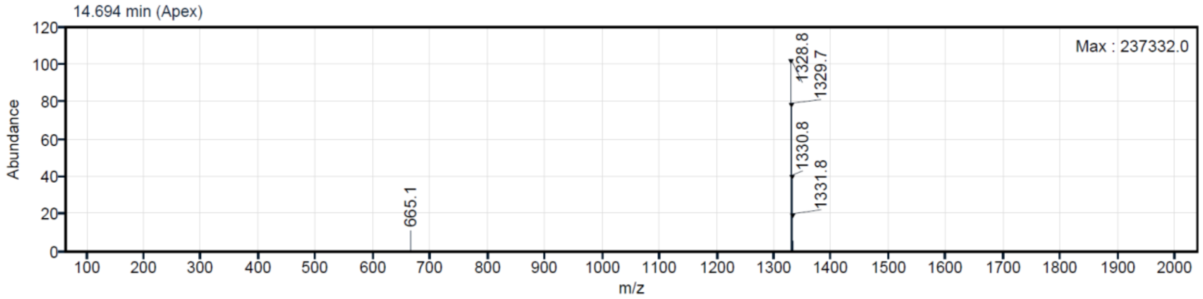

HRMS (Nanochip-based ESI/LTQ-Orbitrap) m/z: $[M + H]^+$ Calcd for $C_{73}H_{66}N_{15}O_9S^+$ 1328.4883; Found 1328.4882.

MS/MS fragmentation of **6ia**:

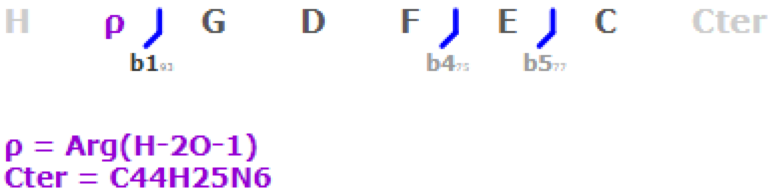

Sequence	Type	MF	MF Mass	m/z	Intensity	Similarity
R	b1	C6H11N4(+1)	139.0984	139.0978	1.29	92.63%
RGDFE	b5	C26H35N8O8(+1)	587.2578	587.2572	6.56	77.48%
RGDF	b4	C21H28N7O5(+1)	458.2152	458.2146	1.8	75.02%

GRGDSPC-NH$_2$ (4j)

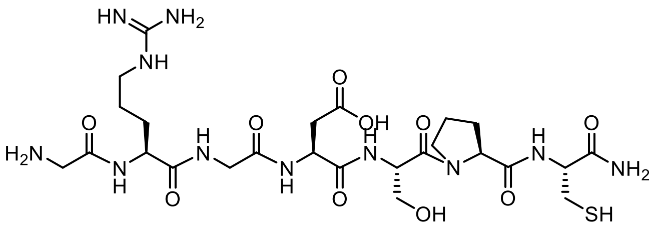

HPLC-UV chromatogram (210 nm) of **4j** by **Method 1**:

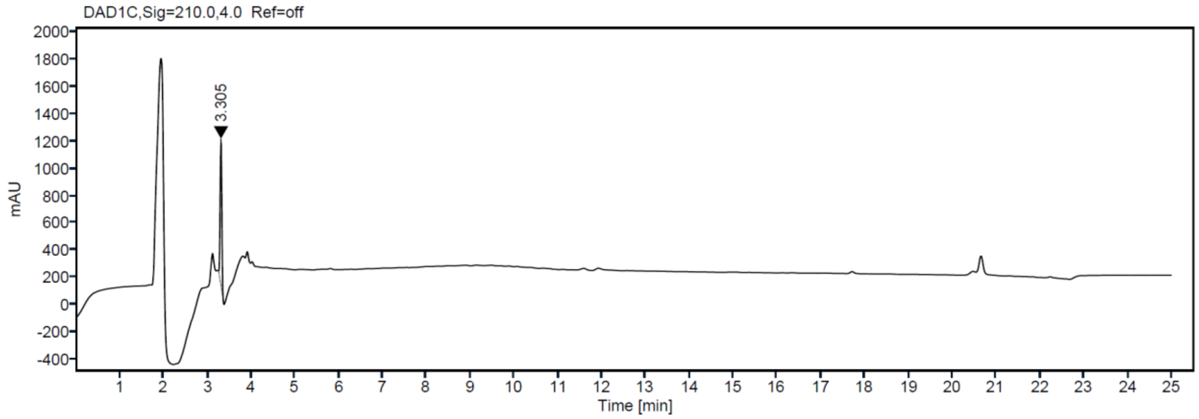

Retention time: 3.338 min **Area Percent:** 100%

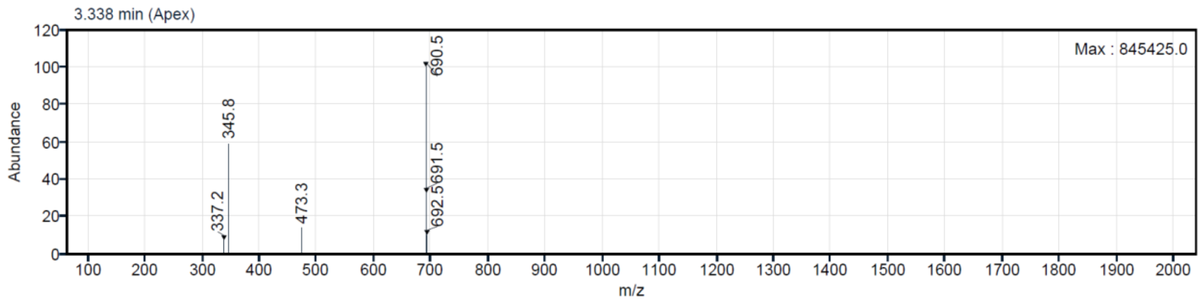

HRMS (ESI/QTOF) m/z: $[M + H]^+$ Calcd for $C_{25}H_{44}N_{11}O_{10}S^+$ 690.2988; Found 690.2996.

GRGDSC-NH2 6ja

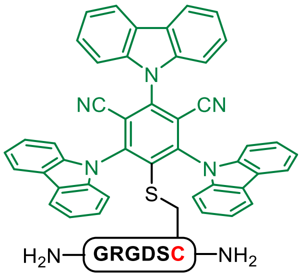

Following the **General procedure**, the reaction was conducted on a 3.8 μmol scale. The desired product **6ja** (3.3 mg, 2.5 μmol, 65% yield) was isolated by **Method 3.**

HPLC-UV chromatogram (210 nm) of the crude by **Method 1**:

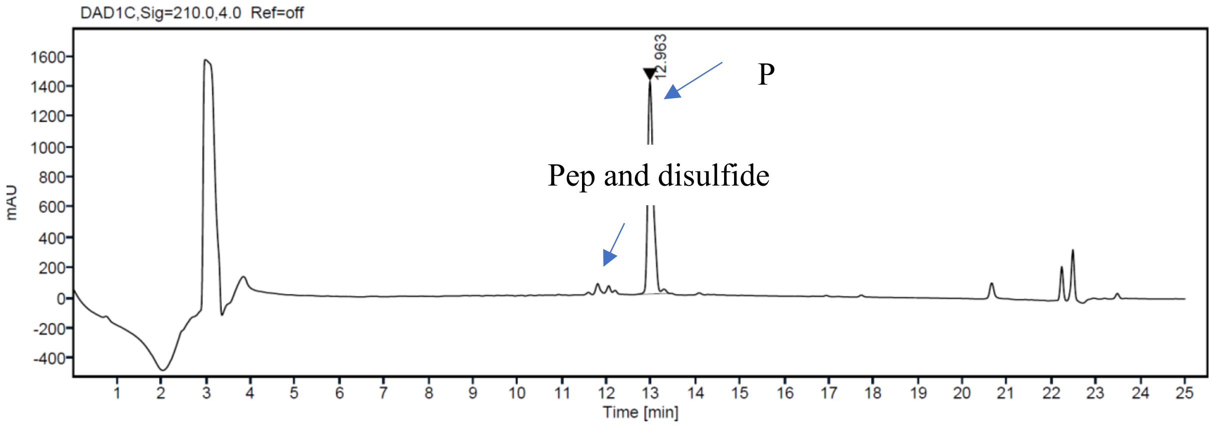

HPLC-UV chromatogram (210 nm) of **6ja** by **Method 1**:

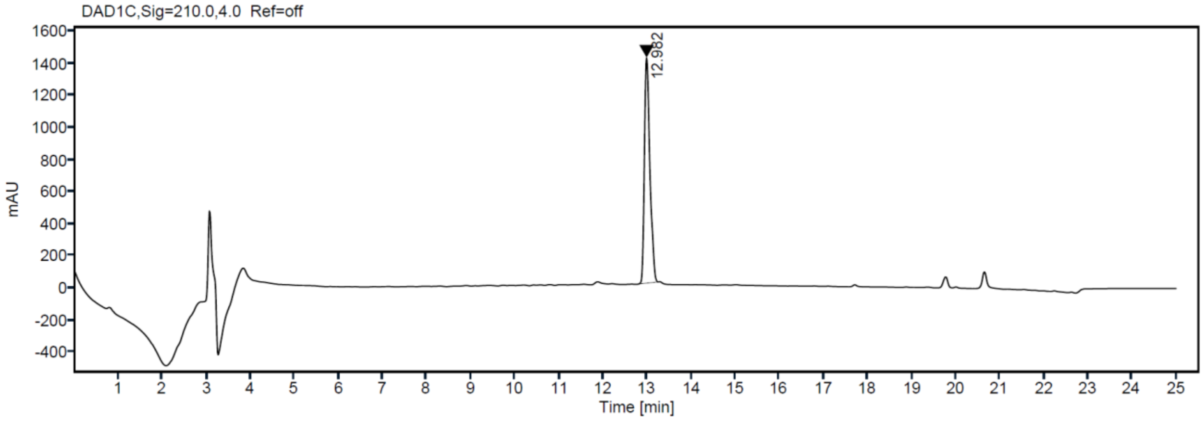

Retention time: 13.017 min **Area Percent:** 100%

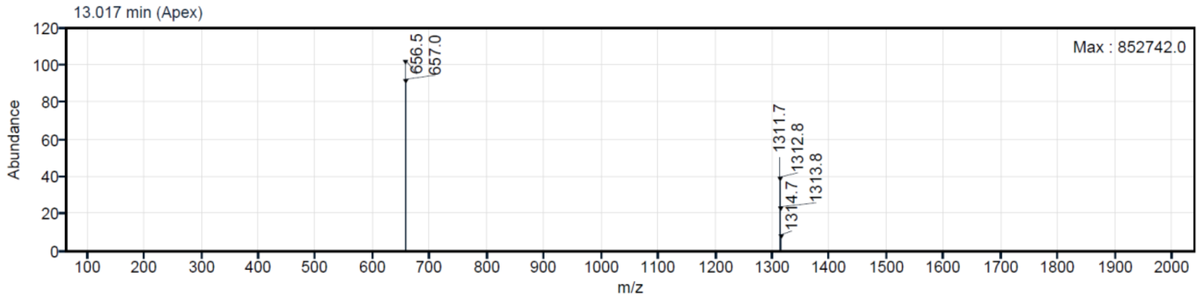

HRMS (nanochip-ESI/LTQ-Orbitrap) m/z: $[M + H_2]^{+2}$ Calcd for $C_{69}H_{68}N_{16}O_{10}S^{+2}$ 656.2507; Found 656.2506.

MS/MS fragmentation of **6ja**:

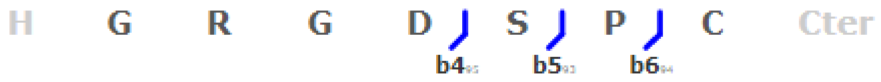

Cter = C44H23N5O

Sequence	Type	MF	MF Mass	m/z	Intensity	Similarity
GRGD	b4	C14H24N7O6(+1)	386.1788	386.1783	9.18	94.97%
GRGDSP	b6	C22H36N9O9(+1)	570.2636	570.2631	10.24	93.91%
GRGDS	b5	C17H29N8O8(+1)	473.2108	473.2103	39.73	93.16%

AcNH-GCRPKPQQFFGLM-NH$_2$ (4k)

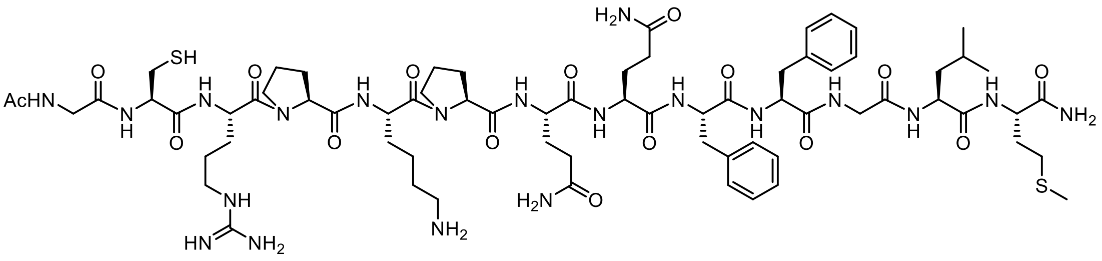

HPLC-UV chromatogram (210 nm) of **4k** by **Method 1**:

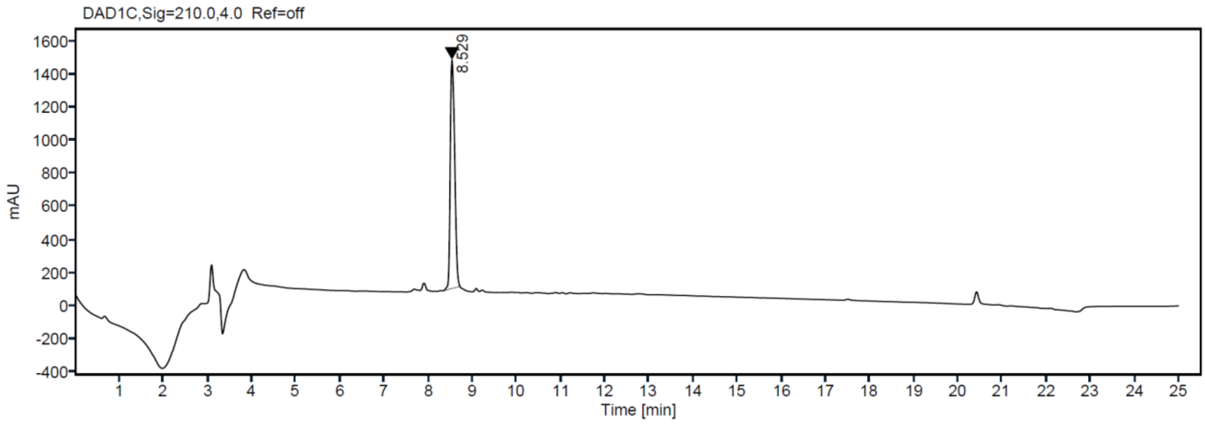

Retention time: 8.602 min **Area Percent:** 100%

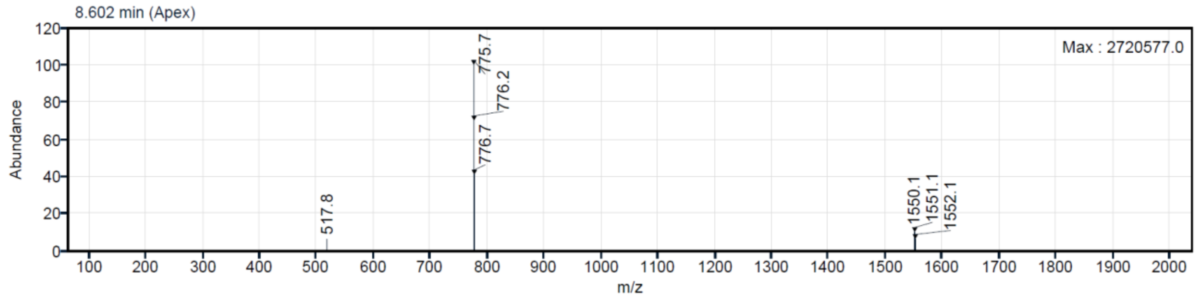

HRMS (Nanochip-based ESI/LTQ-Orbitrap) m/z: $[M + H_2]^{+2}$ Calcd for $C_{70}H_{110}N_{20}O_{16}S_2^{+2}$ 775.3920; Found 775.3925.

AcNH-GCRPKPQQFFGLM-NH2 3CzIPN (6ka)

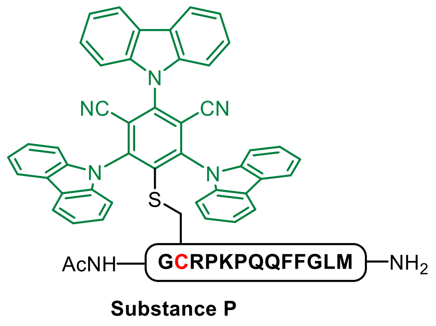

Following the **General procedure**, the reaction was conducted on a 3.0 μmol scale. The desired product **6ka** (3.7 mg, 1.7 μmol, 57% yield) was isolated by **Method 3.**
HPLC-UV chromatogram (210 nm) of the crude by **Method 1**:

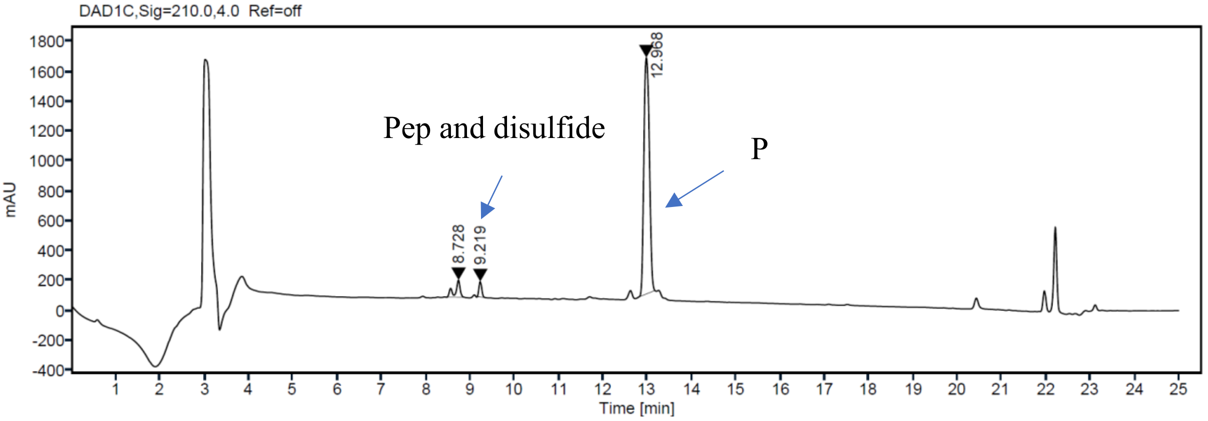

HPLC-UV chromatogram (210 nm) of **6ka** by **Method 1**:

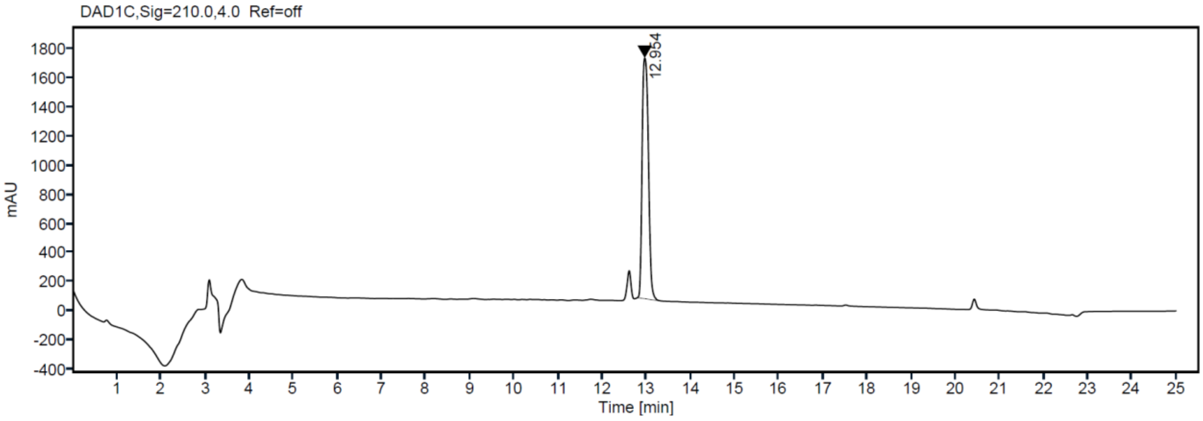

Retention time: 13.066 min **Area Percent:** 100%

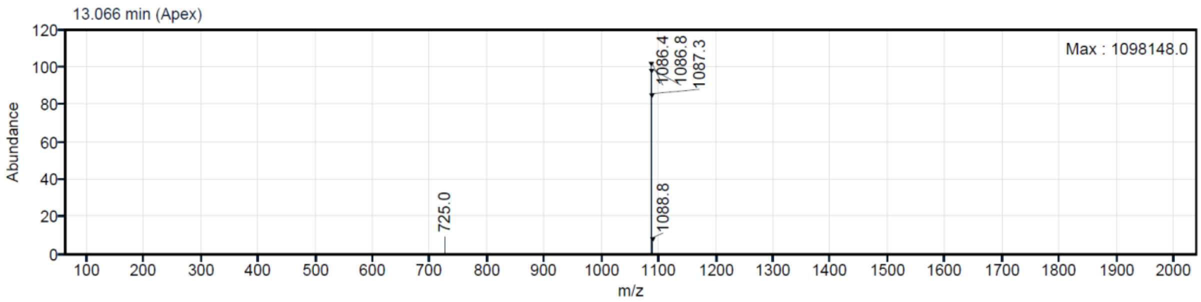

HRMS (Nanochip-based ESI/LTQ-Orbitrap) m/z: $[M + H_2]^{+2}$ Calcd for $C_{114}H_{133}N_{25}O_{16}S_2^{+2}$ 1085.9896; Found 1085.9946.
MS/MS fragmentation of **6ka**:

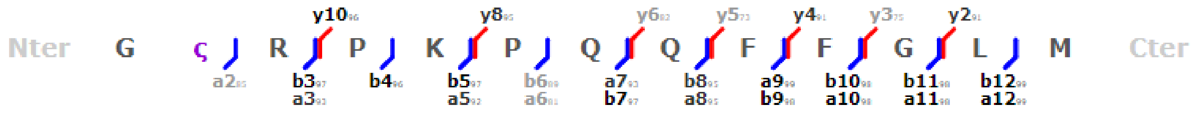

ç = Cys(C44H23N5)
Nter = C2H3O
Cter = NH2

Sequence	Type	MF	MF Mass	m/z	Intensity	Similarity
GCRPKPQQFFGLM		C114H131N25O16S2	2169.965	1085.99	40.7	99.36%
GCRPKPQQFFGL	b12	C109H120N23O15S(+1)	2022.906	1011.956	32.98	99.22%
GCRPKPQQFFGL	a12	C108H120N23O14S(+1)	1994.911	1994.91	12.93	99.21%
GCRPKPQQFFGL	a12	C108H120N23O14S(+1)	1994.911	997.9587	76.09	99.17%
GCRPKPQQF	a9	C91H97N20O11S(+1)	1677.737	1677.736	20.8	99.14%
GCRPKPQQF	b9	C92H97N20O12S(+1)	1705.732	1705.731	23.35	98.99%
GCRPKPQQFF	b10	C101H106N21O13S(+1)	1852.8	1852.799	11.37	98.93%
GCRPKPQQFFG	b11	C103H109N22O14S(+1)	1909.821	1909.821	22.22	98.51%
GCRPKPQ	a7	C77H80N17O8S(+1)	1402.61	1402.609	9.98	98.40%
GCRPKPQQFF	a10	C100H106N21O12S(+1)	1824.805	912.9059	21.53	98.38%
GCRPKPQQFF	a10	C100H106N21O12S(+1)	1824.805	1824.805	10.62	98.30%
GCRPKPQQFFGL	b12	C109H120N23O15S(+1)	2022.906	2022.905	16.44	98.28%
GCRPKPQQF	a9	C91H97N20O11S(+1)	1677.737	839.3717	33.42	98.17%
GCRPKPQQFFG	a11	C102H109N22O13S(+1)	1881.827	1881.826	3.87	98.11%
GCRPKPQ	b7	C78H80N17O9S(+1)	1430.605	1430.604	30.47	98.06%
GCRPK	b5	C68H65N14O6S(+1)	1205.493	1205.493	82.78	97.79%

GCRPKPQQFFG	b11	C103H109N22O14S(+1)	1909.821	955.4141	27.43	97.75%
GCRPKPQQ	b8	C83H88N19O11S(+1)	1558.663	1558.663	34.78	97.51%
GCRPKPQQFFG	a11	C102H109N22O13S(+1)	1881.827	941.4166	10.36	97.38%
GCR	b3	C57H46N11O4S(+1)	980.3455	980.3449	90.07	97.17%
GCRPKPQQF	b9	C92H97N20O12S(+1)	1705.732	853.3691	20.67	96.82%
GCRPKPQQFF	b10	C101H106N21O13S(+1)	1852.8	926.9033	14.4	96.60%
PKPQQFFGLM	y10	C57H87N14O12S(+1)	1191.635	1191.634	6.14	96.37%
GCRP	b4	C62H53N12O5S(+1)	1077.398	1077.398	7.44	95.69%
GCRPK	b5	C68H65N14O6S(+1)	1205.493	603.25	1.51	95.34%
GCRPKPQ	b7	C78H80N17O9S(+1)	1430.605	715.8056	5.04	95.32%
PQQFFGLM	y8	C46H68N11O10S(+1)	966.4871	966.4866	9.88	94.93%
GCRPKPQQ	a8	C82H88N19O10S(+1)	1530.668	1530.668	11.38	94.91%
GCR	a3	C56H46N11O3S(+1)	952.3506	952.35	10.51	93.21%
GCRPKPQQ	b8	C83H88N19O11S(+1)	1558.663	779.8349	10.19	91.75%
GCRPK	a5	C67H65N14O5S(+1)	1177.498	1177.498	2.52	91.55%
LM	y2	C11H24N3O2S(+1)	262.1589	262.1584	6.84	91.18%
FGLM	y4	C22H36N5O4S(+1)	466.2488	466.2483	2.67	90.52%
GCRPKP	b6	C73H72N15O7S(+1)	1302.546	1302.545	2.49	88.68%
GCRPKPQ	a7	C77H80N17O8S(+1)	1402.61	701.8082	2.08	87.08%
GC	a2	C50H34N7O2S(+1)	796.2495	796.2489	1.26	85.31%
QFFGLM	y6	C36H53N8O7S(+1)	741.3758	741.3752	1.83	82.43%
GCRPKP	a6	C72H72N15O6S(+1)	1274.551	1274.551	5.55	81.27%
GLM	y3	C13H27N4O3S(+1)	319.1804	319.1798	1.9	75.08%
FFGLM	y5	C31H45N6O5S(+1)	613.3172	613.3167	1.9	73.38%

NH_2-ENPEGILDCHVQRVM-NH_2 (4l)

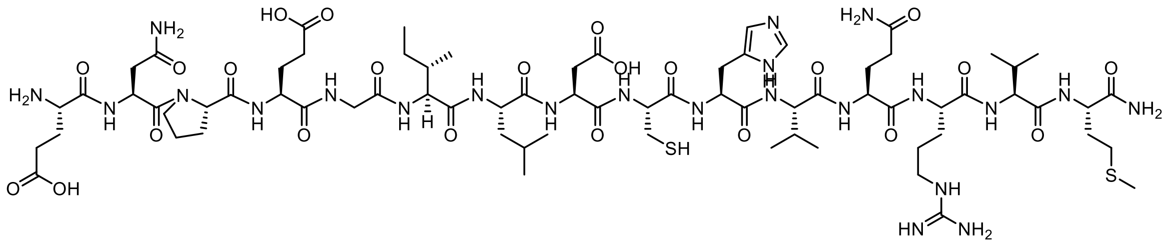

HPLC-UV chromatogram (210 nm) of **4l** by **Method 1**:

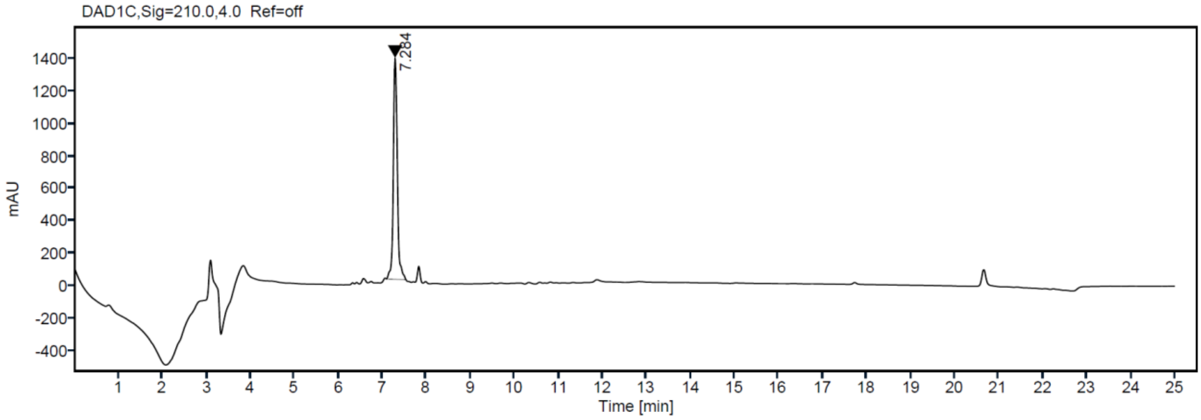

Retention time: 7.367 min **Area Percent:** 100%

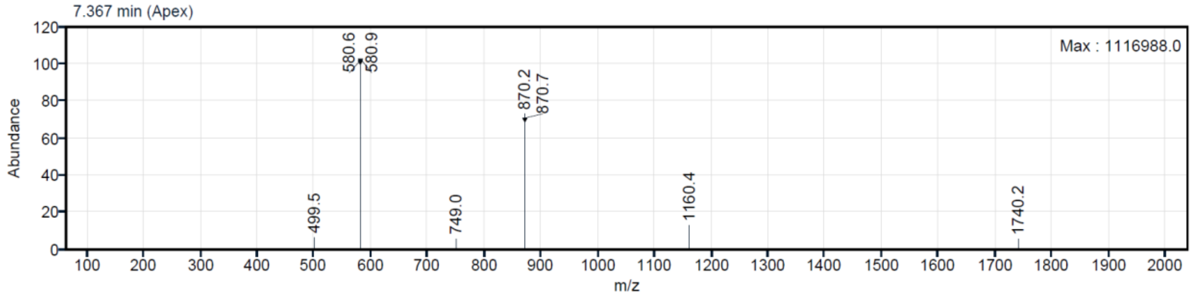

HRMS (Nanochip-based ESI/LTQ-Orbitrap) m/z: $[M + H_3]^{+3}$ Calcd for $C_{72}H_{122}N_{23}O_{23}S_2^{+3}$ 580.2836; Found 580.2839.

ENPEGILDCHVQRVM-NH₂ 3CzIPN (6la)

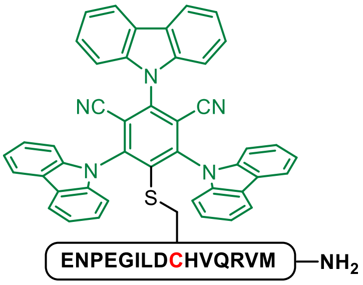

Following the **General procedure**, the reaction was conducted on a 4.0 μmol scale. The desired product **6la** (4.7 mg, 2.0 μmol, 49% yield) was isolated by **Method 3.**

HPLC-UV chromatogram (210 nm) of the crude by **Method 1**:

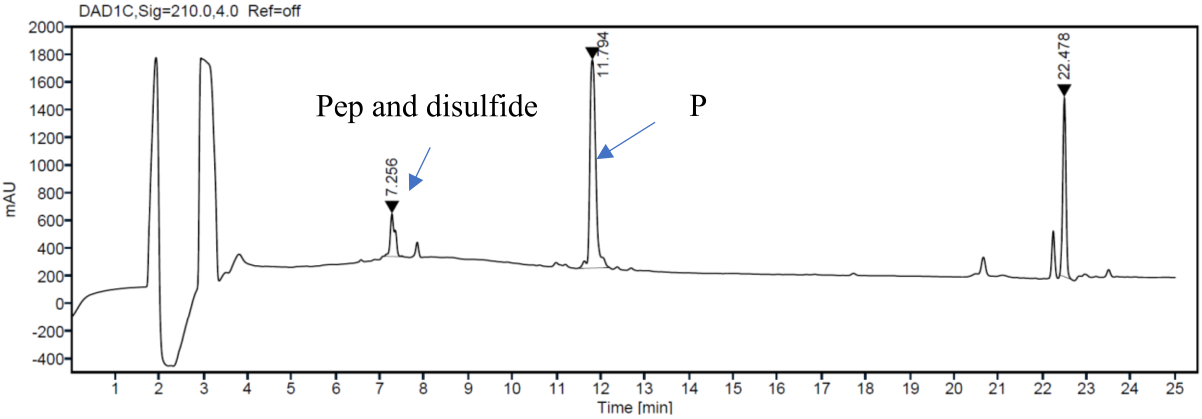

HPLC-UV chromatogram (210 nm) of **6la** by **Method 1**:

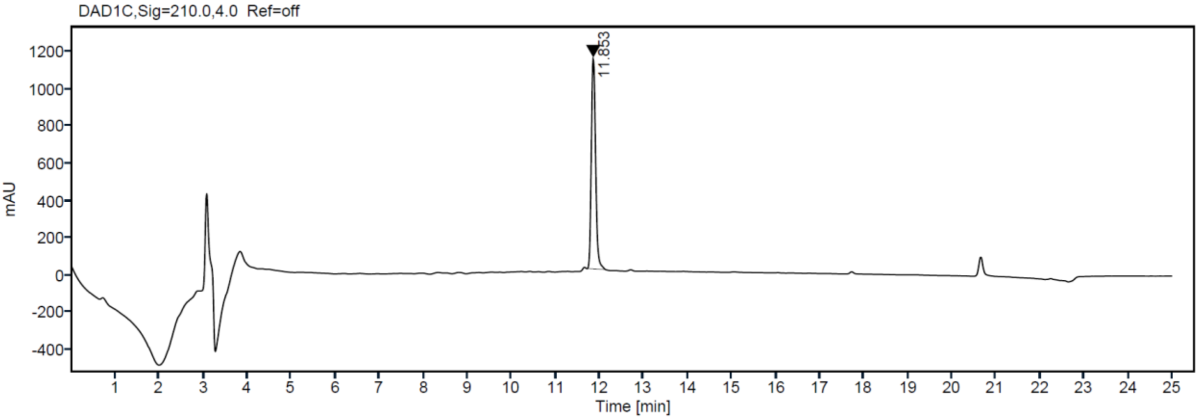

Retention time: 11.895 min **Area Percent:** 100%

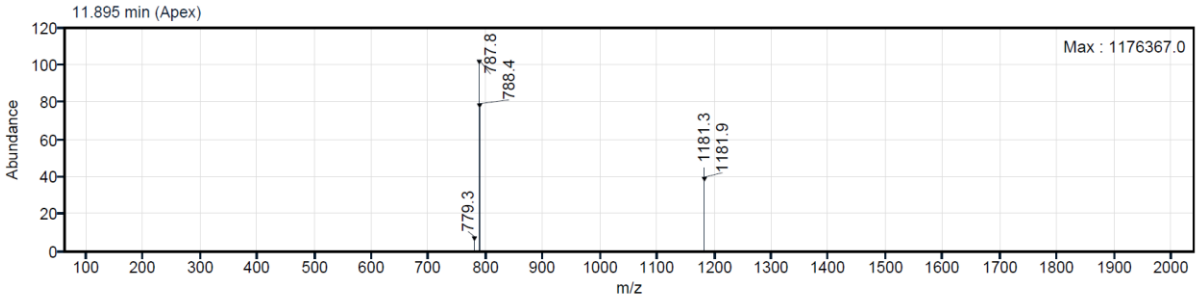

HRMS (Nanochip-based ESI/LTQ-Orbitrap) m/z: $[M + H_3]^{+3}$ Calcd for $C_{116}H_{145}N_{28}O_{23}S_2^{+3}$ 787.3487; Found 787.3497.

MS/MS fragmentation of **6la**:

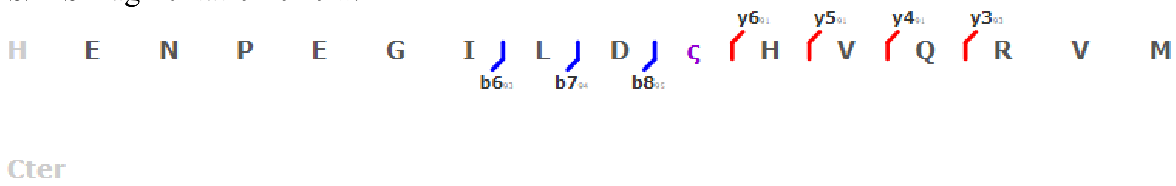

ç = Cys(C44H22N5)
Cter = NH2

Sequence	Type	MF	MF Mass	m/z	Intensity	Similarity
ENPEGILD	b8	C37H58N9O15(+1)	868.41	868.4	3.48	94.85%
ENPEGIL	b7	C33H53N8O12(+1)	753.38	753.38	3.4	94.35%
RVM	y3	C16H34N7O3S(+1)	404.24	404.24	2.4	93.26%
ENPEGI	b6	C27H42N7O11(+1)	640.29	640.29	5.74	92.69%
QRVM	y4	C21H42N9O5S(+1)	532.3	532.3	9.98	91.16%
VQRVM	y5	C26H51N10O6S(+1)	631.37	631.37	7.06	91.10%

RRWPRCILDKHVRRVWR-NH₂ (4n)

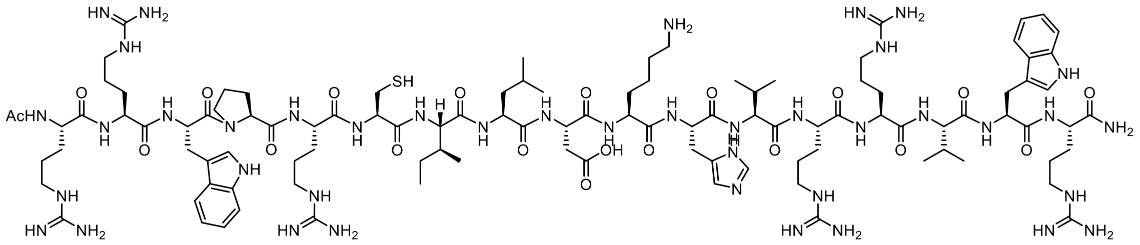

HPLC-UV chromatogram (210 nm) of **4n** by **Method 1**:

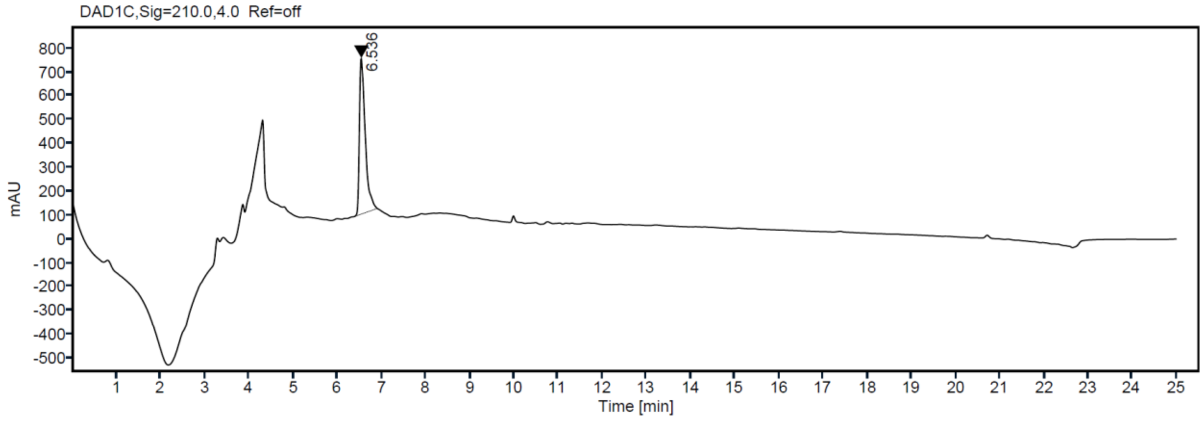

Retention time: 6.561 min **Area Percent:** 100%

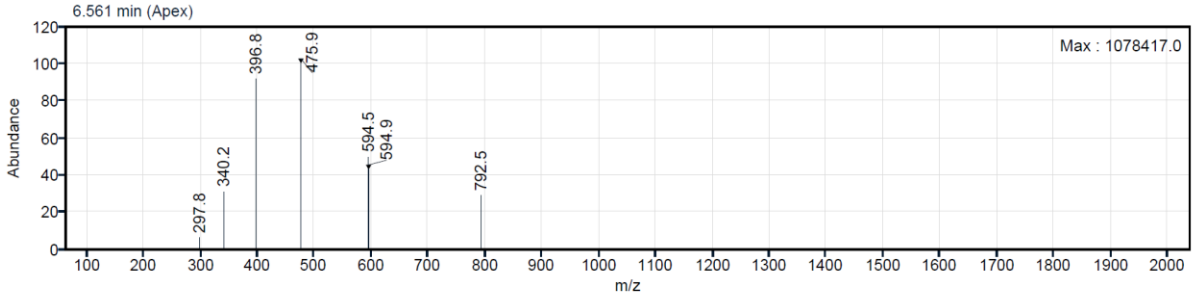

HRMS (nanochip-ESI/LTQ-Orbitrap) m/z: $[M + H_4]^{+4}$ Calcd for $C_{106}H_{178}N_{41}O_{20}S^{+4}$ 594.3454; Found 594.3465.

RRWPRCILDKHVRRVWR-NH₂ 3CzIPN (6na)

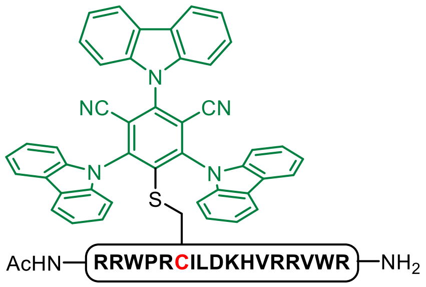

Following the **General procedure**, the reaction was conducted on a 3.5 µmol scale. The desired product **6na** (4.5 mg, 1.5 µmol, 43% yield) was isolated by **Method 3.**
HPLC-UV chromatogram (210 nm) of the crude by **Method 1**:

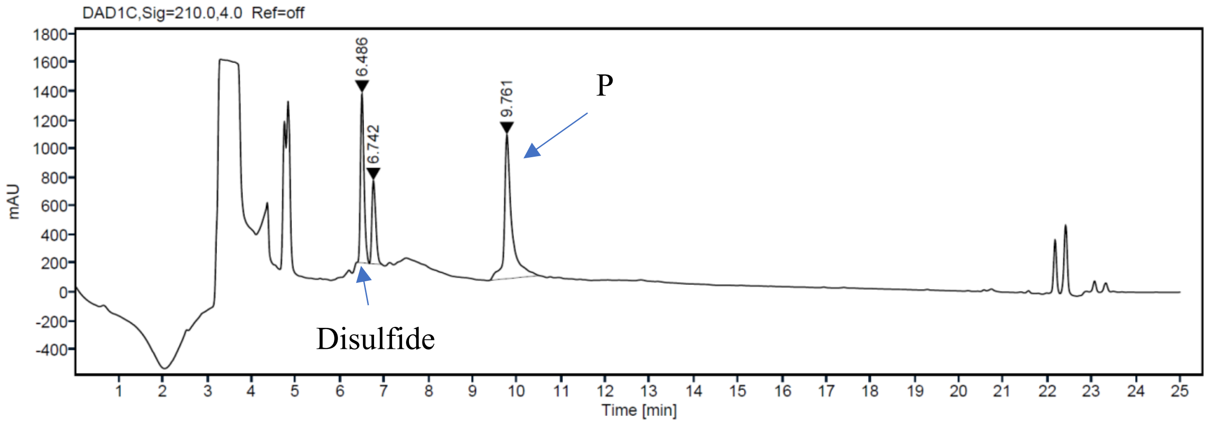

HPLC-UV chromatogram (210 nm) of **6na** by **Method 1**:

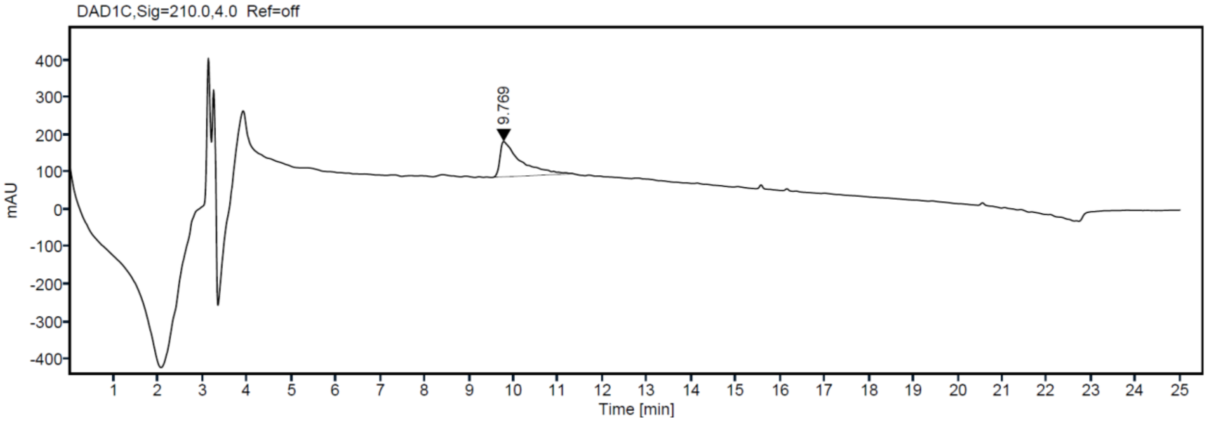

Retention time: 9.77 min **Area Percent:** 100%

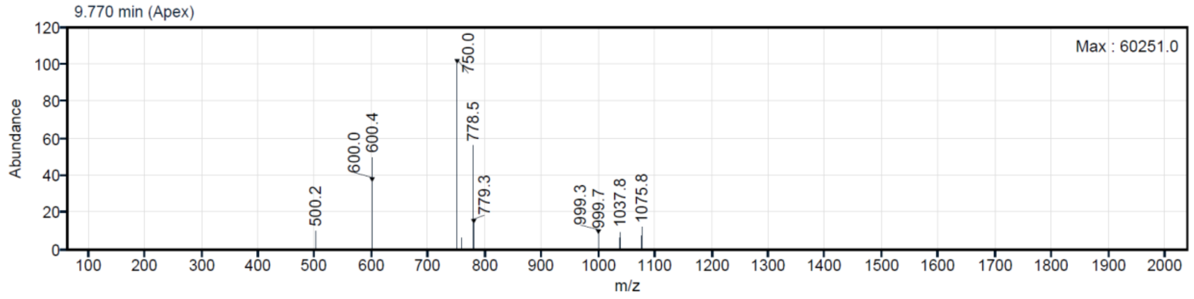

HRMS (nanochip-ESI/LTQ-Orbitrap) m/z: $[M + H_5]^{+5}$ Calcd for $C_{150}H_{201}N_{46}O_{20}S^{+5}$ 599.7164; Found 599.7178.

MS/MS fragmentation of **6na**:

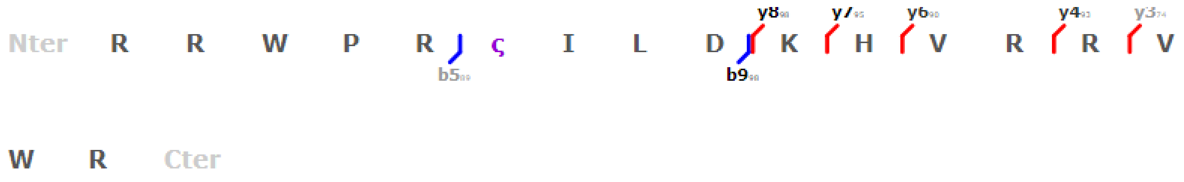

ç = Cys(C44H23N5)
Nter = C2H3O
Cter = NH2

Sequence	Type	MF	MF Mass	m/z	Intensity	Similarity
KHVRRVWR	y8	C51H87N22O8(+1)	1135.7	568.36	7.16	98.69%
HVRRVWR	y7	C45H75N20O7(+1)	1007.6	336.54	2.36	98.41%
KHVRRVWR	y8	C51H87N22O8(+1)	1135.7	379.24	103.66	98.27%
RRWPRCILD	b9	C99H111N24O12S(+1)	1859.9	620.62	10.95	98.02%
RRWPRCILD	b9	C99H111N24O12S(+1)	1859.9	930.43	14.48	97.68%
RVWR	y4	C28H47N12O4(+1)	615.38	308.2	1.45	93.30%
HVRRVWR	y7	C45H75N20O7(+1)	1007.6	504.31	2.07	91.70%
VRRVWR	y6	C39H68N17O6(+1)	870.55	435.78	1.22	90.49%
RRWPR	b5	C36H56N15O6(+1)	794.45	397.73	1.73	88.55%
VWR	y3	C22H35N8O3(+1)	459.28	459.28	2.68	73.78%

HVQRVM	y6	C32H58N13O7S(+1)	768.43	768.43	8.56	91.07%

QSQQTFCNLWRLLKQN-NH₂ (4m)

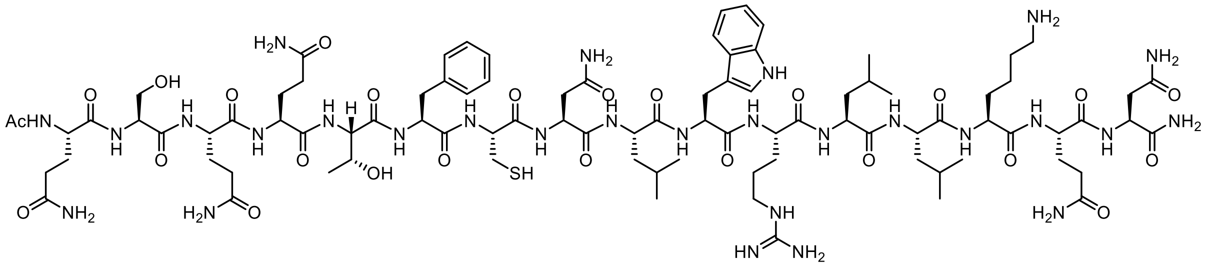

HPLC-UV chromatogram (210 nm) of **4m** by **Method 1**:

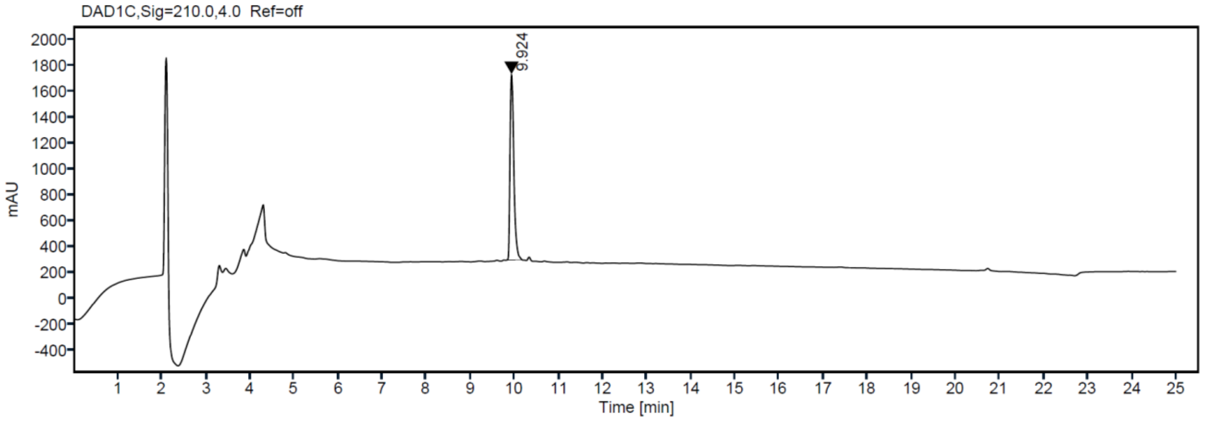

Retention time: 9.93 min **Area Percent:** 100%

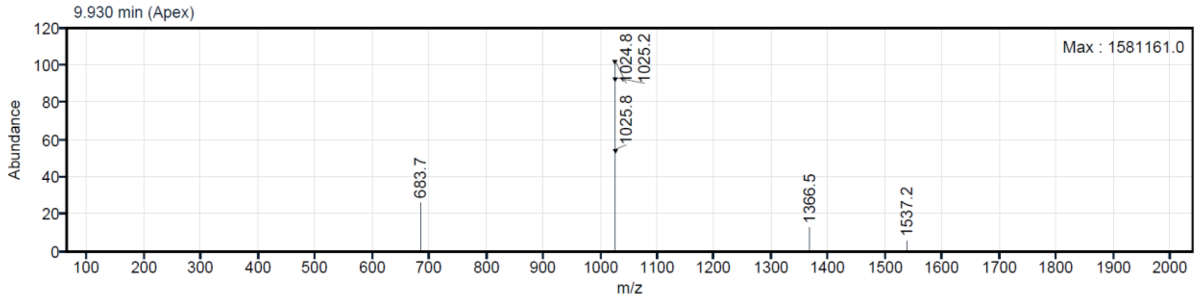

HRMS (nanochip-ESI/LTQ-Orbitrap) m/z: $[M + H_2]^{+2}$ Calcd for $C_{90}H_{142}N_{28}O_{25}S^{+5}$ 1025.0305; Found 1025.0311.

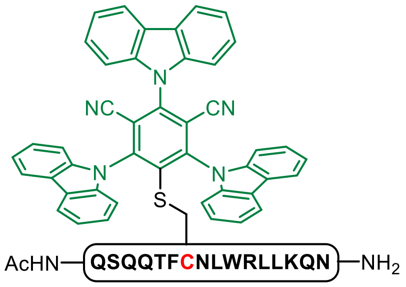

MDM2 binder

Following the **General procedure**, the reaction was conducted on a 3.0 μmol scale. The desired product **6ma** (3.2 mg, 1.2 μmol, 40% yield) was isolated by **Method 3.**

HPLC-UV chromatogram (210 nm) of the crude by **Method 1**:

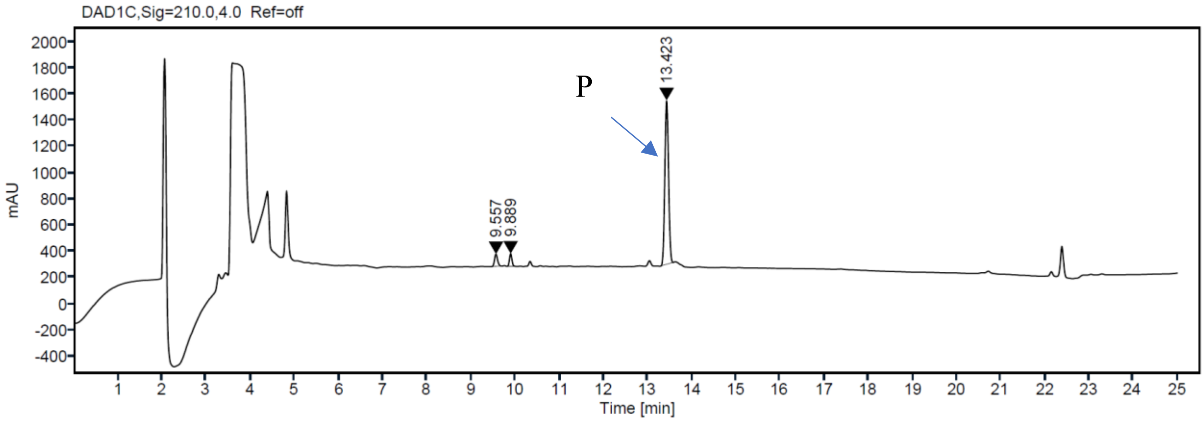

HPLC-UV chromatogram (210 nm) of **6ma** by **Method 1**:

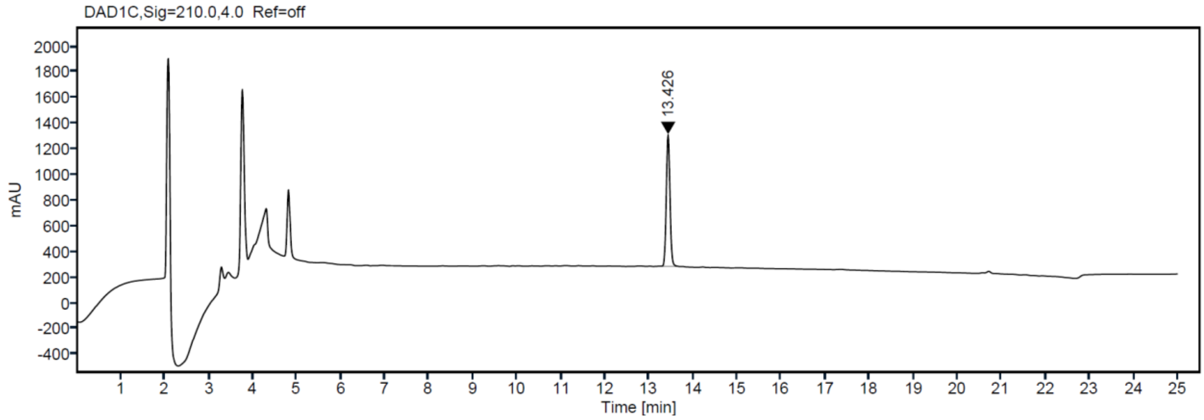

Retention time: 13.443 min **Area Percent:** 100%

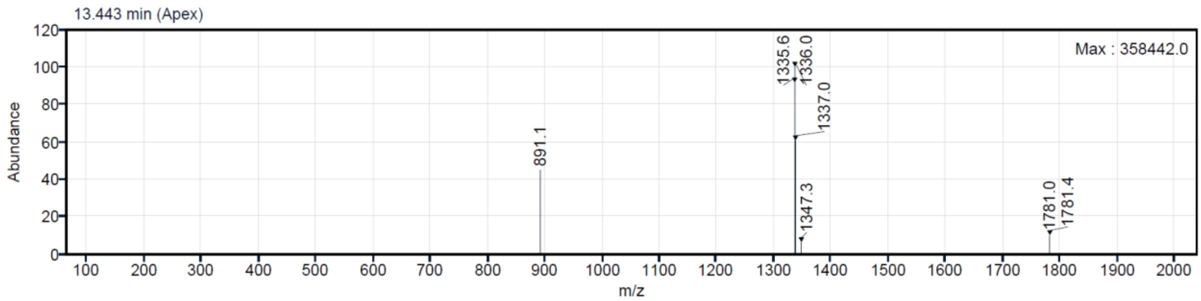

HRMS (nanochip-ESI/LTQ-Orbitrap) m/z: $[M + H_5]^{+5}$ Calcd for $C_{150}H_{201}N_{46}O_{20}S^{+5}$ 599.7164; Found 599.7178.

MS/MS fragmentation of **6ma**:

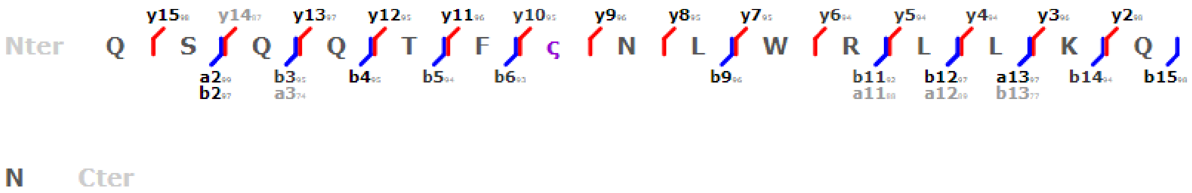

ç = Cys(C44H23N5)
Nter = C2H3O
Cter = NH2

Sequence	Type	MF	MF Mass	m/z	Intensity	Similarity

QS	a2	C9H16N3O4(+1)	230.11	230.11	21.24	98.96%
QN	y2	C9H18N5O4(+1)	260.14	260.14	40.89	98.36%
QSQQTFCNLWRLLKQN		C134H165N33O25S	2668.2	1335.1	22.29	98.12%
SQQTFCNLWRLLKQN	y15	C127H156N31O22S(+1)	2499.2	2499.2	14.09	97.97%
QSQQTFCNLWRLLKQ	b15	C130H157N30O23S(+1)	2538.2	1269.6	9.71	97.73%
QTFCNLWRLLKQN	y13	C119H143N28O18S(+1)	2284.1	2284.1	29.55	97.56%
SQQTFCNLWRLLKQN	y15	C127H156N31O22S(+1)	2499.2	1250.1	7.33	97.54%
QSQQTFCNLWRLL	a13	C118H137N26O19S(+1)	2254	2254	19.58	97.23%
QS	b2	C10H16N3O5(+1)	258.11	258.11	35.86	96.98%
FCNLWRLLKQN	y11	C110H128N25O14S(+1)	2055	2055	48.82	96.82%
TFCNLWRLLKQN	y12	C114H135N26O16S(+1)	2156	2156	65.18	96.69%
QSQQTFCNLWRL	b12	C113H126N25O19S(+1)	2168.9	2168.9	9.16	96.63%
QTFCNLWRLLKQN	y13	C119H143N28O18S(+1)	2284.1	1142.5	3.23	96.53%
QQTFCNLWRLLKQN	y14	C124H151N30O20S(+1)	2412.1	1206.6	1.54	96.46%
FCNLWRLLKQN	y11	C110H128N25O14S(+1)	2055	1028	2.13	96.02%
QSQQTFCNL	b9	C90H93N18O16S(+1)	1713.7	1713.7	6.7	95.89%
NLWRLLKQN	y9	C54H91N18O12(+1)	1183.7	1183.7	42.88	95.85%
KQN	y3	C15H30N7O5(+1)	388.23	388.23	80.01	95.79%
CNLWRLLKQN	y10	C101H119N24O13S(+1)	1907.9	1907.9	55.8	95.77%
WRLLKQN	y7	C44H74N15O9(+1)	956.58	956.58	67.59	95.47%
QSQQ	b4	C20H32N7O9(+1)	514.23	514.23	31.42	95.39%
LWRLLKQN	y8	C50H85N16O10(+1)	1069.7	1069.7	33.61	95.05%
QSQ	b3	C15H24N5O7(+1)	386.17	386.17	23.2	94.84%
RLLKQN	y6	C33H64N13O8(+1)	770.5	770.5	54.24	94.25%
TFCNLWRLLKQN	y12	C114H135N26O16S(+1)	2156	1078.5	3.44	94.23%
QSQQT	b5	C24H39N8O11(+1)	615.27	615.27	35.09	94.22%
CNLWRLLKQN	y10	C101H119N24O13S(+1)	1907.9	954.46	1.77	94.01%
LLKQN	y5	C27H52N9O7(+1)	614.4	614.4	7.95	93.85%
LKQN	y4	C21H41N8O6(+1)	501.31	501.31	13.8	93.72%
QSQQTFCNLWRLLK	b14	C125H149N28O21S(+1)	2410.1	2410.1	17.73	93.66%
QSQQTF	b6	C33H48N9O12(+1)	762.34	762.34	1.3	92.71%
QSQQTFCNLWR	b11	C107H115N24O18S(+1)	2055.9	2055.9	11.02	92.25%
QSQQTFCNLWRL	a12	C112H126N25O18S(+1)	2140.9	2140.9	3.52	89.45%
QSQQTFCNLWR	a11	C106H115N24O17S(+1)	2027.9	2027.9	1.77	87.94%
QSQQTFCNLWRLL	b13	C119H137N26O20S(+1)	2282	2282	21.7	76.80%
QQTFCNLWRLLKQN	y14	C124H151N30O20S(+1)	2412.1	2412.1	25.2	76.56%
QSQ	a3	C14H24N5O6(+1)	358.17	358.17	6.11	74.21%

6 Photophysical properties of CzPN-peptide conjugates

10 μM aerated solutions of the samples in DMSO or in water + 1 % DMSO were analysed in a quartz capillary.The emission and excitation spectra were recorded at room temperature on a Horiba-Jobin Yvon Fluorolog FL-3-22 fluorimeter equipped with CW 450W Xenon source for fluorescence mode. Data were collected by using a thermoelectrically cooled R2658P PMT (Hamamatsu, range 220-1010 nm) or a NIR PMT (950-1700 nm, thermoelectrically cooled H10330-75 NIR-PMT; Hamamatsu). The spectra were corrected by the instrumental correction function. Data processing was

performed with the program Origin 8®. Life time decays were recorded by using TCSPC Delta time unit, using a Xenon source. The data were analysed by using DAS software, by using a mono, bi- or tri-exponential function, and the best fits were kept. They are the averages of at least three independent measurements.

Quantum yield measurements were performed using a G8 GMP integrating sphere [$\Phi = (E_c - E_a)/((L_a - L_c).F_{att}(\lambda)$, where E_c is the integrated emission spectrum of the sample, E_a is the integrated "blank" emission spectrum, L_a is the "blank" absorption, and L_c is the sample absorption at the excitation wavelength). $F_{att}(\lambda) = 100/T$ where T is the attenuation factor of the neutral density filter expressed in percentage. Neutral density filters with an optical density of 1, 2 or 3 were used. Values are the averages of at least three independent measurements.

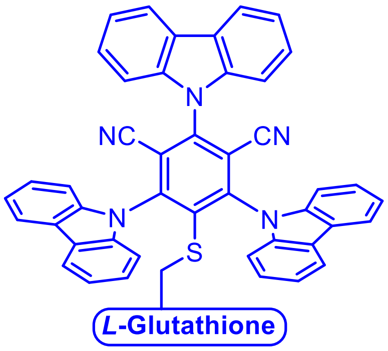

6ca

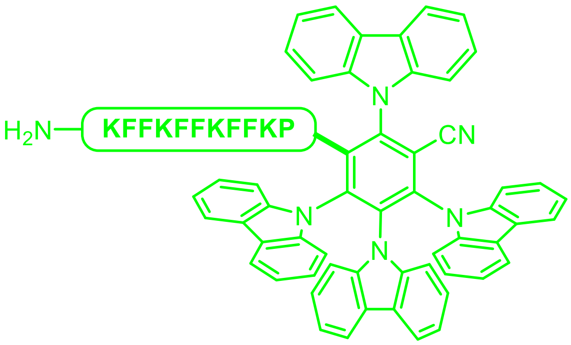

3ya

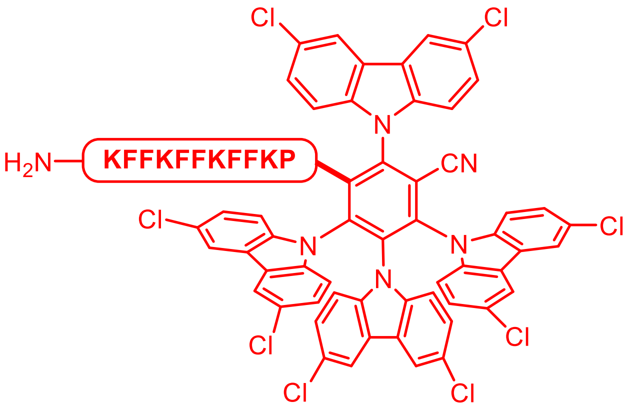

3yb

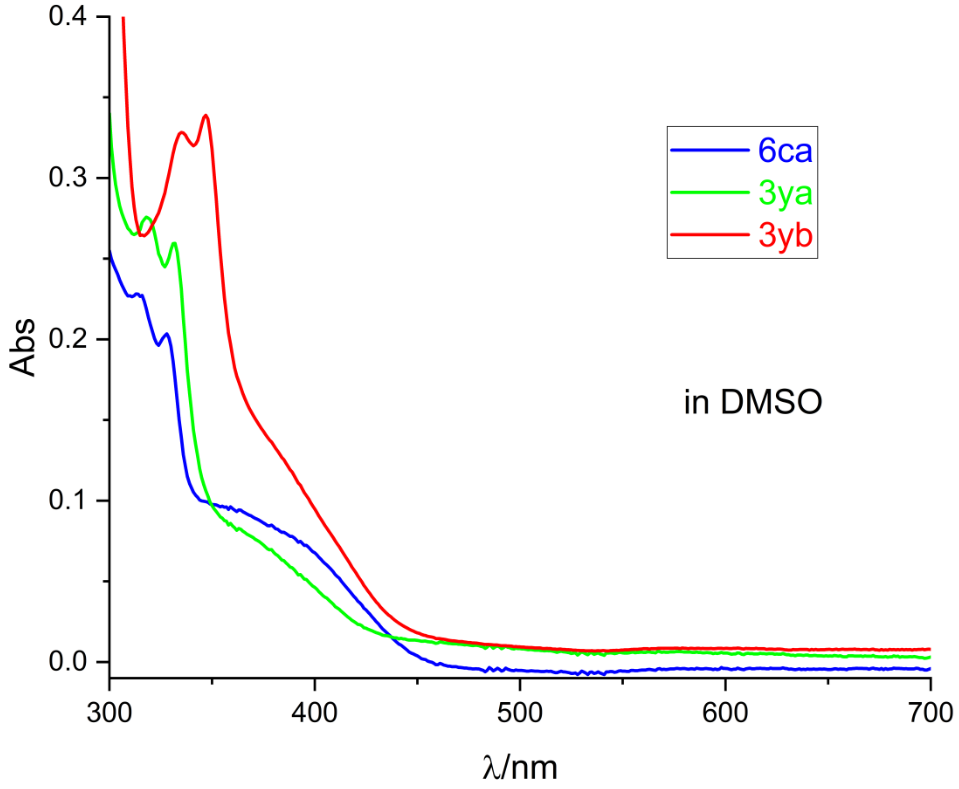
0.4
0.3
0.2
0.1
0.0
Abs
6ca
3ya
3yb
in DMSO
300
400
500
600
700
λ/nm

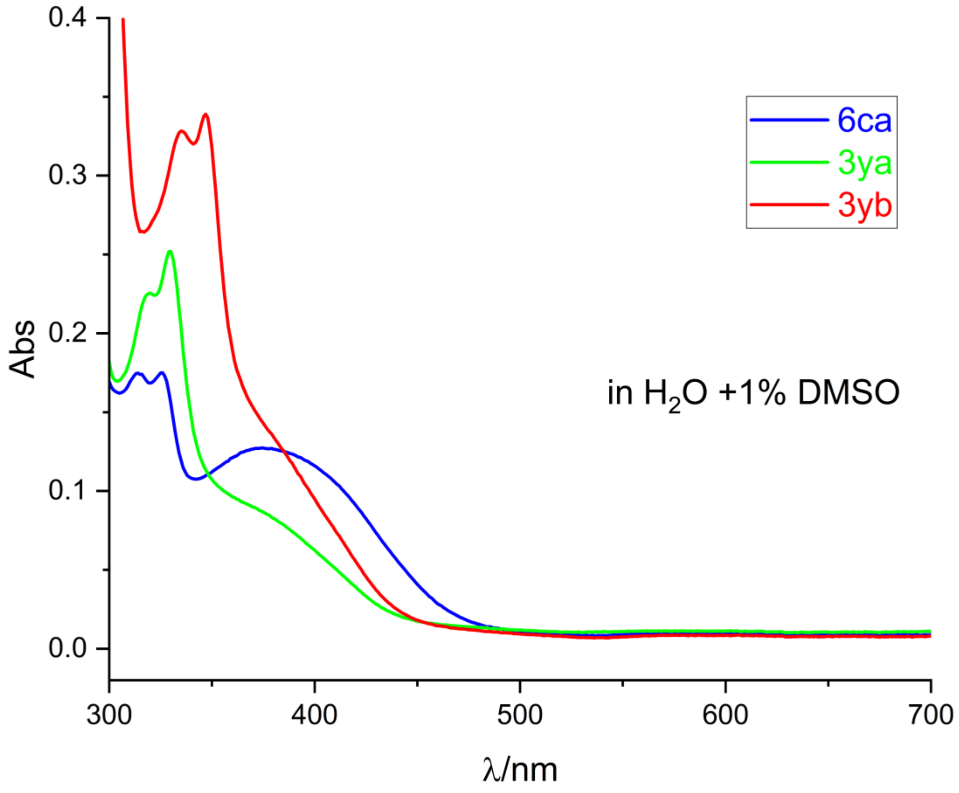
0.4
0.3
0.2
0.1
0.0
Abs
6ca
3ya
3yb
in H2O +1% DMSO
300
400
500
600
700
λ/nm

Figure S1: UV-Vis spectra of the different compounds in 10 μM solutions DMSO (top) or in water in presence of+0.1% DMSO(down).

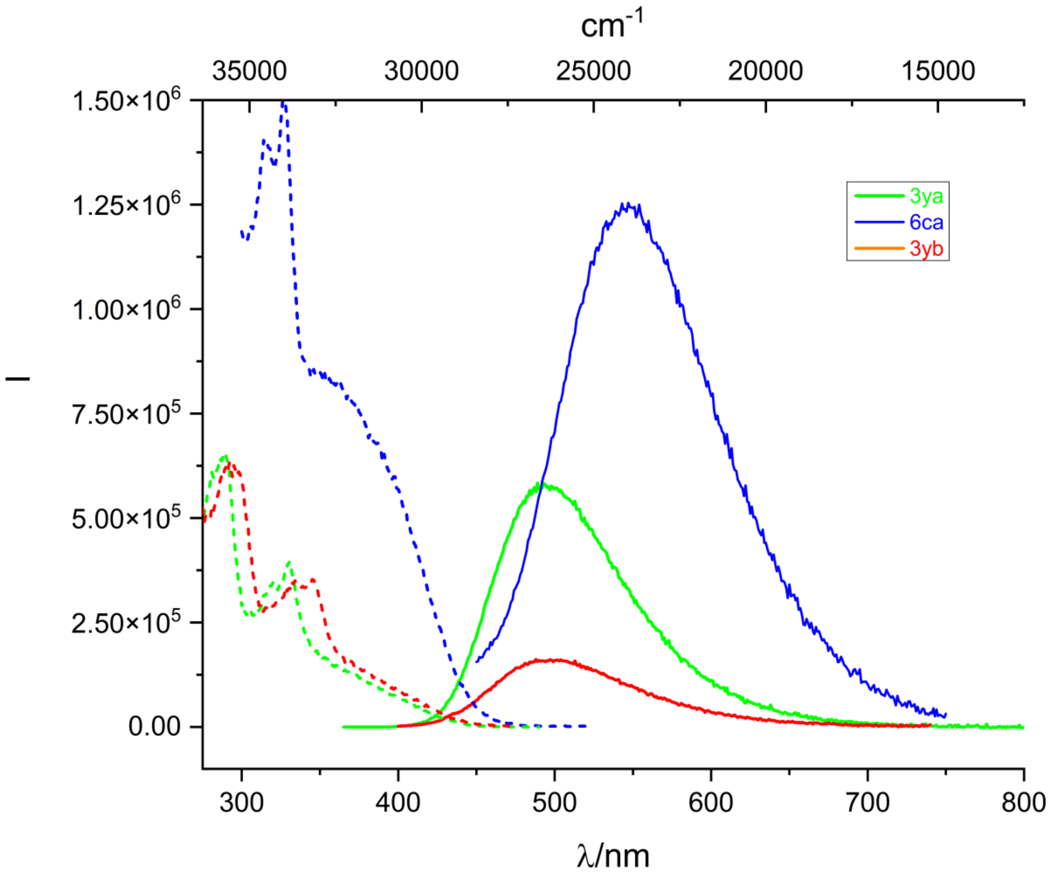

Figure S2: Corrected excitation and emission spectra of the different CzPN-peptide conjugates at room temperature, in water in presence of 0.1% DMSO (10 μM). Solid lines: emission spectra with λ_{ex}= 330 nm Dashed lines: excitation spectra, λ_{em} corresponds to the maximum of the emission spectra, see table S3 for exact values.

Table S3: Lifetimes of CzPN-peptide conjugates 10 μM in DMSO or water in presence of 0.1% DMSO λ_{ex} = 330 nm and λ_{em} corresponds to the maximum of the emission,n as indicated in the column below.

Entry	Compound	Solvent	λ_{em}/nm	τ_1/μs	τ_2/μs
1	4CzIPN	DMSO	552	1.52 ± 0.06	11.6 ± 0.97
2	**3ya**	DMSO	500	1.72 ± 0.03	8.81 ± 0.09
3		H_2O*	493	1.36 ± 0.04	8.70 ± 0.11
4	**6ca**	DMSO	541	0.99±0.01	8.54±0.07
5		H_2O*	546	0.99±0.01	8.60±0.10
6	8Cl-4CzIPN	DMSO	542	1.19 ± 0.03	10.11 ± 0.31
7	**3yb**	DMSO	493	1.06 ± 0.02	8.30 ± 0.17
8		H_2O*	497	1.24 ± 0.03	8.64 ± 0.12

*Contain 0.1% DMSO

Table S4: Absolute quantum yield of CzPN-peptide conjugates in %.

Entry	Compound	DMSO		H_2O (0.1% DMSO)	
		λ_{ex} = 330 nm	λ_{ex} = 380nm	λ_{ex} = 330 nm	λ_{ex} = 380nm
1	4CzIPN	19.7 ± 1.0	15.2 ± 1.3	-[a]	-[a]
2	**3ya**	15.4 ± 0.8	5.3 ± 0.8	7.3 ± 0.5	2.2 ± 0.5
3	**6ca**	7.7 ± 0.6	5.9 ± 0.3	5.6 ± 0.1	3.1 ± 0.2
4	8Cl-4CzIPN	14.8 ± 1.2	13.6 ± 0.2	-[a]	-[a]
5	**3yb**	8.0 ± 3.1	5.8 ± 0.3	4.7 ± 1.1	4.6 ± 0.2

Concentration: 10 µM. [a]The quantum yield in H_2O was not measured due to limited solubility

7. Electrochemical measurements of CzPN-peptide conjugates

Cyclic Voltammetry (CV) was performed using an Autolab Potentiostat, with a three-electrode cell configuration: a glassy carbon electrode as the working electrode, Pt wire as a counter electrode and an Ag/AgCl (KCl, 3M) electrode as the reference electrode. Bu_4NPF_6 was employed as the electrolyte (0.1 M) and ferrocene was added as the internal standard.

Table S5. Redox potential of CzPN-peptide conjugates

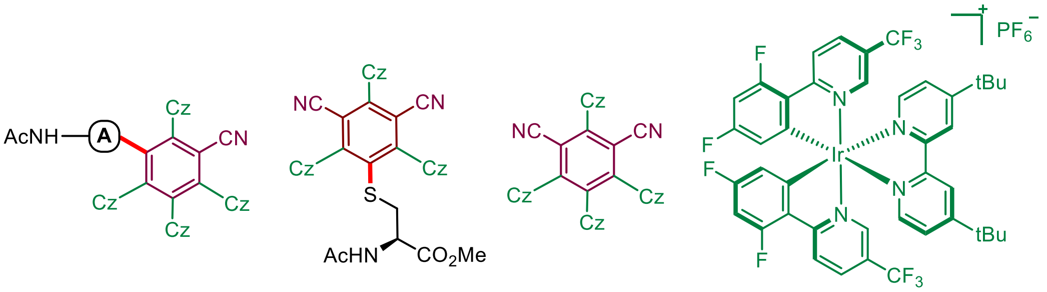

3za **6aa** **4CzIPN** **[Ir{dF(CF$_3$)ppy}$_2$(dtbpy)]PF$_6$**

PC	$E_{0\text{-}0}$ eV (kcal/mol)	$E_{1/2}(P/P^-)$ eV	$E_{1/2}(P^*/P^-)$ eV	$E_{1/2}(P^+/P)$ eV	$E_{1/2}(P^+/P^*)$ eV
**3za**	2.93 (67.6)	-1.59	1.34	1.51	-1.50
**6aa**	2.65 (61.1)	-1.41	1.24	1.64	-1.01
**4CzIPN**[a]	2.56 (59.0)	-1.21	1.52	1.35	-1.04
**Ir**[a]	2.61 (60.1)	-1.37	1.21	1.69	-0.89

All the data was measured in MeCN. The excitation energy $E_{0\text{-}0}$ was estimated by the point of intersection of the normalized absorbance and emission signals. $E_{1/2}(P^+/P^*) = E1/2(P^+/P) - E_{0\text{-}0}$ and $E_{1/2}(P^*/P^-) = E_{0\text{-}0} + E_{1/2}(P/P^-)$. [a]The data was taken from the reported literature.[17]

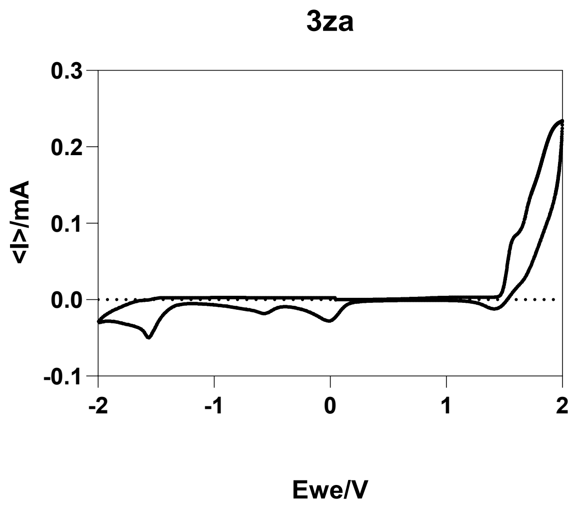

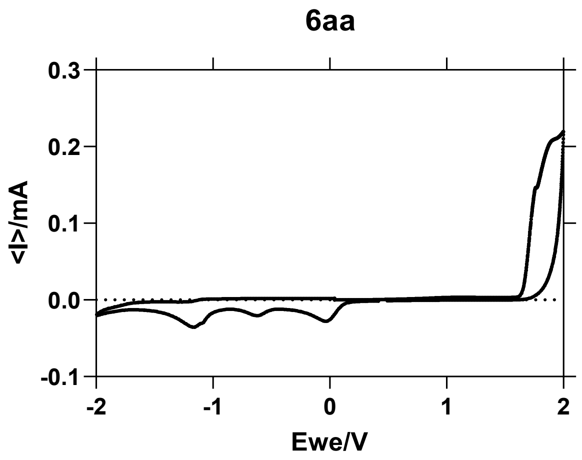

Figure S3: CV diagram of **3za** and **6aa**

8. Application of CzBN/CzIPN-peptide conjugates on photo-mediated biomolecule functionalizations

8.1 Decarboxylative alkynylation of peptide C-termini enabled by CzBn/CzIPN-peptide conjugates

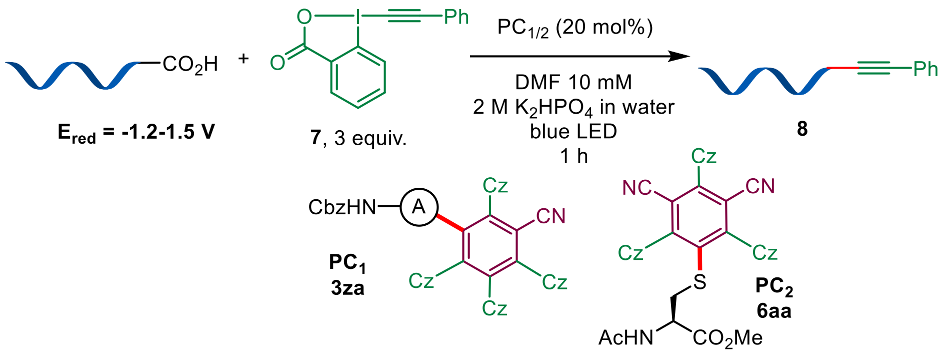

General procedure: 20 μL of peptide (1.0 μmol, 10 mM, 1 equiv., 50 mM stock solution in DMF), 10 μL of a solution of CzBn/CzIPN conjugates **3za/6aa** (2.0 mM, 20 mol%, 20 mM stock solution in DMF), 5 μL of a solution of K_2HPO_4 (10 μmol, 10 equiv., 2 M in water) and 60 μL of Ph-EBX (3.0 μmol, 20 mM, 3.0 equiv., 50 mM stock solution in DMF) were added in a 1 mL vial. Then 15 μL of DMF was added into the vial to reach the overall concentration: 10 mM. The vial was then capped and bubbled with N_2 for 15 min. The reaction mixture was irradiated with 440 nm Kessil lamp (intensity: 25%) for 1 hour at RT.
The reaction crude was analyzed by HPLC/MS, and the desired product **8** was isolated by RP-HPLC (the isolated yield was not calculated due to the small reaction scale).

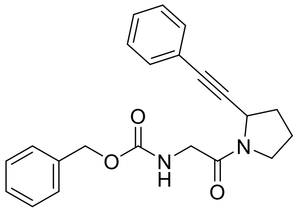

Following the **general procedure**, the desired product **8a** was isolated by RP-HPLC with **Method 1**. The isolated yield was not reported due to the limited reaction scale.
HPLC-UV chromatogram (210 nm) of the crude with **3za** by **Method 1**:
HPLC-UV yield: 89%. The rention time is consist with the reported data (>95% HPLC yield was reported using 4CzIPN).[9]

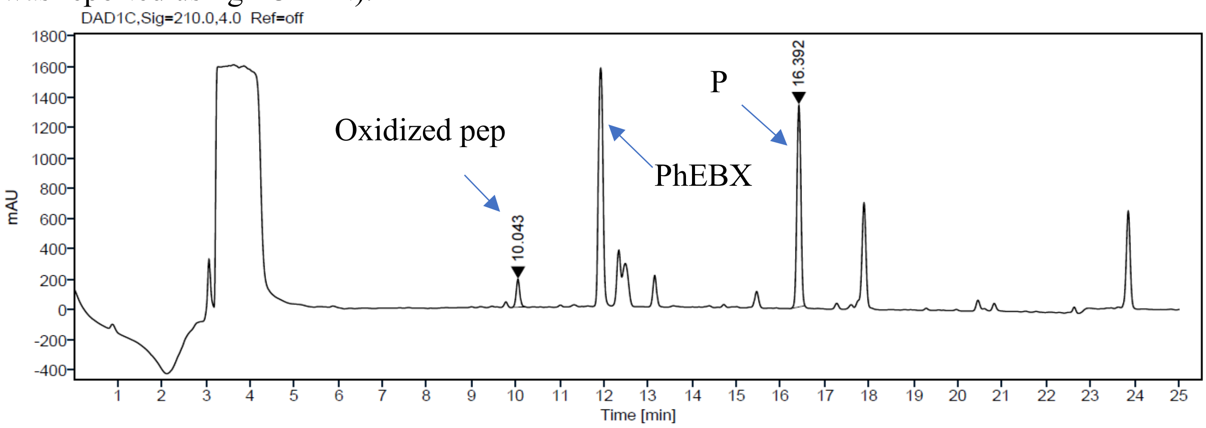

HPLC-UV chromatogram (210 nm) of the crude with **6aa** by **Method 1**:
HPLC-UV yield: 79%

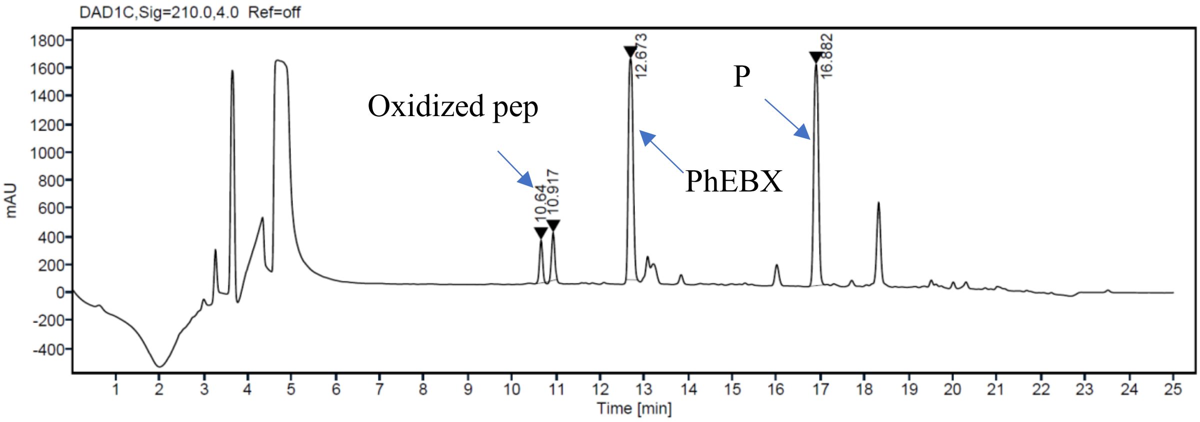

HPLC-UV chromatogram (210 nm) of product **8a** by **Method 1**:

[9] Garreau, M.; Le Vaillant, F.; Waser, J., *Angew. Chem., Int. Ed.* **2019,** *58* (24), 8182-8186.

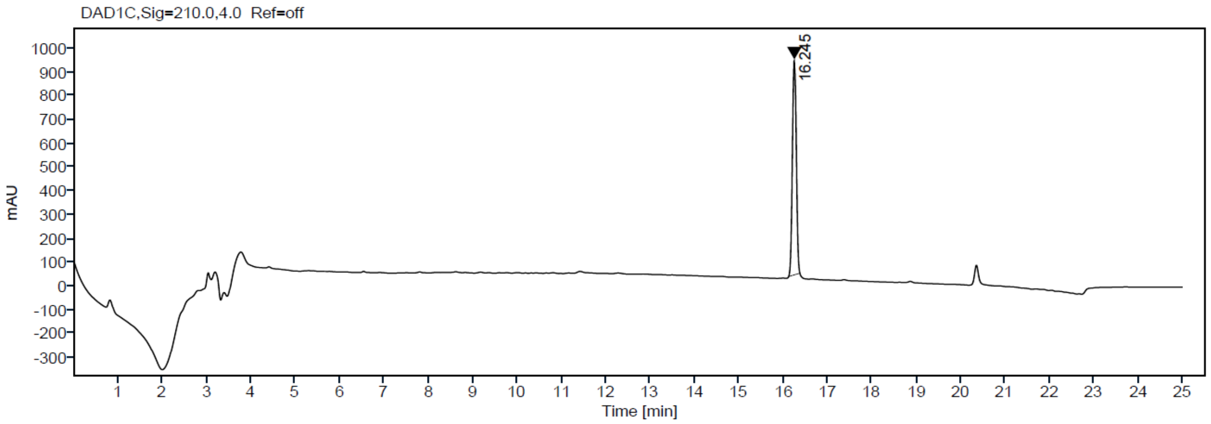

Retention time: 16.269 min **Area Percent:** 100%

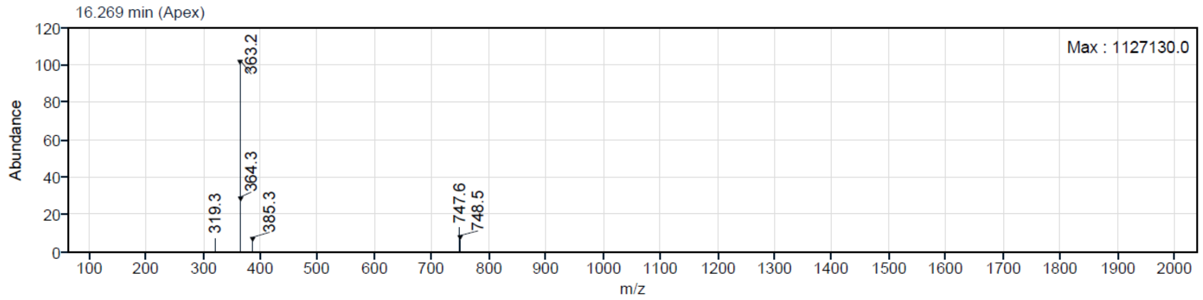

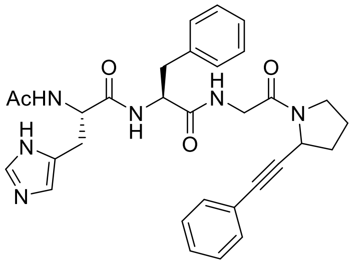

Following the **general procedure**, the desired product **8b** was isolated by RP-HPLC with **Method 1**.

HPLC-UV chromatogram (210 nm) of the crude with PC1 **3za** by **Method 1**:

HPLC-UV yield: 15%

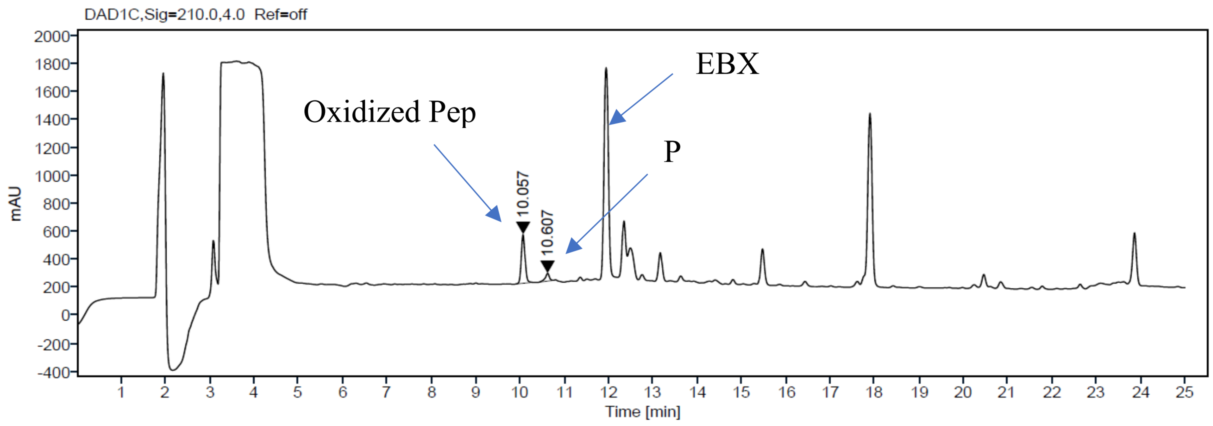

HPLC-UV chromatogram (210 nm) of the crude with PC2 **6aa** by **Method 1**:

HPLC-UV yield: 52%

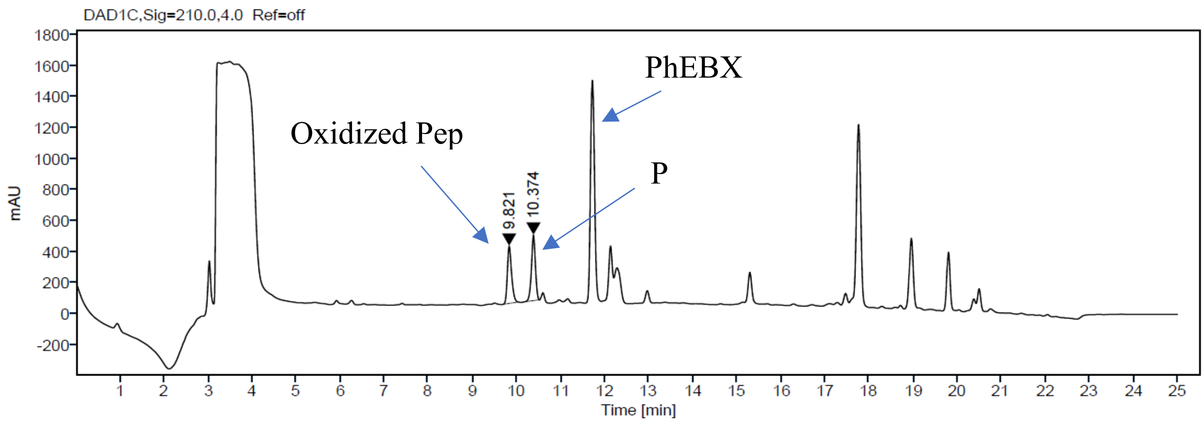

HPLC-UV chromatogram (210 nm) of the crude with 4CzIPN **2a** by **Method 1**:
HPLC-UV yield: 51%

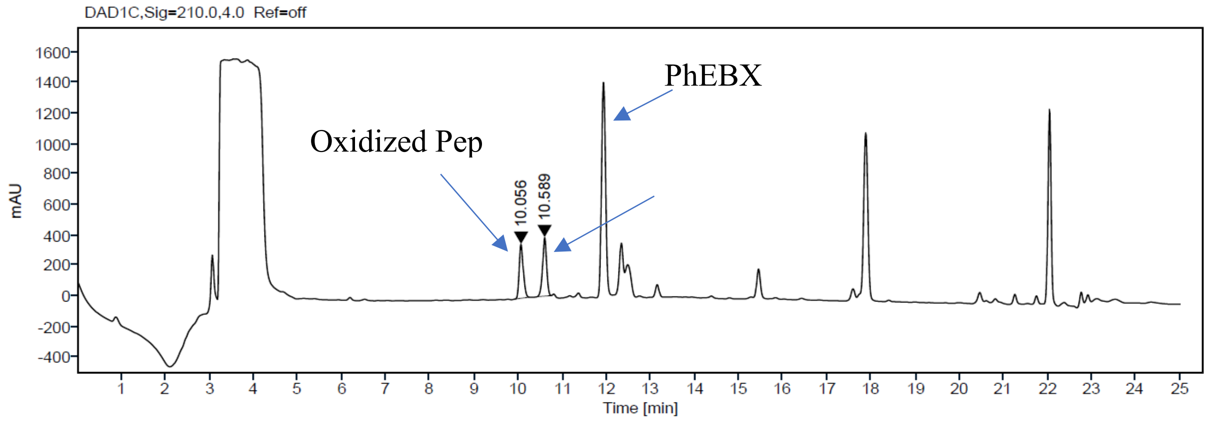

HPLC-UV chromatogram (210 nm) of the crude without **PC** by **Method 1**: (Messy HPLC)

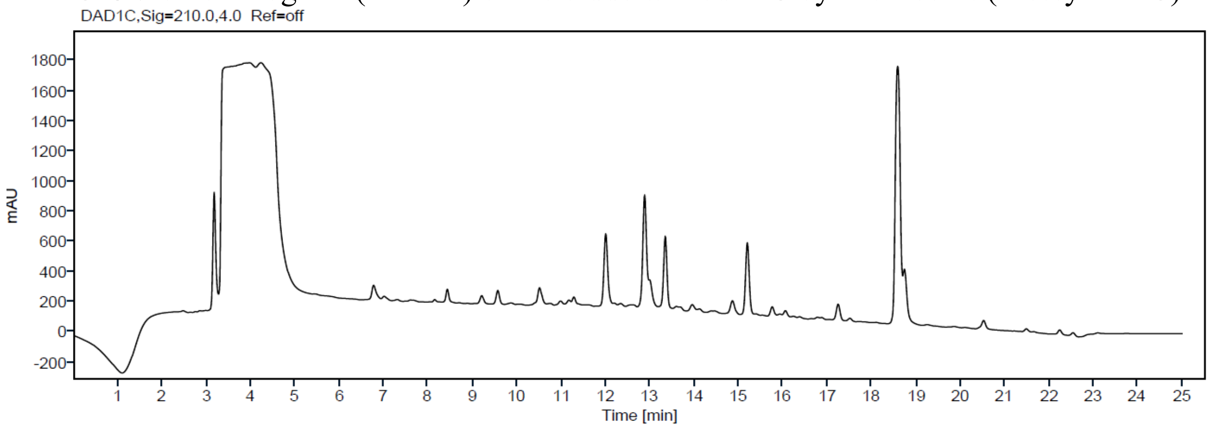

HPLC-UV chromatogram (210 nm) of product **8b** by **Method 1**:

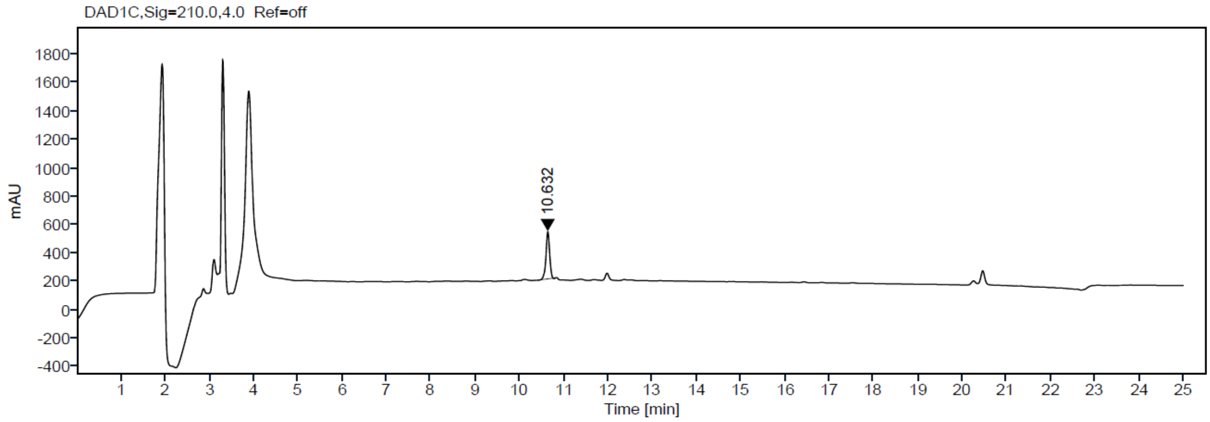

Retention time: 10.673 min **Area Percent:** 100%

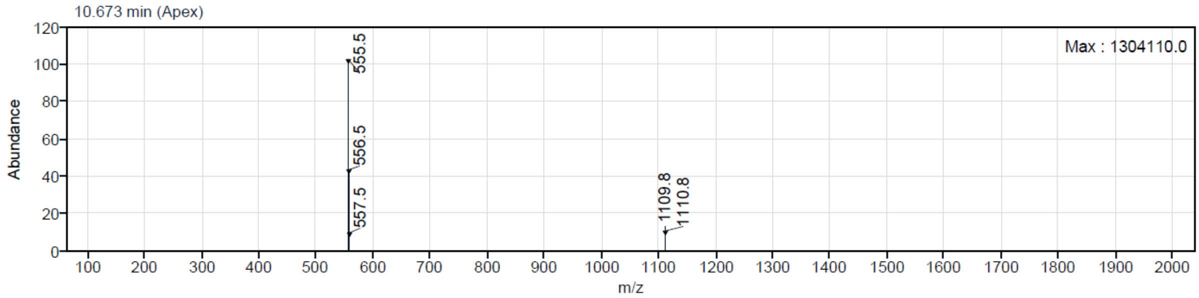

HRMS (nanochip-ESI/LTQ-Orbitrap) m/z: $[M + H]^+$ Calcd for $C_{31}H_{35}N_6O_4^+$ 555.2714; Found 555.2723.

MS/MS fragmentation of **8b**:

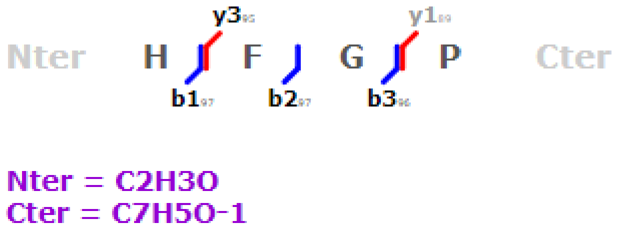

Sequence	Type	MF	MF Mass	m/z	Intensity	Similarity
HF	b2	C17H19N4O3(+1)	327.15	327.15	47.87	97.33%
H	b1	C8H10N3O2(+1)	180.08	180.08	6.67	96.89%
HFG	b3	C19H22N5O4(+1)	384.17	384.17	102.55	95.53%
FGP	y3	C23H26N3O2(+1)	376.2	376.2	54.12	95.43%
HFGP		C31H34N6O4	554.26	555.27	14.48	94.68%
P	y1	C12H14N(+1)	172.11	172.11	2.93	88.51%

8.2 Thiol-ene of peptide enabled by CzPN-peptide conjugates

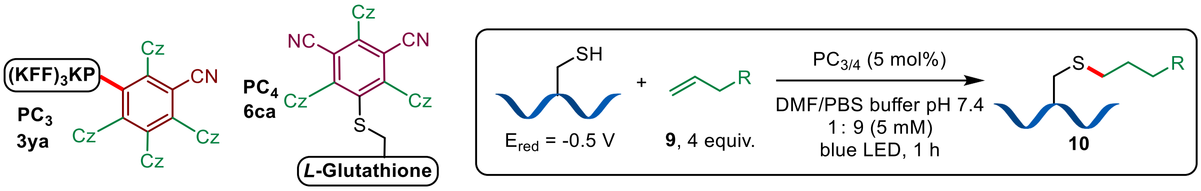

General procedure: 10 μL of peptide **4** (1 μmol, 5 mM, 1 equiv., 50 mM stock solution in DMF), 1 μL of a solution of CzBN/3CzIPN conjugates (0.25 mM, 5.0 mol%, 50 mM stock solution in DMF) and 4 μL of alkene **9** (4.0 μmol, 20 mM, 4.0 equiv., 1 M stock solution in DMF) were added in a 1 mL vial. Then 5 μL of DMF and 180 μL of PBS buffer (pH 7.4) were added into the vial to reach the overall concentration: 5 mM (DMF : PBS buffer 1:9). The vial was then capped and freeze-pump thaw for 3 times. The reaction mixture was irradiated with 440 nm Kessil lamp (intensity: 25%) for 1 hour at RT.
The reaction mixture was analyzed by HPLC/MS, and the product was purified by RP-HPLC (the isolated yield was not calculated due to the small reaction scale).

N-acetyl-S-(3-hydroxypropyl)-L-cysteine 10a

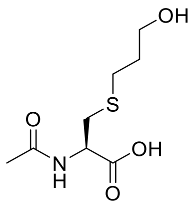

Following the **General procedure**, the reaction was run on 0.02 mmol scale with **6ca** as the photocatalyst. The desired product **10a** was isolated as colorless solid (2.7 mg, 0.012 mmol, 61%) by RP-HPLC with **Method 1**.

^{1}H NMR (800 MHz, DMSO) δ 8.18 (d, J = 8.0 Hz, 1H, COOH), 4.48 (s, 1H, NH), 4.33 (td, J = 8.1, 4.9 Hz, 1H, NCH), 3.43 (t, J = 6.2 Hz, 2H, CH_2OH), 2.86 (dd, J = 13.6, 5.0 Hz, 1H, CH_2S), 2.70 (dd, J = 13.6, 8.2 Hz, 1H, CH_2S), 2.54 (t, J = 7.3 Hz, 2H, SCH_2C), 1.85 (s, 3H, CH_3CO), 1.66 – 1.60 (m, 2H, CCH_2C).

^{13}C NMR (201 MHz, DMSO) δ 172.3, 169.3, 59.3, 52.2, 33.0, 32.3, 28.3, 22.4.

HPLC-UV chromatogram (210 nm) of the crude with **3ya** by **Method 1**:

HPLC-UV yield: 94%

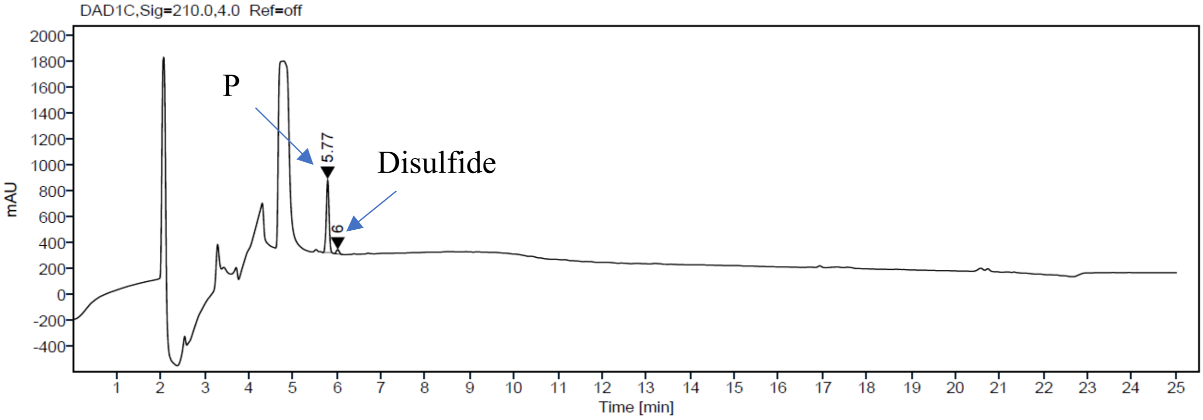

HPLC-UV chromatogram (210 nm) of the crude with **6ca** by **Method 1**:

HPLC-UV yield: 84%

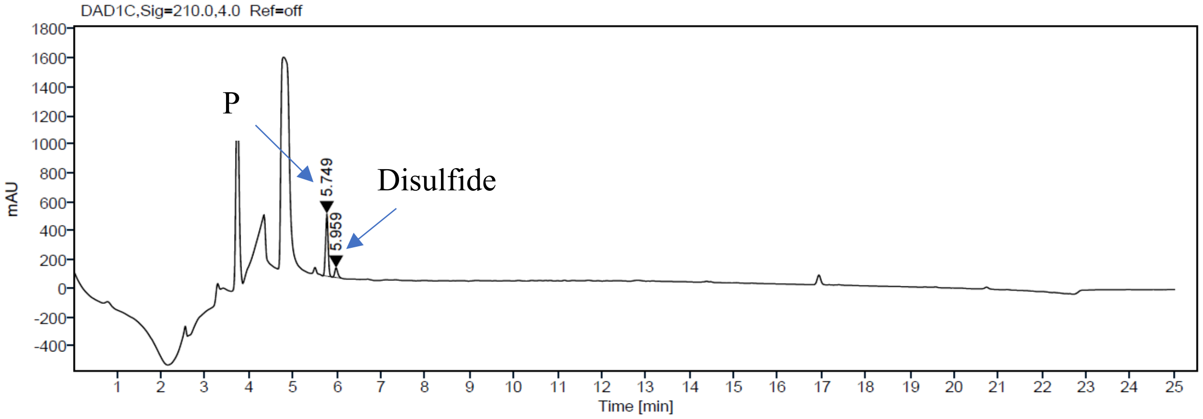

HPLC-UV chromatogram (210 nm) of the crude with **4CzIPN** by **Method 1**:

HPLC-UV yield: 24%, rest is starting material Ac-Cys-OH.

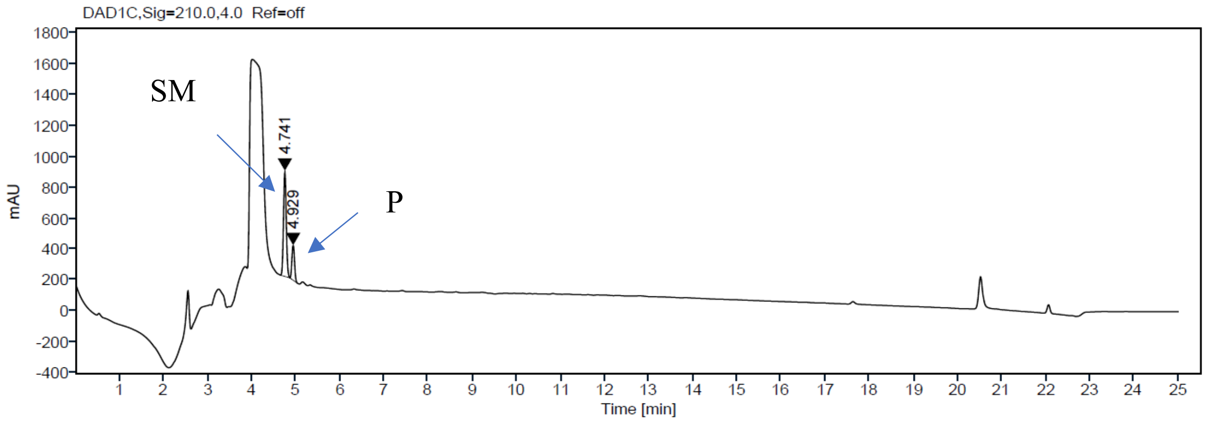

HPLC-UV chromatogram (210 nm) of product **10a** by **Method 1**:

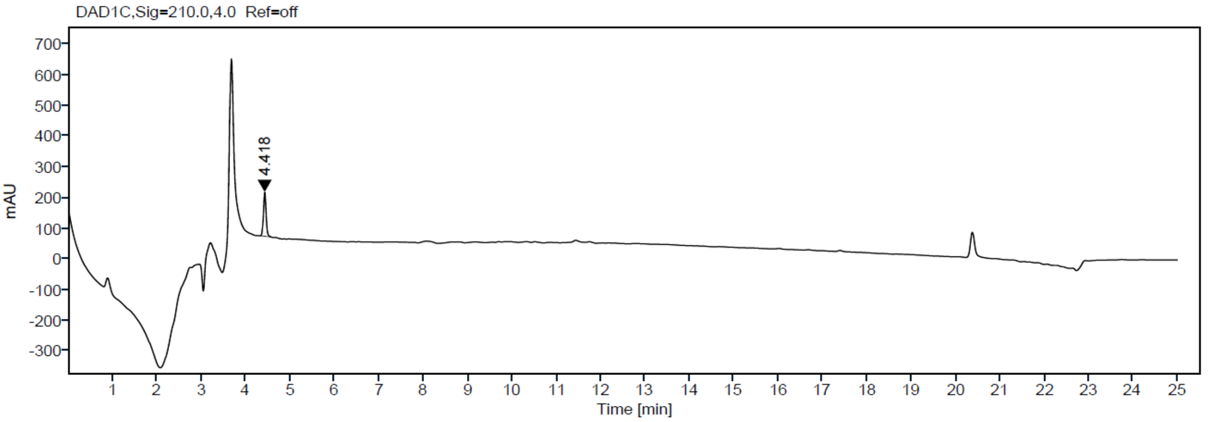

Retention time: 4.441 min **Area Percent:** 100%

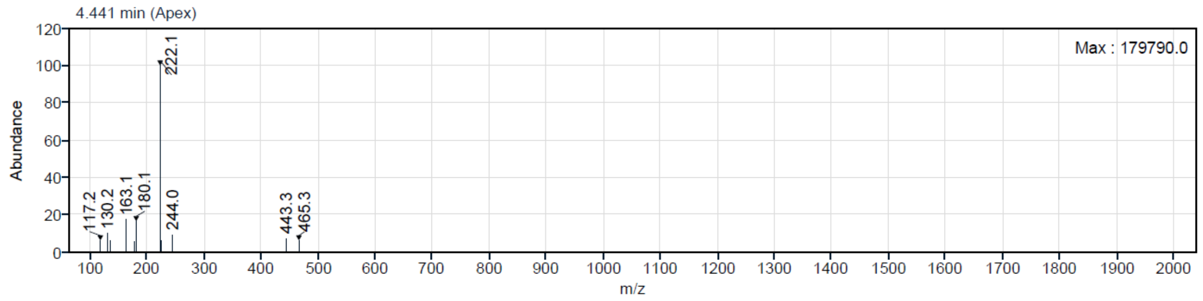

HRMS (ESI/QTOF) m/z: $[M + Na]^+$ Calcd for $C_8H_{15}NNaO_4S^+$ 244.0614; Found 244.0617.

AcGC(allyl alcohol)RPKPQQFFGLM-NH₂ 10b

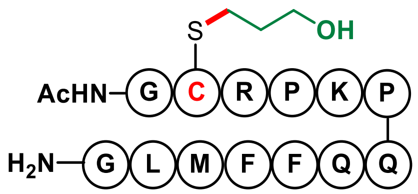

Following the **General procedure**, the desired product **10b** was isolated as colorless solid by RP-HPLC with **Method 1.** The isolated yield was not reported due to the limited reaction scale.
HPLC-UV chromatogram (210 nm) of the crude with **3ya** by **Method 1**:
HPLC-UV yield: messy HPLC, low yield.

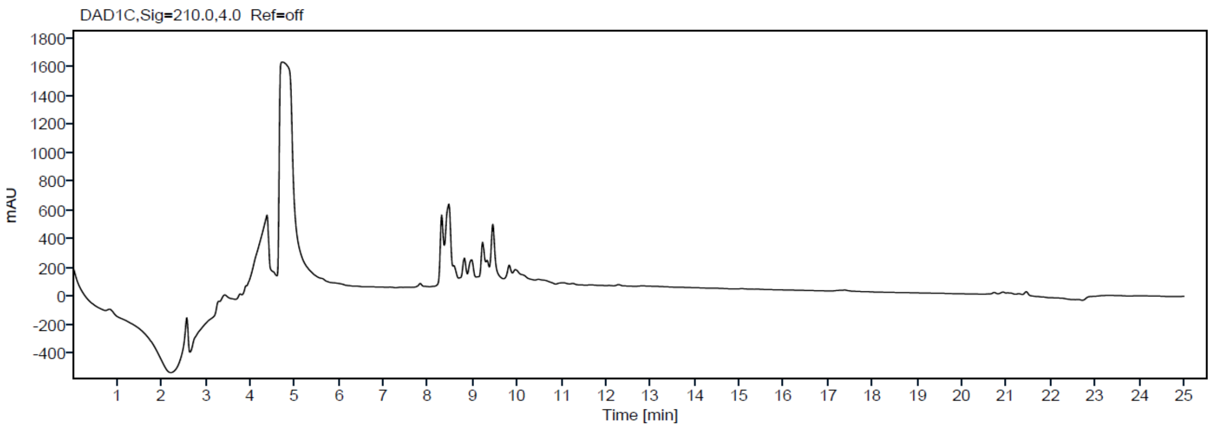

HPLC-UV chromatogram (210 nm) of the crude with **6ca** by **Method 1**:
HPLC-UV yield: 69%.

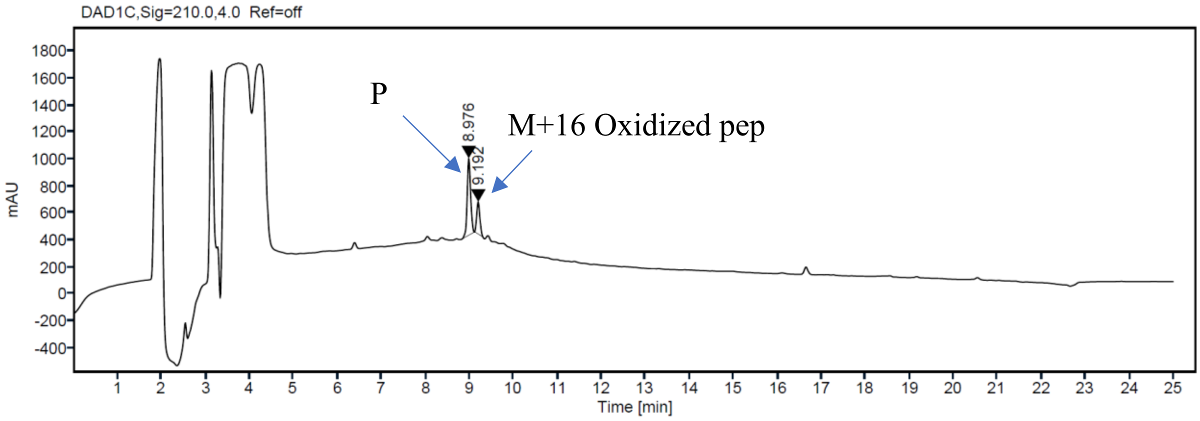

HPLC-UV chromatogram (210 nm) of **10b** by **Method 1**:

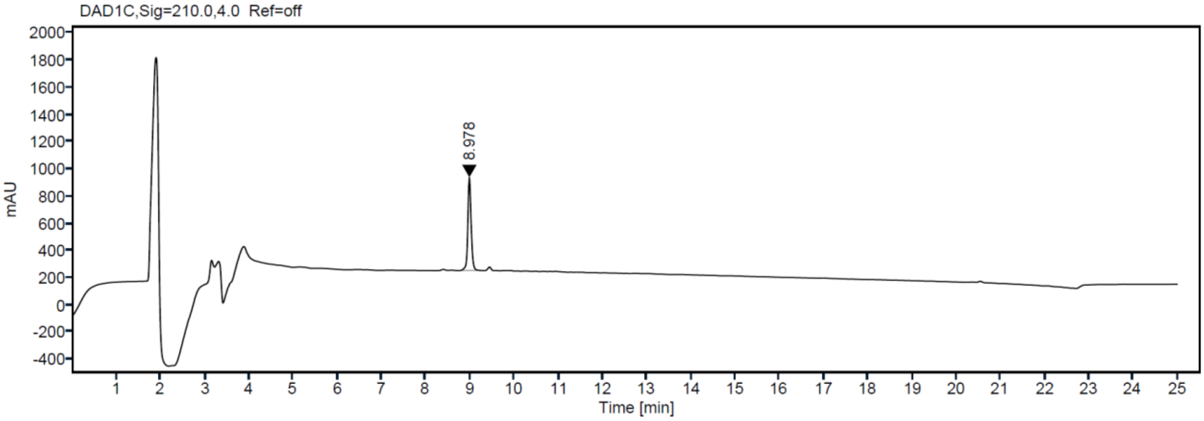

Retention time: 8.993 min **Area Percent:** 100%

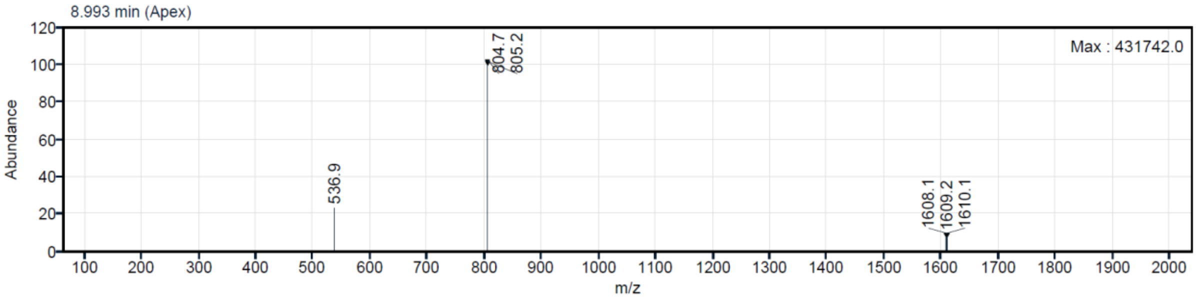

HRMS (nanochip-ESI/LTQ-Orbitrap) m/z: $[M + H_2]^{+2}$ Calcd for $C_{73}H_{116}N_{20}O_{17}S_2^{+2}$ 804.4129; Found 804.4144.

MS/MS fragmentation of **10b**:

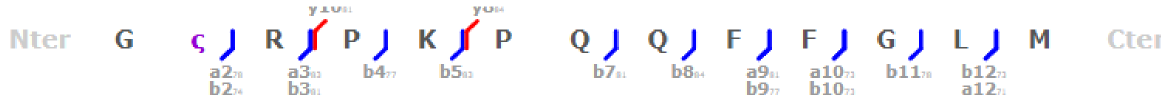

ς = Cys(C13H20N2O3S)
Nter = C2H3O
Cter = NH2

Sequence	Type	MF	MF Mass	m/z	Intensity	Similarity
GCRPKPQQ	b8	C52H85N16O14S2(+1)	1221.6	1221.6	17.81	84.59%
PQQFFGLM	y8	C46H68N11O10S(+1)	966.49	966.49	16.67	84.33%
GCRPKPQQ	b8	C52H85N16O14S2(+1)	1221.6	611.3	2.86	83.62%
GCR	a3	C25H43N8O6S2(+1)	615.27	615.27	5.64	82.66%
GCRPK	b5	C37H62N11O9S2(+1)	868.42	868.42	74.88	82.64%
GCRPKPQQF	a9	C60H94N17O14S2(+1)	1340.7	670.83	1.84	81.25%
GCR	b3	C26H43N8O7S2(+1)	643.27	643.27	103.33	81.14%
GCRPKPQ	b7	C47H77N14O12S2(+1)	1093.5	1093.5	14.35	80.96%
PKPQQFFGLM	y10	C57H87N14O12S(+1)	1191.6	1191.6	9.25	80.80%
GC	a2	C19H31N4O5S2(+1)	459.17	459.17	1.84	78.48%
GCRPKPQQFFG	b11	C72H106N19O17S2(+1)	1572.7	786.88	5.29	78.11%
GCRPKPQQF	b9	C61H94N17O15S2(+1)	1368.7	684.83	5.73	77.36%
GCRP	b4	C31H50N9O8S2(+1)	740.32	740.32	3.73	77.34%
GCRPKPQQF	b9	C61H94N17O15S2(+1)	1368.7	1368.7	10.05	75.81%
GC	b2	C20H31N4O6S2(+1)	487.17	487.17	2.19	73.97%
GCRPKPQQFF	a10	C69H103N18O15S2(+1)	1487.7	744.37	4.86	73.31%
GCRPKPQQFF	b10	C70H103N18O16S2(+1)	1515.7	758.37	14.72	73.02%
GCRPKPQQFF	b10	C70H103N18O16S2(+1)	1515.7	1515.7	1.66	72.90%
GCRPKPQQFFGL	b12	C78H117N20O18S2(+1)	1685.8	843.42	48.96	72.66%
GCRPKPQQFFGL	a12	C77H117N20O17S2(+1)	1657.8	829.42	20.74	70.80%

AcGC(biotin)RPKQQFFGLM-NH$_2$ 10c

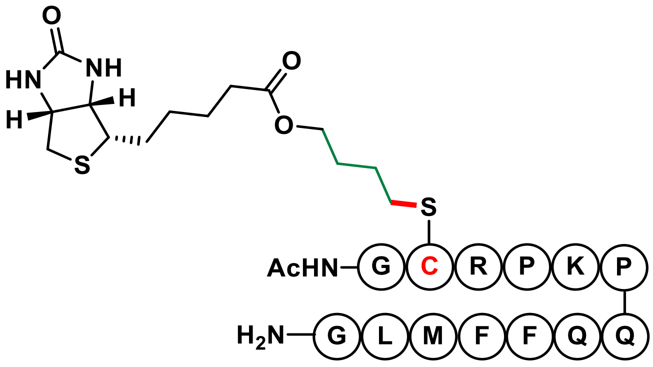

Following the **General procedure**, the desired product **10c** was isolated as colorless solid by RP-HPLC with **Method 1.** The isolated yield was not reported due to the limited reaction scale.

HPLC-UV chromatogram (210 nm) of the crude with **6ca** by **Method 1**:
HPLC-UV yield: 64%.

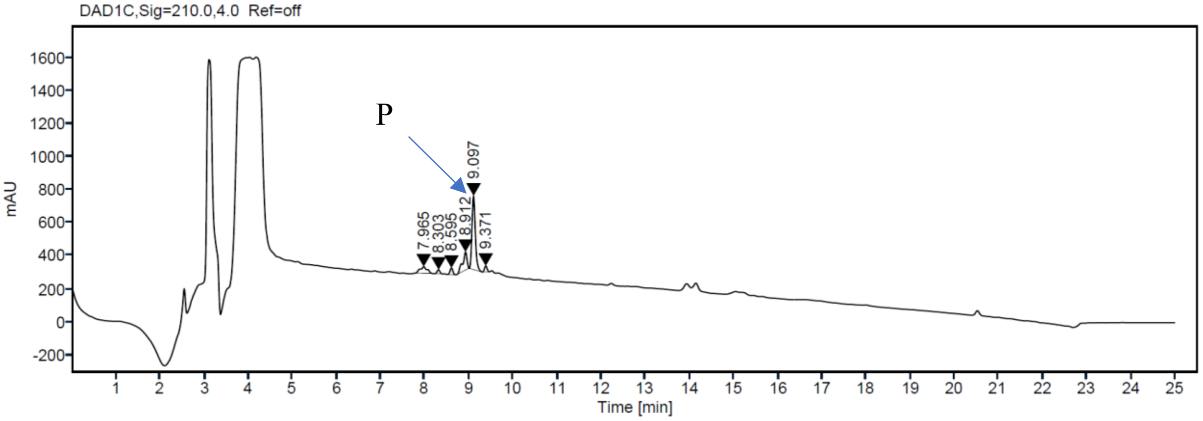

HPLC-UV chromatogram (210 nm) of **10c** by **Method 1**:

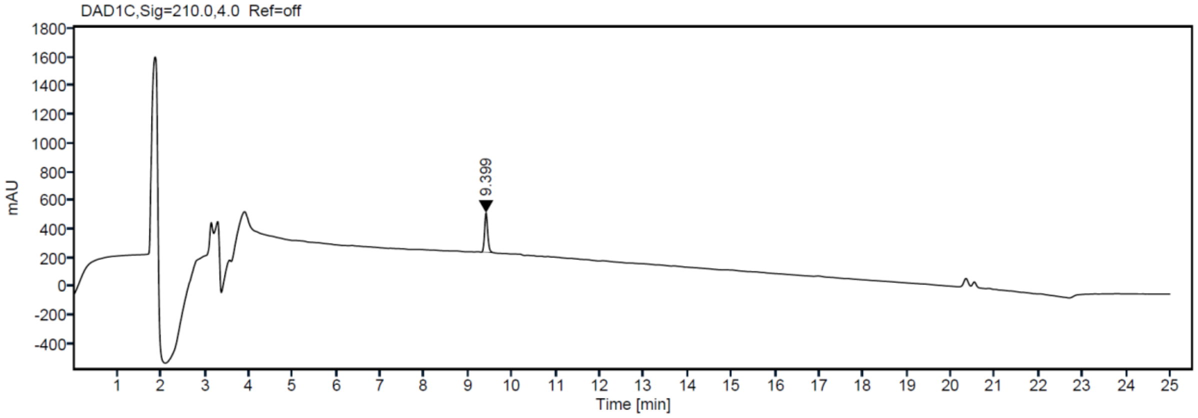

Retention time: 9.439 min **Area Percent:** 100%

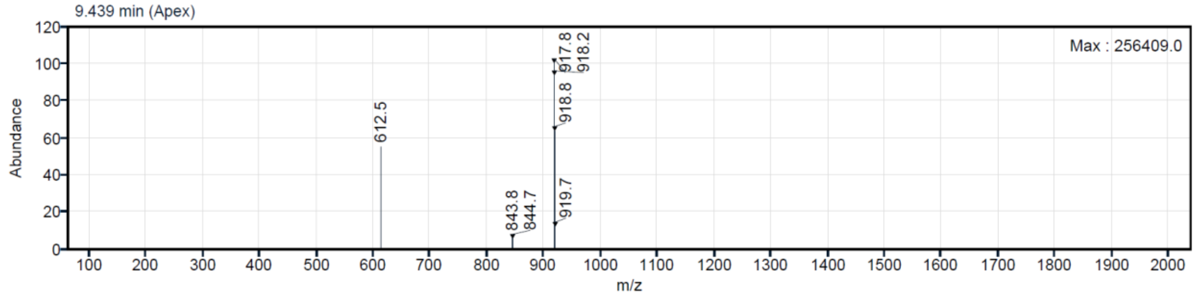

HRMS (nanochip-ESI/LTQ-Orbitrap) m/z: $[M + H_2]^{+2}$ Calcd for $C_{83}H_{130}N_{22}O_{19}S_3^{+2}$ 917.4517; Found 917.4536.

MS/MS fragmentation of **10c**:

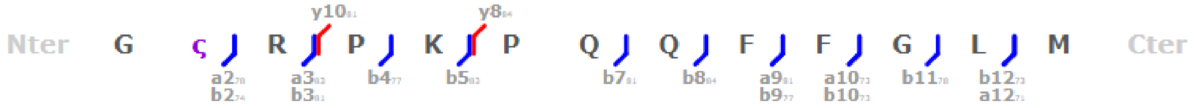

ς = Cys(C13H20N2O3S)
Nter = C2H3O
Cter = NH2

Sequence	Type	MF	MF Mass	m/z	Intensity	Similarity

GCRPKPQQ	b8	C52H85N16O14S2(+1)	1221.6	1221.6	17.81	84.59%
PQQFFGLM	y8	C46H68N11O10S(+1)	966.49	966.49	16.67	84.33%
GCRPKPQQ	b8	C52H85N16O14S2(+1)	1221.6	611.3	2.86	83.62%
GCR	a3	C25H43N8O6S2(+1)	615.27	615.27	5.64	82.66%
GCRPK	b5	C37H62N11O9S2(+1)	868.42	868.42	74.88	82.64%
GCRPKPQQF	a9	C60H94N17O14S2(+1)	1340.7	670.83	1.84	81.25%
GCR	b3	C26H43N8O7S2(+1)	643.27	643.27	103.33	81.14%
GCRPKPQ	b7	C47H77N14O12S2(+1)	1093.5	1093.5	14.35	80.96%
PKPQQFFGLM	y10	C57H87N14O12S(+1)	1191.6	1191.6	9.25	80.80%
GC	a2	C19H31N4O5S2(+1)	459.17	459.17	1.84	78.48%
GCRPKPQQFFG	b11	C72H106N19O17S2(+1)	1572.7	786.88	5.29	78.11%
GCRPKPQQF	b9	C61H94N17O15S2(+1)	1368.7	684.83	5.73	77.36%
GCRP	b4	C31H50N9O8S2(+1)	740.32	740.32	3.73	77.34%
GCRPKPQQF	b9	C61H94N17O15S2(+1)	1368.7	1368.7	10.05	75.81%
GC	b2	C20H31N4O6S2(+1)	487.17	487.17	2.19	73.97%
GCRPKPQQFF	a10	C69H103N18O15S2(+1)	1487.7	744.37	4.86	73.31%
GCRPKPQQFF	b10	C70H103N18O16S2(+1)	1515.7	758.37	14.72	73.02%
GCRPKPQQFF	b10	C70H103N18O16S2(+1)	1515.7	1515.7	1.66	72.90%
GCRPKPQQFFGL	b12	C78H117N20O18S2(+1)	1685.8	843.42	48.96	72.66%
GCRPKPQQFFGL	a12	C77H117N20O17S2(+1)	1657.8	829.42	20.74	70.80%

ENPEGILDC(biotin)HVQRVM-NH$_2$ 10d

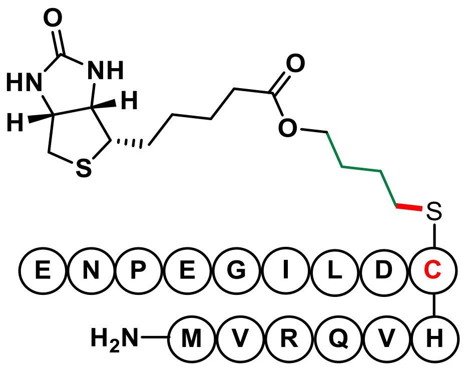

Following the **General procedure**, the desired product **10d** was isolated as colorless solid by RP-HPLC with **Method 1.** The isolated yield was not reported due to the limited reaction scale.
HPLC-UV chromatogram (210 nm) of the crude with **6ca** by **Method 1**:
HPLC-UV yield: 71%.

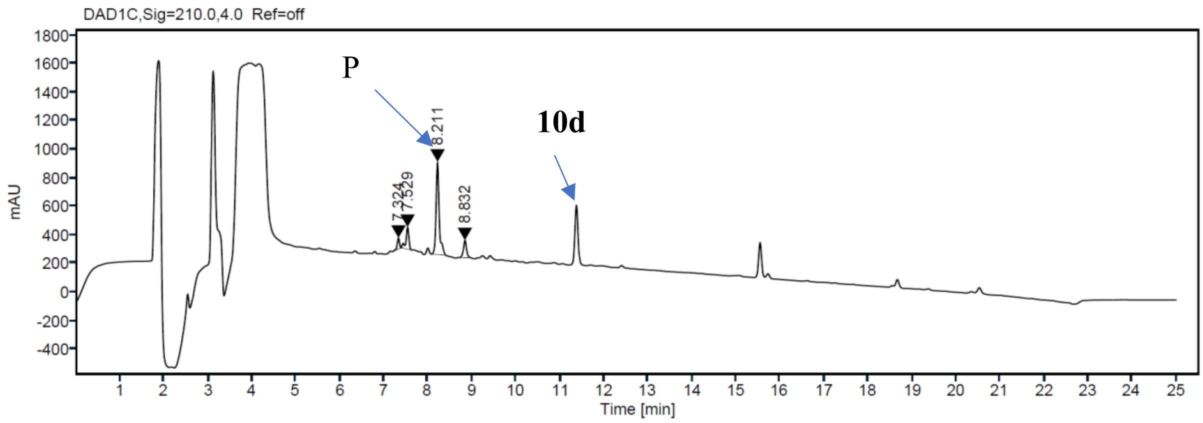

HPLC-UV chromatogram (210 nm) of **10d** by **Method 1**:

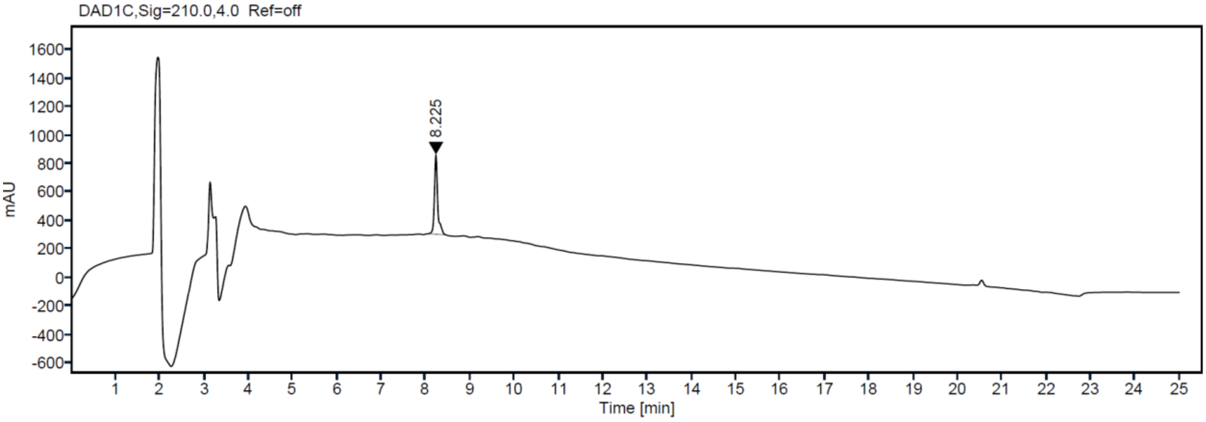

Retention time: 8.238 min **Area Percent:** 100%

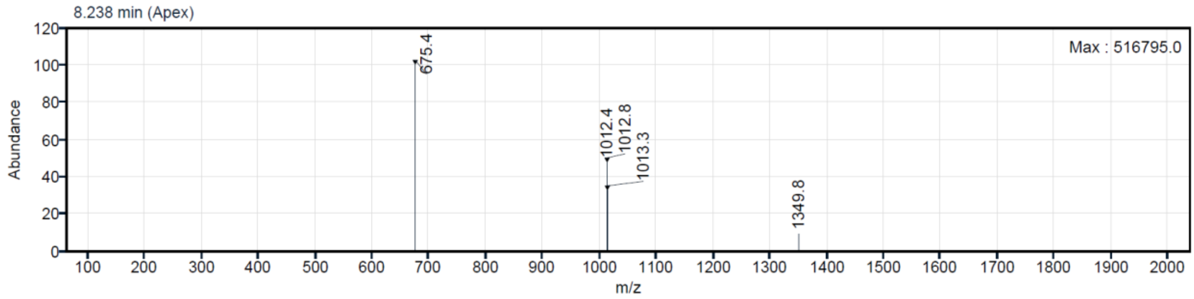

HRMS (LTQ-Orbitrap) m/z: $[M + H_3]^{+3}$ Calcd for $C_{85}H_{142}N_{25}O_{26}S_3{}^{+3}$ 674.9901; Found 674.9927.

MS/MS fragmentation of **10d**:

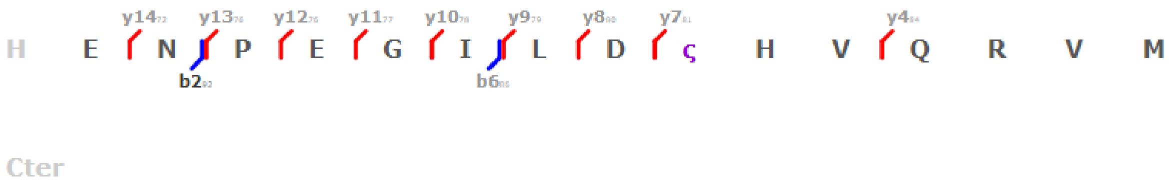

ς = Cys(C13H20N2O3S)
Cter = NH2

Sequence	Type	MF	MF Mass	m/z	Intensity	Similarity
EN	b2	C9H14N3O5(+1)	244.09	244.09	1.98	92.46%

ENPEGI	b6	C27H42N7O11(+1)	640.29	640.29	1.34	86.50%
QRVM	y4	C21H42N9O5S(+1)	532.3	532.3	1.16	83.83%
CHVQRVM	y7	C48H83N16O11S3(+1)	1155.6	578.28	8.2	80.79%
DCHVQRVM	y8	C52H88N17O14S3(+1)	1270.6	635.8	14.69	80.06%
LDCHVQRVM	y9	C58H99N18O15S3(+1)	1383.7	692.34	68.85	79.27%
ILDCHVQRVM	y10	C64H110N19O16S3(+1)	1496.8	748.88	6.88	77.81%
GILDCHVQRVM	y11	C66H113N20O17S3(+1)	1553.8	777.39	24.67	77.44%
EGILDCHVQRVM	y12	C71H120N21O20S3(+1)	1682.8	841.91	4.13	75.90%
PEGILDCHVQRVM	y13	C76H127N22O21S3(+1)	1779.9	890.44	51.7	75.84%
PEGILDCHVQRVM	y13	C76H127N22O21S3(+1)	1779.9	593.96	100.18	75.73%
NPEGILDCHVQRVM	y14	C80H133N24O23S3(+1)	1893.9	631.98	1.05	72.49%

8.3 Aryl azide excitation enabled by CzPN-peptide conjugates with blue light

8.3.1 Aryl azide excitation on small molecule

Procedure: Modified based on a reported procedure,[10] Azide **S6** (17.7 mg, 0.100 mmol, 1 equiv.) and CzPN conjugates **3za** and **6aa** (0.005 mmol, 5 mol%) were weighed in a vial. n-butylamine (50 µL, 0.50 mmol, 5.0 equiv.) and MeOH (0.5 mL, 0.1 M) were added under nitrogen. The vial was placed on a stirring plate and irradiated (Kessil lamp 467 nm, intensity 100%) for 12 h. The crude was concentrated and analyzed by ^{1}H-NMR (CH_2Br_2 as internal standard).

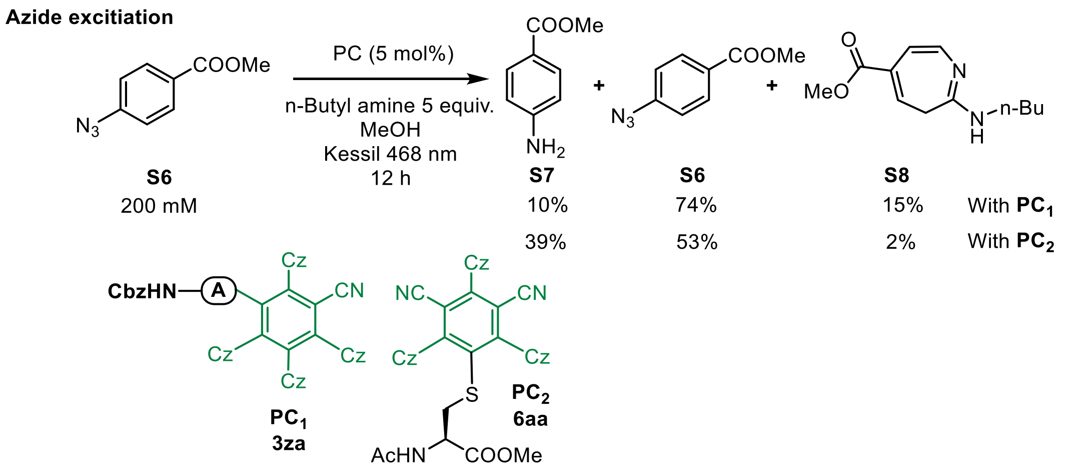

NMR crude of aryl azide excitation with **3za**

[10] Tay, N. E. S. *et al.* Targeted activation in localized protein environments via deep red photoredox catalysis. *Nat. Chem.* **15**, 101-109, (2023).

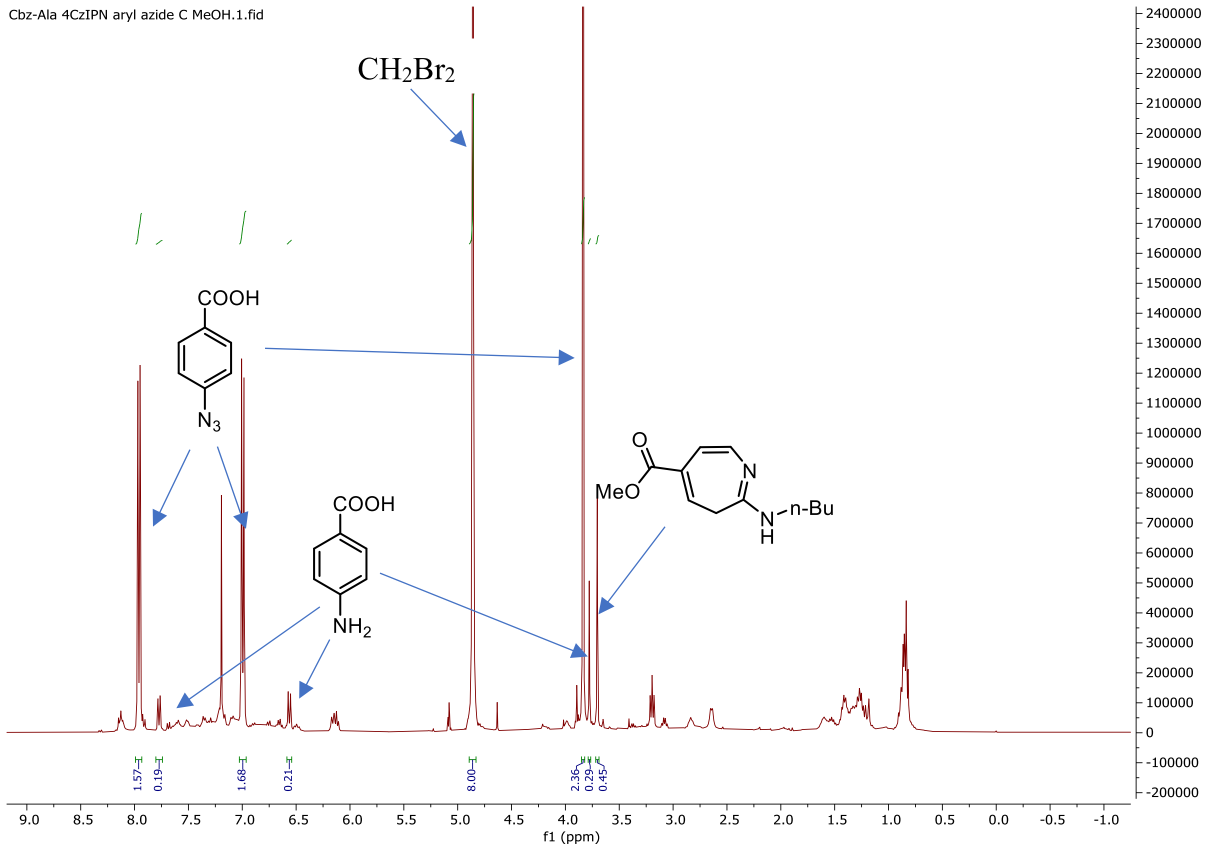

NMR crude of aryl azide excitation with **6aa**

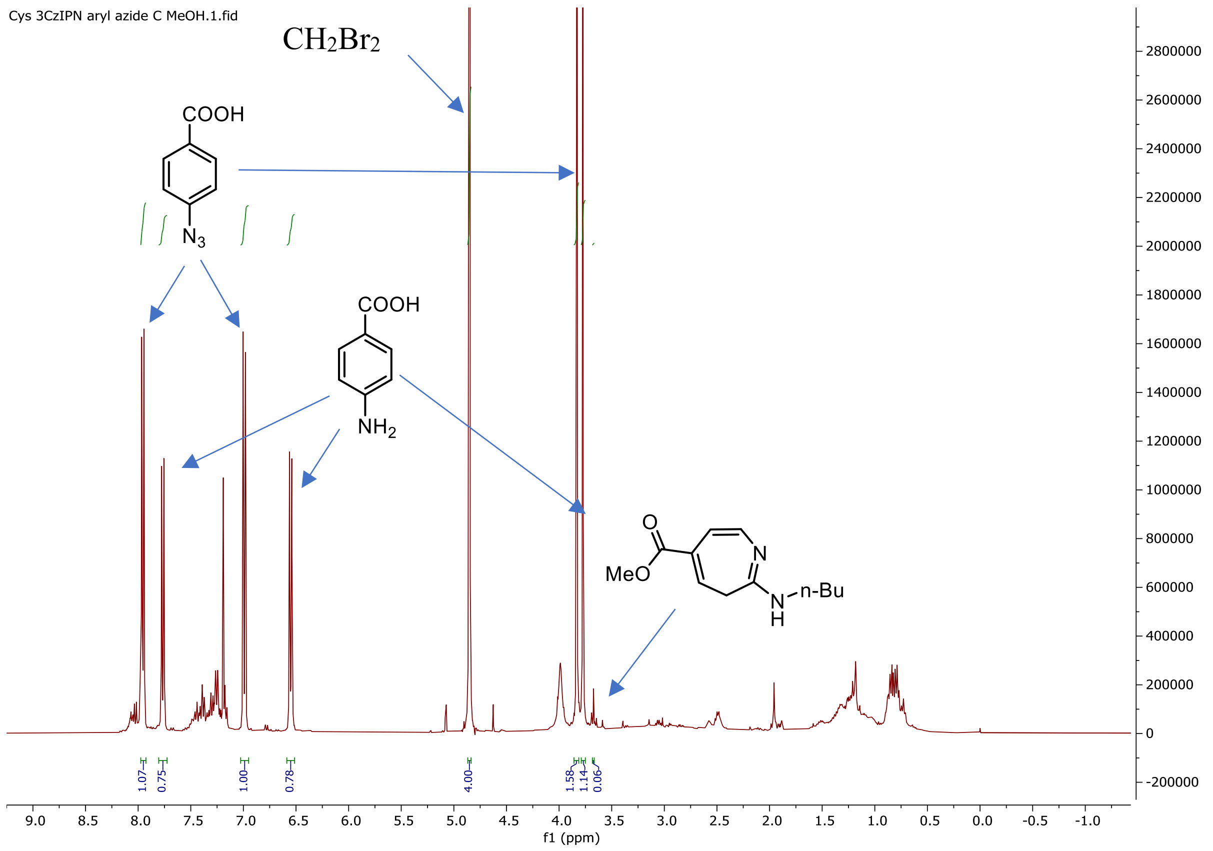

8.3.2 Fluoroaryl azide excitation on small molecule

Azide **S8** (17.7 mg, 0.100 mmol, 1 equiv.) and CzPN conjugates **3za** and **6aa** (0.005 mmol, 5 mol%) were weighed in a vial. THF/Water 1:1 (0.5 mL, 0.1 M) was added under nitrogen. The vial was placed on a stirring plate and irradiated (Kessil lamp 467 nm, intensity 100%) for 12 h. The crude was concentrated and analyzed by ^{19}F-NMR.

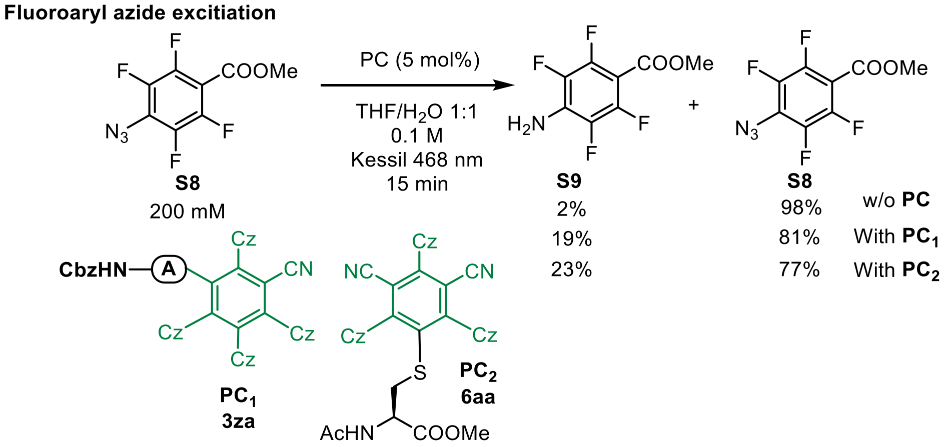

^{19}F-NMR crude of aryl azide excitation without photocatalyst:

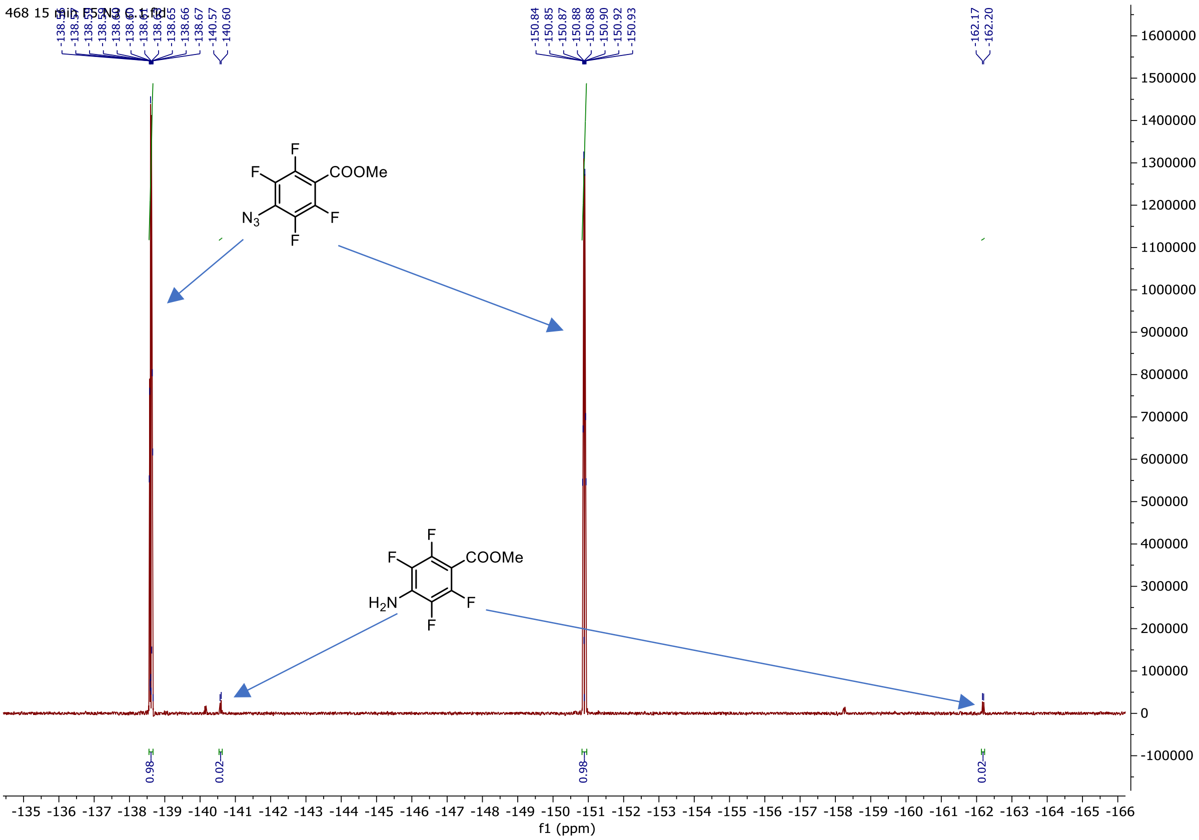

^{19}F-NMR crude of aryl azide excitation with **3za**:

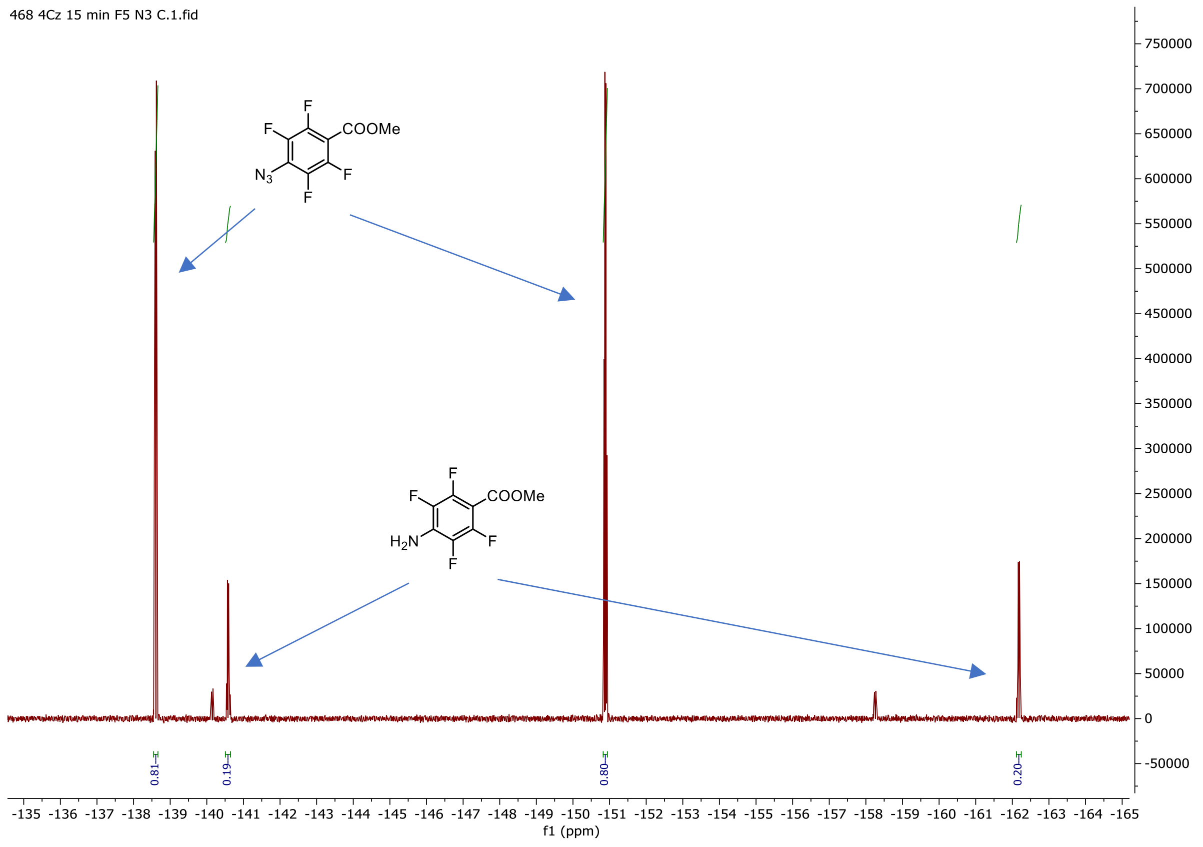

^{19}F-NMR crude of aryl azide excitation with **6ca**:

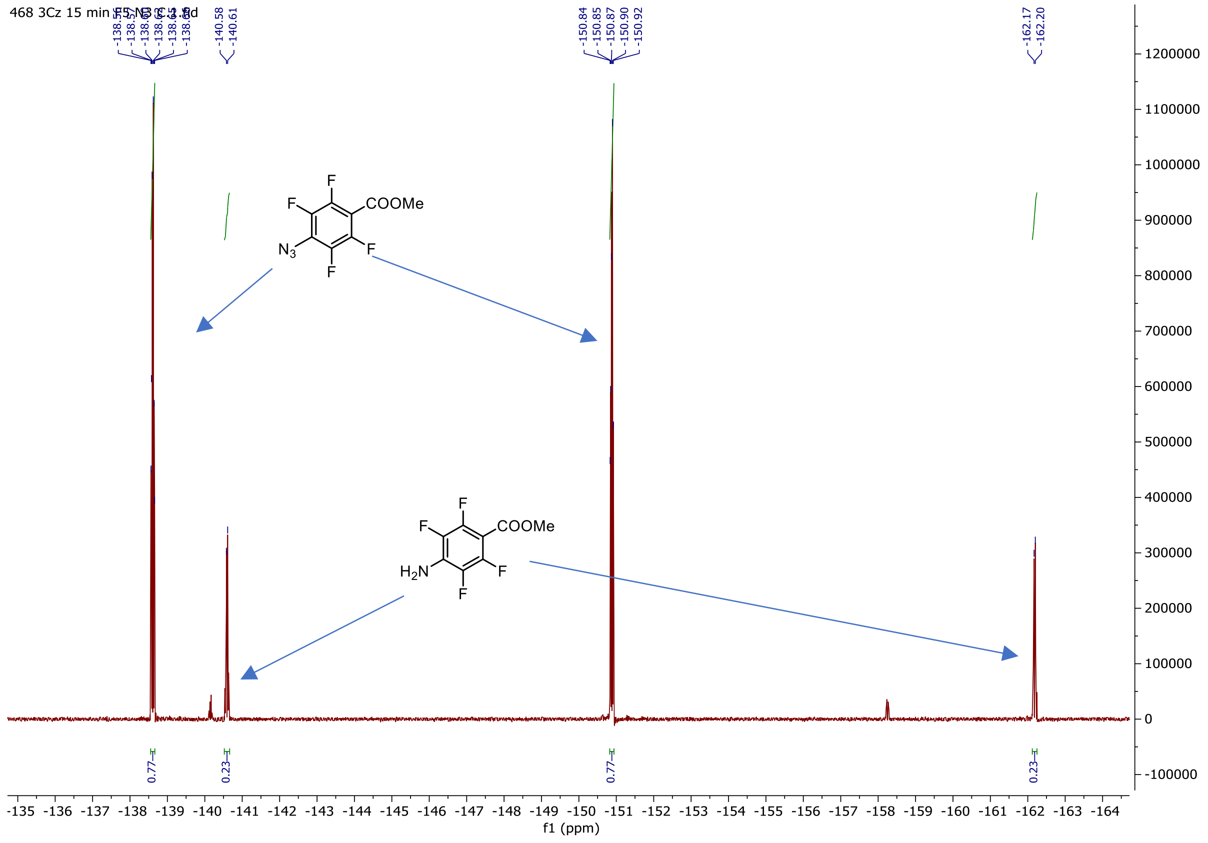

The observation of aniline as the major product, rather than the ring-expanded azepine, from both aryl azide and fluoroaryl azide under excitation with CzBN or 3CzIPN suggests the formation of a triplet nitrene intermediate. This outcome is consistent with previous reports on triplet nitrene generation.[10] In contrast to previous Ir (E_T = 60 kcal/mol) or Os (E_T = 45 kcal/mol) photocatalyst system, CzBN(E_T = 67.6 kcal/mol)/3CzIPN(E_T = 61.1 kcal/mol) possess higher excited state energies. These elevated energy levels are sufficient to promote direct triplet-triplet EnT to aryl azide (E_T around 63 kcal/mol). Although previous studies have indicated that aryl azide excitation likely proceeds via an electron transfer (ET) mechanism, the higher triplet energies of our photocatalysts suggest that the EnT pathway cannot be ruled out in our system.

8.3.3 Stern-Volmer quenching between CzPN-peptide conjugates and aryl azide

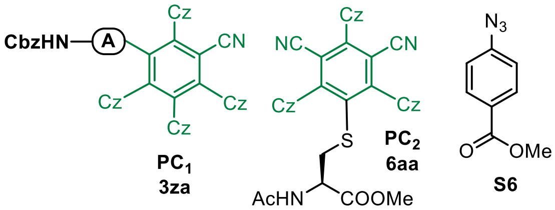

Solutions containing **3za**/**6aa** (20.0 µM) and aryl azide **S6** (0–50 mM) were prepared in DMSO in 1.0 cm path-length quartz fluorescence cuvettes equipped with septum screwcaps. The solutions were sparged for 15 minutes with N_2. Emission spectra were recorded with excitation at 380 nm, and emission intensity at the peak maximum was recorded.

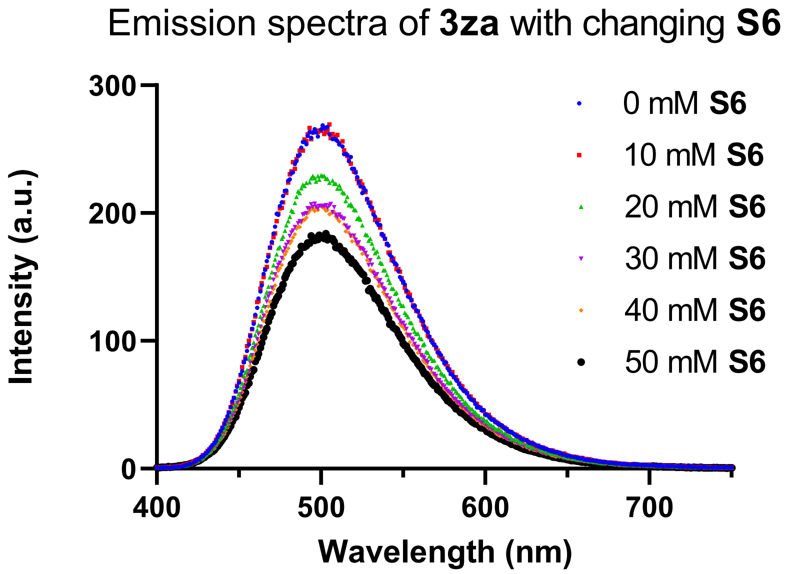

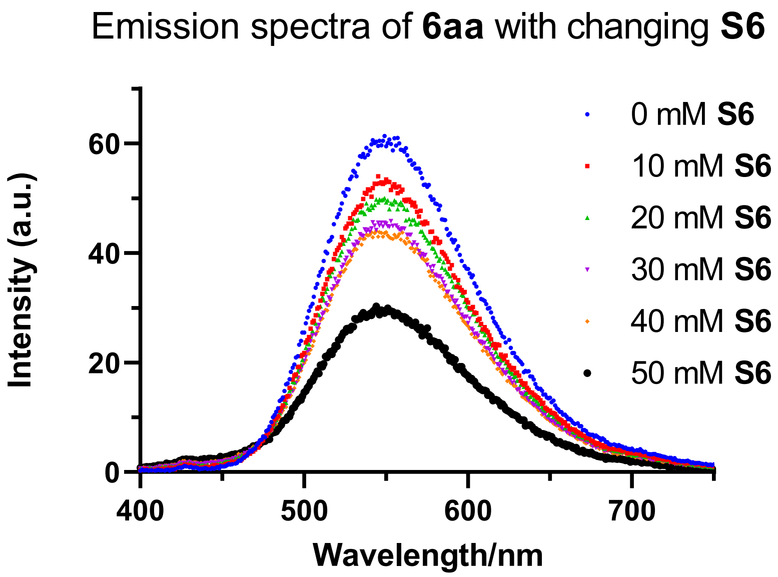

Increasing the concentration of **S6** quenches emission from **3za/6aa** in DMSO, reflecting an increase in nonradiative decay of the excited state of **3za/6aa** to **S6**.

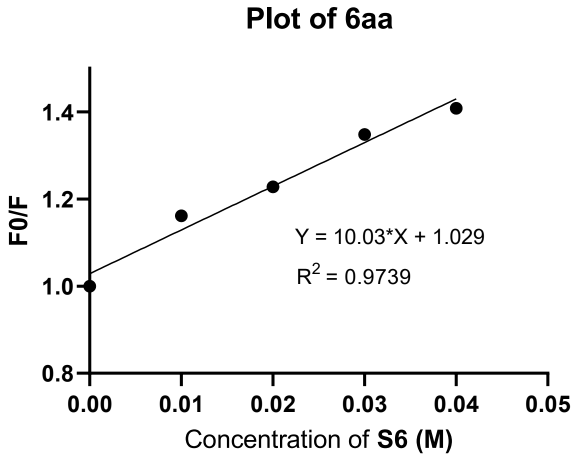

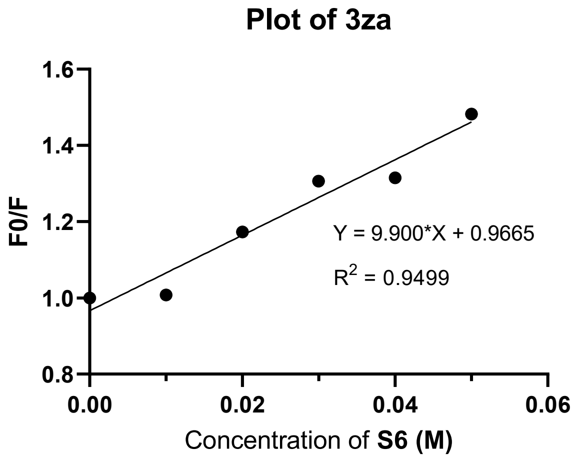

Figure S4. Stern-Volmer plot

8.4 Synthesis of aryl azide probes

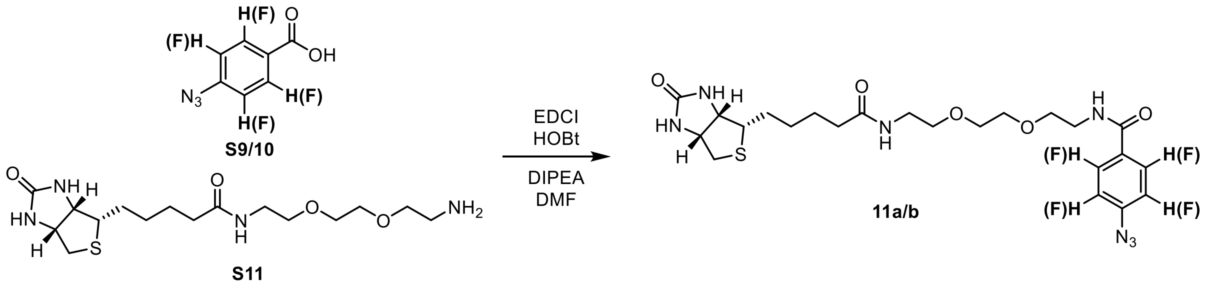

EDCI (36 mg, 0.19 mmol, 1.4 equiv.), N,N-diisopropylethylamine (42 µL, 0.27 mmol, 2.0 equiv.) and HOBt (25.3 mg, 0.19 mmol, 1.4 equiv.) were added to a solution of 4-(fluoro)azidobenzoic acid **S9/S10** (24 mg, 0.15 mmol, 1.1 equiv.) in anhydrous DMF (1.0 mL), and the mixture was stirred at room temperature for 5 min under an Ar atmosphere. Compound **S11** (50 mg, 0.13 mmol, 1 equiv.) in DMF (0.5 mL) was then added to the mixture, and the resulting mixture was stirred overnight at room temperature. The solvent was then removed under reduced pressure, and the residue was purified by RP-HPLC, followed by lyophilization. The desired product **11a** was obtained as colorless solid (42 mg, 0.081 mmol, 61%).

11a: ^{1}H NMR (400 MHz, $CDCl_3$) δ 7.84 (d, *J* = 8.5 Hz, 2H, ArH), 7.07 (d, *J* = 8.6 Hz, 2H, ArH), 4.57 (dd, *J* = 8.0, 4.8 Hz, 1H, BiotinNCH), 4.36 (dd, *J* = 7.9, 4.6 Hz, 1H, BiotinNCH), 3.69 (d, *J* = 4.0 Hz, 1H), 3.68 – 3.62 (m, 5H), 3.56 (t, *J* = 5.1 Hz, 1H), 3.47 – 3.35 (m, 1H), 3.16 (q, *J* = 7.1 Hz, 1H), 2.93 (dd, *J* = 13.1, 4.8 Hz, 1H), 2.78 (d, *J* = 13.0 Hz, 1H), 1.76 – 1.57 (m, 2H), 1.43 (p, *J* = 7.4 Hz, 1H).

The **^{1}H NMR** shift are consistent with reported data.[11]

11b: ^{1}H NMR (400 MHz, $CDCl_3$) δ 4.54 – 4.48 (m, 1H, BiotinNCH), 4.33 – 4.27 (m, 1H, BiotinNCH), 3.73 – 3.60 (m, 8H), 3.56 (t, *J* = 5.7 Hz, 2H), 3.51 – 3.43 (m, 1H), 3.41 – 3.31 (m, 1H), 3.19 – 3.12 (m, 1H), 2.92 (d, *J* = 11.6 Hz, 1H), 2.73 (d, *J* = 12.8 Hz, 1H), 2.19 (t, *J* = 7.3 Hz, 2H), 1.70 – 1.63 (m, 4H), 1.47 – 1.37 (m, 2H).

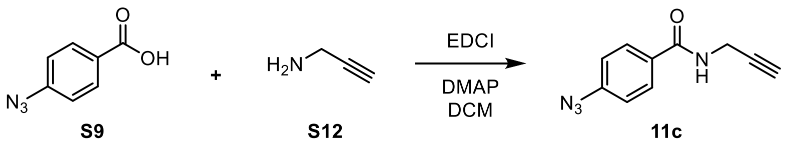

To a solution of 4-azidobenzoic acid **S9** (489 mg, 3.00 mmol, 1 equiv.) dissolved in DCM (18 mL) were successively added propargylamine **S12** (0.477 mL, 6.60 mmol, 2.20 equiv.), EDCI (559 mg, 3.60 mmol, 1.20 equiv.), and DMAP (36.7 mg, 0.300 mmol, 0.100 equiv.) at room temperature. After stirring for 15 h, the mixture was concentrated under reduced pressure. After the addition of H_2O (20 mL), the mixture was extracted with Et_2O (30 mL × 3), and the combined organic was washed with brine (20 mL), dried (Na_2SO_4), and after filtration, the filtrate was concentrated under reduced pressure. The residue was purified by flash column chromatography (pentane/EtOAc = 1/1) to give the desired product **11c** as colorless solid (513 mg, 2.56 mmol, 85% yield).

^{1}H NMR (400 MHz, $CDCl_3$) δ 7.81 (d, *J* = 8.6 Hz, 2H, ArH), 7.10 (d, *J* = 8.6 Hz, 2H, ArH), 6.23 (s, 1H, NH), 4.28 (dd, *J* = 5.2, 2.6 Hz, 1H, CH_2), 2.32 (t, *J* = 2.6 Hz, 1H, alkyne CH).

The **^{1}H NMR** shift are consistent with reported data.[12]

---

[11] For **11a**: Chiba, K.; Asanuma, M.; Ishikawa, M.; Hashimoto, Y.; Dodo, K.; Sodeoka, M.; Yamaguchi, T., *Chem. Commun.* **2017,** *53* (62), 8751-8754. For **11b**: Ref 8.

[12] Meguro, T.; Yoshida, S.; Igawa, K.; Tomooka, K.; Hosoya, T., *Org. Lett.* **2018,** *20* (13), 4126-4130.

8.5 Protein labeling in-vitro via aryl azide excitation

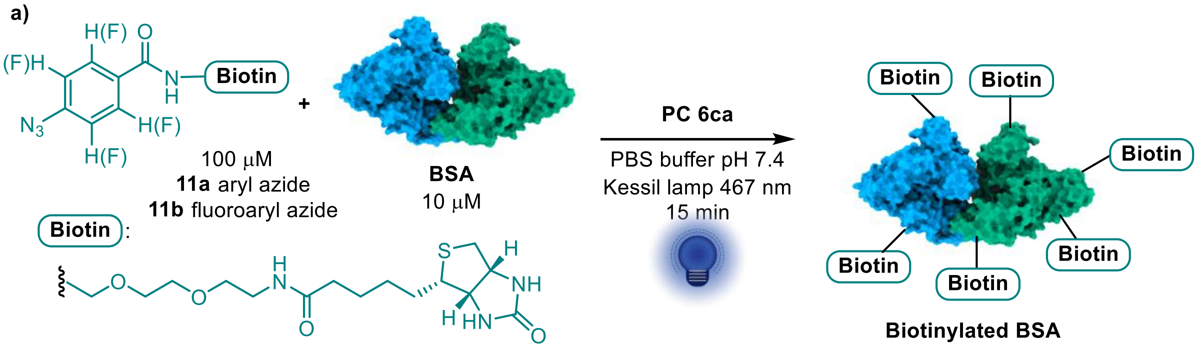

General procedure:

To a solution of 10 µM bovine serum albumin (BSA) in PBS buffer (pH 7.4) in 2 mL vial was added the biotin probe **11a/b** (100 µM, 50 mM stock solution in DMSO), CzPN-peptide conjugates **6ca** (20 mM stock solution in DMSO), in a total volume 1 mL. The mixture was irradiated with a 467 nm Kessil lamp (intensity: 100%) for 15 min. 30 µL samples were then removed, combined with 10 µL of 4x reducing Laemmli sample buffer (5% ß-mercaptoethanol), vortexed, and heated at 95 °C for 10 minutes. 10 µL of each sample was then analyzed by Western blot.

8.6 General Western blot procedure:

The samples were loaded on SDS-polyacrylamide gel (7.5% acrylamide) electrophoresis (PAGE) at 180 V for 60 min. After electrophoresis, gels were transferred from PVDF membranes using an iBlot transfer device, and washed with water. The membrane was blocked with 4% BSA in TBST at 4 °C for 1 hour, followed by TBST wash 3 times. The blots were incubated with streptavidin-HRP (Proteintech) (dilute 2000 times) in TBST at room temperature for 60 minutes, then washed with TBST 3×10 minutes before development with SuperSignal West Pico reagent (Thermo Scientific) and the image was obtained with a ChemiDoc XRS System and Image Lab Software (Bio-Rad). The duplicated PAGE gels were stained with Coomassie brilliant blue (CBB) and the image obtained with the ChemiDoc XRS System and Image Lab Software.

Uncropped gel:

Aryl azide WB:

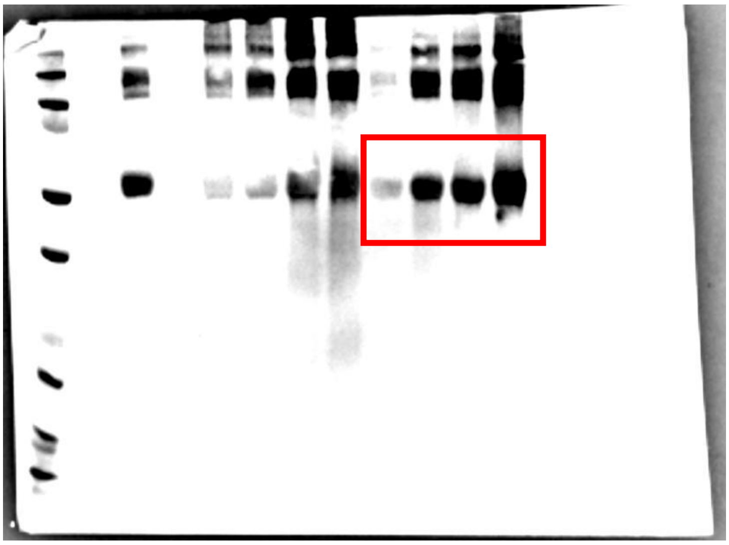

Coomassie:

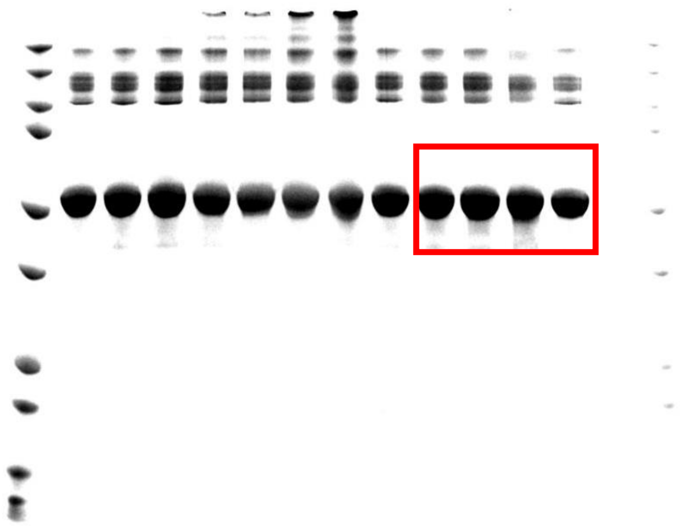

Fluoroaryl azide WB:

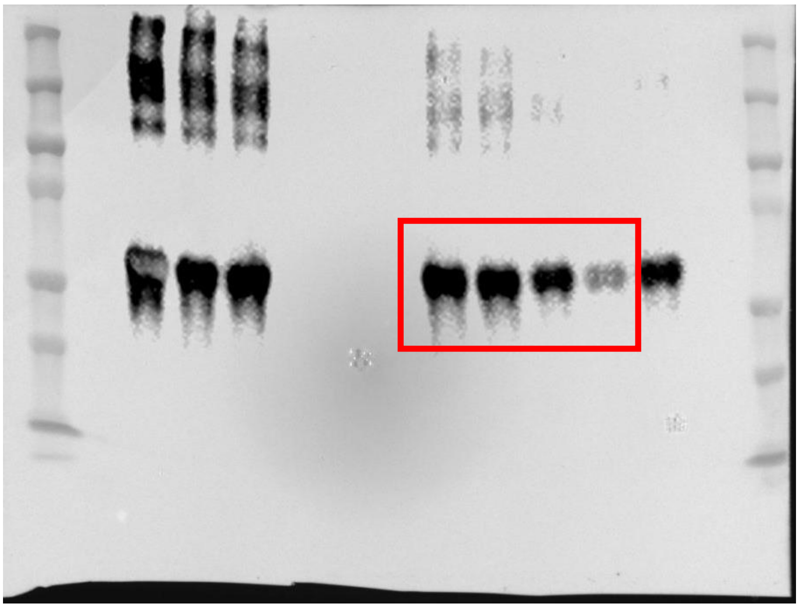

Coomassie:

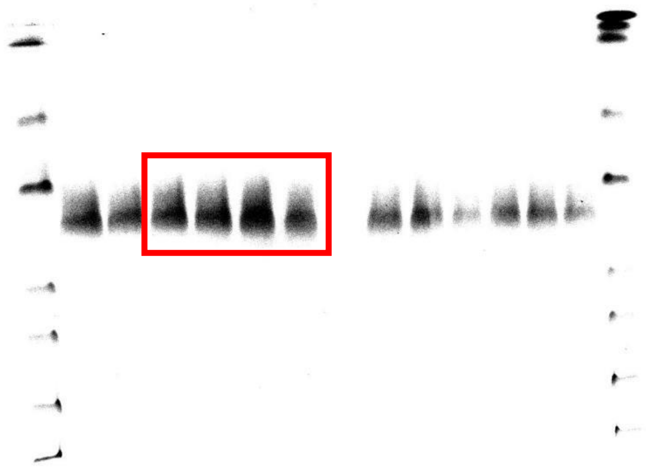

8.7 Live-cell protein labelling via aryl azide excitation

8.7.1 Cell line stocks

Hela and A549 cell lines were obtained from the European Collection of Animal Cell Cultures (ECACC, United Kingdom) and they were grown in complete medium (DMEM) supplemented with 10% fetal bovine serum (FBS) and maintained in an incubator at 37 °C in an atmosphere of 5% CO_2. Hela cells were seeded on poly-lysine coated 8-well glass bottom cell culture plates at a density of 10’000 cells/well the day before the experiment. The fluorescent signal was detected using an Eclipse Ti2 microscope with a Yokogawa CUS W2 confocal spinning disk unit and equipped with a Prime 95B sCMOS camera (Photometrics). We used a 60x oil immersion objective with a N.A. of 1.40 to observe cells. Imaging was performed using the following parameters: peptide-CzPN conjugates (excited with a 405 nm laser and captured with a 447/60 nm bandpass filter), FITC-integrin $\alpha_v\beta_3$ monoclonal antibody (excited with a 488 nm

laser and captured with a 542/27 nm bandpass filter) and Cy5 (excited with a 638 nm laser and captured with a 600/52 nm bandpass filter). Image analysis was performed using Fiji/ImageJ.[13]

8.7.2 Workflow for aryl azide excitation

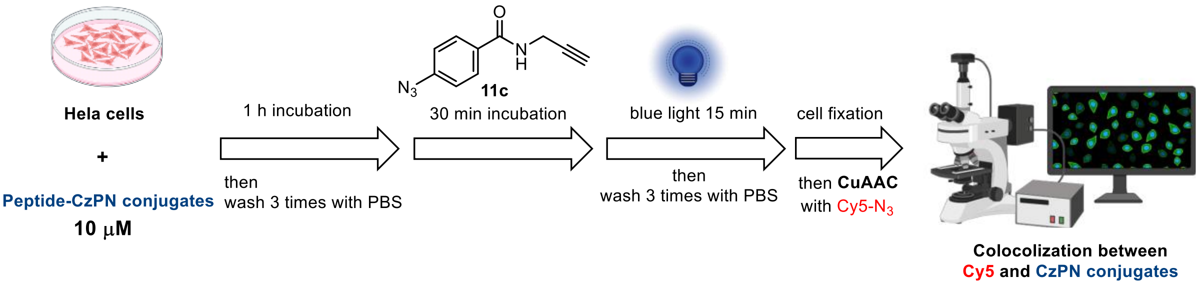

Excitation of aryl azide (100 μM) with CzPN-peptide conjugates (10 μM) at the cellular level. Scale bar: 10 μM.

For the experiment, the medium was exchanged to fresh medium containing 10 μM of the indicated peptide (stock solution was prepared to a concentration of 10 mM in DMSO). Hela cells were incubated for 1 hour. After that, the medium was removed and cells were washed with 3 times*PBS. Then the cell was treated with the aryl azide probe **11c** (100 μM) for 30 min. The dish was irradiated with the 440/468 nm Kessil lamp (10 cm from the top of the dish, 100% intensity) at room temperature for 15 min. After the photolabeling, cells were washed with PBS for 3 times, followed by fixation with 4% paraformaldehyde in PBS at room temperature for 15 min. The fixed cells were washed with PBS for 3 times and permeabilized with 0.2% Triton X-100 in PBS at room temperature for 15 min. After 3 times PBS washing, the fixed cells were blocked with 4% BSA in PBS at room temperature for 30 min. A click solution including 50 μM $CuSO_4$, 250 μM THPTA, 2.5 mM sodium ascorbate and 20 μM Cy5-N_3 was added into the dish and incubated at room temperature for 30 min, followed by PBS washing for 3 times. The signal was detected using an Eclipse Ti2 microscope with a Yokogawa CUS W2 confocal spinning disk unit and equipped with a Prime 95B sCMOS camera (Photometrics). We used a 60x oil immersion objective with a N.A. of 1.40 to observe cells at room temperature.

For co-localization studies, imaging was performed using the following parameters: CzPN-peptide conjugates (excited with a 405 nm laser and captured with a 447/60 nm bandpass filter), Cy5 (excited with a 638 nm laser and captured with a 600/52 nm bandpass filter). Image analysis was performed using Fiji/ImageJ. For quantitative image analysis, the following threshold were used: CzPN-peptide channel: 110, Cy5 channel: 137, an area for analysis was selected with the “create selection” function and fluorescent intensity of the whole image was measured.

Without peptide-CzPN conjugates (scale bar 10 μm)

[13] Schindelin, J.; Arganda-Carreras, I.; Frise, E.; Kaynig, V.; Longair, M.; Pietzsch, T.; Preibisch, S.; Rueden, C.; Saalfeld, S.; Schmid, B.; Tinevez, J.-Y.; White, D. J.; Hartenstein, V.; Eliceiri, K.; Tomancak, P.; Cardona, A., Fiji: an open-source platform for biological-image analysis. *Nat. Meth.* **2012,** *9* (7), 676-682.

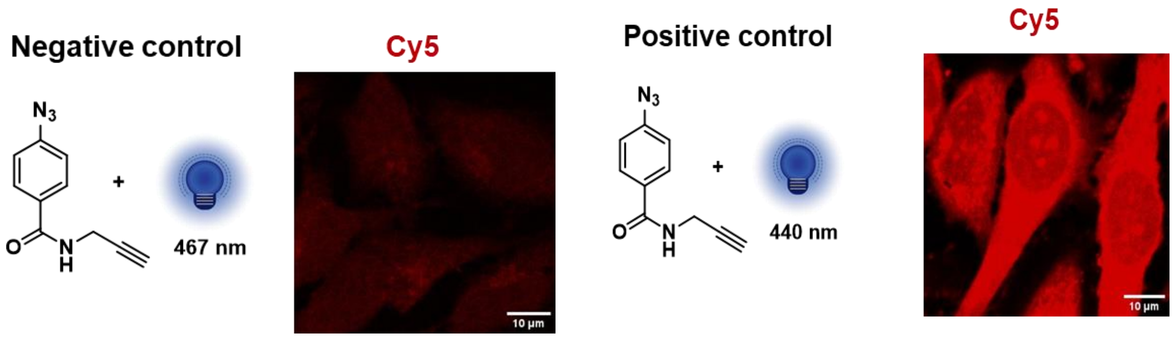

With peptide-CzPN conjugate 6fa

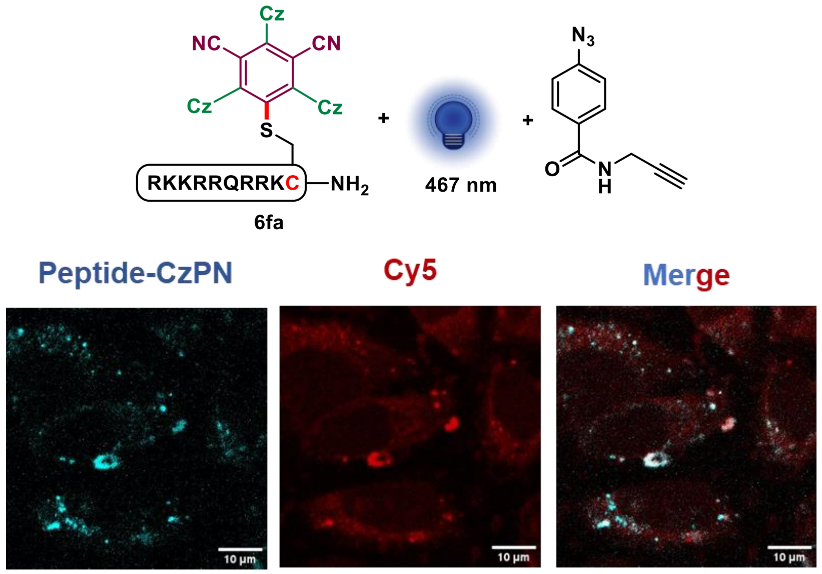

Figure S5: Cell imaging after aryl azide excitation

8.7.3 Labelling of integrin avb3 via aryl azide excitation

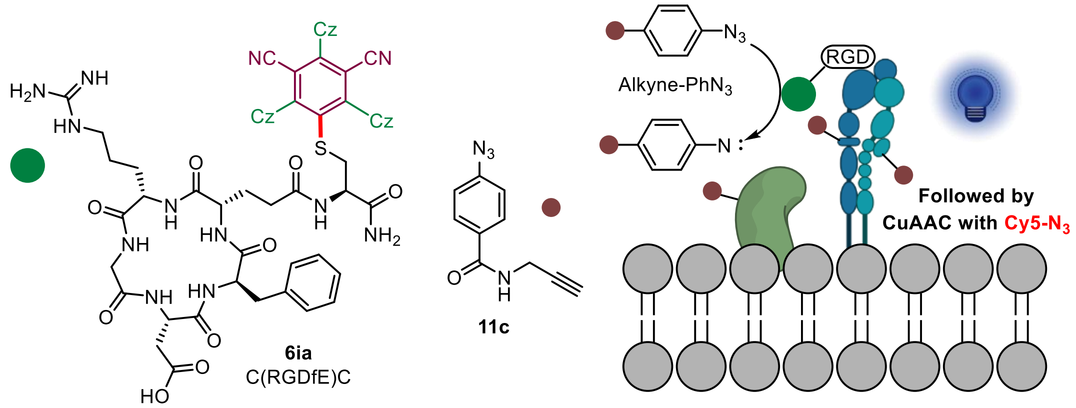

For the experiment, the medium was exchanged to fresh medium containing 10 μM of the **6ia** (stock solution was prepared to a concentration of 10 mM in DMSO). A549 cells were incubated for 1 hour. After that, the medium was removed and cells were washed with 3 times*PBS. Then the cell was treated with the aryl azide probe **11c** (100 μM) for 30 min. The dish was irradiated with the 468 nm Kessil lamp (10 cm from the top of the dish, 100% intensity) at room temperature for 15 min. After the photolabeling, cells were washed with PBS for 3 times, followed by fixation with 4% paraformaldehyde in PBS at room temperature for 15 min.

The fixed cells were washed with PBS for 3 times and permeabilized with 0.2% Triton X-100 in PBS at room temperature for 15 min. After 3 times PBS washing, the fixed cells were blocked with 4% BSA in PBS at room temperature for 30 min. A click solution including 50 mM CuSO4, 250 mM THPTA, 2.5 mM sodium ascorbate and 20 mM Cy5-N3 was added into the dish and incubated at room temperature for 30 min, followed by PBS washing for 3 times. The cell was incubated with FITC-integrin $\alpha_v\beta_3$ monoclonal antibody (Thermofisher, dilute for 1000 times) overnight, followed by PBS washing for 3 times. The signal was detected using an Eclipse Ti2 microscope with a Yokogawa CUS W2 confocal spinning disk unit and equipped with a Prime 95B sCMOS camera (Photometrics). We used a 60x oil immersion objective with a N.A. of 1.40 to observe cells at room temperature. For quantitative image analysis, the following threshold were used: FITC-integrin $\alpha_v\beta_3$ monoclonal antibody channel: 741. Cy5 channel: 109. Pearson's coefficient for FITC-integrin $\alpha_v\beta_3$ monoclonal antibody and Cy5: 0.628.

Control Experiment in Integrin αvβ3–Deficient HeLa Cells

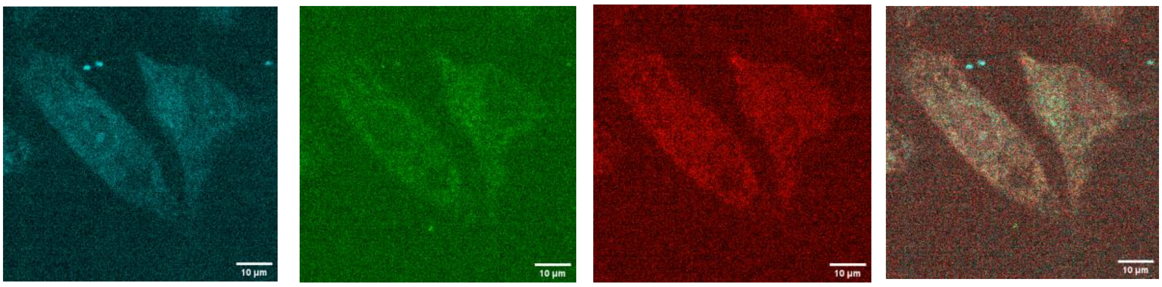

Figure S6: Peptide-3CzIPN: 6ia (10 μM, 1 h), FITC: FITC-integrin αvβ3 monoclonal antibody (overnight), scale bar: 10 μm

Minimal fluorescence signal was observed in both the antibody and Cy5 channels, supporting the specificity of our probe **6ia** for integrin αvβ3.

8.7.4 MTT assay

Cells were seeded into 96-well tissue culture plates with the cell density around 10000 cells per well, and incubated overnight. Then cells in each well were incubated with complete medium containing different CzPN-peptide conjugates **6fa/6ca** (10 μM). Control cells were incubated with complete medium containing 0.1% of DMSO and azide probe **11c**. After 1 hour of incubation, the wells incubated with CzPN conjugates were washed with PBS for 3 times, followed by incubation of aryl azide for 30 min. The 96-well was placed under 468 nm Kessil lamp or in the dark for 15 min. Each well was washed with PBS for 3 times, then 10 μL of 3-(4,5-dimethylthiazol-2-yl)-2,5-diphenyltetrazolium bromide (MTT) solution was added to the 100 μL of medium in each well, and the plates were incubated for 4 hours, 200 μL of DMSO was added directly into the medium in each well and pipette up and down several times to dissolve the formazan salt. The absorbance signal was measured on a spectrophotometer at 570 nm (background absorbance at 630 nm) The signal of absorbance was obtained by the subtraction of both values. The absorbance of each sample was normalized with its DMSO control ($A_{\text{treated cells}}$ / A_{blank}) × 100%.

With Hela cells and **6fa**

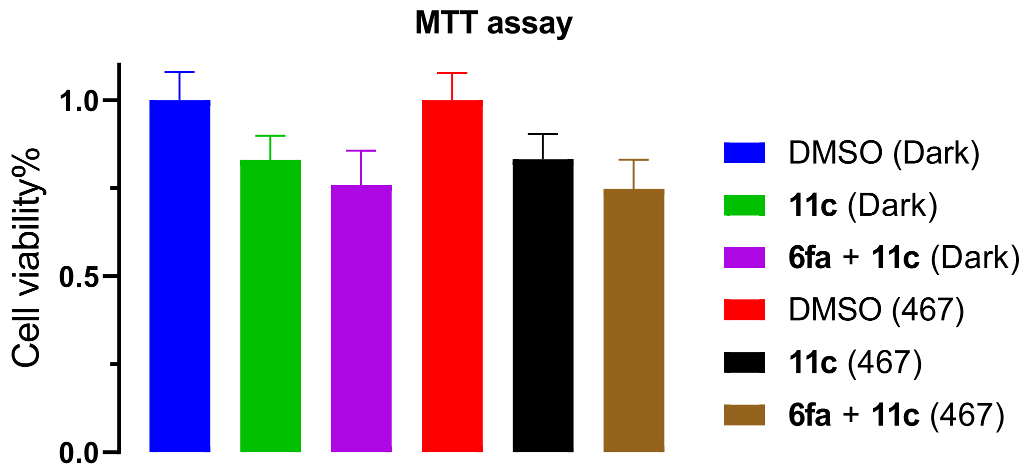

With A549 cells and **6ia**

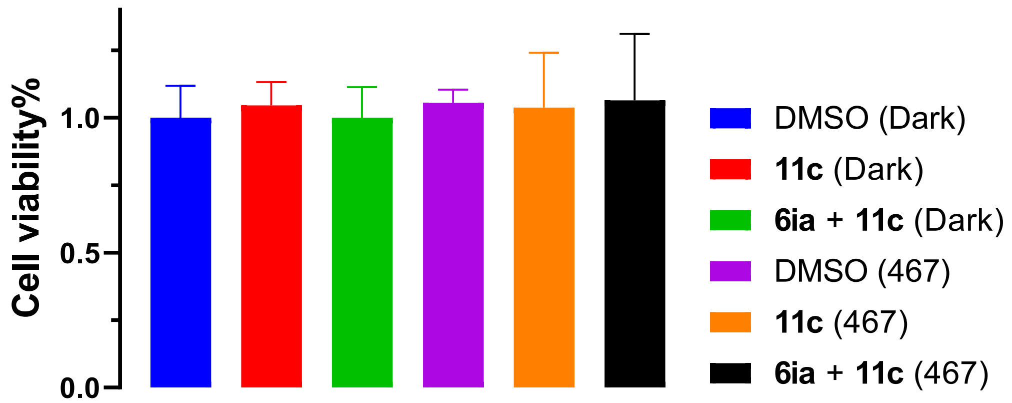

Figure S7: Cell viability before and after aryl azide excitation

9. X-Ray crystallographic data of 3za

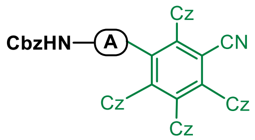

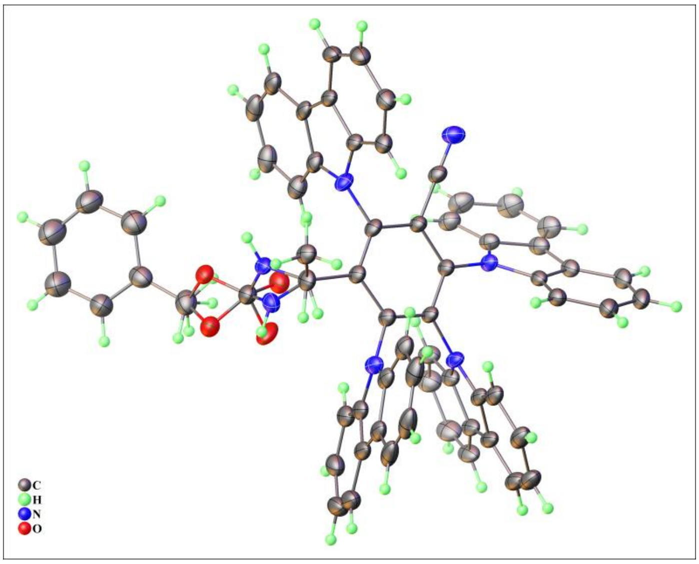

Experimental. Single clear intense yellow prism-shaped crystals of **3za** were used as supplied. A suitable crystal with dimensions 0.17 × 0.07 × 0.04 mm^3 was selected and mounted on a XtaLAB Synergy R, DW system, HyPix-Arc 150 diffractometer. The crystal was kept at a steady *T* = 139.99(10) K during data collection. The structure was solved with the **ShelXT** 2018/2 (Sheldrick, 2015) solution program using dual methods and by using **Olex2** 1.5 (Dolomanov et al., 2009) as the graphical interface. The model was refined with **ShelXL** 2018/3 (Sheldrick, 2015) using full matrix least squares minimisation on $\boldsymbol{F}^2$.

Crystal Data. $C_{65}H_{44}N_6O_2$, M_r = 941.06, monoclinic, *P*2$_1$/*c* (No. 14), a = 14.3978(4) Å, b = 20.1347(4) Å, c = 20.8012(3) Å, □ = 104.402(2)°, □ = □ = 90°, *V* = 5840.7(2) Å^3, *T* = 139.99(10) K, *Z* = 4, *Z'* = 1, □(Cu K□) = 0.515, 84455 reflections measured, 11695 unique (R_{int} = 0.0411) which were used in all calculations. The final wR_2 was 0.1991 (all data) and R_1 was 0.0647 (I≥2 □(I)).

**Compound**	**3za**
Formula	$C_{65}H_{44}N_6O_2$
$D_{calc.}$/ g cm^{-3}	1.070
□/mm^{-1}	0.515
Formula Weight	941.06
Colour	clear intense yellow
Shape	prism-shaped
Size/mm^3	0.17×0.07×0.04
T/K	139.99(10)
Crystal System	monoclinic
Space Group	*P*2$_1$/*c*
a/Å	14.3978(4)
b/Å	20.1347(4)
c/Å	20.8012(3)
□/°	90
□/°	104.402(2)
□/°	90
V/Å^3	5840.7(2)
Z	4
Z'	1
Wavelength/Å	1.54184
Radiation type	Cu*K*□
□$_{min}$/°	3.103
□$_{max}$/°	74.902
Measured Refl's.	84455
Indep't Refl's	11695
Refl's I≥2□(I)	7505
R_{int}	0.0411
Parameters	688
Restraints	204
Largest Peak/e Å^{-3}	0.424
Deepest Hole/e Å^{-3}	-0.371
GooF	1.037
wR_2 (all data)	0.1991
wR_2	0.1795
R_1 (all data)	0.0967
R_1	0.0647
CCDC number	2141925

Structure Quality Indicators

**Reflections:**	d min (Cu\a) 2Θ=149.8° 0.80	I/σ(I) CIF 27.9	Rint CIF 4.11%	Full 135.4° 97% to 149.8° 99.9
**Refinement:**	Shift CIF 0.000	Max Peak CIF 0.4	Min Peak CIF -0.4	GooF CIF 1.037

A clear intense yellow prism-shaped-shaped crystal with dimensions 0.17 × 0.07 × 0.04 mm^3 was mounted. Data were collected using a XtaLAB Synergy R, DW system, HyPix-Arc 150 diffractometer operating at T = 139.99(10) K.

Data were measured using □ scans with Cu K□ radiation. The diffraction pattern was indexed and the total number of runs and images was based on the strategy calculation from the program CrysAlisPro 1.171.41.122a (Rigaku OD, 2021). The maximum resolution achieved was □ = 74.902° (0.80 Å).

The unit cell was refined using CrysAlisPro 1.171.41.122a (Rigaku OD, 2021) on 18940 reflections, 22% of the observed reflections.

Data reduction, scaling and absorption corrections were performed using CrysAlisPro 1.171.41.122a (Rigaku OD, 2021). The final completeness is 99.90 % out to 74.902° in □. A Gaussian absorption correction was performed using CrysAlisPro 1.171.41.122a (Rigaku Oxford Diffraction, 2021) Numerical absorption correction based on Gaussian integration over a multifaceted crystal model. Empirical absorption correction using spherical harmonics as implemented in SCALE3 ABSPACK scaling algorithm. The absorption coefficient □ of this material is 0.515 mm^{-1} at this wavelength (□ = 1.54184Å) and the minimum and maximum transmissions are 0.849 and 1.000.

The structure was solved and the space group $P2_1/c$ (# 14) determined by the ShelXT 2018/2 (Sheldrick, 2015) structure solution program using using dual methods and refined by full matrix least squares minimisation on $\boldsymbol{F}^2$ using version 2018/3 of **ShelXL** 2018/3 (Sheldrick, 2015). All non-hydrogen atoms were refined anisotropically. Hydrogen atom positions were calculated geometrically and refined using the riding model.

There is a single molecule in the asymmetric unit, which is represented by the reported sum formula. In other words: Z is 4 and Z' is 1.

Citations

CrysAlis^Pro Software System, Rigaku Oxford Diffraction, (2021).

Sheldrick, G.M., ShelXT-Integrated space-group and crystal-structure determination, *Acta Cryst.*, (2015), **A71**, 3-8.

Sheldrick, G.M., Crystal structure refinement with ShelXL, *Acta Cryst.*, (2015), **C71**, 3-8.

O.V. Dolomanov and L.J. Bourhis and R.J. Gildea and J.A.K. Howard and H. Puschmann, **Olex2**: A complete structure solution, refinement and analysis program, *J. Appl. Cryst.*, (2009), **42**, 339-341.

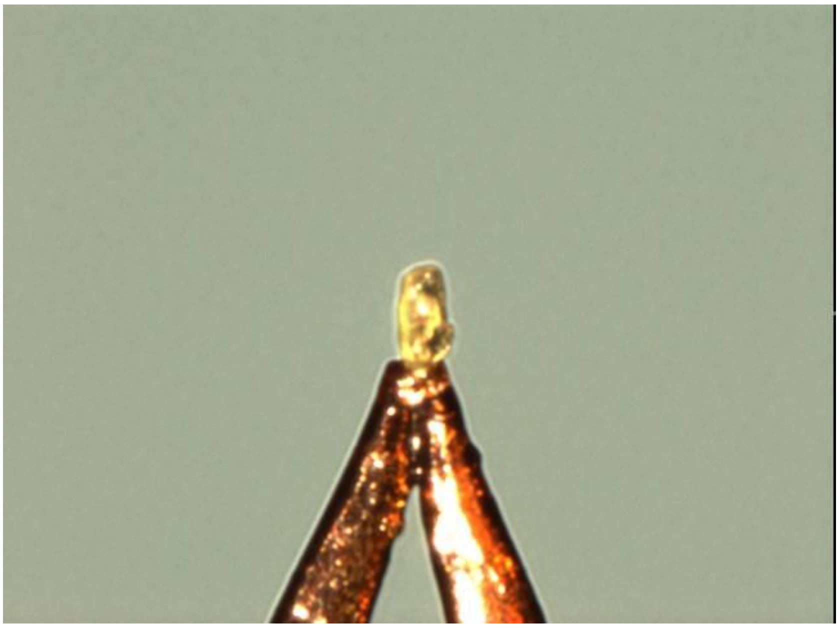

Image of the Crystal on the Diffractometer.

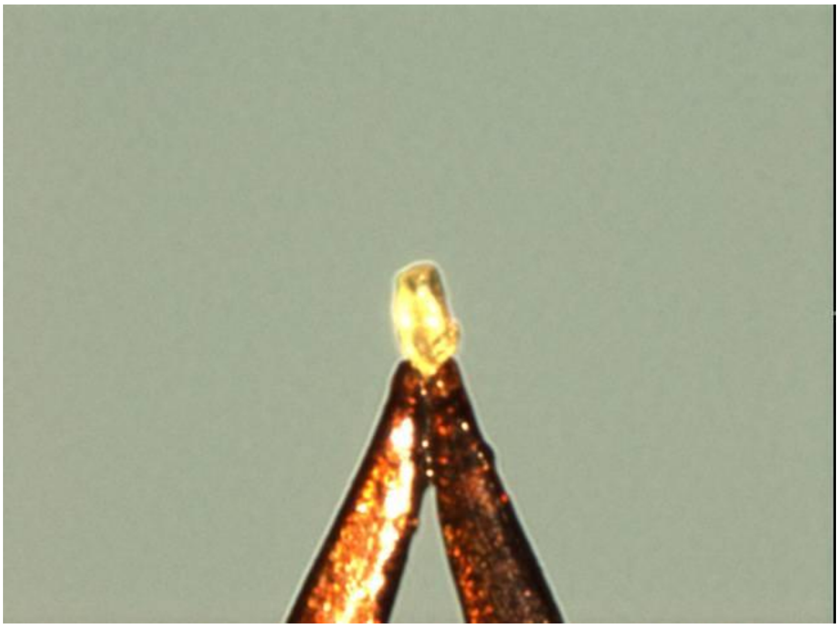

Image of the Crystal on the Diffractometer.

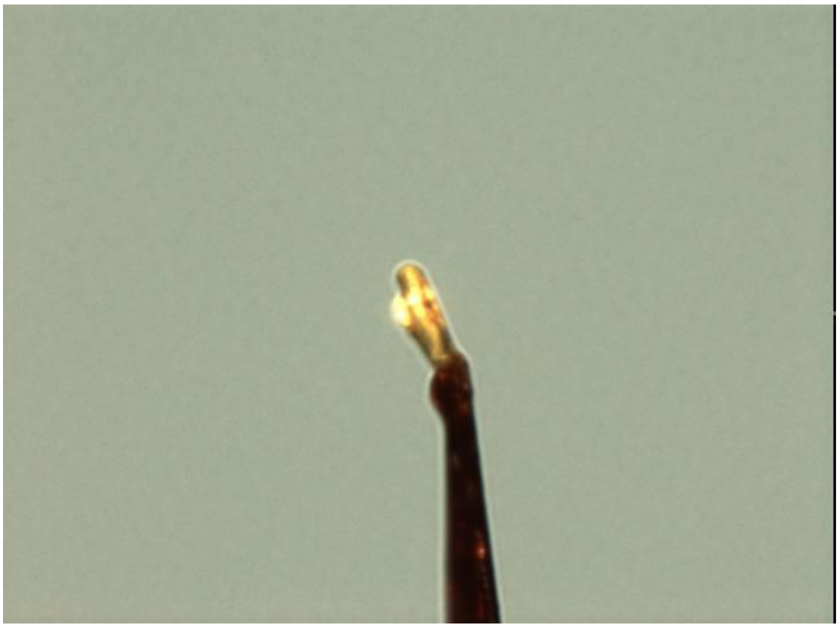

Image of the Crystal on the Diffractometer.

Data Plots: Diffraction Data

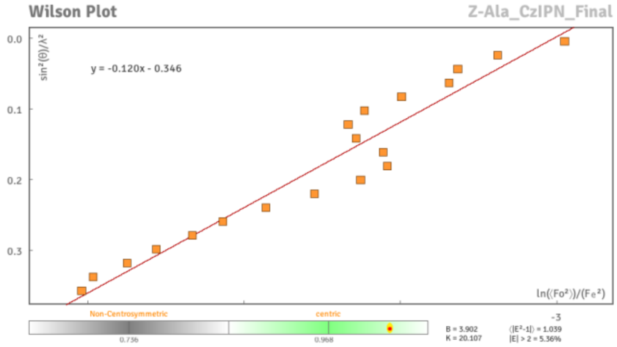

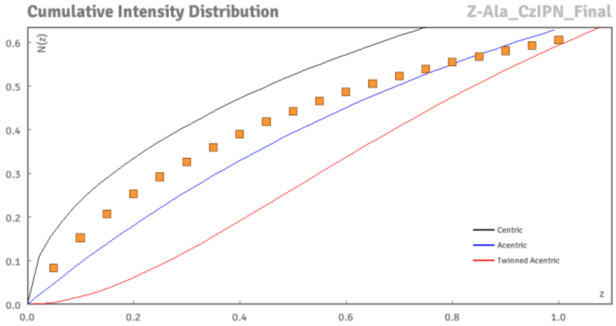

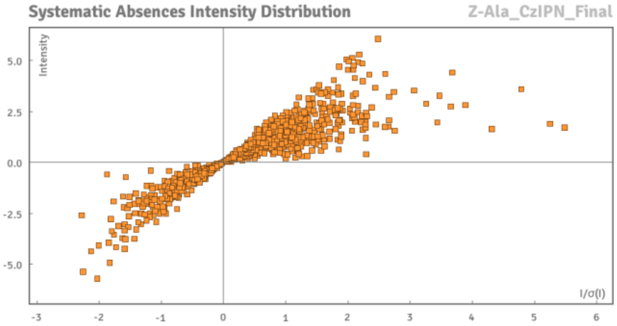

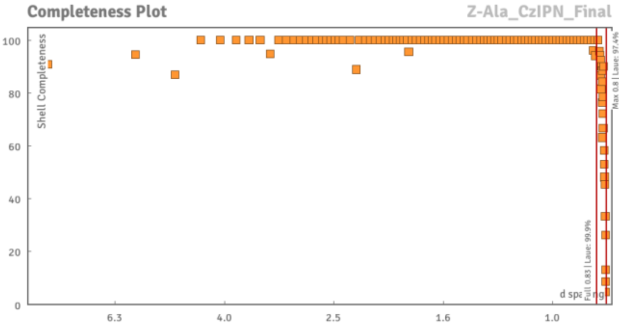

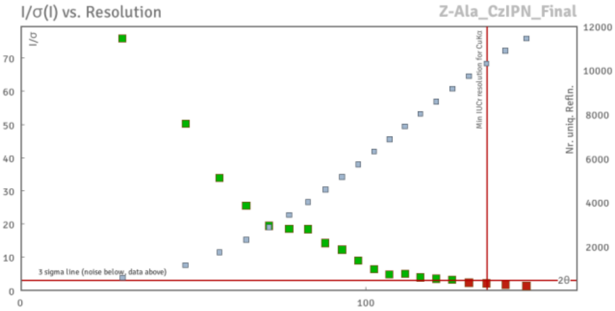

Data Plots: Refinement and Data

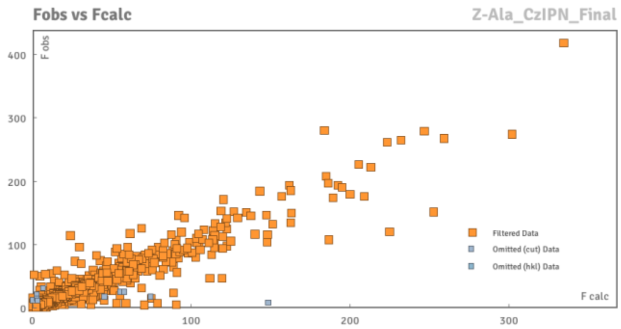

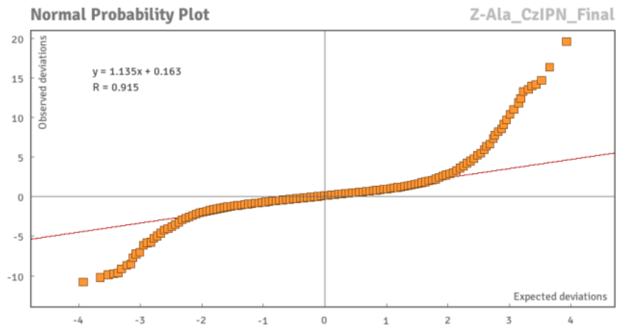

Reflection Statistics

Total reflections (after filtering)	86403	Unique reflections	11695
Completeness	0.974	Mean I/□	15.93
hkl_{max} collected	(17, 24, 22)	hkl_{min} collected	(-17, -24, -26)
hkl_{max} used	(17, 24, 26)	hkl_{min} used	(-17, 0, 0)
Lim d_{max} collected	100.0	Lim d_{min} collected	0.77
d_{max} used	20.15	d_{min} used	0.8
Friedel pairs	10475	Friedel pairs merged	1
Inconsistent equivalents	0	R_{int}	0.0411
R_{sigma}	0.0358	Intensity transformed	0
Omitted reflections	0	Omitted by user (OMIT hkl)	154
Multiplicity	(10558, 10995, 6099, 2414, 939, 533, 413, 324, 292, 307, 217, 152, 90, 34, 32, 17, 2, 6, 3, 1)	Maximum multiplicity	36
Removed systematic absences	1794	Filtered off (Shel/OMIT)	0

Table S6: Fractional Atomic Coordinates (×10^4) and Equivalent Isotropic Displacement Parameters (Å^2×10^3) for **3za**. U_{eq} is defined as 1/3 of the trace of the orthogonalised U_{ij}.

Atom	x	y	z	U_{eq}
O1	4382(11)	2501(7)	6966(6)	66(4)
O1A	3947.1(18)	3195.7(12)	7021.8(10)	74.9(8)
O2A	4828.3(15)	2700.9(9)	7953.7(8)	52.1(6)
O2B	4573(9)	3331(6)	7710(6)	61(3)
N1A	3316.4(18)	2387.9(12)	7537.0(10)	46.4(6)
N1B	3138(9)	2816(8)	7310(8)	52(3)
N2	2977.1(15)	1028.7(10)	6846.5(9)	46.4(5)
N3	2896.8(17)	-1.8(11)	5511.1(11)	58.0(6)
N4	2590.7(15)	1441.0(9)	4511.7(8)	42.4(5)
N5	2137.4(16)	2789.7(9)	4684.8(8)	45.1(5)
N6	1778.8(16)	3197.0(10)	5906.8(9)	49.0(5)
C1	2368.8(18)	2129.4(11)	6403.5(10)	43.9(6)
C2	2653.3(18)	1482.5(12)	6307.5(10)	44.1(6)

Atom	x	y	z	U_{eq}
C3	2672.3(17)	1250.6(11)	5676.1(10)	40.7(5)
C4	2492.5(17)	1680.1(11)	5130.3(10)	40.3(5)
C5	2233.3(18)	2332.6(11)	5217.8(10)	40.9(5)
C6	2131.2(18)	2545.8(11)	5843.9(10)	43.8(6)
C7	2382(2)	2409.6(14)	7092.1(11)	54.4(7)
C8	1647(2)	2105.8(13)	7403.8(12)	54.4(7)
C9	4025(2)	2793.5(13)	7455.5(12)	55.1(7)
C10	5634(2)	3106.9(17)	7886.6(14)	70.6(8)
C11	6428(3)	2958.0(15)	8446.2(15)	69.0(8)
C12	7054(3)	3458.0(17)	8719.7(17)	84.0(10)
C13	7871(3)	3355(2)	9228(2)	103.6(12)
C14	8040(3)	2730(2)	9494(2)	108.4(13)
C15	7429(3)	2216(2)	9256.9(19)	90.8(11)
C16	6649(3)	2309.6(18)	8729.2(17)	81.7(9)
C17	3953.0(19)	881.9(13)	7104.2(10)	48.9(6)
C18	4749(2)	1242.2(15)	7036.2(12)	60.4(7)
C19	5636(2)	996.4(18)	7366.1(13)	68.4(8)
C20	5726(2)	416.8(17)	7751.4(13)	69.5(9)
C21	4933(2)	59.7(15)	7804.7(12)	60.4(7)
C22	4025(2)	285.9(12)	7470.5(10)	48.2(6)
C23	3065(2)	48.9(12)	7410.8(10)	47.0(6)
C24	2667(2)	-495.6(12)	7667.2(12)	59.0(7)
C25	1690(3)	-570.8(13)	7509.6(13)	64.7(8)
C26	1088(2)	-120.6(14)	7097.7(12)	60.5(7)
C27	1455(2)	424.0(13)	6844.1(11)	50.8(6)
C28	2436.2(19)	506.3(11)	7010.7(10)	44.4(6)
C29	2820.7(18)	553.4(12)	5583.5(11)	45.3(6)
C30	3424.0(19)	1160.5(11)	4388.0(11)	46.5(6)
C31	4315(2)	1049.7(13)	4821.0(13)	54.8(7)
C32	5013(2)	758.8(15)	4559.5(15)	66.7(8)
C33	4834(3)	598.5(16)	3886.6(16)	73.9(9)
C34	3961(2)	723.8(14)	3461.6(14)	66.2(8)
C35	3234(2)	1010.6(12)	3707.1(12)	51.7(6)
C36	2259(2)	1183.5(12)	3413.9(11)	48.5(6)
C37	1659(2)	1108.9(13)	2773.8(11)	58.2(7)
C38	727(2)	1305.2(13)	2656.7(12)	60.7(8)
C39	353(2)	1563.9(13)	3171.2(12)	56.2(7)
C40	926(2)	1625.0(12)	3809.6(11)	48.1(6)
C41	1866.1(19)	1443.7(10)	3916.1(10)	41.6(6)
C42	1339(2)	3170.4(12)	4377.7(10)	48.5(6)
C43	404(2)	3110.0(13)	4403.3(11)	56.0(7)
C44	-264(2)	3539.6(15)	4016.9(12)	68.0(9)
C45	24(3)	4016.0(15)	3614.2(13)	73.3(10)
C46	954(3)	4072.2(14)	3584.2(12)	66.9(9)
C47	1630(2)	3646.5(12)	3966.6(11)	54.6(7)
C48	2637(2)	3551.0(12)	4030.8(10)	54.2(7)
C49	3320(3)	3866.0(13)	3765.4(12)	66.2(9)
C50	4237(3)	3645.5(15)	3915.9(14)	71.2(9)
C51	4513(3)	3106.7(15)	4345.9(14)	69.3(8)
C52	3867(2)	2794.1(13)	4631.1(12)	56.4(7)
C53	2935(2)	3011.2(11)	4466.5(10)	48.4(6)
C54	837(2)	3332.5(14)	5921.6(10)	53.8(7)

Atom	x	y	z	U_{eq}
C55	118(2)	2897.6(16)	5977.5(11)	59.3(7)
C56	-774(2)	3171(2)	5950.3(13)	78.0(10)
C57	-953(3)	3843(2)	5867.6(15)	87.7(12)
C58	-235(3)	4274(2)	5807.9(14)	80.7(11)
C59	674(2)	4021.9(14)	5836.1(11)	60.1(8)
C60	1542(3)	4310.5(13)	5752.8(11)	62.8(8)
C61	1806(3)	4962.5(15)	5633.3(14)	77.2(10)
C62	2702(3)	5072.9(16)	5558.2(15)	86.1(12)
C63	3368(3)	4558.7(15)	5611.4(13)	73.2(9)
C64	3134(3)	3913.6(14)	5736.4(12)	62.5(8)
C65	2210(2)	3799.3(13)	5794.0(10)	53.5(7)

Table S7: Anisotropic Displacement Parameters (×10[4]) for **3za**. The anisotropic displacement factor exponent takes the form: $-2\square^2[h^2a^{*2} \times U_{11} + ... + 2hka^* \times b^* \times U_{12}]$

Atom	U_{11}	U_{22}	U_{33}	U_{23}	U_{13}	U_{12}
O1	90(9)	63(8)	45(7)	-3(6)	16(7)	-6(7)
O1A	95.6(18)	78.4(16)	43.8(12)	26.6(11)	4.5(11)	-17.2(13)
O2A	70.2(14)	49.4(11)	34.2(10)	6.4(8)	8.5(9)	-6.7(10)
O2B	75(6)	55(5)	46(5)	1(5)	5(5)	2(5)
N1A	73.7(17)	40.0(13)	25.9(10)	3.7(9)	13.2(11)	-0.3(11)
N1B	73(6)	46(6)	38(5)	-6(5)	14(5)	-8(5)
N2	59.9(13)	49.3(11)	30.4(9)	12.4(8)	12.0(9)	2.5(10)
N3	77.0(16)	43.2(12)	57.5(13)	6.8(10)	23.9(12)	11.0(11)
N4	64.8(13)	38.7(10)	26.3(9)	-0.3(7)	16.4(9)	9.4(9)
N5	75.0(14)	36.5(10)	25.6(9)	5.7(7)	16.2(9)	10.1(10)
N6	76.5(15)	43.0(11)	30.2(9)	-1.6(8)	18.6(10)	12.4(10)
C1	65.3(16)	44.2(13)	24.9(10)	2.3(9)	16.1(10)	3.3(11)
C2	60.3(16)	47.5(13)	25.8(10)	9.1(9)	12.9(10)	1.2(11)
C3	57.7(15)	36.4(12)	29.3(10)	3.8(9)	13.4(10)	3.7(10)
C4	60.5(15)	37.4(12)	25.2(10)	1.0(8)	15.0(10)	3.2(10)
C5	63.6(16)	37.4(12)	23.3(10)	3.7(8)	13.8(10)	7.4(10)
C6	63.3(16)	41.8(12)	28.5(10)	-0.2(9)	15.9(10)	6.7(11)
C7	80(2)	56.4(15)	28.9(11)	-3.0(10)	18.0(12)	-2.6(14)
C8	79.2(19)	52.4(15)	36.3(12)	1.4(11)	23.1(12)	8.6(13)
C9	75.2(18)	49.6(14)	37.5(12)	0.7(11)	8.4(12)	-0.3(13)
C10	81(2)	80(2)	51.8(16)	-1.4(14)	20.1(15)	-15.4(17)
C11	97(2)	57.1(17)	59.5(16)	-0.1(13)	32.1(16)	-9.1(16)
C12	109(3)	63.3(19)	71(2)	-1.3(16)	4.9(19)	0.4(19)
C13	121(3)	84(2)	93(3)	7(2)	2(2)	-8(2)
C14	116(3)	99(3)	98(3)	22(2)	4(2)	-12(2)
C15	98(3)	83(2)	86(2)	23.5(19)	12(2)	1(2)
C16	92(2)	79(2)	77(2)	3.7(17)	28.0(19)	-9.2(18)
C17	61.7(16)	59.1(15)	26.2(10)	-1.4(10)	11.1(10)	4.8(12)
C18	67.2(19)	74.4(18)	40.7(13)	-0.7(12)	15.6(13)	3.4(15)
C19	64.0(19)	96(2)	45.3(14)	-13.2(15)	13.4(13)	2.3(16)
C20	73(2)	91(2)	39.9(14)	-17.8(14)	3.7(13)	25.9(17)
C21	82(2)	64.8(17)	31.0(12)	-9.4(11)	6.8(13)	17.7(15)
C22	71.4(17)	50.4(13)	20.7(10)	-3.0(9)	7.5(10)	16.3(12)
C23	77.6(18)	38.9(12)	23.5(10)	0.0(9)	10.8(10)	8.4(12)

Atom	U_{11}	U_{22}	U_{33}	U_{23}	U_{13}	U_{12}
C24	103(2)	35.9(13)	35.6(12)	3.2(10)	13.1(14)	3.8(14)
C25	102(2)	46.1(15)	46.6(14)	2.0(12)	20.2(15)	-12.6(15)
C26	84(2)	56.2(16)	43.9(13)	-2.0(12)	21.2(13)	-14.1(14)
C27	71.1(18)	49.6(14)	31.9(11)	-1.1(10)	13.4(11)	-3.6(12)
C28	70.1(17)	39.4(12)	24.7(10)	2.5(9)	13.6(10)	4.4(11)
C29	62.1(16)	43.2(14)	32.7(11)	7.0(10)	15.9(11)	7.8(11)
C30	69.5(17)	36.7(12)	39.5(12)	2.0(10)	24.8(12)	10.1(11)
C31	73.2(19)	50.2(14)	46.7(14)	6.2(11)	25.9(13)	13.4(13)
C32	76(2)	66.4(18)	64.9(18)	10.7(14)	31.0(15)	22.9(15)
C33	91(2)	75(2)	68.1(19)	-1.6(16)	44.3(18)	25.1(18)
C34	96(2)	63.7(18)	49.6(15)	-2.5(13)	38.5(16)	14.5(16)
C35	82(2)	42.2(13)	39.4(12)	-0.1(10)	29.9(13)	7.0(13)
C36	79.9(19)	39.4(12)	30.8(11)	-0.6(9)	22.5(12)	-1.5(12)
C37	96(2)	51.5(15)	30.6(12)	-3.8(10)	23.2(13)	-7.6(15)
C38	93(2)	55.2(16)	30.8(12)	1.9(11)	10.4(13)	-14.7(15)
C39	73.3(19)	49.0(15)	43.3(13)	1.8(11)	8.9(13)	-6.3(13)
C40	69.4(18)	39.5(13)	36.2(12)	-1.2(10)	14.6(11)	0.1(12)
C41	68.5(17)	30.9(11)	27.1(10)	1.7(8)	15.0(10)	-2.3(11)
C42	80(2)	42.3(13)	22.7(10)	-0.5(9)	12.8(11)	14.9(12)
C43	88(2)	52.2(15)	27.1(11)	-3.1(10)	12.3(12)	19.6(14)
C44	97(2)	65.4(18)	39.0(14)	-2.6(13)	11.3(14)	30.9(17)
C45	114(3)	58.6(18)	41.9(15)	4.5(13)	8.3(16)	34.9(18)
C46	119(3)	47.7(15)	33.8(13)	5.2(11)	18.3(15)	19.9(17)
C47	102(2)	37.1(13)	24.9(11)	1.0(9)	16.3(12)	11.5(13)
C48	102(2)	38.5(13)	24.9(11)	1.3(9)	21.8(12)	3.3(13)
C49	124(3)	44.0(15)	36.9(13)	6.3(11)	33.1(16)	-4.2(17)
C50	109(3)	59.4(18)	57.0(17)	8.8(14)	43.2(18)	-8.7(18)
C51	95(2)	63.7(18)	58.8(17)	6.0(14)	37.7(16)	-1.9(16)
C52	87(2)	45.4(14)	44.6(13)	6.2(11)	31.5(14)	2.7(14)
C53	85(2)	38.1(12)	27.1(11)	-0.2(9)	23.3(12)	1.6(12)
C54	81(2)	58.8(16)	22.0(10)	-5.1(10)	12.5(11)	20.0(14)
C55	72(2)	75.4(19)	31.2(12)	-4.2(12)	15.3(12)	14.4(16)
C56	77(2)	121(3)	34.0(14)	-10.8(16)	10.0(14)	18(2)
C57	87(3)	124(3)	42.8(16)	-24.7(19)	-1.6(16)	48(3)
C58	105(3)	87(2)	38.1(15)	-17.4(15)	-6.1(17)	46(2)
C59	94(2)	56.5(16)	25.0(11)	-9.3(11)	5.0(13)	22.4(16)
C60	113(3)	44.5(15)	25.3(11)	-7.4(10)	6.7(13)	19.4(15)
C61	131(3)	47.7(17)	44.0(15)	-5.1(12)	5.1(18)	11.4(19)
C62	153(4)	44.5(17)	52.8(18)	-5.1(13)	10(2)	-1(2)
C63	115(3)	58.8(18)	41.8(14)	-5.2(13)	11.6(16)	-16.8(18)
C64	101(2)	54.7(16)	30.9(12)	-5.1(11)	14.9(13)	0.0(16)
C65	88(2)	47.2(14)	23.6(11)	-6.1(10)	10.8(12)	4.8(14)

Table S8: Bond Lengths in Å for **3za**.

Atom	Atom	Length/Å
O1	C9	1.382(12)
O1A	C9	1.197(3)
O2A	C9	1.360(3)
O2A	C10	1.454(3)
O2B	C9	1.365(11)
O2B	C10	1.548(12)
N1A	C7	1.432(4)
N1A	C9	1.350(4)

Atom	Atom	Length/Å
N1B	C7	1.347(12)
N1B	C9	1.238(13)
N2	C2	1.431(3)
N2	C17	1.405(3)
N2	C28	1.401(3)
N3	C29	1.137(3)
N4	C4	1.414(3)
N4	C30	1.406(3)
N4	C41	1.407(3)
N5	C5	1.421(3)
N5	C42	1.397(3)
N5	C53	1.409(3)
N6	C6	1.424(3)
N6	C54	1.391(3)
N6	C65	1.409(3)
C1	C2	1.395(3)
C1	C6	1.406(3)
C1	C7	1.535(3)
C2	C3	1.401(3)
C3	C4	1.399(3)
C3	C29	1.440(3)
C4	C5	1.390(3)
C5	C6	1.413(3)
C7	C8	1.502(4)
C10	C11	1.445(4)
C11	C12	1.376(4)
C11	C16	1.435(4)
C12	C13	1.388(5)
C13	C14	1.371(5)
C14	C15	1.368(5)
C15	C16	1.374(5)
C17	C18	1.393(4)
C17	C22	1.411(3)
C18	C19	1.382(4)
C19	C20	1.403(5)
C20	C21	1.377(4)
C21	C22	1.395(4)
C22	C23	1.439(4)
C23	C24	1.403(4)
C23	C28	1.409(3)
C24	C25	1.371(4)
C25	C26	1.392(4)

Atom	Atom	Length/Å
C26	C27	1.378(4)
C27	C28	1.378(4)
C30	C31	1.390(4)
C30	C35	1.407(3)
C31	C32	1.386(4)
C32	C33	1.397(4)
C33	C34	1.369(4)
C34	C35	1.398(4)
C35	C36	1.428(4)
C36	C37	1.404(4)
C36	C41	1.407(3)
C37	C38	1.361(4)
C38	C39	1.412(4)
C39	C40	1.384(3)
C40	C41	1.366(4)
C42	C43	1.366(4)
C42	C47	1.415(3)
C43	C44	1.391(4)
C44	C45	1.402(5)
C45	C46	1.360(5)
C46	C47	1.389(4)
C47	C48	1.436(4)
C48	C49	1.395(4)
C48	C53	1.412(3)
C49	C50	1.354(4)
C50	C51	1.399(4)
C51	C52	1.374(4)
C52	C53	1.370(4)
C54	C55	1.382(4)
C54	C59	1.412(4)
C55	C56	1.386(4)
C56	C57	1.381(5)
C57	C58	1.379(5)
C58	C59	1.391(4)
C59	C60	1.427(4)
C60	C61	1.406(4)
C60	C65	1.397(4)
C61	C62	1.355(5)
C62	C63	1.397(5)
C63	C64	1.382(4)
C64	C65	1.384(4)

Table S9: Bond Angles in ° for **3za**.

Atom	Atom	Atom	Angle/°
C9	O2A	C10	113.4(2)
C9	O2B	C10	107.6(8)
C9	N1A	C7	121.2(2)
C9	N1B	C7	139.4(14)
C17	N2	C2	122.2(2)

Atom	Atom	Atom	Angle/°
C28	N2	C2	125.3(2)
C28	N2	C17	108.23(19)
C30	N4	C4	125.9(2)
C30	N4	C41	108.65(18)
C41	N4	C4	125.4(2)

Atom	Atom	Atom	Angle/°
C42	N5	C5	128.4(2)
C42	N5	C53	108.84(19)
C53	N5	C5	121.9(2)
C54	N6	C6	123.7(2)
C54	N6	C65	107.9(2)
C65	N6	C6	126.5(2)
C2	C1	C6	117.27(19)
C2	C1	C7	122.7(2)
C6	C1	C7	119.8(2)
C1	C2	N2	122.28(19)
C1	C2	C3	121.37(19)
C3	C2	N2	116.3(2)
C2	C3	C29	119.25(19)
C4	C3	C2	120.8(2)
C4	C3	C29	119.85(19)
C3	C4	N4	119.20(19)
C5	C4	N4	122.14(18)
C5	C4	C3	118.66(18)
C4	C5	N5	119.15(17)
C4	C5	C6	120.03(19)
C6	C5	N5	120.68(19)
C1	C6	N6	119.47(18)
C1	C6	C5	121.5(2)
C5	C6	N6	119.05(19)
N1A	C7	C1	112.5(2)
N1A	C7	C8	111.0(2)
N1B	C7	C1	111.0(7)
N1B	C7	C8	134.4(7)
C8	C7	C1	114.0(2)
O1A	C9	O2A	124.8(3)
O1A	C9	N1A	124.9(3)
O2B	C9	O1	109.8(8)
N1A	C9	O2A	110.3(2)
N1B	C9	O1	112.7(10)
N1B	C9	O2B	121.8(9)
C11	C10	O2A	107.2(2)
C11	C10	O2B	142.0(5)
C12	C11	C10	119.1(3)
C12	C11	C16	116.1(3)
C16	C11	C10	124.7(3)
C11	C12	C13	123.3(3)
C14	C13	C12	118.5(4)
C15	C14	C13	121.0(4)
C14	C15	C16	120.5(4)
C15	C16	C11	120.4(3)
N2	C17	C22	108.5(2)
C18	C17	N2	128.4(2)
C18	C17	C22	123.1(2)
C19	C18	C17	116.4(3)
C18	C19	C20	121.6(3)
C21	C20	C19	121.3(3)
C20	C21	C22	118.8(3)
C17	C22	C23	107.2(2)
C21	C22	C17	118.7(3)
C21	C22	C23	134.1(2)
C24	C23	C22	134.5(2)
C24	C23	C28	118.2(3)
C28	C23	C22	107.2(2)
C25	C24	C23	119.2(3)
C24	C25	C26	121.3(3)
C27	C26	C25	121.0(3)
C26	C27	C28	117.8(3)
N2	C28	C23	108.7(2)
C27	C28	N2	128.8(2)
C27	C28	C23	122.5(2)
N3	C29	C3	177.0(3)
N4	C30	C35	108.0(2)
C31	C30	N4	129.6(2)
C31	C30	C35	122.3(2)
C32	C31	C30	117.1(2)
C31	C32	C33	121.3(3)
C34	C33	C32	121.1(3)
C33	C34	C35	119.4(3)
C30	C35	C36	107.6(2)
C34	C35	C30	118.7(3)
C34	C35	C36	133.7(2)
C37	C36	C35	133.4(2)
C37	C36	C41	118.5(3)
C41	C36	C35	107.9(2)
C38	C37	C36	119.1(2)
C37	C38	C39	121.1(2)
C40	C39	C38	120.7(3)
C41	C40	C39	117.6(2)
C36	C41	N4	107.8(2)
C40	C41	N4	129.2(2)
C40	C41	C36	123.0(2)
N5	C42	C47	108.4(2)
C43	C42	N5	129.8(2)
C43	C42	C47	121.7(2)
C42	C43	C44	117.9(3)
C43	C44	C45	120.4(3)
C46	C45	C44	121.7(3)
C45	C46	C47	118.7(3)
C42	C47	C48	107.2(2)
C46	C47	C42	119.6(3)
C46	C47	C48	133.1(3)
C49	C48	C47	134.3(2)
C49	C48	C53	118.2(3)
C53	C48	C47	107.6(2)
C50	C49	C48	120.2(3)
C49	C50	C51	120.5(3)
C52	C51	C50	121.1(3)
C53	C52	C51	118.1(3)
N5	C53	C48	107.9(2)

Atom	Atom	Atom	Angle/°
C52	C53	N5	130.1(2)
C52	C53	C48	121.9(2)
N6	C54	C59	108.8(3)
C55	C54	N6	129.2(2)
C55	C54	C59	121.9(3)
C54	C55	C56	116.8(3)
C57	C56	C55	122.4(4)
C58	C57	C56	120.5(3)
C57	C58	C59	119.0(3)
C54	C59	C60	107.0(3)
C58	C59	C54	119.3(3)
C58	C59	C60	133.5(3)
C61	C60	C59	133.3(3)
C65	C60	C59	107.7(3)
C65	C60	C61	119.1(3)
C62	C61	C60	118.7(3)
C61	C62	C63	121.6(3)
C64	C63	C62	121.1(4)
C63	C64	C65	117.2(3)
C60	C65	N6	108.6(3)
C64	C65	N6	129.1(2)
C64	C65	C60	122.3(3)

Table S10: Torsion Angles in ° for **3za**.

Atom	Atom	Atom	Atom	Angle/°
O2A	C10	C11	C12	-145.0(3)
O2A	C10	C11	C16	38.1(4)
O2B	C10	C11	C12	-90.9(9)
O2B	C10	C11	C16	92.2(9)
N2	C2	C3	C4	-171.5(2)
N2	C2	C3	C29	12.3(3)
N2	C17	C18	C19	177.5(2)
N2	C17	C22	C21	-176.57(19)
N2	C17	C22	C23	2.4(2)
N4	C4	C5	N5	-5.1(4)
N4	C4	C5	C6	179.1(2)
N4	C30	C31	C32	179.7(2)
N4	C30	C35	C34	-179.9(2)
N4	C30	C35	C36	-1.8(3)
N5	C5	C6	N6	10.1(4)
N5	C5	C6	C1	-169.9(2)
N5	C42	C43	C44	-176.6(2)
N5	C42	C47	C46	177.5(2)
N5	C42	C47	C48	-0.3(2)
N6	C54	C55	C56	-177.3(2)
N6	C54	C59	C58	177.5(2)
N6	C54	C59	C60	1.2(2)
C1	C2	C3	C4	6.1(4)
C1	C2	C3	C29	-170.1(2)
C2	N2	C17	C18	18.8(4)
C2	N2	C17	C22	-161.8(2)
C2	N2	C28	C23	161.0(2)
C2	N2	C28	C27	-20.6(4)
C2	C1	C6	N6	176.1(2)
C2	C1	C6	C5	-3.9(4)
C2	C1	C7	N1A	58.5(3)
C2	C1	C7	N1B	103.2(9)
C2	C1	C7	C8	-69.0(3)
C2	C3	C4	N4	175.1(2)
C2	C3	C4	C5	-4.1(4)

Atom	Atom	Atom	Atom	Angle/°
C3	C4	C5	N5	174.1(2)
C3	C4	C5	C6	-1.7(4)
C4	N4	C30	C31	0.4(4)
C4	N4	C30	C35	-177.8(2)
C4	N4	C41	C36	178.2(2)
C4	N4	C41	C40	-4.9(4)
C4	C5	C6	N6	-174.2(2)
C4	C5	C6	C1	5.8(4)
C5	N5	C42	C43	-15.4(4)
C5	N5	C42	C47	168.4(2)
C5	N5	C53	C48	-168.47(19)
C5	N5	C53	C52	9.5(4)
C6	N6	C54	C55	10.4(3)
C6	N6	C54	C59	-166.84(19)
C6	N6	C65	C60	166.0(2)
C6	N6	C65	C64	-14.9(4)
C6	C1	C2	N2	175.5(2)
C6	C1	C2	C3	-2.0(4)
C6	C1	C7	N1A	-116.6(3)
C6	C1	C7	N1B	-71.9(9)
C6	C1	C7	C8	115.9(3)
C7	N1A	C9	O1A	0.1(4)
C7	N1A	C9	O2A	177.3(2)
C7	N1B	C9	O1	51(2)
C7	N1B	C9	O2B	-175.7(16)
C7	C1	C2	N2	0.2(4)
C7	C1	C2	C3	-177.2(2)
C7	C1	C6	N6	-8.6(4)
C7	C1	C6	C5	171.5(2)
C9	O2A	C10	C11	179.9(2)
C9	O2B	C10	C11	-107.2(8)
C9	N1A	C7	C1	70.8(3)
C9	N1A	C7	C8	-160.1(2)
C9	N1B	C7	C1	-62(2)
C9	N1B	C7	C8	108.0(18)
C10	O2A	C9	O1A	-4.6(4)
C10	O2A	C9	N1A	178.1(2)
C10	O2B	C9	O1	-49.9(11)
C10	O2B	C9	N1B	175.2(10)
C10	C11	C12	C13	-175.5(4)
C10	C11	C16	C15	179.1(3)
C11	C12	C13	C14	-3.4(7)
C12	C11	C16	C15	2.1(5)
C12	C13	C14	C15	1.3(7)
C13	C14	C15	C16	2.4(7)
C14	C15	C16	C11	-4.1(6)
C16	C11	C12	C13	1.6(6)
C17	N2	C2	C1	-102.8(3)
C17	N2	C2	C3	74.7(3)
C17	N2	C28	C23	3.9(2)
C17	N2	C28	C27	-177.6(2)
C17	C18	C19	C20	-0.3(4)

Atom	Atom	Atom	Atom	Angle/°
C17	C22	C23	C24	-178.1(2)
C17	C22	C23	C28	0.0(2)
C18	C17	C22	C21	2.9(3)
C18	C17	C22	C23	-178.2(2)
C18	C19	C20	C21	1.5(4)
C19	C20	C21	C22	-0.4(4)
C20	C21	C22	C17	-1.6(3)
C20	C21	C22	C23	179.7(2)
C21	C22	C23	C24	0.6(4)
C21	C22	C23	C28	178.7(2)
C22	C17	C18	C19	-1.8(4)
C22	C23	C24	C25	179.4(2)
C22	C23	C28	N2	-2.4(2)
C22	C23	C28	C27	179.0(2)
C23	C24	C25	C26	0.4(4)
C24	C23	C28	N2	176.09(19)
C24	C23	C28	C27	-2.5(3)
C24	C25	C26	C27	-1.2(4)
C25	C26	C27	C28	0.2(4)
C26	C27	C28	N2	-176.6(2)
C26	C27	C28	C23	1.7(3)
C28	N2	C2	C1	103.2(3)
C28	N2	C2	C3	-79.3(3)
C28	N2	C17	C18	176.7(2)
C28	N2	C17	C22	-3.9(2)
C28	C23	C24	C25	1.4(3)
C29	C3	C4	N4	-8.7(4)
C29	C3	C4	C5	172.0(2)
C30	N4	C4	C3	-57.5(3)
C30	N4	C4	C5	121.7(3)
C30	N4	C41	C36	-1.9(2)
C30	N4	C41	C40	175.0(2)
C30	C31	C32	C33	1.5(4)
C30	C35	C36	C37	-175.2(3)
C30	C35	C36	C41	0.6(3)
C31	C30	C35	C34	1.7(4)
C31	C30	C35	C36	179.9(2)
C31	C32	C33	C34	0.0(5)
C32	C33	C34	C35	-0.6(5)
C33	C34	C35	C30	-0.2(4)
C33	C34	C35	C36	-177.8(3)
C34	C35	C36	C37	2.5(5)
C34	C35	C36	C41	178.4(3)
C35	C30	C31	C32	-2.3(4)
C35	C36	C37	C38	177.1(3)
C35	C36	C41	N4	0.8(3)
C35	C36	C41	C40	-176.3(2)
C36	C37	C38	C39	-1.8(4)
C37	C36	C41	N4	177.3(2)
C37	C36	C41	C40	0.2(3)
C37	C38	C39	C40	0.0(4)
C38	C39	C40	C41	1.8(4)

Atom	Atom	Atom	Atom	Angle/°
C39	C40	C41	N4	-178.4(2)
C39	C40	C41	C36	-1.9(3)
C41	N4	C4	C3	122.4(2)
C41	N4	C4	C5	-58.4(3)
C41	N4	C30	C31	-179.5(2)
C41	N4	C30	C35	2.3(3)
C41	C36	C37	C38	1.6(4)
C42	N5	C5	C4	123.1(3)
C42	N5	C5	C6	-61.2(3)
C42	N5	C53	C48	1.7(2)
C42	N5	C53	C52	179.6(2)
C42	C43	C44	C45	0.2(4)
C42	C47	C48	C49	-178.7(3)
C42	C47	C48	C53	1.3(3)
C43	C42	C47	C46	0.9(3)
C43	C42	C47	C48	-176.8(2)
C43	C44	C45	C46	0.4(4)
C44	C45	C46	C47	-0.3(4)
C45	C46	C47	C42	-0.3(4)
C45	C46	C47	C48	176.8(3)
C46	C47	C48	C49	4.0(5)
C46	C47	C48	C53	-176.1(3)
C47	C42	C43	C44	-0.9(3)
C47	C48	C49	C50	-178.7(3)
C47	C48	C53	N5	-1.8(2)
C47	C48	C53	C52	-180.0(2)
C48	C49	C50	C51	-0.9(4)
C49	C48	C53	N5	178.2(2)
C49	C48	C53	C52	0.0(3)
C49	C50	C51	C52	-0.8(5)
C50	C51	C52	C53	2.0(4)
C51	C52	C53	N5	-179.4(2)
C51	C52	C53	C48	-1.6(4)
C53	N5	C5	C4	-68.9(3)
C53	N5	C5	C6	106.8(3)
C53	N5	C42	C43	175.4(2)
C53	N5	C42	C47	-0.9(2)
C53	C48	C49	C50	1.3(4)
C54	N6	C6	C1	-79.2(3)
C54	N6	C6	C5	100.8(3)
C54	N6	C65	C60	1.2(2)
C54	N6	C65	C64	-179.6(2)
C54	C55	C56	C57	0.4(4)
C54	C59	C60	C61	178.1(3)
C54	C59	C60	C65	-0.5(2)
C55	C54	C59	C58	0.1(3)
C55	C54	C59	C60	-176.2(2)
C55	C56	C57	C58	0.0(4)
C56	C57	C58	C59	-0.4(4)
C57	C58	C59	C54	0.3(4)
C57	C58	C59	C60	175.4(3)
C58	C59	C60	C61	2.5(5)

Atom	Atom	Atom	Atom	Angle/°
C58	C59	C60	C65	-176.0(3)
C59	C54	C55	C56	-0.4(3)
C59	C60	C61	C62	-178.1(3)
C59	C60	C65	N6	-0.5(2)
C59	C60	C65	C64	-179.7(2)
C60	C61	C62	C63	-1.4(4)
C61	C60	C65	N6	-179.3(2)
C61	C60	C65	C64	1.5(3)
C61	C62	C63	C64	0.7(4)
C62	C63	C64	C65	1.2(4)
C63	C64	C65	N6	178.7(2)
C63	C64	C65	C60	-2.3(3)
C65	N6	C6	C1	118.3(3)
C65	N6	C6	C5	-61.7(3)
C65	N6	C54	C55	175.7(2)
C65	N6	C54	C59	-1.5(2)
C65	C60	C61	C62	0.4(4)

Table S11: Hydrogen Fractional Atomic Coordinates (×10[4]) and Equivalent Isotropic Displacement Parameters (Å^2×10^3) for **3za**. U_{eq} is defined as 1/3 of the trace of the orthogonalised U_{ij}.

Atom	x	y	z	U_{eq}
H1A	3431.07	2104.9	7869.89	56
H1B	2941.84	3220.56	7366.42	62
H7A	2210.87	2890.34	7024.69	65
H7B	1948.99	2786.58	6900.41	65
H8A	1015.98	2120.8	7088.15	82
H8B	1624.88	2355.25	7804.32	82
H8C	1820	1643.07	7522.39	82
H10A	5806.43	3002.3	7465.73	85
H10B	5467.22	3583.98	7885.15	85
H10C	5598.53	2696.4	7619.63	85
H10D	5924.91	3440.25	7645.99	85
H12	6919.38	3895.95	8552.31	101
H13	8303.48	3708.38	9387.52	124
H14	8588.75	2653.3	9848.25	130
H15	7545.21	1790.77	9458.87	109
H16	6254.44	1942.64	8550.42	98
H18	4685.98	1635.75	6776.91	72
H19	6198.26	1225.34	7331.15	82
H20	6346.38	267.77	7979.88	83
H21	5002.43	-333.63	8064.51	72
H24	3068.79	-808.38	7946.63	71
H25	1417.83	-937.53	7685.01	78
H26	414.42	-189.8	6989.7	73
H27	1045.33	732.52	6563.77	61
H31	4440.44	1168.29	5276.52	66
H32	5625.92	666.5	4843.74	80
H33	5326.3	399.48	3721.17	89

Atom	x	y	z	U_{eq}
H34	3849.18	616.71	3003.97	79
H37	1899.11	924	2427.24	70
H38	321.35	1267.51	2221.63	73
H39	-300.08	1697.62	3078.05	67
H40	674.91	1787.05	4160.92	58
H43	215.65	2784.56	4676.54	67
H44	-919.27	3510.19	4025.87	82
H45	-441.92	4306.77	3355.84	88
H46	1136.95	4396.11	3307.3	80
H49	3141.54	4236.35	3478.18	79
H50	4697.3	3858.1	3728.2	85
H51	5157.12	2954.5	4442.24	83
H52	4060.29	2437.49	4933.86	68
H55	230.99	2433.65	6032.02	71
H56	-1282.02	2884.8	5990.03	94
H57	-1575.53	4010.45	5851.6	105
H58	-358.27	4736.77	5748.47	97
H61	1365.25	5318.81	5605.76	93
H62	2880.59	5510.45	5467.27	103
H63	3991.99	4653.81	5560.68	88
H64	3588.35	3563.69	5781	75

Table S12: Atomic Occupancies for all atoms that are not fully occupied in **3za**.

Atom	Occupancy	Atom	Occupancy	Atom	Occupancy	Atom	Occupancy
O1	0.128(3)	N1A	0.872(3)	H7A	0.872(3)	H10C	0.128(3)
O1A	0.872(3)	H1A	0.872(3)	H7B	0.128(3)	H10D	0.128(3)
O2A	0.872(3)	N1B	0.128(3)	H10A	0.872(3)		
O2B	0.128(3)	H1B	0.128(3)	H10B	0.872(3)		

Table S13: Solvent masking (PLATON/SQUEEZE) information for **3za**.

No	x	y	z	V	e	Content
1	-0.409	0.409	0.500	793.3	193.7	4C4H8O2
2	-0.568	-0.068	0.000	793.3	193.7	4C4H8O2

10. NMR spectrum

4CzBN Z-Ala **3za** ^{1}**H NMR (500 MHz, CDCl$_3$)**

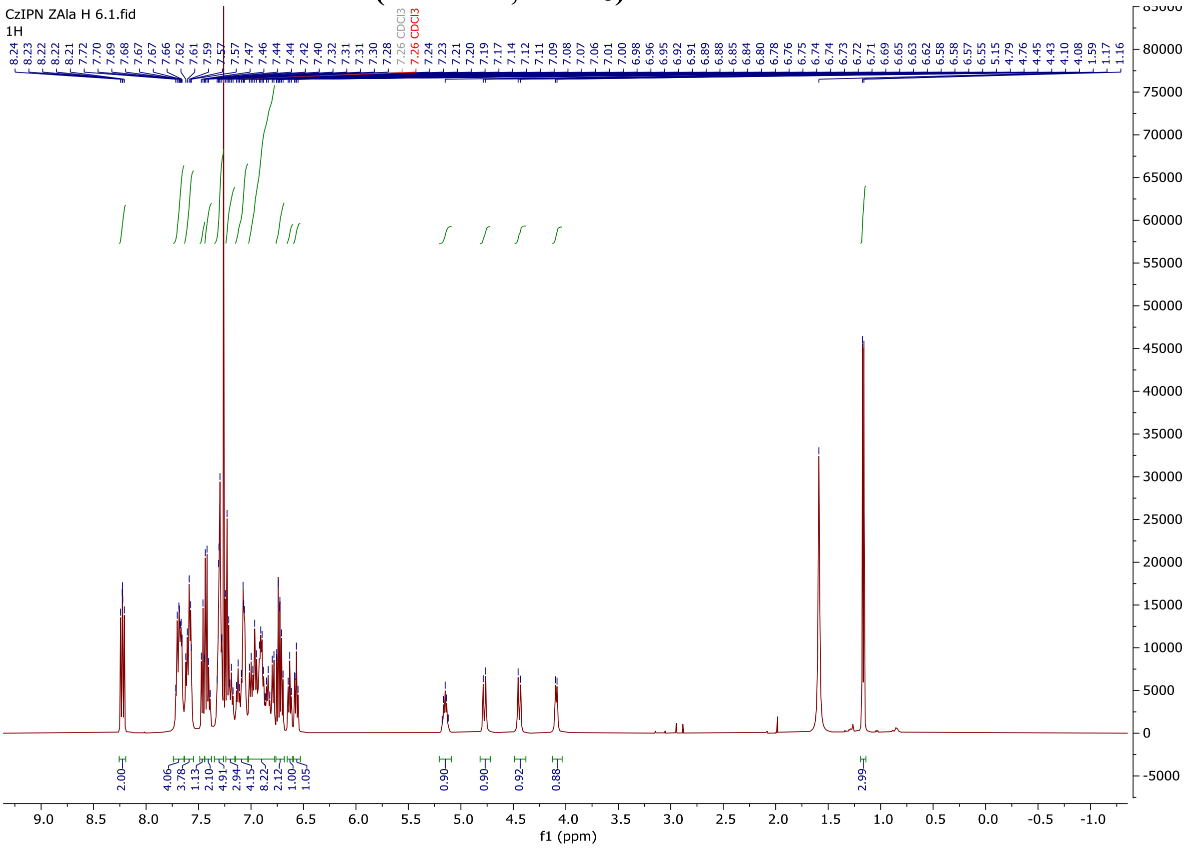

^{13}**C NMR (126 MHz, CDCl$_3$)**

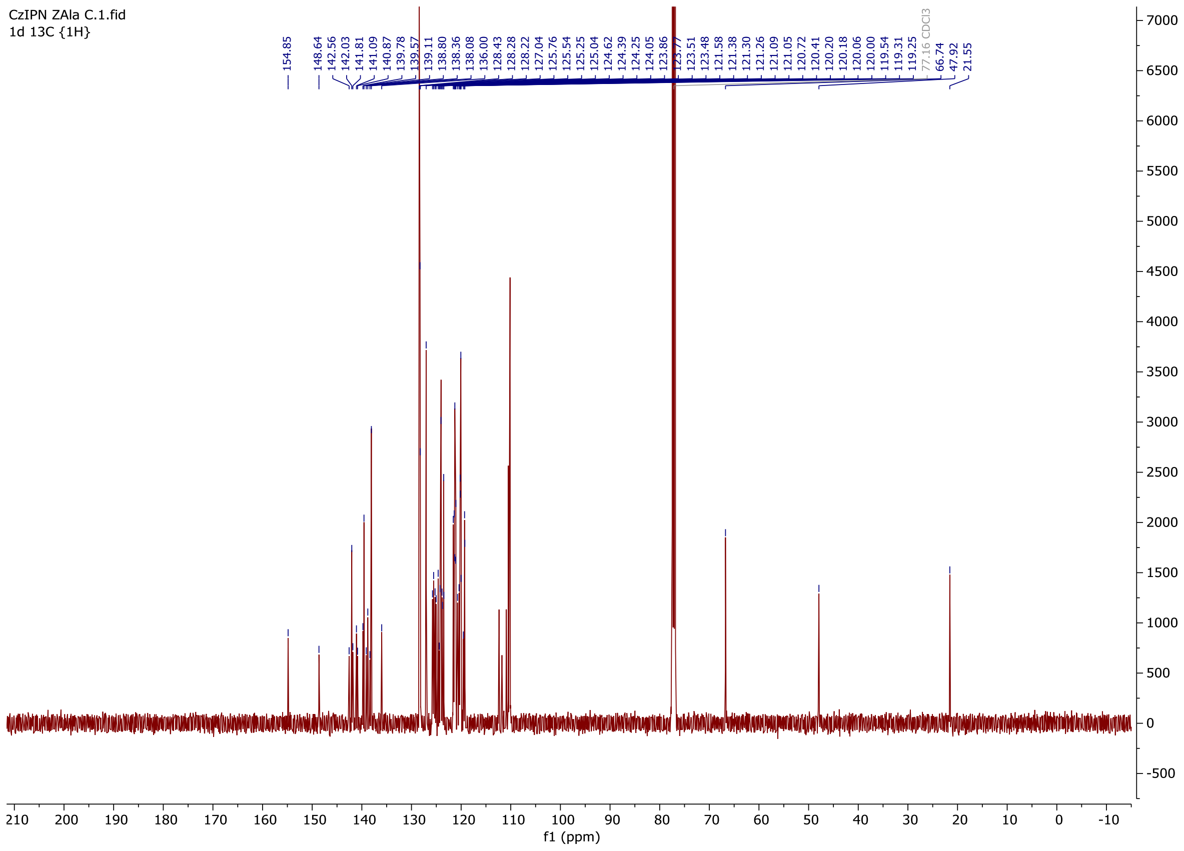
CzIPN ZAla C.1.fid
1d 13C {1H}
154.85
148.64
142.56
142.03
141.81
141.09
140.87
139.78
139.57
139.11
138.80
138.36
138.08
136.00
128.43
128.28
128.22
127.04
125.76
125.54
125.25
125.04
124.62
124.39
124.25
124.05
123.86
123.77
123.51
123.48
121.58
121.38
121.30
121.26
121.09
121.05
120.72
120.41
120.20
120.18
120.06
120.00
119.54
119.31
119.25
77.16 CDCl3
66.74
47.92
21.55
f1 (ppm)

HMBC

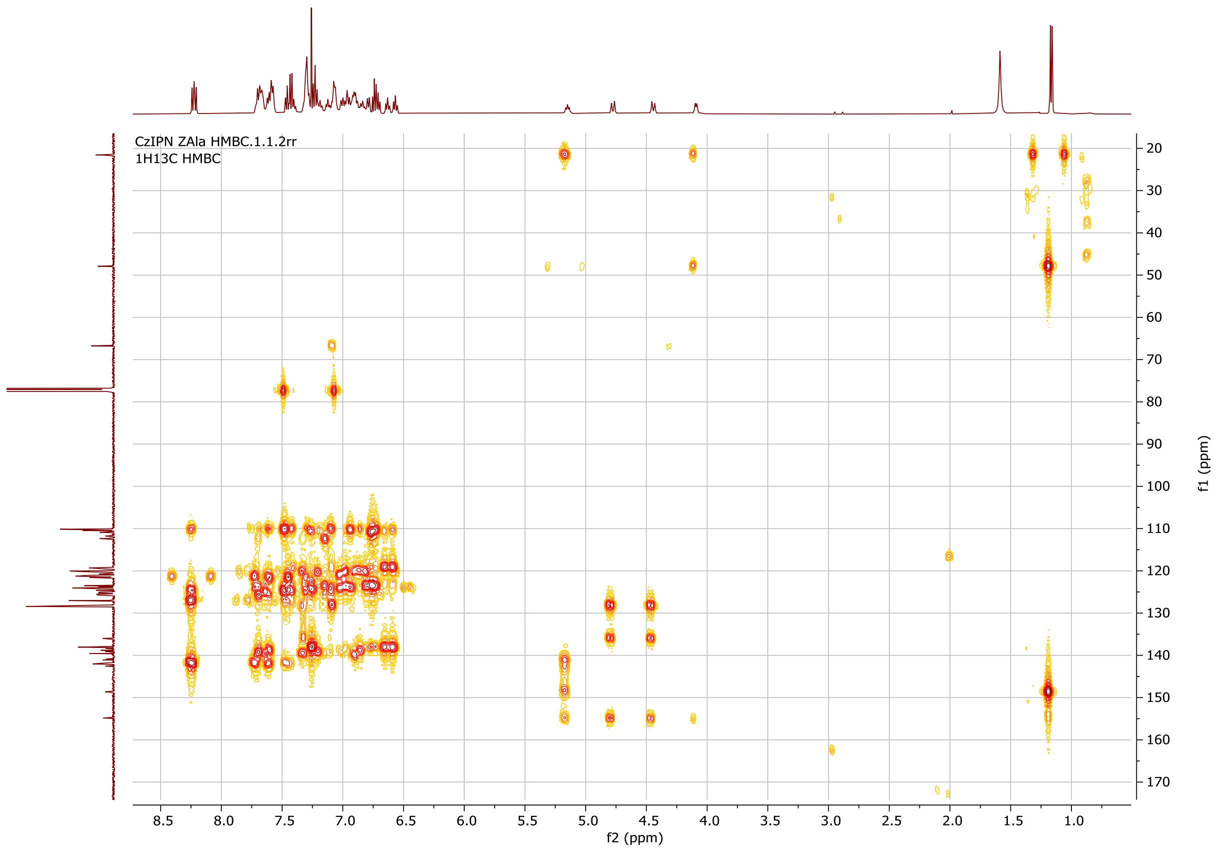
CzIPN ZAla HMBC.1.1.2rr
1H13C HMBC
f2 (ppm)
f1 (ppm)

Zoom-in

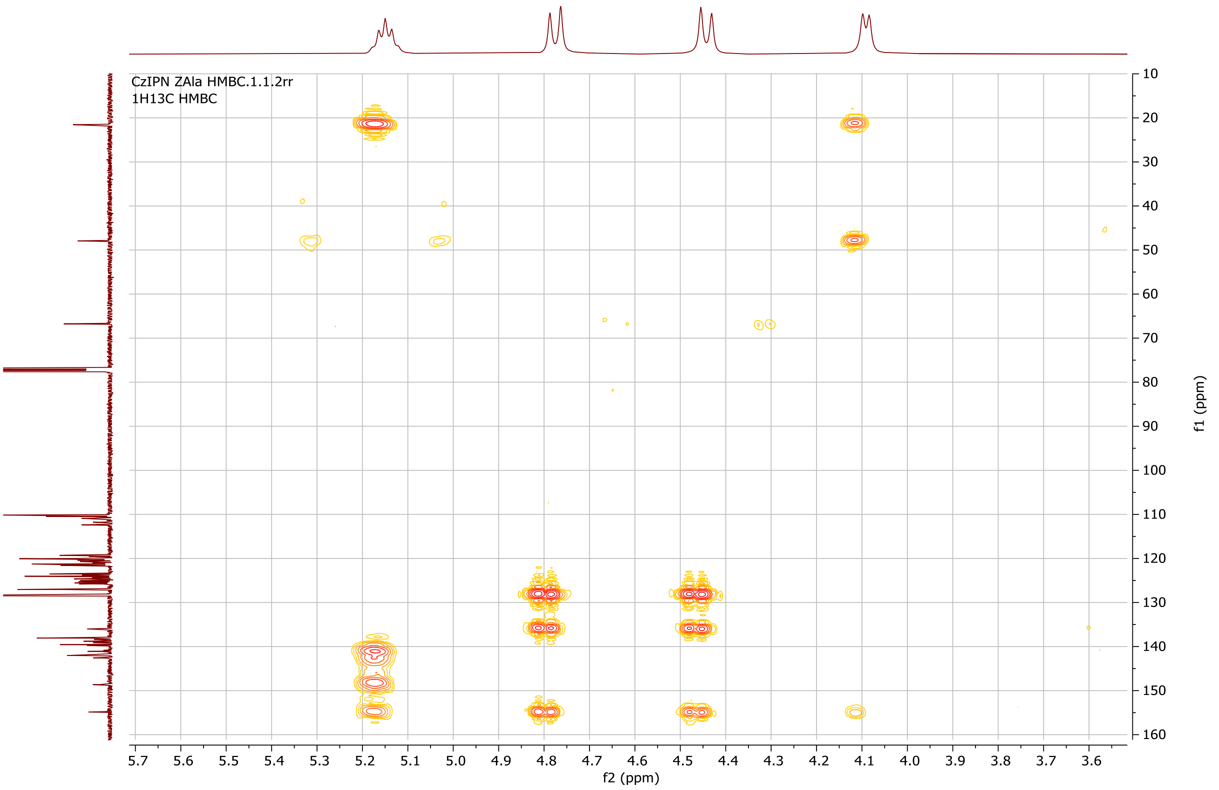

AcHFGP CzBN **3aa** **^{1}H NMR (500 MHz, DMSO) 90 °C**

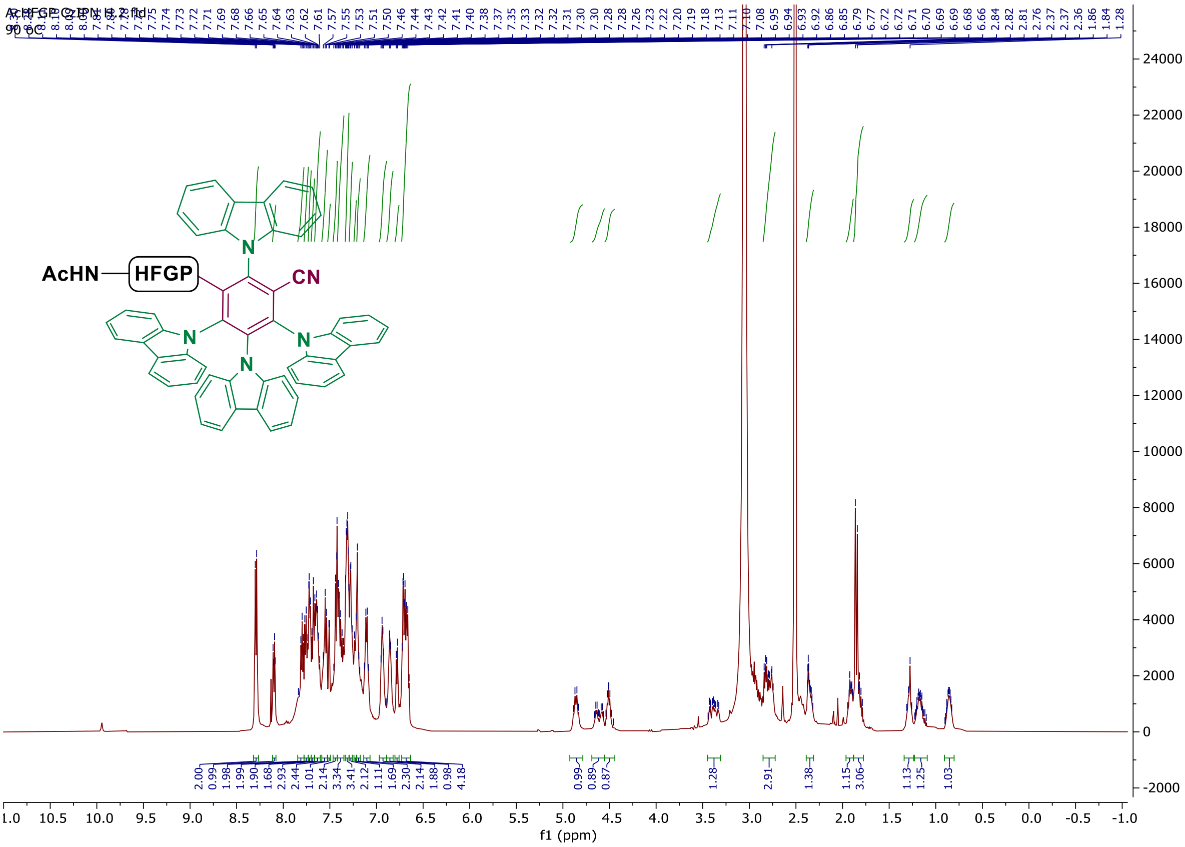

^{13}C NMR (126 MHz, DMSO) 60 °C

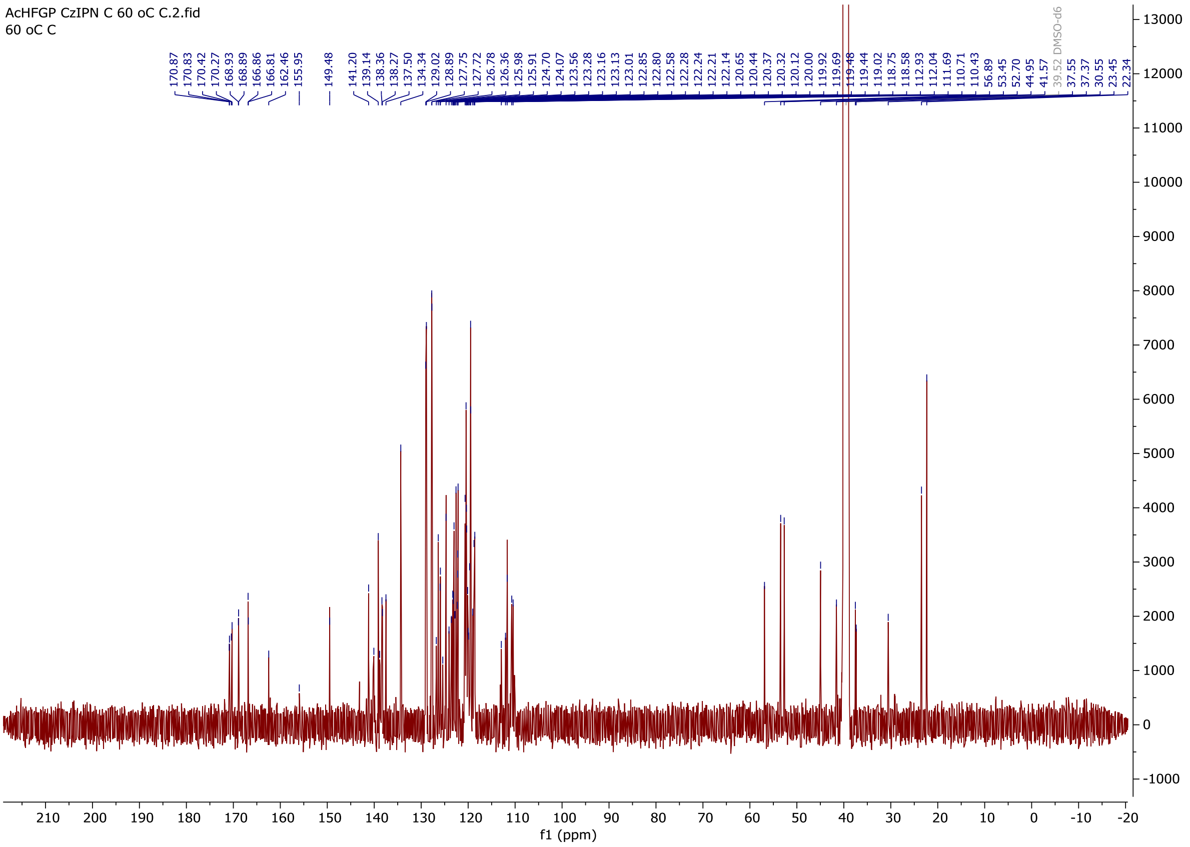

NFGP 4CzBN **3ea** ^{1}**H NMR (500 MHz, DMSO) room temperature**

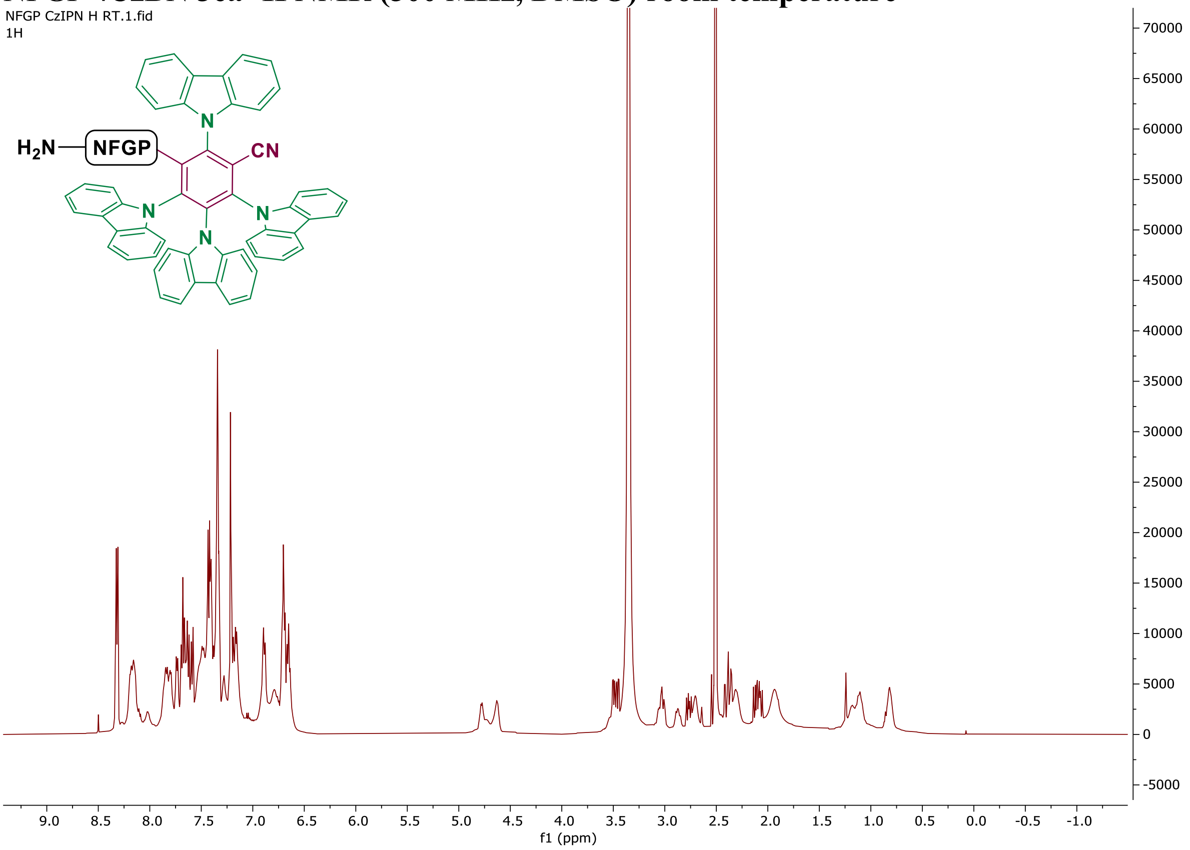

^{1}**H NMR (500 MHz, DMSO) 90 °C**

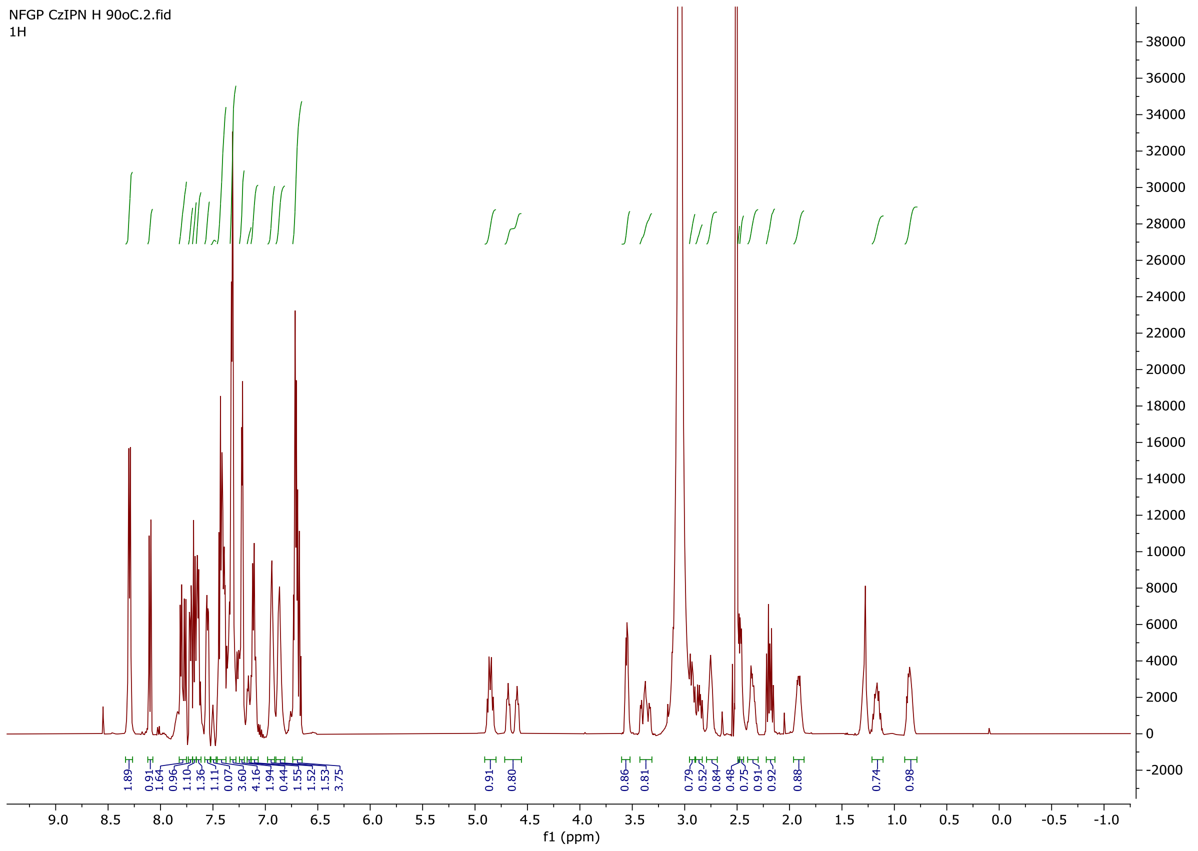

3CzIPN Ac-Cys-OMe **6aa** **^{1}H NMR (500 MHz, $CDCl_3$)**

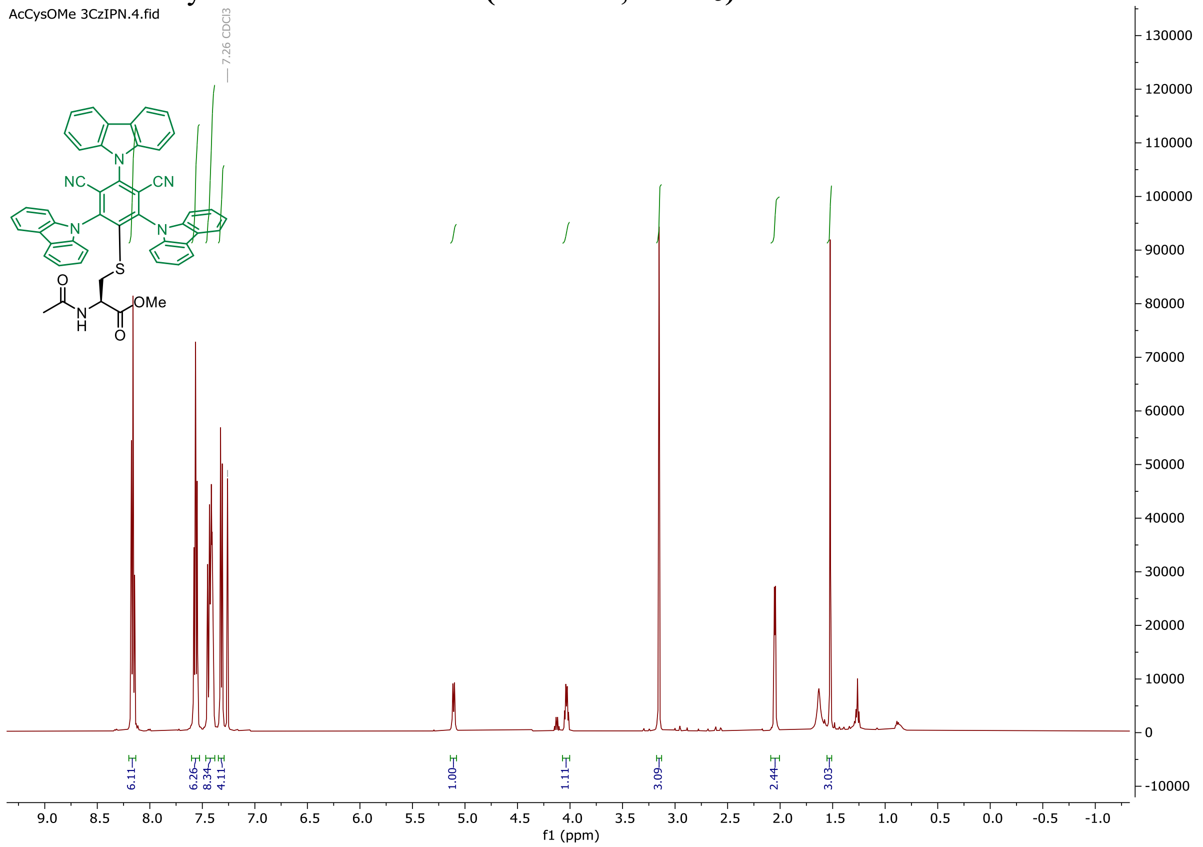

3CzIPN Ac-Cys-OMe **6aa** ^{13}C NMR (500 MHz, CDCl$_3$)

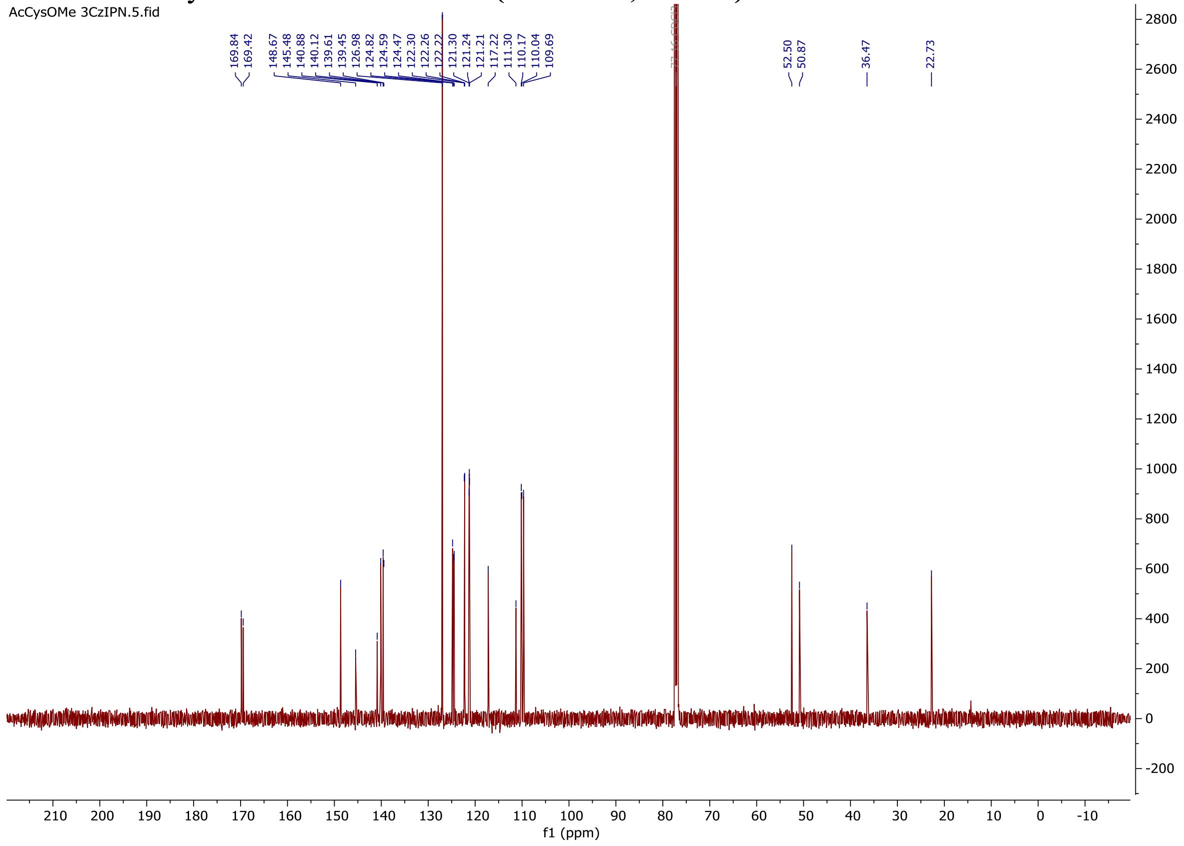

3CzIPN GSH **6ca** ^{1}H NMR (500 MHz, CDCl$_3$)

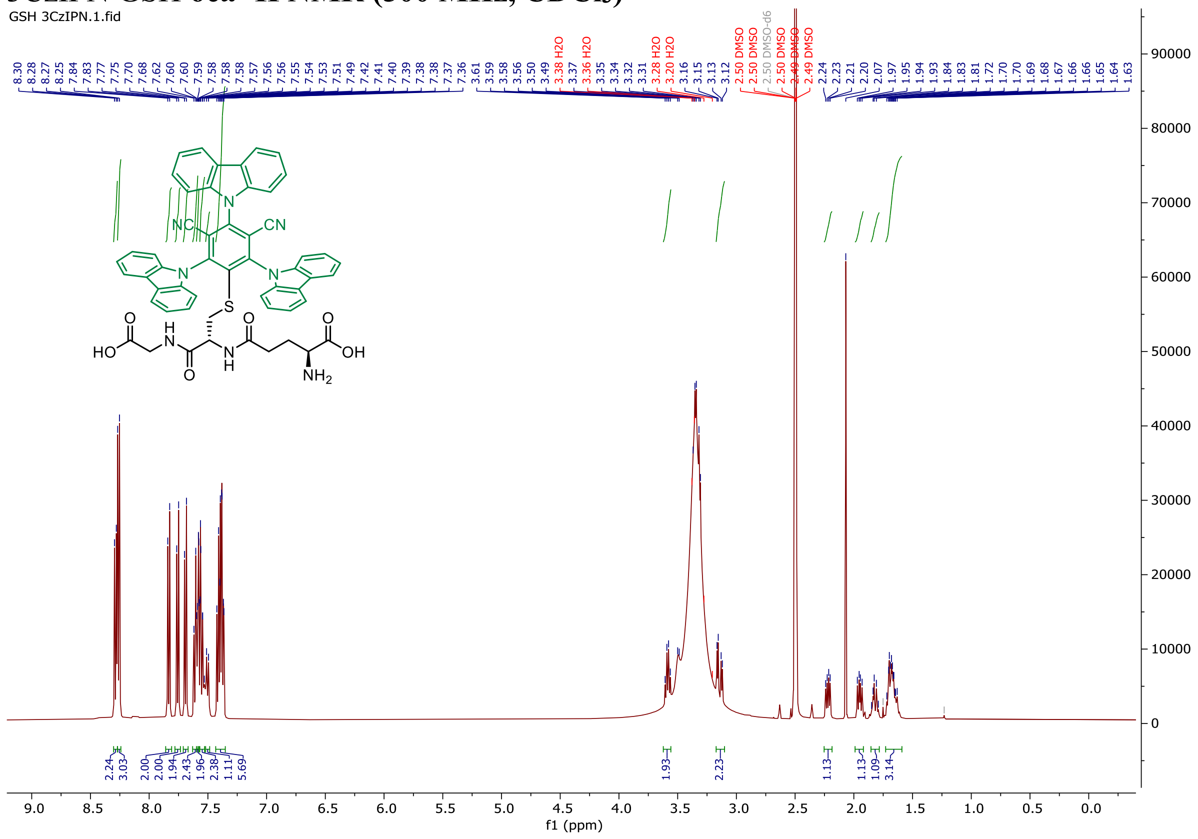

3CzIPN GSH **6ca** ^{13}C NMR (126 MHz, CDCl$_3$)

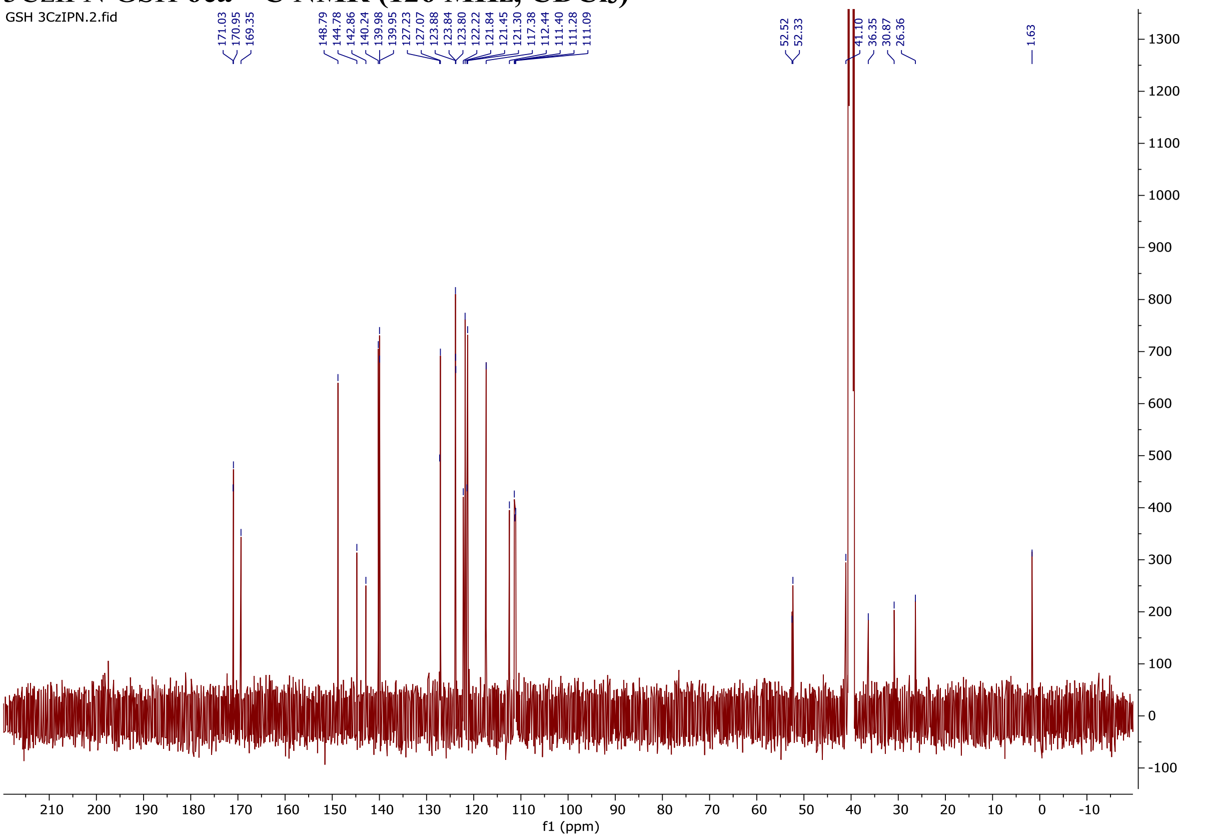

10a ^{1}H NMR (800 MHz, CDCl$_3$)

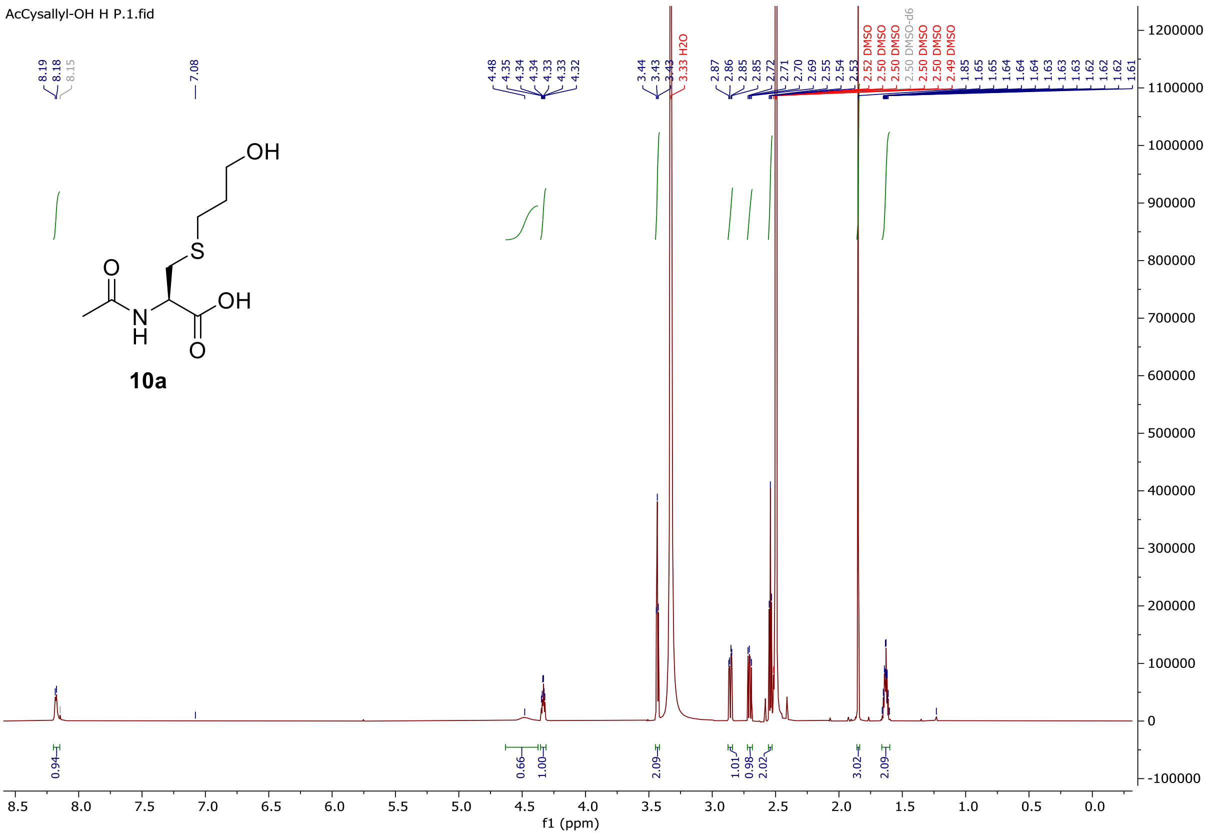

10a ^{13}C NMR (201 MHz, CDCl$_3$)

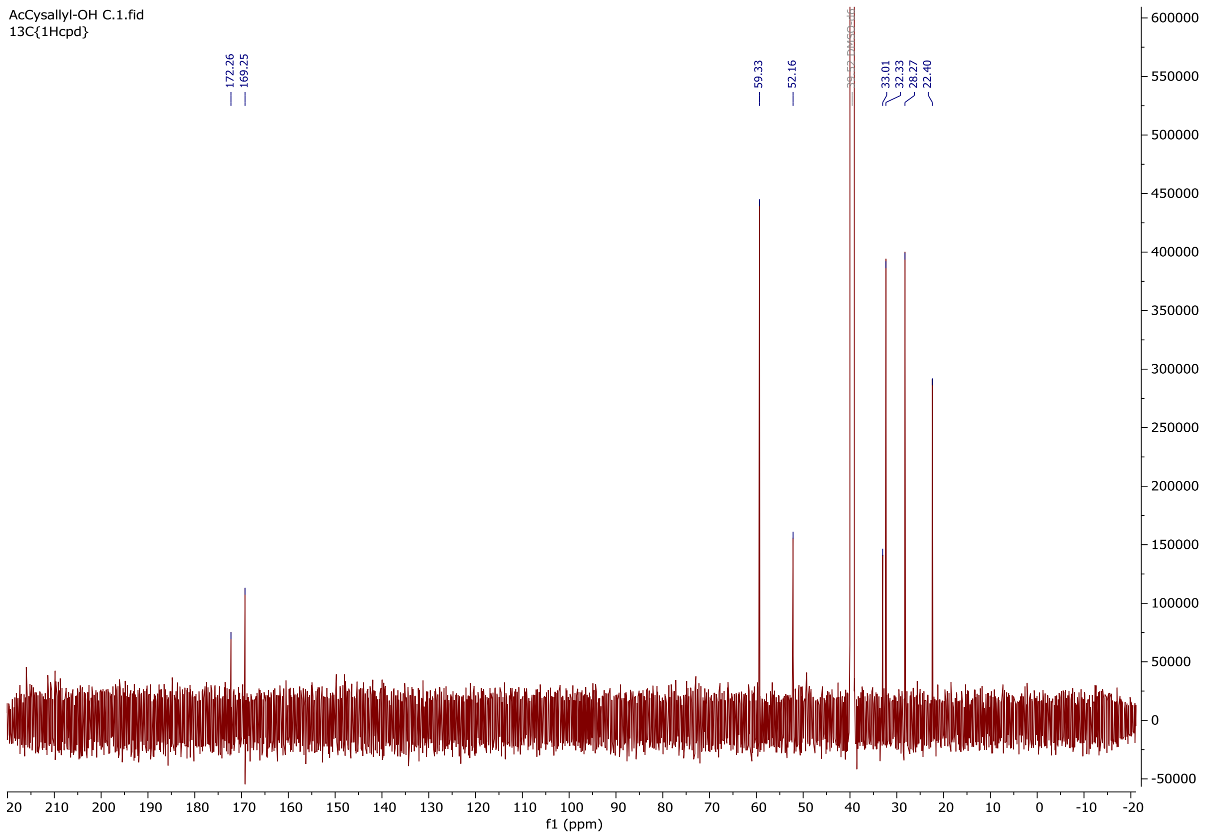

AcCysallyl-OH C.1.fid
13C{1Hcpd}
172.26
169.25
59.33
52.16
39.52 DMSO-d6
33.01
32.33
28.27
22.40
f1 (ppm)
